# Decision Aid to Technologically Enhance Shared decision making (DATES): study protocol for a randomized controlled trial

**DOI:** 10.1186/1745-6215-14-381

**Published:** 2013-11-11

**Authors:** Masahito Jimbo, Karen Kelly-Blake, Ananda Sen, Sarah T Hawley, Mack T Ruffin

**Affiliations:** 1 Department of Family Medicine, University of Michigan, Ann Arbor, MI, USA; 2 Center for Ethics and Humanities in the Life Sciences, Michigan State University, East Lansing, MI, USA; 3 Departments of Internal Medicine and Health Management and Policy, University of Michigan, Ann Arbor, MI, USA

**Keywords:** Colorectal neoplasms, Early detection of cancer, Cancer screening, Decision aids, Decision support techniques, Decision making, Shared, Health communication

## Abstract

**Background:**

Clinicians face challenges in promoting colorectal cancer screening due to multiple competing demands. A decision aid that clarifies patient preferences and improves decision quality can aid shared decision making and be effective at increasing colorectal cancer screening rates. However, exactly how such an intervention improves shared decision making is unclear. This study, funded by the National Cancer Institute, seeks to provide detailed understanding of how an interactive decision aid that elicits patient’s risks and preferences impacts patient-clinician communication and shared decision making, and ultimately colorectal cancer screening adherence.

**Methods/Design:**

This is a two-armed single-blinded randomized controlled trial with the target of 300 patients per arm. The setting is eleven community and three academic primary care practices in Metro Detroit. Patients are men and women aged between 50 and 75 years who are not up to date on colorectal cancer screening. ColoDATES Web (intervention arm), a decision aid that incorporates interactive personal risk assessment and preference clarification tools, is compared to a non-interactive website that matches ColoDATES Web in content but does not contain interactive tools (control arm). Primary outcomes are patient uptake of colorectal cancer screening; patient decision quality (knowledge, preference clarification, intent); clinician’s degree of shared decision making; and patient-clinician concordance in the screening test chosen. Secondary outcome incorporates a Structural Equation Modeling approach to understand the mechanism of the causal pathway and test the validity of the proposed conceptual model based on Theory of Planned Behavior. Clinicians and those performing the analysis are blinded to arms.

**Discussion:**

The central hypothesis is that ColoDATES Web will improve colorectal cancer screening adherence through improvement in patient behavioral factors, shared decision making between the patient and the clinician, and concordance between the patient’s and clinician’s preferred colorectal cancer screening test. The results of this study will be among the first to examine the effect of a real-time preference assessment exercise on colorectal cancer screening and mediators, and, in doing so, will shed light on the patient-clinician communication and shared decision making ‘black box’ that currently exists between the delivery of decision aids to patients and subsequent patient behavior.

**Trial Registration:**

ClinicalTrials.gov ID NCT01514786

## Background

Colorectal cancer screening (CRCS) is recommended for all average-risk United States (US) adults aged ≥ 50 years, because it reduces colorectal cancer (CRC) death and morbidity [1-6]. Unlike other countries, cancer screening in the US is almost always done within the context of patients’ office visits to their clinicians. In the US, several CRCS options are available including stool blood test, flexible sigmoidoscopy, and colonoscopy, unless the adult is at increased risk for CRC in which case the test option is limited to colonoscopy [1,3,7]. CRCS rates have shown an upward trend, with overall screening rates increasing from 20% in 1997 to nearly 65% in 2010 [8]. However, millions of eligible people remain unscreened by any method [9,10]. No strong evidence exists that favors one CRCS test over another for reducing CRC mortality [1,2]. The US Preventive Services Task Force, the American Cancer Society, and the National Institutes of Health State-of-the-Science Conference recommend that, in order to optimize the CRCS rate, CRCS should be based on patient preferences [1,2,9,11,12].

Patient preferences for CRCS are highly variable and relate to particular test characteristics of efficacy, sensitivity, cost, complexity, and possible harm [13-23]. Patient preference clarification does not mean merely offering choices without guidance: when the information and options are not provided within the context of their preferences and values, patients’ ability to make a decision may actually decrease [24-28]. Clinicians are encouraged to incorporate patient values when discussing CRCS and eliciting their screening choice, counselling patients to choose the CRCS test most congruous with their preferences and values [11,23,29]. Shared decision making (SDM) recognizes the central role of the patient-clinician relationship in helping patients make such decisions [29,30]. However, SDM requires more time and resources than most clinicians have for a single issue, especially with multiple, competing agendas [31-34]. Also, clinicians do not always correctly perceive and address those factors important to patients and may not have the training and skills to provide effective SDM [23,30,33,35-38].

Decision aids (DAs) can potentially facilitate SDM by reducing patient decisional conflict, improving patient knowledge, and stimulating patients to be more active in decision making without increasing anxiety [39]. They usually include information on the disease/condition and the associated tests/treatments, probabilities of outcomes (benefits and harms) for each test/treatment option, and some form of a values clarification exercise to help patients determine which option would best match their values. Studies utilizing DAs by video, informed consent, and analytical hierarchy process have shown small increases in CRCS [40-42]. However, an important step that has not been evaluated to date is how the patients activated by DAs subsequently communicate with their clinicians, and whether the patients and clinicians are able to engage in SDM at a level consistent with the patients’ desires. This is important, because the clinician’s recommendation has a significant impact on patients’ behavior change, including CRCS adherence [43-45].

We have developed ColoDATES Web (CW), an interactive decision aid (DA) for CRCS designed to be used prior to a clinic visit to clarify patients’ preferences, promote SDM, and increase CRCS adherence [46-48]. It was developed through extensive formative research. First, focus groups of unscreened adults aged 50 to 64 years in three communities (urban, suburban, and rural) in Michigan, USA, revealed a clear enthusiasm for something to help individuals decide among the CRCS options, and the Internet as the ideal information source [46]. Next, an Internet search of over 65 English language websites on CRCS targeting American adults revealed little factual variation, user-directed navigation without guidance, high reading level text format, lack of readily available risk assessment tool, and lack of interactive assistance to establish CRCS preference [47]. These findings led to the development of CW, which was further refined with thirty intensive individual interviews with adults using the program. Users highly rated CW’s Comparison Table of the CRCS test options that summarizes and contrasts ten key aspects of each test: frequency, preparation, sedation required, discomfort, embarrassment, inconvenience, accuracy, additional tests, risk of complications, and cost. CW provides a unique interactive Preference Clarification Tool, which helps patients determine the CRCS test option that best matches their preferences. CW was tested in a pilot randomized controlled trial, which showed that 42% of previously unscreened patients using CW underwent CRCS at six months, compared to 20% of those who used a non-interactive website (*P* = 0.035). This increase in CRCS was similar in Caucasians and African Americans; thus, CW could have higher impact in the latter group which suffers from a higher CRC burden [48]. To our knowledge, our pilot randomized controlled trial was the first published study to show that an interactive, preference-tailored DA improved CRCS adherence. However, patients who used CW did not always complete CRCS through their preferred screening test option. We surmised that the patient-clinician communication during the subsequent clinic visit affected the final choice, but the pilot trial did not directly address this issue.

**Figure 1:**
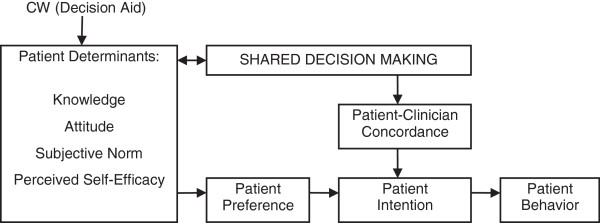
Conceptual model.

Our proposed project, Decision Aid to Technologically Enhance Shared decision making (DATES), will test CW’s effectiveness for increasing CRCS and its facilitation of SDM in primary care practices through rigorous analysis of a conceptual model adapted from the Theory of Planned Behavior [49-53] (See Figure 1). It provides a framework that clarifies the relationship between DAs and SDM [54,55]. The theory incorporates three patient determinants (attitude, subjective norm and perceived self-efficacy) that influence patient intention, which in turn influences patient behavior. In our conceptual model, we have added knowledge to patient determinants, since DAs increase knowledge [56,57]. Additionally, based on our pilot randomized controlled trial, we believe that the patient’s preference toward a specific CRCS test option is a key mediator between CW and patient intention to undergo screening for CRC. Thus, interaction with CW positively affects patient determinants, leading to the patient establishing a preference for a particular CRCS test option. This leads to greater patient intention to undergo screening and ultimately, higher CRCS rate. This process is also mediated by what occurs between the patient and the clinician during the clinic visit to discuss CRCS: the degree of SDM reached and whether the patient and the clinician agree on which CRCS test option to choose. The latter will be termed concordance.

We will test an updated version of CW that added another interactive tool, Personal Risk Assessment. The updated CW also has the reading level decreased from the previous 11th grade level to eighth grade level. It uses a renewed platform with faster speed, better graphics, and more straightforward flow, and aligns the CRCS options to those actually available in the community practice setting, namely, colonoscopy and stool blood test. It provides real-time information to the patient and the clinician during the clinic visit, making CW more applicable to real-world primary care practices. We will perform a two-armed randomized controlled trial (300 patients per arm) comparing the intervention arm using CW to the control arm using a non-interactive website, Standard Web (SW), in eleven community primary care and three university family medicine practices in Metro Detroit. The aims are as below:

### Aim 1: to measure the impact of CW on patient uptake of CRCS

H-1: patients in the intervention arm will have higher rates of CRCS adherence at the six month follow-up than those in the control arm.

### Aim 2: to evaluate the impact of CW on patient determinants, patient preference, and patient intention before the patient-clinician encounter

H2-1: patients in the intervention arm will show greater improvement from baseline in patient determinants (knowledge, attitude, subjective norm, perceived self-efficacy) compared to the control arm after the web intervention and before the patient-clinician encounter.

H2-2: patients in the intervention arm will be more likely to have a preference for a particular CRCS test option than those in the control arm after the web intervention and before the patient-clinician encounter.

H2-3: patients in the intervention arm will have higher intention to undergo CRCS than those in the control arm after the web intervention and before the patient-clinician encounter.

### Aim 3: to evaluate the impact of CW on SDM, concordance, and patient intention during and after the patient-clinician encounter

H3-1: patients in the intervention arm will experience a higher level of SDM than those in the control arm

H3-2: higher rates of concordance will be reached between the patient’s preferred CRCS test and the clinician’s recommended CRCS test in the intervention arm than those in the control arm.

H3-3: patient’s intention to undergo CRCS after the patient-clinician encounter will be predicted by the study arm, degree of SDM, concordance, and interaction between SDM and concordance.

Secondary analysis will employ a Structural Equation Modeling approach to understand the mechanism of the causal pathway and to test the validity of our proposed conceptual model [58-61]. Our study will provide detailed understanding of how an interactive DA that incorporates patient preference clarification and risk assessment, and that provides real-time information to the patient and the clinician during the clinic visit, will impact SDM and ultimately, CRCS adherence.

## Methods/Design

The study is a two-armed randomized controlled trial with a target of 300 patients per arm. The intervention arm is CW, the web-based interactive DA that contains personal risk assessment and preference clarification tools. The control arm is SW, a non-interactive website that matches CW in design and content except for the omission of interactive risk assessment and preference clarification tools. It should be noted that most US primary care practices do not routinely use web-based DAs. SW in the control arm was created to rigorously assess the effect of the interactive features in CW on the outcomes detailed in the Aims section. The study protocol received ethical approval from the University of Michigan Institutional Review Board (HUM00044733).

### Setting

Eleven community primary care (family medicine or internal medicine) and three university-based family medicine practices in Metro Detroit, Michigan, USA, have been recruited for the study. The community practices were recruited through a practice-based research network in the State of Michigan called the Great Lakes Research Into Practice Network. The university-based family medicine practices are operated by the department of the principal investigator. To ensure clinician and patient privacy, all practices signed a Data Sharing Agreement with the primary investigator’s academic institution. The demographics of the practices are detailed in Table 1.

**Table 1:** Practice demographics

**Practice type**	**Specialty**	**Location type**	**Number of clinicians**
Community-based	Internal medicine	Suburban	2
Community-based	Family medicine	Suburban	2
Community-based	Family medicine	Urban	2
Community-based	Internal medicine	Suburban	1
Community-based	Internal medicine	Suburban	3
Community-based	Internal medicine	Suburban	1
Community-based	Family medicine	Urban	3
Community-based	Family medicine	Suburban	2
Community-based	Family medicine	Suburban	1
Community-based	Internal medicine	Urban	1
Community-based	Internal medicine	Urban	1
University-based	Family medicine	Suburban	12
University-based	Family medicine	Suburban	14
University-based	Family medicine	Suburban	4

### Practice recruitment, pre-intervention practice assessment, and clinician survey

Practice recruitment was a multi-step process. First, an introductory meeting involved the practice clinicians and staff and the project team (principal investigator, project manager, research assistant). The visit had two objectives: to describe the project detail to the practice personnel, and obtain key information about the practice and clinicians. Practice and patient information data were verified through administrative personnel. The clinicians received and signed an informed consent at the time of the introductory meeting, since data will also be obtained from them. A clinician survey (see Additional file 1 for the actual survey; completion time five minutes), adapted from Murray, was distributed to all consented clinicians [62,63]. The survey obtained information about clinician’s beliefs and practices regarding CRCS, decision-making style (for example, paternalism, SDM, informed consumer) [62], and clinician demographics (for example, specialty, age, gender, race/ethnicity). During the meeting, each step of the implementation process was reviewed. Discussions focused on potential barriers to the implementation and ways to overcome them. The discussion notes were combined with the clinician survey for the final practice assessment. At the present time, the project team is making regular contacts and meetings with the practices to optimize the implementation process as it pertains to each practice [63-66]. At the completion of the study, each participating practice will receive an honorarium of US$1000 for their help.

### Patient population and eligibility criteria

The number of participants recruited per practice will be proportional to the practice size. African Americans will be overrepresented to comprise 25% of the total patient number (75 patients per arm). Patients are eligible for the study if aged between 50 and 75 years; complete their address and telephone number; are scheduled for a health maintenance examination or a chronic care visit; have not undergone a current CRCS procedure (stool blood test within the past year, flexible sigmoidoscopy within the past five years, double contrast barium enema within the past five years, computerized tomography colonography within the past five years, colonoscopy within the past ten years); have no dementia or psychosis; have no history of CRC; have no medical contraindication to CRCS; are able to read and write English; are able to give informed consent. The research assistant designated to each practice will review medical records and prepare a list of eligible patients. The practice clinicians will review the list and exclude those that should not be contacted and why. Each patient will receive an honorarium of US$25 at the completion of the study intervention.

### Potential risks and protections against them

The potential risks to the patients participating in the study are violation of their confidential information and psychological distress. The psychological risks are related to possible feelings of distress if patients using CW learn that their risk of CRC is higher than expected. Patients may also feel distress if their preferred CRCS test option and the recommended screening test by CW based on their risk assessment differ. The potential risks to the clinicians participating in the study are violation of their confidential information. Every effort will made to minimize the risk. All data including the digital audio recording will be stored in a secure location with no personal identifiers in the data. The links to personal identifiers will be stored in a separate secure location. The study participant, either the patient or the clinician, may withdraw from the study at any time point. During the clinic visit, the patient or the clinician can turn off the digital audio recorder at any time to block recording of sensitive discussions. It may be turned back on once the sensitive discussion is completed. Since all study patients will be meeting with their clinician, the clinician will directly address any psychological distress related to an individual’s participation in the study.

### Data and safety monitoring administration

The data and safety monitoring is administered by the Cancer Prevention Data and Safety Monitoring Committee within the University of Michigan Comprehensive Cancer Center. The committee reviews projects in cancer prevention/biomarker development upon the request of the principal investigator (PI). The committee reviews, makes recommendations, and acts on the following:

• Progress towards completion of the trial - recruitment and retention of study subjects

• Insufficient accrual to warrant continuation of the trial

• Evaluation of interim data analyses

• Evaluation of interim new information

• Evaluation of toxicity events including reporting of adverse events

• Timeliness of data

• Quality of data

• Ethical conduct of research

The Data and Safety Monitoring Committee is empowered with the authority to recommend a trial be suspended or terminated based upon concerns in any of the above areas of review. The committee reviews all serious adverse events and ensures that these events have been correctly reported to all institutional review boards, and that adverse events have been correctly classified as serious or not serious. The Institutional Review Board assesses the impact of these events upon the conduct of the clinical trial. The Institutional Review Board is empowered with the authority to suspend or terminate any trials for which there are concerns of toxicity that endanger human subjects. Monitoring also considers factors external to the study, such as scientific or therapeutic developments that may have an impact on the safety of the subjects or the ethics of the study. Recommendations that emanate from monitoring activities are reviewed by the PI and addressed.

### Patient recruitment and consent

Patient recruitment will occur over a period of two-and-a-half years. (Note: recruitment has started as of May 2012 and is projected to be completed by the end of September 2014.) The project team will work with practice staff to identify potentially eligible patients. At a minimum, the practice electronic billing database, scheduling system, and/or medical records will be used to accomplish this task. Medical records of patients that are identified as being potentially eligible will be reviewed to determine whether they are up to date on CRCS. If they are not up to date, they will be candidates for recruitment. The practice clinicians will review their names and exclude those they identify as meeting the exclusion criteria described above. Using the clinician-screened list of potential participants, the project team will send the Invitation Letter packets. These packets will be sent in monthly batches, on the first day of each month, to the eligible patients who are scheduled for their regular visits. The packet will contain an introductory letter with the practice letterhead and the practice clinician’s signature and an information brochure that includes Health Insurance Portability and Accountability Act privacy notice and information regarding the US$25 honorarium. Patients who do not want further contact about the study may return an enclosed postage-paid return postcard or call a toll-free telephone number. One week after the mailing, the research assistant assigned to the patient’s practice will call those patients who have not declined further contact. The research assistant will make up to five repeat phone calls to those who could not be reached initially. If patients cannot be contacted after the repeated phone calls, they will be assumed to have passively declined, and no further contact will be made. Once the patients are reached, the research assistant will briefly describe the study and ask about their interest in participation. Patients who decline will not be contacted further. For those patients who agree to participate, the research assistant will ask them to arrive at the clinic an hour earlier than the scheduled appointment. When the patient arrives at the clinic, the research assistant will present the informed consent form and describe it and the study in a private room. Those who decline to consent will be thanked and proceed to their appointment without study participation. Those who consent will be handed a laptop with wireless Internet access and proceed with the study as detailed below.

### Patient baseline survey

The participating patients will enter the website and will first complete the patient baseline survey (see Additional file 2 for the actual survey; completion time: 15 minutes). The survey will assess patient demographics; past experience with CRC screening; knowledge, attitude, subjective norm, and perceived self efficacy about CRC screening; preferred CRC screening test option; and intent to undergo screening for CRC.

### Randomization of the patients to the intervention arm and the control arm

After completing the patient baseline survey, the computer program will randomize the patients into the intervention arm and the control arm. The randomization will be stratified by practice, gender, and race to ensure there are equal proportions of patients by these factors in both arms.

### Delivery of the DA intervention (CW/SW)

**Figure 2:**
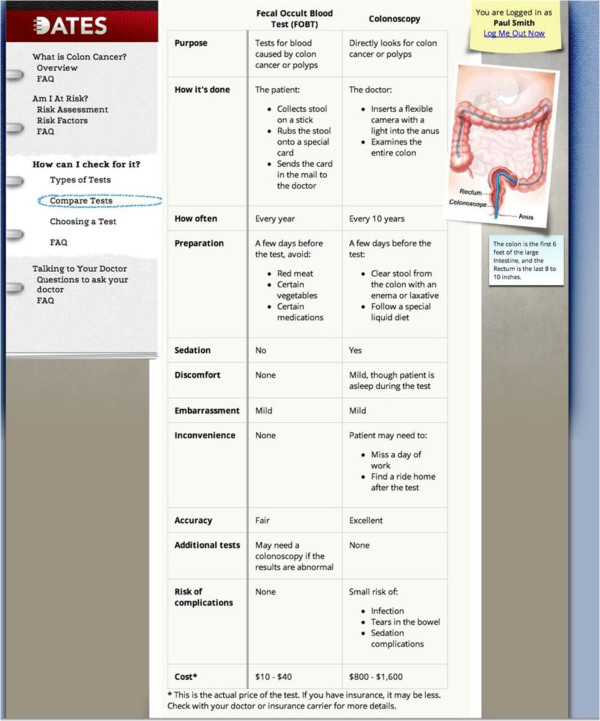
**Screen shots of ColoDATES Web (Figures **2**A-2E).**

**Figure 3:**
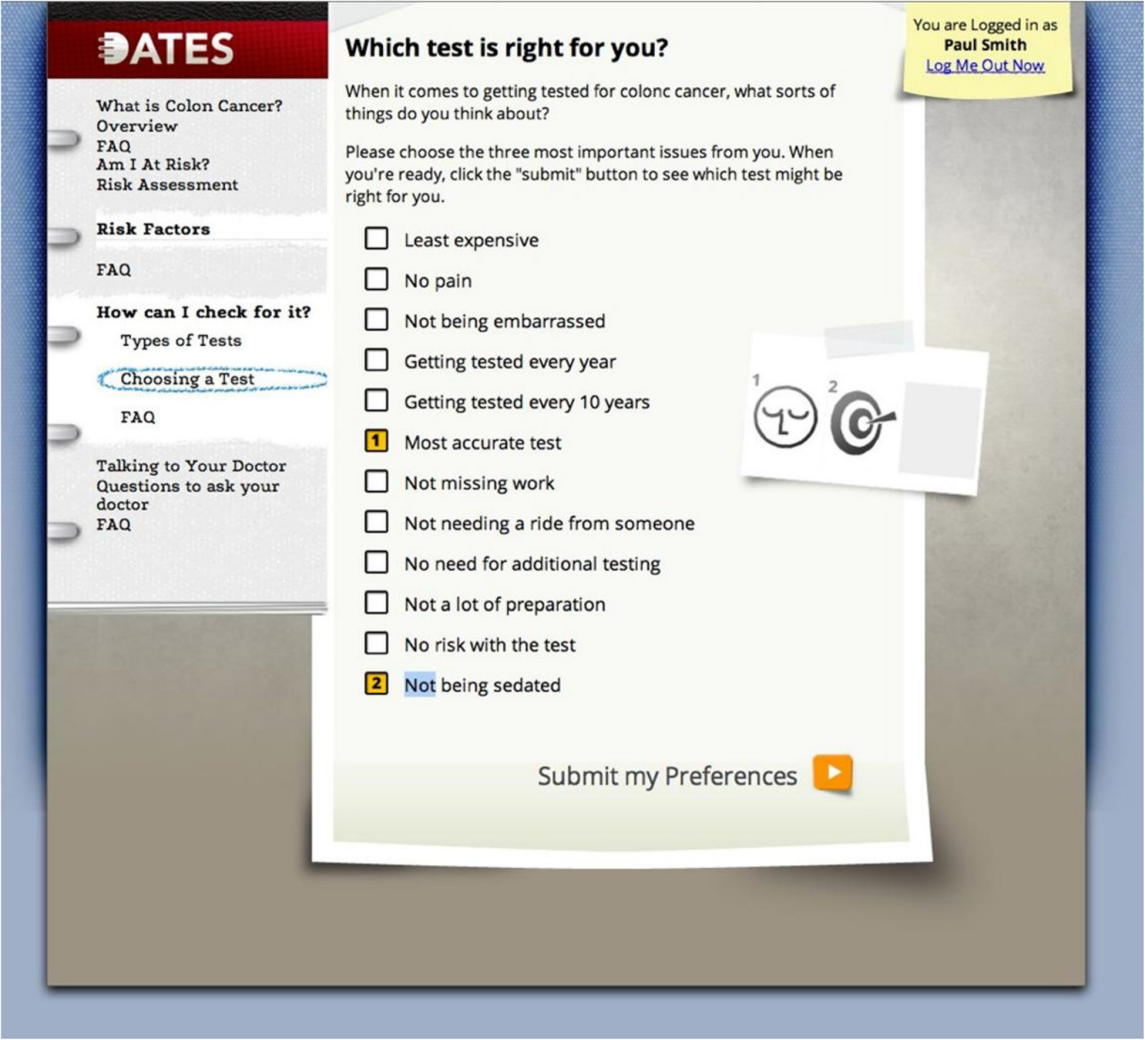
Structural equation model.

After randomization, the participating patients will enter the introductory page for CW for those randomized to the intervention arm and SW for those randomized into the Control Arm. In the intervention arm, the patients will access and work through CW. On entering CW, the patient will be guided to Overview, then Personal Risk Assessment, where they will interactively answer questions about their personal and family health history. There will be immediate feedback on whether they are ‘Average Risk’, ‘Increased Risk’, or ‘Unknown Risk’ (this occurs if the patient, who is otherwise average risk, answers ‘I don’t know’ to any of the questions in the Personal Risk Assessment). Next, the patients will be guided to access the Comparison Table providing information on CRCS tests (see Figure 2) and a Preference Clarification Tool from which they may select the three most important issues of a CRCS test (see Figure 3). The test most closely matching the three selected issues will be provided. The patients may work on the tool for as long as they wish. Once they select the matched test, a Feedback Page reviewing their test of choice in the context of their risk will be provided: ‘Average Risk’, ‘Increased Risk’, or ‘Unknown Risk’. The combination of patient risk profile and preference will help the patients to understand the details of CRCS, how each test correlates with their preference, their risk for CRC, and how their preference may or may not match the recommended test based on their risk. They will be given a final opportunity to select a CRCS test based on their risk and preferences. Once they submit their choice, a Summary Page will appear that shows them: their risk, their three most important issues, CRCS test based on the three issues, and the test they ultimately selected. Participating patients in both intervention and control arms will undergo the intervention in a private room. For patients needing help using the computer, the research assistant will provide this support but will not counsel them on CRCS. These provisions will ensure privacy, standardization, and inclusion of patients without Internet access and low computer literacy and skills.

### CW and SW data retrieval protocol

For both CW and SW, the following data will be retrieved: time from login to logoff; frequency/duration each screen page was accessed; CRCS test option selected.

### Patient post-web survey

The survey (see Additional file 3 for the actual survey; completion time: ten minutes) will be administered online immediately after the DA intervention (CW or SW). The survey will assess patient’s knowledge, attitude, subjective norm, and perceived self efficacy about CRC screening; preferred CRC screening test option; and intent to get screened for CRC. It will also include manipulation-check questions to assess what components of CW were most helpful for the patients in selecting a CRCS test option.

### Linking the summary page to the patient’s clinic visit

The research assistant will have a portable printer to ensure that the Summary Page is provided to the patient and clinician prior to the appointment. Clinicians will not be aware of which arm (intervention arm or control arm) the patient will be in since patients from both arms will have a printed Summary Page, formatted in such a way that it is not clear which Summary Page constitutes the intervention arm and the control arm.

It should be noted that the clinicians are free to make their own recommendation, and should they disagree with the patient’s choice of CRCS test option, may recommend another option. This may occur when a patient prefers a CRCS test other than colonoscopy, since clinicians increasingly prefer colonoscopy for CRCS, as shown in previous studies [35,36], our preliminary survey to the primary care practices, and a recent state-wide survey of Michigan physicians [67]. However, current guidelines do not recommend colonoscopy over other CRCS options [1,3], and ultimately, due to lack of colonoscopy access, insurance, and other reasons including patient preference, a CRCS test other than colonoscopy may be selected.

### Digital audio recording of patient-clinician encounter/OPTION scale

Both intervention and control arms will undergo audio recording. The research assistant will place a small digital audio recorder in the examination room just before the patient/clinician encounter, turn it on, and leave the room. During the encounter, the patient or clinician can turn off the digital audio recorder at any time to block recording of sensitive discussions. It may be turned back on once the sensitive discussion is completed. At the end of the encounter, the research assistant will retrieve the recorder, turn it off, remove the media, record on the data sheet, and upload the data to a secure server with the corresponding code. The investigators (PI, project manager, and three co-investigators) will read all transcripts to assign an Observing Patient Involvement (OPTION) score. The OPTION scale (see Additional file 4 for the actual form) is a validated 12-item instrument considered to be the most efficient and sensitive SDM coding system for research purposes [68-71]. Because the OPTION scale focuses on a single agenda during a patient-clinician encounter, and assesses to what degree clinicians involve patients in SDM, it is well suited to assess how the patient activated by CW affects the clinician’s SDM performance. The trained observers will listen to the audio recorded portion of the visit pertaining to CRCS and assign raw scores for each of 12 constructs in SDM, which will be converted to a scale of 0 to 100, with 0 indicating minimum and 100 indicating maximum degree of SDM. The observers will be blinded to the study arm (intervention arm or control arm) assignment, patient name, clinician name, and clinical site. The audio recording occurs in both study arms; therefore, no observational bias would be introduced into the comparison of the study arm interventions.

### Patient post-encounter survey

The research assistant will administer this survey (see Additional file 5 for the actual survey; completion time: five minutes) by paper to the patient after the patient-clinician encounter. The survey will assess patient’s preferred CRCS test; whether concordance was achieved with the clinician (that is, whether the clinician's final CRCS test recommendation matched the patient’s preferred CRCS test option); whether the patient felt his/her role in deciding to get checked for CRC was just right, tilted too much towards the clinician or too much toward the patient; and intent to undergo screening for CRC.

### Administering the endpoint chart audit and survey

Six months from the patient visit, the chart will be audited by the research assistant (see Additional file 6 for the actual audit form). Information on CRCS available in the medical chart will include stool blood test, flexible sigmoidoscopy, double contrast barium enema, computerized tomography colonography, and colonoscopy. Further, we will ascertain follow-up procedures used with patients who have an abnormal CRCS test result. Patients without medical record documentation will be called to check whether or not CRCS had been done and, if so, type of test and by which clinician/practice. If the clinician/practice identified is outside of the participating clinician/practice of the study, we will obtain a release of information for medical records from these outside sites to confirm the self-report. If a release is not provided or medical records cannot confirm the self-report, the patient will be coded as not having completed CRCS. The only acceptable outcome endpoint will be a medically documented CRCS procedure. Self-report of completing CRCS with no documentation, and self-report of not completing CRCS, will be coded as no CRCS done [72-79]. Documented incomplete CRCS test (for example, unsuccessful colonoscopy, incomplete number of stool blood cards submitted) will be coded as incomplete CRCS. Thus, there will be no missing data for this outcome endpoint. In addition to ‘CRCS done’, ‘CRCS incomplete’, and ‘CRCS not done’, information on the type of CRCS test done will be collected.

### Data collection instruments

Table 2 summarizes the data collection instruments with respect to source and time point of collection. Post-web survey will include manipulation-check questions. Also, data regarding patient’s CW/SW usage will be retrieved. They will allow us to check whether the patients in the intervention arm actually used the interactive tools in CW and found them to be useful. A more detailed table that correlates each question of the surveys with the domains of the conceptual model is included in Additional file 7.

**Table 2:** Mediators and outcomes and data collection points

	**Patient survey**			**Clinician survey**	**Digital audio recording of patient visit**	**Endpoint chart audit**
	**Baseline**	**Post**-**web**	**Post**-**encounter**			
Demographic data (BRFSS)	×			×		
Past experience	×					
Knowledge	×	×				
Attitude	×	×				
Subjective norm	×	×	×			
Perceived self efficacy	×	×				
Patient preference	×	×	×			
Manipulation-check questions		×				
Clinician decision making Style				×		
Shared decision making			×		×	
Concordance			×		×	
Patient intention	×	×	×			
Action (CRCS)						×
Method of collection	web	web	paper	paper	recording	medical chart

x: Signifies that the mediators and outcomes in question were addressed in the survey, recording or audit.

### Analysis

In the year following the end of six-month chart audit (April 2015 to March 2016), we will complete data analyses to test study hypotheses.

### Aim 1: to measure the impact of CW on patient uptake of CRCS

H-1: patients in the intervention arm will have higher rates of CRCS adherence at the six month follow-up than those in the control arm.

The study arms will be compared to assure that randomization achieved similar study population with respect to age, gender, race, previous CRCS, practice site, and data completion. Medically documented completion of CRCS will be compared between the intervention arm and the control arm by means of a chi-square test (unadjusted analysis) and a logistic regression adjusting for potential predictors at baseline. The regression analysis will be performed with data at the patient level with completing CRCS (yes/no) as the outcome measure and the arm assignment (intervention arm versus control arm) as the main covariate of interest. The practice indicator will further be included as a covariate in order to account for the variation between practices. The clustering effect due to clinician will be accounted for by using a generalized estimating equations approach. Personal patient-level background information (for example, age, race, education, marital status, smoking, health status, health insurance) from the patient baseline survey will be considered for inclusion in the regression model. The model will further be adjusted for information about the patient’s past experience with the CRCS procedures and the baseline level of patient determinants (for example, knowledge, attitude, subjective norm, self-efficacy) and intent. We will also adjust for OPTION score and pertinent clinician-level covariates such as the clinician’s communication style from the clinician survey. In order to understand whether there is any difference in the completion of CRCS rates between groups of patients who (a) complete the risk assessment tool only, (b) complete the preference clarification tool only, (c) complete both, and (d) complete neither, we will use a four level factor indicating this group membership as an independent variable in the regression model.

The contribution of a specific group of covariates will be measured by means of increase in generalized R-square defined as:

$$R^{2}\left(\mathrm{increase}\right)=\exp\left(-L_{1}^{2}/n\right)-\exp\left(-L_{2}^{2}/n\right)$$

where $L_{1}^{2},L_{2}^{2}$ are the likelihood ratio statistics of the smaller and larger models, respectively.

### Variable selection

In view of a potentially large number of candidates for inclusion as covariates, we will use a simple screening method as follows. Each potential covariate will be investigated for *confounding* effect by running a preliminary screening analysis with and without the covariate in the model along with the study arm and retaining the ones for the final logistic regression model, which either: (a) have a significant effect on the outcome; or (b) change the co-efficient of the study arm variable by more than 5%. *Moderating* effect of a potential covariate × will be investigated by including a study arm × interaction term in the model.

### Handling missing data

Missing covariate values for the subject-level information will be imputed using multiple imputation methods. All missing values will be imputed using the chained equation method that allows both categorical and continuous variables to be imputed together without making any multivariate joint distributional assumption [80]. Finally, we will combine the results from ten imputed datasets using Rubin’s formula [81].

### Aim 2: to evaluate the impact of CW on patient determinants, patient preference, and patient intention before the patient-clinician encounter

H2-1: patients in the intervention arm will show greater improvement from baseline in patient determinants (knowledge, attitude, subjective norm, perceived self-efficacy) compared to the control arm after the web intervention and before the patient-clinician encounter.

H2-2: patients in the intervention arm will be more likely to have a preference for a particular CRCS test option than those in the control arm after the web intervention and before the patient-clinician encounter.

H2-3: patients in the intervention arm will have higher intention to undergo CRCS than those in the control arm after the web intervention and before the patient-clinician encounter.

We will test Hypothesis H2*-*1 by means of linear regression analysis. We will use a separate model for each determinant. In each case, the post-web aggregated score for the scale under consideration will be used as the dependent variable. As before, the arm assignment (intervention arm versus control arm) will be the primary independent factor. The model will be further adjusted for the baseline score of the corresponding determinant and the patient-level demographic information.

Hypothesis H2*-*2 will be investigated by a logistic regression analysis with a dichotomous outcome indicating whether or not the subject has a clear preference for a CRCS test option in the patient post-web survey. This analysis will be undertaken only for the subgroup of subjects in either arm who did not have a clear preference at baseline. The demographic information will be used as independent variables as before.

Hypothesis H2*-*3 will be investigated using a model similar to that for Hypothesis H2*-*1, but the patient intention post-web will replace the patient determinants post-web as outcome, and the patient intention score at baseline will replace the patient determinants at baseline as independent variable.

### Aim 3: to evaluate the impact of CW on SDM, concordance, and patient intention during and after the patient-clinician encounter

H3-1: patients in the intervention arm will experience a higher level of SDM than those in the control arm.

H3-2: higher rates of concordance will be reached between the patient’s preferred CRCS test and the clinician’s recommended CRCS test in the intervention arm than those in the control arm.

H3-3: patient’s intention to undergo CRCS after the patient-clinician encounter will be predicted by the study arm, degree of SDM, concordance, and interaction between SDM and concordance.

We will test Hypothesis H3*-*1 by means of multiple linear regression with the OPTION score as the outcome. Otherwise the analytical strategy will be identical to that in aim 1. Hypothesis H3*-*2 will be investigated by a logistic regression analysis with a dichotomous outcome indicating whether or not the subject’s preferred CRCS test option matched with the clinician’s recommendation. Once again the analytical strategy will be similar to that in aim 1. To investigate the independent effect of SDM on concordance, we will include the OPTION score as a covariate in the regression model. Hypothesis H3*-*3 will be studied by a multiple linear regression analysis with the patient intention after the patient-clinician encounter (post-encounter) as the outcome. The primary predictors in the model will be the study arm, the OPTION score, concordance, and the interaction between the OPTION score and concordance. The interaction term allows us to investigate any differential association within SDM and intention between the concordant and the discordant groups. The model will further be controlled for the patient intention before the patient-clinician encounter. The analytical strategy will be similar to that in aim 1.

**Figure 4:**
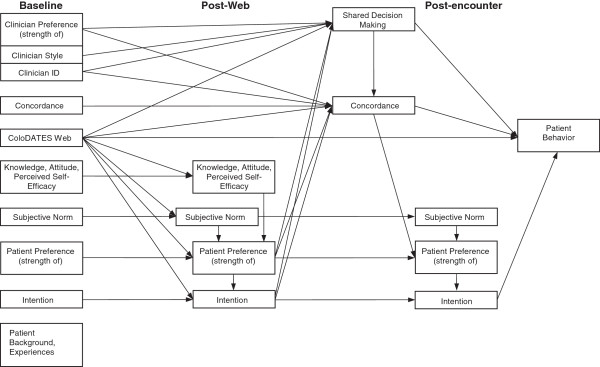
Sample selection and attrition.

Secondary analysis will be carried out by means of Structural Equation Modeling approach to estimate the parameters of the analysis model (Figure 4) that corresponds to the conceptual model (Figure 1) [49,58-61]. Specifically, a path analysis will estimate the direct and indirect effects in order to identify the significance or otherwise of specific causal relationships. These will be done by standard *t* or *F-*tests in the context of a multiple regression analysis. Testing mediation (indirect) effects will further require using Sobel’s formula [82]. Moderation is investigated by testing for significance of hypothesized interaction terms or interaction effects that are identified during the initial screening. The analysis model will have several variables: subjective norm, perceived self efficacy, patient preference, and patient intention, each measured longitudinally; concordance, measured at one time point; practice indicators, intervention arm versus control arm, clinician communication style, SDM, and patient behavior/action each measured once along with the covariates identified during the preliminary screening. Figure 4 illustrates the full complexity of the theorized causal pathway.

Structural Equation Modeling will allow us to (a) identify the significant direct and indirect effects in specific causal relationships and (b) test the plausibility of the overall model or parts thereof. In the unlikely event that the aims are not met, the information obtained will still lead us to better understand how DAs affect patient behavior and SDM between the patient and the clinician.

### Sample size and power

In aims 1, 2 and 3, we make comparisons between the intervention arm and the control arm. The effect size we wish to detect between the study arms is 15%. Based on published data [48], the estimated proportion of study participants in the control arm that will complete CRCS by six months post intervention is 10 to 15%. Based on the pilot randomized controlled trial, the proportion is slightly higher (approximately 24%). We take this higher value as the anticipated rate in the control arm after six months, since this gives more conservative (larger) sample size estimates. To detect with 90% power a 15% increase in the completion rate in the intervention arm to be significant, one needs to have 213 patients per study arm. The calculation is based on a two-sided continuity-corrected chi-square test of independent proportions with 5% type-I error rate. Since we target to recruit 300 patients in each study arm, with a rough balance across practice, race, and gender, we have good power to detect such differences in the gender subgroups as well. For example, assuming that we have 150 men in each study arm, we have 76% power to detect the effect size described herein. With 300 patients per arm, a standardized mean difference of 0.23 can be detected between the two arms for continuous outcomes with 80% power. For dichotomous outcomes such as concordance, a 15% difference between the two arms can be detected with a power of ≥ 80%, when the percentage of occurrence in the control arm ranges between 25% and 50%. Thus, we are adequately powered to detect small differences for all aims. For the Structural Equation Modeling analysis, we estimate that the conceptual model will have > 100 degrees of freedom. Assuming a sample of 600 patients, power will be > 99% for a test of the overall model. Power to reject the model will be assessed by root mean square error of approximation [83].

## Discussion

### Rationale for undergoing the trial

The results of our proposed randomized controlled trial will be among the first to examine the effect of a real-time preference assessment exercise on CRCS and mediators, and, in doing so, will shed light on the patient-clinician communication and SDM ‘black box’ that currently exists between the delivery of DAs to patients and the subsequent patient behavior (that is, CRCS adherence). Our conceptual model will elucidate not only the mechanism of how a DA influences patient behavior, but also how the DA affects the SDM between the patient and the clinician [58-61]. In addition, CW has several highly innovative features. First, CW is an interactive web-based DA rigorously developed through extensive formative research [46-48]. Second, CW assists patients in clarifying their own CRCS preferences *and* risk. To our knowledge, no previous tools have integrated interactive preference clarification and personal risk assessment to tailor CRCS recommendation, not just assessing them separately [36]. Third, CW offloads the time devoted to providing knowledge, preference clarification, and risk assessment from the clinic visit, permitting the patient and clinician to engage in SDM at a more advanced level. Fourth, it can be easily incorporated into routine clinical care.

### Randomization strategy

Randomizing at the patient level facilitates recruitment and makes the study more feasible to complete in the given timeframe. Also, patient level randomization helps in balancing across potential patient level confounders and increases statistical power to detect an intervention effect. Clinicians will be blinded to the randomization. We considered randomization at the level of the practice. Such a design would be necessary if there was a risk of contamination between the intervention arm and the control arm within a practice if randomization occurred at the patient level. This is not a concern in our proposed project for two reasons. First, patients in the intervention arm will not have access to SW, and patients in the control arm will not have access to CW. Second, clinicians will not have the resources (time, expertise, desire) to replicate the CW experience. The clinicians could give an information brochure that replicates the SW content, but this would only be additive at best to either CW in the intervention arm or SW in the control arm.

### Alternative strategies

We considered providing the DA intervention through a compact disc. However, this would add substantial cost to the development. Also, an Internet-based approach would be much more feasible for future dissemination. We also considered recruiting patients without health insurance but decided to focus our efforts on over-sampling African Americans, to test in the community practices our pilot randomized controlled trial finding that CW led to increased CRCS adherence in both Caucasians and African Americans. We decided to provide CW in English only for this study, so that we could prove its effectiveness first before expanding to other languages, such as Spanish, with their attendant costs. Finally, because our focus was on how CW affects SDM between the patient and the clinician in community practices, we needed patients who had a primary care clinician and did not recruit patients without a primary care clinician.

Since CW has interactive risk assessment and preference clarification, we considered a 2 × 2 design of interactive risk assessment (+/-) and preference clarification (+/-). We also considered a three-arm study of SW, CW with interactive preference clarification only, and CW with interactive risk assessment and preference clarification. We ultimately decided against these designs due to feasibility and cost concerns in a multi-site community practice setting and the input from the practice clinicians that CW without risk assessment had no clinical utility. We utilize manipulation-check questions to understand what particular features of CW in the intervention arm were instrumental in the differential outcomes.

Ideally, the CW would be available to patients to complete from home prior to an appointment, thereby not requiring them to come an hour before an appointment. The original proposed study design was to have patients complete the website exposure from home with telephone assistance if needed. Reviewers expressed concern that this design would lead to significantly different experiences for each patient. Some might do it weeks ahead of time leading to discussion with many others about the decision. Others would wait to the very last minute to do it. Also, without the research team verification, a person other than the intended participant may actually log in to the website. The reviewers felt it was critical that everyone have the same exposure. The research team made the change with concerns that this would impair recruitment of clinicians and patients. However, this has not been a barrier to date.

### Dissemination

The participating clinicians receive regular newsletters and Email updates about the study. Upon completion of the study and analysis of data for the primary hypothesis, the participating clinicians will receive a summary report and be invited to meet with the PI for discussion. The participating patients will receive a summary letter about the results. Updates about the findings will be posted in each participating clinical site.

The data including audiotapes will be open to other investigative teams for additional analysis by contacting the PI with a formal request. Contact information will be provided with all presentations and publications. The request form is available as Additional file 8. Each request will be reviewed by the research team and the Data and Safety Monitoring Committee for approval.

### Future directions

Once our aims are accomplished, we can use the barrier and facilitator information to move into dissemination of the intervention, and address the issues above. Different language versions of CW can be created and tested. For dissemination, CW needs to be modified to a deliverable product that can be used alone, integrated into other’s website or electronic medical record, or used with mobile devices. Also, since we will now have information on how CW affects patient-clinician communication, CW can be tested as a community-wide intervention with particular efforts to target the patients without health insurance and/or PC clinicians. The recorded patient-clinician encounter will be a rich source of data for further exploration focused on communication, SDM, and competing demands addressed in the clinic visit. We are particularly interested in further examining the characteristics of patients and clinicians whose preferred decision making style leans towards SDM. In addition, we will have data related to facilitators, barriers, and cost to implement CW in community-based primary care practices. We will be able to perform cost effective simulation of CW.

## Trial status

Patient recruitment started in May 2012 and will continue through the end of September 2014. As of 28 October 2013, the study has recruited 339 participants (target: 600).

## Abbreviations

CRC: Colorectal cancer; CRCS: Colorectal cancer screening; CW: ColoDATES Web; DA: Decision aid; DAs: Decision aids; DATES: Decision Aid to Technologically Enhance Shared decision making; PI: Principal investigator; SDM: Shared decision making; SW: Standard web.

## Competing interests

There are no competing interests for any of the authors.

## Authors’ contributions

Masahito Jimbo (MJ), MD, PhD, MPH, Karen Kelly-Blake (KK-B), PhD, Ananda Sen (AS), PhD, Sarah T Hawley (SH), PhD, MPH, Mack T Ruffin IV (MR), MD, MPH, MJ conceived of the study, created the application that succeeded in obtaining the funding from the National Cancer Institute (R01CA152413), is the principal investigator of the study, and drafted the manuscript. KK-B contributed to the development of analysis plan for patient/clinician communication and shared decision making. AS contributed to the development of the design and all other analysis plan of the study. SH contributed to the refinement of aims and recruitment process. MR developed the original ColoDATES website and contributed to the refinement of all aspects of the study. All authors read and approved the final manuscript.

## Author’s information

Masahito Jimbo (MJ), MD, PhD, MPH, Associate Professor of Family Medicine and Urology, University of Michigan, Ann Arbor, MI, USA, mjimbo@med.umich.edu, MJ is a family physician and has worked extensively on research projects in primary care practices. Having worked as a family physician in both urban and rural underserved areas, he has first-hand knowledge of how competing patient issues can crowd out important preventive and patient education issues from the patients’ visit with their physicians. Karen Kelly-Blake (KK-B), PhD, Research Associate, Center for Ethics and Humanities, Michigan State University, East Lansing, MI, USA, kkellybl@med.umich.edu, KK-B is a medical anthropologist with extensive experience in qualitative methods and shared decision making. Her professional training and background have been strongly interdisciplinary, focused on research dedicated to critically analyzing gender, racial, and age health disparities and how these relate to health care decision-making. Much of her research has been dedicated to identifying the ways patients gather information about a health issue and how they process that information to make health care decisions. Ananda Sen (AS), PhD, Research Associate Professor of Family Medicine, University of Michigan, Ann Arbor, MI, USA, anandas@med.umich.edu, AS is a biostatistician with significant experience working with the clinician investigators in the development of the design and analysis plan of community-based research projects. He has contributed extensively to the statistical analysis of all major research projects in the Department of Family Medicine at the University of Michigan. Sarah T Hawley (SH), PhD, MPH, Associate Professor of Medicine, University of Michigan, Ann Arbor, MI, USA, sarahawl@med.umich.edu, SH is a social scientist trained in health services research, with a focus on understanding and improving quality of cancer care for newly diagnosed patients through cancer survivors. She has expertise in the design and implementation of large population-based surveys of cancer patients and their clinicians. She also has expertise in the development of interactive, tailored decision tools focused on colorectal cancer screening, breast cancer treatment, and prostate cancer treatment. Mack T Ruffin IV (MR), MD, MPH, Dr. Max and Buena Lichter Research Professor of Family Medicine, University of Michigan, 1018 Fuller Street, Ann Arbor, MI 48104, USA, mruffin@med.umich.edu, MR is the Associate Chair of Research in the Department of Family Medicine at the University of Michigan. He has over 20 years of experience as a National Institutes of Health-funded investigator focused in the area of cancer prevention and early detection. He has extensive experience conducting studies, including clinical trials, in community-based practice setting.

## Supplementary Material

Additional file 1 Clinician Survey. Click here for file

Additional file 2 DATES Patient Baseline Survey. Click here for file

Additional file 3 DATES Patient Post-Intervention Survey. Click here for file

Additional file 4 OPTION Observing patient involvement. Click here for file

Additional file 5 DATES Patient Post-Encounter Survey: Patient ID. Click here for file

Additional file 6 Six-Month Chart Audit. Click here for file

Additional file 7 Domains and Data Collection Points: Actual Questions. Click here for file

Additional file 8 01. General Study Information. Click here for file
