# Supplementary material for: Decision Aid to Technologically Enhance Shared decision making (DATES): study protocol for a randomized controlled trial
Source: Trials. 2013 Nov 11;14:381. doi: 10.1186/1745-6215-14-381 (PMC3842677; doi:10.1186/1745-6215-14-381)
Supplement: Additional file 1 — Clinician Survey. [file 1745-6215-14-381-S1.pdf]

DATES Physician Survey

Physician ID#_________________ Date __/__/__ Site #_____________

Please complete the following information related to your thoughts and *usual practice* on *Colorectal Cancer (CRC)* screening. This will help us understand your views of CRC screening and develop ways in which we may facilitate the study implementation in your office.

A. YOUR BELIEFS AND PRACTICES

1. How effective or ineffective do you believe the following screening procedures are in reducing CRC mortality in average-risk patients aged 50 years and older?

		Please check one box on each line				
		Very Ineffective	Ineffective	Neither Effective or Ineffective	Effective	Very Effective
a.	Fecal Occult Blood Test (Guaiac)	☐	☐	☐	☐	☐
b.	Fecal Immunochemical Test	☐	☐	☐	☐	☐
c.	Flexible Sigmoidoscopy	☐	☐	☐	☐	☐
d.	Double Contrast Barium Enema...	☐	☐	☐	☐	☐
e.	Computer Tomography Colonography	☐	☐	☐	☐	☐
f.	Colonoscopy	☐	☐	☐	☐	☐

2. Which CRC screening test or test combination do you *most often* recommend to an asymptomatic, average-risk patient, aged 50 or older, as an initial CRC screening strategy?

(Please check just one answer.)

- ☐ Stool blood test (fecal occult blood or fecal immunochemical test) alone
- ☐ ***Either**** stool blood test alone or colonoscopy alone, based on patient's preference
- ☐ ***Both**** stool blood test and colonoscopy
- ☐ Colonoscopy alone

Either means you recommend only one test, either stool blood test or colonoscopy.

Both means you recommend that both stool blood test and colonoscopy be completed.

DATES Physician Survey

Physician ID#_________________ Date __/__/__ Site #_____________

B. DECISION MAKING: Please check off *one* box for Questions #3-6.

	Strongly Disagree	Disagree	Neither Agree nor Disagree	Agree	Strongly Agree
**3. Overall, my patients are well informed about CRC screening.**	☐	☐	☐	☐	☐
**4. My patients have a clear preference about a CRC screening test before we discuss it.**	☐	☐	☐	☐	☐
**5. My patients have a clear preference about a CRC screening test after we discuss it.**	☐	☐	☐	☐	☐
**6. I believe my patients are likely to follow through with CRC screening.**	☐	☐	☐	☐	☐

7. What role do you take when discussing CRC screening with your patients?

(Please check just one answer.)

- ☐ The patient makes all the decisions.
- ☐ The patient makes the final decision after seriously considering my opinion.
- ☐ The patient and I share responsibility for the decision.
- ☐ I make the final decision after seriously considering the patients opinion.
- ☐ I make all the decisions.

Physician ID#_________________ Date __/__/__ Site #_____________

C. PRACTICE AND PATIENT INFORMATION: Finally, we would like to ask you a few background questions about your patients, your practice, and yourself.

8. During the past 12 months, how many newly diagnosed CRC patients have you *personally* seen in your practice? An estimate is fine. __________

9. Of the above patients, what percentage was diagnosed through screening? _________%

10. During the past 12 months, how many newly diagnosed colorectal polyp patients have you *personally* seen in your practice? An estimate is fine. __________

11. Of the above patients, what percentage was diagnosed through screening? _________%

12. Do you as an individual have an affiliation with a medical school?

(Please check just one answer.)

- ☐ Yes (Name of medical school: ____)
- ☐ No

13. What is your primary medical specialty? *(Please check just one answer.)*

- ☐ Family Medicine
- ☐ General Practice
- ☐ General Internal Medicine
- ☐ Other (Please specify: ____)

14. Are you board certified in that specialty? *(Please check just one answer.)*

- ☐ Yes
- ☐ No

15. Year of Medical School Graduation: _____________

16. Year of Birth: _____________

17. Your gender is:

- ☐ Female
- ☐ Male

Physician ID#_________________ Date __/__/__ Site #_____________

18. Are you Hispanic or Latino?

- ☐ Yes
- ☐ No
- ☐ I do not know

19. Which one or more of the following would you say is your race? ***(Please check all that apply.)***

- ☐ White
- ☐ Black or African American
- ☐ American Indian or Alaska Native
- ☐ Asian
- ☐ Native Hawaiian or Other Pacific Islander
- ☐ Other

Thank you so much for your cooperation!
