# Supplementary material for: Decision Aid to Technologically Enhance Shared decision making (DATES): study protocol for a randomized controlled trial
Source: Trials. 2013 Nov 11;14:381. doi: 10.1186/1745-6215-14-381 (PMC3842677; doi:10.1186/1745-6215-14-381)
Supplement: Additional file 2 — DATES Patient Baseline Survey. [file 1745-6215-14-381-S2.pdf]

DATES Patient Baseline Survey: Patient ID #___ __________ Date __/__/__ Site #___________

A. Questions #1-2 will ask about your health.

1. Which one of the following best describes your current health? [CurrentHealth] [LooseValidation]

- ☐ Excellent [Excellent]
- ☐ Very Good [VeryGood]
- ☐ Good [Good]
- ☐ Fair [Fair]
- ☐ Poor [Poor]

2. When you make decisions about your health, who do you want to make the decision? [DecisionHealthWho] [Required]

- ☐ I want to make all the decisions. [MeAll]
- ☐ I want to make the final decision after seriously considering my doctor's opinion. [MeFinal]
- ☐ I want to have my doctor and I make the decision together. [DoctorAndMe]
- ☐ I want my doctor to make the final decision after seriously considering my opinion. [DoctorFinal]
- ☐ I want my doctor to make all the decisions. [DoctorAll]

B. Stool Blood Test

Questions #3-5 are about the stool blood test, a test to check for colon cancer. It is done at home using a set of 2 or 3 cards to determine whether your stool contains blood. You smear a sample of your fecal matter or stool on a card from 2 or 3 separate bowel movements and return the cards to be tested.

3. Before this test was described to you, had you ever heard of a stool blood test? [HearOfFOBT] [Required]

- ☐ Yes [Yes]
- ☐ No [No] **[Computer will skip to Question #6]**

4. Have you ever done a stool blood test using a home test kit? [DoneFOBT] [Required]

- ☐ Yes [Yes]
- ☐ No [No] **[Computer will skip to Question #6]**

5. How would you describe your overall experience with stool blood test? [FOBTExperience] [LooseValidation]

- ☐ Good [Good]
- ☐ Bad [Bad]
- ☐ Neither [Neither]

C. Sigmoidoscopy or Colonoscopy

Questions #6-11 are about sigmoidoscopy and colonoscopy, two other tests to check for colon cancer. Both tests examine the colon using a narrow, lighted tube that is inserted in the rectum. <u>Sigmoidoscopy</u> only examines the lower part of the colon, while <u>colonoscopy</u>

examines the entire colon. With the sigmoidoscopy, you are awake. You are able to drive yourself home. You are able to resume your normal activities.

6. Before this test was described to you, had you ever heard of sigmoidoscopy? [HearOfSig] [Required]

- ☐ Yes [Yes]
- ☐ No [No] **[Computer will skip to Question #9]**

7. Have you ever had a sigmoidoscopy? [HadSig] [Required]

- ☐ Yes [Yes]
- ☐ No [No] **[Computer will skip to Question #9]**

8. How would you describe your overall experience with sigmoidoscopy? [SigExperience] [LooseValidation]

- ☐ Good [Good]
- ☐ Bad [Bad]
- ☐ Neither [Neither]

With the colonoscopy, you are given medicine through a needle in your arm to make you sleepy. You need someone to drive you home. You may need to take the rest of your day off from your usual activities. The day before the test, you are asked to drink a lot of liquids and to take laxatives, and no solid food is permitted.

9. Before this test was described to you, had you ever heard of colonoscopy? [HeardOfColonoscopy] [Required]

- ☐ Yes [Yes]
- ☐ No [No] **[Computer will skip to Question #12]**

10. Have you ever had a colonoscopy? [HadColonoscopy] [Required]

- ☐ Yes [Yes]
- ☐ No [No] **[Computer will skip to Question #12]**

11. How would you describe your overall experience with colonoscopy? [ColonoscopyExperience] [LooseValidation]

- ☐ Good [Good]
- ☐ Bad [Bad]
- ☐ Neither [Neither]

D. Barium Enema

Questions #12-14 are about barium enema, or a lower gastrointestinal series, another test to check for colon cancer. X-rays are taken of the colon after barium or barium and air are given by enema (liquid given through the rectum). The day before the test, you are asked to drink a lot of liquids and to take laxatives, and no solid food is permitted.

12. Before this test was described to you, had you ever heard of barium enema? [HeardOfBarEn] [Required]

- ☐ Yes [Yes]
- ☐ No [No] **[Computer will skip to Question #15]**

13. Have you ever had a barium enema? [HadBarEn] [Required]

- ☐ Yes [Yes]
- ☐ No [No] **[Computer will skip to Question #15]**

14. How would you describe your overall experience with barium enema? [BarEnExperience] [LooseValidation]

- ☐ Good [Good]
- ☐ Bad [Bad]
- ☐ Neither [Neither]

E. CT Colonography

Questions #15-17 are about CT colonography, or virtual colonoscopy, another test to check for colon cancer. CT scan pictures are taken of the colon after barium or barium and air are given by enema (liquid given through the rectum). The day before the test, you are asked to drink a lot of liquids and to take laxatives, and no solid food is permitted.

15. Before this test was described to you, had you ever heard of CT colonography? [HeardOfCTCol] [Required]

- ☐ Yes [Yes]
- ☐ No [No] **[Computer will skip to Question #18]**

16. Have you ever had a CT colonography? [HadCTCol] [Required]

- ☐ Yes [Yes]
- ☐ No [No] **[Computer will skip to Question #18]**

17. How would you describe your overall experience with CT colonography? [CTColExperience] [LooseValidation]

- ☐ Good [Good]
- ☐ Bad [Bad]
- ☐ Neither [Neither]

F. For Questions #18-36, please indicate if you agree or disagree with the following general statements about colon cancer screening.

	Strongly 1Disagree [StronglyDisagree]	Disagree [Disagree]	Neither Agree nor Disagree [Neither]	Agree [Agree]	Strongly Agree [StronglyAgree]

**18.**	**Checking for colon cancer makes sense to me. [MakesSense] [LooseValidation]**	☐	☐	☐	☐	☐
**19.**	**Checking for colon cancer is an important thing for me to do. [Important] [LooseValidation]**	☐	☐	☐	☐	☐
**20.**	**Getting checked for colon cancer is an easy thing for me to do. [Easy] [LooseValidation]**	☐	☐	☐	☐	☐
**21.**	**Checking for colon cancer can help to protect my health. [ProtectHealth] [LooseValidation]**	☐	☐	☐	☐	☐
**22.**	**I am afraid of having an abnormal colon cancer screening test result. [Afraid] [LooseValidation]**	☐	☐	☐	☐	☐
**23.**	**I will be just as healthy if I avoid getting checked for colon cancer. [Avoid] [LooseValidation]**	☐	☐	☐	☐	☐
**24.**	**Checking for colon cancer is embarrassing. [Embaressing] [LooseValidation]**	☐	☐	☐	☐	☐
**25.**	**I am worried that checking for colon cancer will show that I have colon cancer. [Show] [LooseValidation]**	☐	☐	☐	☐	☐
**26.**	**The chance that I might develop colon cancer is high compared to my peers. [HighChance] [LooseValidation]**	☐	☐	☐	☐	☐
**27.**	**When colon polyps (benign growth in colon) are found and removed, colon cancer can be prevented. [Polyps] [LooseValidation]**	☐	☐	☐	☐	☐
**28.**	**When colon cancer is found early, it can be cured. [FoundEarly] [LooseValidation]**	☐	☐	☐	☐	☐
**29.**	**Checking for colon cancer is inconvenient. [Inconvenient] [LooseValidation]**	☐	☐	☐	☐	☐
**30.**	**Checking for colon cancer is painful. [Painful] [LooseValidation]**	☐	☐	☐	☐	☐
**31.**	**Having a family member with colon cancer increases a person's risk of getting colon cancer. [FamilyMember] [LooseValidation]**	☐	☐	☐	☐	☐
**32.**	**A person can have colon cancer without symptoms. [NoSymptoms] [LooseValidation]**	☐	☐	☐	☐	☐
**33.**	**Men and women are equally likely to**	☐	☐	☐	☐	☐

	**get colon cancer.** [MenWomenEqual] [LooseValidation]					
**34.**	**People under age 50 are more likely to get colon cancer than those over age 50.** [UnderAge50] [LooseValidation]	☐	☐	☐	☐	☐
**35.**	**In order to be most effective in finding cancer, a stool blood test must be done every year.** [StoolBloodTest] [LooseValidation]	☐	☐	☐	☐	☐
**36.**	**For people of average risk, a screening colonoscopy should be performed every 3 years if results are normal.** [Colonoscopy] [LooseValidation]	☐	☐	☐	☐	☐

G. Questions #37-39 will ask more about getting checked for colon cancer.

37. Has a doctor ever recommended that you be screened or checked for colon cancer? **[DocRecCRCS] [Required]**

- ☐ Yes [Yes]
- ☐ No [No]

38. Which test would you *want to have* to check for colon cancer? [WhichTestWant] [Required]
Please check one:

- ☐ Stool Blood Test [FOBT]
- ☐ Colonoscopy [Colonoscopy]
- ☐ I am fine with doing either test [NoPreference]
- ☐ I am not sure which test I want to do [DK]
- ☐ I do ***not*** want to do either test [Neither]

		I will definitely not do it [Definitely WillDo]	I will not do it [WillNot]	I don't know if I will do it or not [DK]	I will do it [WillDo]	I will definitely do it [Definitely WillNot]
**39.**	**I intend to be checked for colon cancer in the next 6 months. [IntendToCheck] [Required]**	☐	☐	☐	☐	☐

H. Questions #40-43 will ask about your background.

40. Year of Birth: [BirthYear] [Required]_____________

41. Your gender is [Gender] [Required]:

- ☐ Female [Female]
- ☐ Male [Male]

42. Which one or more of the following would you say is your race? [Race] [Required] (Check all that apply)

- ☐ White [White]
- ☐ Black or African American [Black]
- ☐ American Indian or Alaska Native [AmericanIndian]
- ☐ Asian [Asian]
- ☐ Native Hawaiian or Other Pacific Islander [PacificIslander]
- ☐ Other: *please specify*: ______________________ [Other]

☐ I prefer not to answer [NoAnswer]

43. Are you Hispanic or Latino? [Hispanic] [Required] *Skip this question if [Race]==[NoAnswer]*

☐ Yes [Yes]

☐ No [No]

44. What would you like us to call you? Please type your name in the box the way you would like to see it on any materials that we give you (i.e. Mrs. Smith or Jane). [Required] [Name]

____

Thank you for answering these questions!
