# Supplementary material for: Decision Aid to Technologically Enhance Shared decision making (DATES): study protocol for a randomized controlled trial
Source: Trials. 2013 Nov 11;14:381. doi: 10.1186/1745-6215-14-381 (PMC3842677; doi:10.1186/1745-6215-14-381)
Supplement: Additional file 3 — DATES Patient Post-Intervention Survey. [file 1745-6215-14-381-S3.pdf]

DATES Patient Post-Intervention Survey: Patient ID #__________ Date __/__/__ Site #__________

A. For this first set of questions, please indicate if you agree or disagree with the following statements about the website you just used.

		Strongly Disagree [StronglyDisagree]	Disagree [Disagree]	Neither Agree nor Disagree [Neither]	Agree [Agree]	Strongly Agree [StronglyAgree]
1.	Using the website was time well spent. [TimeWellSpent] [LooseValidation]	☐	☐	☐	☐	☐
2.	The website was easy to use. [EasyToUse] [LooseValidation]	☐	☐	☐	☐	☐
3.	The website addressed my questions well. [AddressedQuestions] [Required]	☐	☐	☐	☐	☐
4.	This website helped me select a test that matches my values. [SelectTest] [Required]	☐	☐	☐	☐	☐
5.	I am confident of my decision because of the website. [ConfidentDecision] [Required]	☐	☐	☐	☐	☐
6.	I would recommend the website to a family member. [Recommend] [LooseValidation]	☐	☐	☐	☐	☐

B. For these questions, please indicate if you agree or disagree with the following general statements about colon cancer screening.

		Strongly Disagree [StronglyDisagree]	Disagree [Disagree]	Neither Agree nor Disagree [Neither]	Agree [Agree]	Strongly Agree [StronglyAgree]
7.	Checking for colon cancer makes sense to me. [MakesSense] [LooseValidation]	☐	☐	☐	☐	☐

8.	Checking for colon cancer is an important thing for me to do. [Important] [LooseValidation]	☐	☐	☐	☐	☐
		Strongly Disagree [StronglyDisagree]	Disagree [Disagree]	Neither Agree nor Disagree [Neither]	Agree [Agree]	Strongly Agree [StronglyAgree]
9.	Getting checked for colon cancer is an easy thing for me to do. [Easy] [LooseValidation]	☐	☐	☐	☐	☐
10.	Checking for colon cancer can help to protect my health. [ProtectHealth] [LooseValidation]	☐	☐	☐	☐	☐
11.	I am afraid of having an abnormal colon cancer screening test result. [Afraid] [LooseValidation]	☐	☐	☐	☐	☐
12.	I will be just as healthy if I avoid getting checked for colon cancer. [Avoid] [LooseValidation]	☐	☐	☐	☐	☐
13.	Checking for colon cancer is embarrassing. [Embaressing] [LooseValidation]	☐	☐	☐	☐	☐
14.	I am worried that checking for colon cancer will show that I have colon cancer. [Show] [LooseValidation]	☐	☐	☐	☐	☐
15.	Checking for colon cancer is painful. [Painful] [LooseValidation]	☐	☐	☐	☐	☐
16.	The chance that I might develop colon cancer is high compared to my peers. [HighChance] [LooseValidation]	☐	☐	☐	☐	☐
17.	When colon polyps (benign growth in colon) are found and removed, colon cancer can be prevented. [Polyps] [LooseValidation]	☐	☐	☐	☐	☐
18.	When colon cancer is found early, it can be cured. [FoundEarly] [LooseValidation]	☐	☐	☐	☐	☐
19.	Checking for colon cancer is inconvenient. [Inconvenient]	☐	☐	☐	☐	☐

	[LooseValidation]					
20.	Having a family member with colon cancer increases a person's risk of getting colon cancer. [FamilyMember] [LooseValidation]	☐	☐	☐	☐	☐
21.	A person can have colon cancer without symptoms. [NoSymptoms] [LooseValidation]	☐	☐	☐	☐	☐
22.	Men and women are equally likely to get colon cancer. [MenWomenEqual] [LooseValidation]	☐	☐	☐	☐	☐

		Strongly Disagree [StronglyDisagree]	Disagree [Disagree]	Neither Agree nor Disagree [Neither]	Agree [Agree]	Strongly Agree [StronglyAgree]
23.	People under age 50 are more likely to get colon cancer than those over age 50. [UnderAge50] [LooseValidation]	☐	☐	☐	☐	☐
24.	In order to be most effective in finding cancer, a stool blood test must be done every year. [StoolBloodTest] [LooseValidation]	☐	☐	☐	☐	☐
25.	For people of average risk, a screening colonoscopy should be performed every 3 years if results are normal. [Colonoscopy] [LooseValidation]	☐	☐	☐	☐	☐

C. These next few questions will ask more about getting checked for colon cancer.

26. When you make the decision to get checked for colon cancer, who do you want to make the decision? [DecisionHealthWho] [Required]

- ☐ I want to make all the decisions. [MeAll]
- ☐ I want to make the final decision after seriously considering my doctor's opinion. [MeFinal]
- ☐ I want to have my doctor and I make the decision together. [DoctorAndMe]
- ☐ I want my doctor to make the final decision after seriously considering my opinion. [DoctorFinal]
- ☐ I want my doctor to make all the decisions. [DoctorAll]

27. Which test would you *want to have* to check for colon cancer? [WhichTestWant] [Required]*Please check one:*

- ☐ Stool Blood Test [FOBT]
- ☐ Colonoscopy [Colonoscopy]
- ☐ I am fine with doing either test [NoPreference]
- ☐ I am not sure which test I want to do [DK]
- ☐ I do ***not*** want to do either test [Neither]

	I will definitely not do it [Definitely WillDo]	I will not do it [WillNot]	I don't know if I will do it or not [DK]	I will do it [WillDo]	I will definitely do it [Definitely WillNot]
**28. I intend to be checked for colon cancer in the next 6 months. [IntendToCheck] [Required]**	☐	☐	☐	☐	☐

D. For this last set of questions, please tell us how desirable each issue is to you when you get checked for colon cancer. (*Please check one box* for each question.)

	Very Undesirable [VeryUndesirable]	Undesirable [Undesirable]	Neither Desirable nor Undesirable [Neither]	Desirable [Desirable]	Very Desirable [VeryDesirable]
29. Missing time from work. [MissingWork] [LooseValidation]	☐	☐	☐	☐	☐
30. Not having pain from the test. [Pain] [LooseValidation]	☐	☐	☐	☐	☐
31. Using something to clean out my colon. [CleanOutColon] [LooseValidation]	☐	☐	☐	☐	☐
32. Needing another person to drive me to and from the test. [Transportation] [LooseValidation]	☐	☐	☐	☐	☐
33. Needing to get a sedative through the vein. [Sedative] [LooseValidation]	☐	☐	☐	☐	☐
34. Getting the test done every year. [Yearly] [LooseValidation]	☐	☐	☐	☐	☐
35. Handling my stool, even indirectly. [HandlingStool] [LooseValidation]	☐	☐	☐	☐	☐
36. The accuracy of the test. [Accuracy] [LooseValidation]	☐	☐	☐	☐	☐
37. The need for another test if my test is positive. [PositiveTest] [LooseValidation]	☐	☐	☐	☐	☐
38. My out-of-pocket cost is $50 or less. [Cost] [LooseValidation]	☐	☐	☐	☐	☐

Thank you for answering these questions!
