# Supplementary material for: Decision Aid to Technologically Enhance Shared decision making (DATES): study protocol for a randomized controlled trial
Source: Trials. 2013 Nov 11;14:381. doi: 10.1186/1745-6215-14-381 (PMC3842677; doi:10.1186/1745-6215-14-381)
Supplement: Additional file 4 — OPTION Observing patient involvement. [file 1745-6215-14-381-S4.pdf]

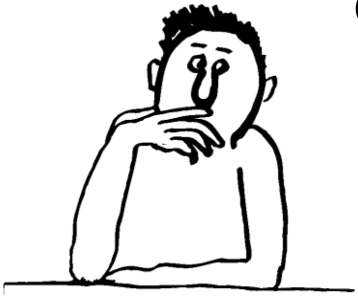

OPTION Observing patient involvement © March 2009

Date of Rating: .... (DD MM YY)

Rater Name: ....

Clinician Code: ....

Consultation Number: ....

Consultation Duration: .... (minutes. seconds)

Practitioner: .... (Age Sex)

Patient: .... (Age Sex)

Consultation Type: ☐ New ☐ Review ☐ Composite

Another Person In The Room? ☐ Yes ☐ No Who? ....

Description of Index Problem:

1. The clinician <u>draws attention</u> to an identified problem as one that requires a decision making process.

0 = No attempt to draw attention to a need for a decision making process *(there is no clarity about problems, or at least no clarity about the decisions to be taken about the problem or problems identified)*.
1 = Very brief or perfunctory attempts to draw attention to the need to embark on a decision making process.
2 = Baseline skill level: Clinician draws attention to a problem that requires a decision making process.
3 = Clinician puts emphasis on the decision making process required.
4 = The skill is exhibited to a high standard *(e.g. supplementary explanations and evidence of patient recognizing the need to engage in the process of decision making).*

2. The clinician states that there is more than one way to deal with the identified problem (‘*equipoise’*).

0 = The clinician does not state that there is more than one way of managing problems.
1 = Perfunctory attempt to convey the existence of more than one option.
2 = Baseline skill level: Clinician conveys the sense that the options are valid and need to be considered in more depth.
3 = Explains ‘equipoise’ in more detail and that options have pros and cons that need to be considered.
4 = The clinician also explains ‘why’ choices are available *(e.g. there is genuine professional uncertainly as to the ‘best’ way of managing the problem – clinical equipoise);* the skill is exhibited to a high standard.

3. The clinician <u>assesses patient’s preferred approach</u> to receiving information to assist decision making *(e.g. discussion in consultations, read printed material, assess graphical data, use videotapes or other media)*.

0 = The behaviour is not observed.
1 = A minimal attempt is made to exhibit the behaviour.
2 = Baseline skill level: Clinician asks for patient’s preferred method of receiving information.
3 = Doing this behaviour well *(e.g. states that there are many ways in which information can be conveyed; provides reading for outside of consultation).*
4 = Gives many examples of the types of information formats and media available for the patient, and then provides an opportunity for the patient to select their preferred method or methods.

4. The clinician <u>lists ‘options’</u>, which can include the choice of ‘no action’.

0 = The behaviour is not observed *(listing options is different from providing details about each option).*
1 = Minimal or perfunctory attempt is made to list options.
2 = Baseline skill level: Clinician lists options as distinct possibilities that are available (*e.g. using ‘either / or’ phrasing to describe the existence of options).*
3 = Careful listing of all possible options, including the choice of taking no action, or deferring the decision.
4 = Clinician exhibited this behaviour to a high standard.

5. The clinician <u>explains the pros and cons</u> of options to the patient *(taking ‘no action’ is an option)*.

0 = No explanation.
1 = The clinician fails to provide information about more than one option (a*ccording to the extent that each option is described*).
2 = Baseline skill level: The clinician provides details about the pros and cons of the options.
3 = The behaviour is exhibited to a good standard.
4 = The skill is exhibited to a high standard *(e.g. by description of options followed with discussion).*

6. The clinician explores the patient’s <u>expectations *(or ideas*</u>) about how the problem(s) are to be managed.

0 = No attempt to ascertain patient’s views about their expectations.
1 = Unskilled or perfunctory attempts to uncover patient’s ideas or expectations about management.
2 = Baseline skill level: The clinician explicitly asks the patient what they expected *(thought)* about the actions required to manage the problem(s). Skilled clinicians are able to explore these expectations and ideas *(using open ended questions, suggesting a range of common expectations, using pauses, being alert to verbal and physical cues and so on).*
3 = This behaviour is exhibited and leads to supplementary questions to clarify expectations or ideas *(e.g. exploration of expectations takes place)*. The behaviour is performed to a good standard.
4 = The behaviour is achieved to high standards and patient’s views are discussed and addressed.

7. The clinician explores the patient's concerns *(fears)* about how problem(s) are to be managed.

0 = No attempt to ascertain patient's views about their fears or concerns.
1= Unskilled or perfunctory attempts to uncover patient's fears or concerns about management.
2 = Baseline skill level: Clinician explicitly asks the patient to voice their fears or concerns about the possible actions required to manage the problem(s). Skilled clinicians are able to explore these fears and ideas (*using open ended questions, suggesting a range of common fears, using pauses, being alert to verbal and physical cues and so on*).
3 = Exhibits behaviour and leads to supplementary questions to clarify concerns.
4 = Achieved to high standards where patient's fears/concerns discussed and addressed.

8. The clinician checks that the patient has understood the information.

0 = No attempt to ascertain patient has understood the information.
1 = Perfunctory attempt to check patient has understood relevant information.
2 = Baseline skill level: Explicit question posed to the patient asking whether they had understood the information provided or obtained from other sources.
3 = The clinician explores nature of the patients understanding by using statements like: "I'd like to check that you have understood the information about the possible options. Would you like to let me know what you now understand about this issue?"
4 = The behaviour is observed and executed to a high standard.

9. The clinician offers the patient explicit opportunities to ask questions during decision making process.

0 = No attempt to offer opportunities to ask questions.
1 = Clinician provides pauses, or other opportunities for queries to be raised *(e.g. appropriate pace within the discourse).*
2 = Baseline skill level: Clinician explicitly asks patient to voice a question *(e.g. "Do you have any questions?").*
3 = The clinician is more specific and asks the patient whether they have questions about the options and the management of the identified problem(s).
4 = The behaviour is observed and executed to a high standard. The clinician will allow time for the patient to respond and will check if there are any other or supplementary questions.

10. The clinician elicits the patient's preferred level of involvement in decision making.

0 = No attempt made to clarify.
1 = Perfunctory or rushed attempt to elicit the patient's preferred role (active or passive) in decision making.
2 = Baseline skill level: Clinician explicitly asks patient about their preferred role.
3 = Clinician provides further explanation and continues to assess patients role preference.
4 = Clinician asks this question in a way that is easy for patient to understand and which signals that the clinician is sensitive to the decisional responsibility that is being expected of the patient.

11. The clinician indicates the need for a decision making *(or deferring)* stage (*how the decision is made is not evaluated – could be paternalistic. How the decision is made between the participants and who takes 'control' is not evaluated*).

0 = The clinician does not clearly indicate that a time has come where a decision *(or deferment)* is required.
1 = Perfunctory or unclear attempt to indicate need for a decision making state.
2 = Baseline skill level: Clear statement such as, "Perhaps it's time now to make a decision about what should be done."
3 = Exhibiting this behavior to a good standard.
4 = Clinician that achieves this task to a high standard and will have signaled the transition from consideration of information and views to one of deliberation and closure.

12. The clinician indicates the need to review the decision *(or deferment)*.

0 = No attempt to indicate a need to review or defer.
1 = Perfunctory *(e.g. that the patient should be seen again)* or rushed attempt.
2 = Baseline skill level: Clinician indicates that the patient should be seen again to re-consider the decision.
3 = The behaviour is performed to a good standard.
4 = The behaviour is observed and executed to a high standard *(e.g. makes it very explicit and encourages this approach).*

For psychometric data see: Elwyn G, Hutchings H, Edwards A, Rapport F, Wensing M, Cheung WY, Grol R. The OPTION scale: measuring the extent that clinicians involve patients in decision-making tasks. Health Expectations, 8: 34-42, 2005.

Acknowledgements: Laurie Pencille and Lilisbeth Perestelo Pérez (Mayo Clinic, Rochester, MN, USA) who worked on improving this.

decision laboratory

For further information:
Decision Laboratory
www.DecisionLaboratory.com
www.OptionInstrument.com
Cardiff University
Email: ElwynG@cardiff.ac.uk

CARDIFF UNIVERSITY
PRIFYSGOL CAERDYD
