# Supplementary material for: Decision Aid to Technologically Enhance Shared decision making (DATES): study protocol for a randomized controlled trial
Source: Trials. 2013 Nov 11;14:381. doi: 10.1186/1745-6215-14-381 (PMC3842677; doi:10.1186/1745-6215-14-381)
Supplement: Additional file 5 — DATES Patient Post-Encounter Survey: Patient ID. [file 1745-6215-14-381-S5.pdf]

DATES Patient Post-Encounter Survey: Patient ID #_____________ Date __/__/__ Site #___________

A. For Questions #1-10, please indicate if you agree or disagree with the following statements.

		Strongly Disagree						Strongly Agree
1.	My doctor* was interested in talking to me.	☐	☐	☐	☐	☐	☐	☐
2.	My doctor seemed to care if I liked him/her.	☐	☐	☐	☐	☐	☐	☐
3.	My doctor was sincere.	☐	☐	☐	☐	☐	☐	☐
4.	My doctor wanted me to trust him/her.	☐	☐	☐	☐	☐	☐	☐
5.	My doctor was willing to listen to me.	☐	☐	☐	☐	☐	☐	☐
6.	My doctor was open to my ideas.	☐	☐	☐	☐	☐	☐	☐
7.	My doctor was honest in communicating with me.	☐	☐	☐	☐	☐	☐	☐
8.	My doctor was comfortable interacting with me.	☐	☐	☐	☐	☐	☐	☐
9.	My doctor wanted to cooperate with me.	☐	☐	☐	☐	☐	☐	☐
10.	My doctor seemed nervous in my presence.	☐	☐	☐	☐	☐	☐	☐

B. Questions #11-28 will ask about your discussion with your doctor*.

(*If you saw a physician assistant or nurse practitioner, please substitute him/her for doctor.)

11. Did your doctor recommend a certain test to check for colon cancer?

☐ Yes

☐ No

12. What did your *doctor* finally recommend? *Please check ONE that applies:*

- ☐ Stool Blood Test ***only***
- ☐ Colonoscopy ***only***
- ☐ ***Both*** Stool Blood Test ***and*** Colonoscopy
- ☐ ***Either*** Stool Blood Test ***or*** Colonoscopy
- ☐ Other (please list): ____
- ☐ I DON'T REMEMBER

13. Who made the decision to get checked for colon cancer?

- ☐ I made all the decision.
- ☐ I made the final decision after seriously considering my doctor's opinion.
- ☐ My doctor and I shared responsibility for the decision.
- ☐ My doctor made the final decision after seriously considering my opinions.
- ☐ My doctor made all the decisions.

		Way too little	Too little	Just right	Too much	Way too much
**14.**	**My role in deciding to get checked for colon cancer was:**	☐	☐	☐	☐	☐

15. Which test do you *want to have* to check for colon cancer? *Please check ONE:*

- ☐ Stool Blood Test
- ☐ Colonoscopy
- ☐ I am fine with doing either test
- ☐ I am not sure which test I want to do
- ☐ I do ***not*** want to do either test

16. What influenced your answer to Question #15? *Please check ONE:*

- ☐ My doctor
- ☐ The website I just did on colon cancer screening
- ☐ ***Both*** my doctor and the website
- ☐ ***Neither*** my doctor nor the website
- ☐ Other (please list): ____

		I will definitely not do it	I will not do it	I don't know if I will do it or not	I will do it	I will definitely do it
**17.**	**I intend to be checked for colon cancer in the next 6 months.**	☐	☐	☐	☐	☐

C. Questions #18-27 will ask about the information presented in the website

DID THE WEBSITE . . .	Not at all	A little	Some-what	Quite a bit	A great deal
**18) Help you recognize that a decision to get tested for colon cancer needs to be made?**	☐	☐	☐	☐	☐
**19) Prepare you to make a better decision on which colon cancer test to pick?**	☐	☐	☐	☐	☐
**20) Help you think about the pros and cons of each colon cancer test option?**	☐	☐	☐	☐	☐
**21) Help you to think about which pros and cons about colon cancer tests are most important?**	☐	☐	☐	☐	☐
**22) Help you know that the colon cancer test decision depends on what matters most to you?**	☐	☐	☐	☐	☐
**23) Help you organize your own thoughts about your colon cancer test decision?**	☐	☐	☐	☐	☐
**24) Help you think about how involved you want to be in the colon cancer test decision?**	☐	☐	☐	☐	☐
**25) Help you identify questions you want to ask your doctor about colon cancer tests?**	☐	☐	☐	☐	☐
**26) Prepare you to talk to your doctor about what matters most to you regarding colon cancer testing?**	☐	☐	☐	☐	☐
**27) Prepare you for a follow-up visit with your doctor to discuss colon cancer testing?**	☐	☐	☐	☐	☐

D. Questions #28-29 will ask about the study coordinator

	Not at all	A little	Some-what	Quite a bit	A great deal
**28) How helpful was the study coordinator?**	☐	☐	☐	☐	☐
**29) Was having the coordinator present important?**	☐	☐	☐	☐	☐

Thank you for answering these questions!
