# Supplementary material for: Decision Aid to Technologically Enhance Shared decision making (DATES): study protocol for a randomized controlled trial
Source: Trials. 2013 Nov 11;14:381. doi: 10.1186/1745-6215-14-381 (PMC3842677; doi:10.1186/1745-6215-14-381)
Supplement: Additional file 6 — Six-Month Chart Audit. [file 1745-6215-14-381-S6.pdf]

6-Month Chart Audit

Participant ID: ____________ **Today's Date**: ________________

Coordinator Auditor: ______________ Study Visit Date: ___________CCV/HME

A. Was DATES Study Summary Sheet found in the chart? YES/NO

GET HEIGHT AND WEIGHT CLOSEST TO STUDY DATE

1. Height: _____________in / cm Date: ______/______/_________

2. Weight: _____________kg / lb Date: ______/______/_________

3. Was a screening test done?

- ☐ Yes
- ☐ No

4. How many times did the clinician see the patient from the date of the study until the date of the test (if done) or the date of the audit (if not done)? ________________________

5. Is there any notation of stool cards being given to the patient?

Yes, cards were given **No notation found** **Patient refused cards**

6. Is there any notation of a colonoscopy referral being given to the patient?

Yes, referral was given **No notation found** **Patient refused referral**

IF NO SCREENING TEST WAS DONE, CHART AUDIT IS COMPLETE

7. Which test was done? (Check all boxes that apply.)

- ☐ Stool Blood 1 → **[Complete Section A, Audit Form 1] Date: ____/_____/_____**
- ☐ Stool Blood 2 → **[Complete Section A, Audit Form 2] Date: ____/_____/_____**

- ☐ Colonoscopy 1 → **[Complete Section B, Audit Form 1] Date: ____/_____/_____**
- ☐ Colonoscopy 2 → **[Complete Section B, Audit Form 2] Date: ____/_____/_____**

- ☐ Other **[Explain]**:

____

____

Section A: Stool Blood Test, Audit Form 1

☐ NOT DONE

1. Date the results were processed: ____________ /__________/__________

2 Test type: (Check only one box.)

- ☐ FOBT- Fecal Occult Blood Test
- ☐ FIT - Fecal Immunochemical Test

3. Result: (Check only one box.)

- ☐ Negative → **[Stop]**
- ☐ Positive → **[Go to Question #4]**
- ☐ No Data → **[Stop]**
- ☐ Other **[Explain]**: ____

____

4. If the test was POSITIVE, was the patient notified? (Check only one box.)

- ☐ Yes
- ☐ No
- ☐ Unknown

5. If the test was positive, did the clinician recommend further testing?

- ☐ Yes → **[Go to Question #7]**
- ☐ No → **[Go to Question #6]**

6. If the clinician did NOT recommend further testing, why not?

- ☐ Unknown
- ☐ Patient deceased
- ☐ Patient had medical contraindications
- ☐ Patient felt to be too old
- ☐ Other reasons: ____

7. If the clinician recommended further testing, what happened?

- ☐ Colonoscopy recommended, patient refused
- ☐ Another test recommended, patient refused (Test: ___________________________)
- ☐ Colonoscopy recommended, patient not on schedule
- ☐ Another test recommended, patient not on schedule (Test: ____________________)
- ☐ Colonoscopy recommended, patient on schedule or tested
- ☐ Another test recommended, patient on schedule or tested (Test: ________________)

Section A: Stool Blood Test, Audit Form 2

☐ NOT DONE

1. Date the results were processed: ____________ /__________/__________

2 Test type: (Check only one box.)

☐ FOBT- Fecal Occult Blood Test

☐ FIT - Fecal Immunochemical Test

3. Result: (Check only one box.)

☐ Negative → **[Stop]**

☐ Positive → **[Go to Question #4]**

☐ No Data → **[Stop]**

☐ Other **[Explain]**: ____

____

4. If the test was POSITIVE, was the patient notified? (Check only one box.)

☐ Yes

☐ No

☐ Unknown

5. If the test was positive, did the clinician recommend further testing?

☐ Yes → **[Go to Question #7]**

☐ No → **[Go to Question #6]**

6. If the clinician did NOT recommend further testing, why not?

☐ Unknown

☐ Patient deceased

☐ Patient had medical contraindications

☐ Patient felt to be too old

☐ Other reasons: ____

7. If the clinician recommended further testing, what happened?

☐ Colonoscopy recommended, patient refused

☐ Another test recommended, patient refused (Test: ___________________________)

☐ Colonoscopy recommended, patient not on schedule

☐ Another test recommended, patient not on schedule (Test: ____________________)

☐ Colonoscopy recommended, patient on schedule or tested

☐ Another test recommended, patient on schedule or tested (Test: ________________)

Section B: Colonoscopy, Audit Form 1

☐ NOT DONE

1. Test Date: ____________

2. GI seen (Scope Driver): ____________

3. What documents do you have? (Check all boxes that apply.)

☐ Letter

☐ Colonoscopy Report

☐ Pathology Report

4. Was the colonoscopy aborted before the end of the procedure? (Check only one box.)

☐ No → **[Answer "Not applicable" for Question 6]**

☐ Yes → **[Complete Question 5]**

5. If aborted, what was the reason? (Check all boxes that apply.)

☐ Not applicable (not aborted)

☐ Insufficient bowel preparation

☐ Inadequate sedation (patient discomfort/intolerance of procedure)

☐ Procedural complication or adverse cardiovascular event

☐ Severe colitis, diverticulitis or obstruction

☐ Other **[Explain]**: ____

____

6. Prep result: (Check all boxes that apply.)

☐ Not applicable – condition of prep was not documented

☐ Excellent

☐ Good

☐ Fair

☐ Poor

☐ Adequate

☐ Inadequate

☐ Adequate to identify polyps > 5mm

☐ Inadequate to identify polyps > 5mm

7. Was cecum successfully reached? (Check only one box.)

☐ No

☐ Yes

8. Colonoscopy Result: (Check all boxes that apply.)

☐ Normal

☐ Hemorrhoids

☐ Polyps

☐ Diverticulitis/Diverticulosis

☐ Inflammatory Bowel Disease (Ulcerative Colitis/Crohn's Disease)

☐ Other **[Explain]**: ____
____

9. Was tissue taken? (Check only one box.)

☐ No → **[Skip to Question 11]**

☐ Yes

10. Pathology result: (Check only one box; the most abnormal finding only.)

☐ Normal tissue only (including mucosal polyps and lymphoid aggregates)

☐ Benign other **[Explain]**:
____
____

☐ Hyperplastic polyps

☐ 1-2 small (<1cm) adenomas or tubular adenomas

☐ At least one adenoma $\geq$ 1cm

☐ At least one adenoma with villous features or high grade dysplasia

☐ 3-10 adenomas

☐ More than 10 adenomas

☐ At least one sessile adenoma removed piecemeal

☐ Cancer

☐ Other **[Explain]**: ____
____

11. Recommended follow-up interval (by Scope Driver): (Check only one box.)

- ☐ < 1 year
- ☐ 1-2 years
- ☐ 3 years
- ☐ 3-5 years
- ☐ 5 years
- ☐ 5-10 years
- ☐ 10 years
- ☐ No recommendations made
- ☐ Other **[Explain]**: ____

____

12. Other recommendations by GI (by Scope Driver): (Check all boxes that apply.)

- ☐ No other recommendations given
- ☐ Repeat colonoscopy
- ☐ Stool blood test
- ☐ Flexible sigmoidoscopy (Flex Sig)
- ☐ Double Contrast Barium Enema (DCBE)
- ☐ Flex Sig + DCBE
- ☐ Other **[Explain]**: ____

____

Section B: Colonoscopy Audit Form 2

☐ NOT DONE

1. **Test Date:** ____________

2. **GI seen (Scope Driver):** ____________

3. **What documents do you have? (Check all boxes that apply.)**
   - ☐ Letter
   - ☐ Colonoscopy Report
   - ☐ Pathology Report

4. **Was the colonoscopy aborted before the end of the procedure? (Check only one box.)**
   - ☐ No → **[Answer "Not applicable" for Question 6]**
   - ☐ Yes → **[Complete Question 5]**

5. **If aborted, what was the reason? (Check all boxes that apply.)**
   - ☐ Not applicable (not aborted)
   - ☐ Insufficient bowel preparation
   - ☐ Inadequate sedation (patient discomfort/intolerance of procedure)
   - ☐ Procedural complication or adverse cardiovascular event
   - ☐ Severe colitis, diverticulitis or obstruction
   - ☐ Other **[Explain]**: ____
   ____

6. **Prep result: (Check all boxes that apply.)**
   - ☐ Not applicable – condition of prep was not documented
   - ☐ Excellent
   - ☐ Good
   - ☐ Fair
   - ☐ Poor
   - ☐ Adequate
   - ☐ Inadequate
   - ☐ Adequate to identify polyps > 5mm
   - ☐ Inadequate to identify polyps > 5mm

7. Was cecum successfully reached? (Check only one box.)

☐ No

☐ Yes

8. Colonoscopy Result: (Check all boxes that apply.)

☐ Normal

☐ Hemorrhoids

☐ Polyps

☐ Diverticulitis/Diverticulosis

☐ Inflammatory Bowel Disease (Ulcerative Colitis/Crohn's Disease)

☐ Other **[Explain]**: ____

____

9. Was tissue taken? (Check only one box.)

☐ No → **[Skip to Question 11]**

☐ Yes

10. Pathology result: (Check only one box; the most abnormal finding only.)

☐ Normal tissue only (including mucosal polyps and lymphoid aggregates)

☐ Benign other **[Explain]**:

____

____

☐ Hyperplastic polyps

☐ 1-2 small (<1cm) adenomas or tubular adenomas

☐ At least one adenoma $\geq$ 1cm

☐ At least one adenoma with villous features or high-grade dysplasia

☐ 3-10 adenomas

☐ More than 10 adenomas

☐ At least one sessile adenoma removed piecemeal

☐ Cancer

☐ Other **[Explain]**: ____

____

11. Recommended follow-up interval (by Scope Driver): (Check only one box.)

- ☐ < 1 year
- ☐ 1-2 years
- ☐ 3 years
- ☐ 3-5 years
- ☐ 5 years
- ☐ 5-10 years
- ☐ 10 years
- ☐ No recommendations made
- ☐ Other **[Explain]**: ____

____

12. Other recommendations by GI (by Scope Driver): (Check all boxes that apply.)

- ☐ No other recommendations given
- ☐ Repeat colonoscopy
- ☐ Stool blood test
- ☐ Flexible sigmoidoscopy (Flex Sig)
- ☐ Double Contrast Barium Enema (DCBE)
- ☐ Flex Sig + DCBE
- ☐ Other **[Explain]**: ____

____
