# Supplementary material for: Decision Aid to Technologically Enhance Shared decision making (DATES): study protocol for a randomized controlled trial
Source: Trials. 2013 Nov 11;14:381. doi: 10.1186/1745-6215-14-381 (PMC3842677; doi:10.1186/1745-6215-14-381)
Supplement: Additional file 7 — Domains and Data Collection Points: Actual Questions. [file 1745-6215-14-381-S7.pdf]

<Domains and Data Collection Points: Actual Questions>

Note: For the survey questions that are in statements, the participant will check ONE answer in a 5-point Likert scale: Strongly Agree/Somewhat Agree/Somewhat Disagree/Strongly Disagree.

Questions	Patient Survey				Physician Survey	Audio Recording: Patient Physician Encounter	Web Data (Para-Data)	6 Month Chart Audit
	Baseline	Post-Web	Post-Encounter	6 Month Follow-Up				
**Demographic Data (BRFSS)**	40-43				8-19			
**Height/Weight**								1, 2
**Physician Belief and Practice**								
• How effective or ineffective do you believe the following screening procedures are in reducing CRC mortality in average-risk patients aged 50 years and older?<br>a. Fecal Occult Blood Test (Guaiac)<br>b. Fecal Immunochemical Test<br>c. Flexible Sigmoidoscopy<br>d. Computer Tomography<br>e. Colonoscopy					1			
• Which CRC screening test or test combination do you *most often* recommend to an asymptomatic, average-risk patient, aged 50 years or older, as an initial CRC screening strategy?<br>☐ Stool blood test (fecal occult blood or fecal immunochemical test) alone.<br>☐ *Either** stool blood test alone or colonoscopy alone, based on patient's preference.<br>☐ *Both*** stool blood test and colonoscopy.<br>☐ Colonoscopy alone.					2			
**Physician Decision-Making**								
• Overall, my patients are well informed about CRC screening.					3			
• My patients have a clear preference about a CRC screening test before we discuss it.					4			
• My patients have a clear preference about a CRC screening test after we discuss it.					5			

• I believe my patients are likely to follow through with CRC screening.<br>• What role do you take when discussing colorectal cancer (CRC) screening with your patients?<br>☐ The patient makes all the decisions. (Informed Consumer)<br>☐ The patient makes the final decision after seriously considering my opinion.<br>☐ The patient and I share responsibility for the decision. (Shared Decision Making)<br>☐ I make the final decision after seriously considering the patient's opinion.<br>☐ I make all the decisions. (Paternalism)		6<br>7			
**Patient Health Status**<br>• Which one of the following best describes your current health?<br>☐ Excellent<br>☐ Very Good<br>☐ Good<br>☐ Fair.<br>☐ Poor	1				
**Patient Past Experience**<br>• The following questions are about the stool blood test, a test to check for colon cancer. It is done at home using a set of 2 or 3 cards to determine whether the stool contains blood. You smear a sample of your fecal matter or stool on a card from 2 or 3 separate bowel movements and return the cards to be tested.<br>Before this test was described to you, had you ever heard of a stool blood test?<br>☐ Yes<br>☐ No<br>Have you ever done a stool blood test using a home test kit?<br>☐ Yes<br>☐ No<br>How would you describe your overall experience with stool blood test?<br>☐ Good<br>☐ Bad<br>☐ Neither	3-5				

• The following questions are about sigmoidoscopy and colonoscopy, two other tests to check for colon cancer. Both tests examine the colon using a narrow, lighted tube that is inserted in the rectum. Sigmoidoscopy only examines the lower part of the colon, while colonoscopy examines the entire colon. With the sigmoidoscopy, you are awake. You are able to drive yourself home. You are able to resume your normal activities.<br>Before this test was described to you, had you ever heard of sigmoidoscopy?<br>☐ Yes<br>☐ No<br>Have you ever had a sigmoidoscopy?<br>☐ Yes<br>☐ No<br>How would you describe your overall experience with sigmoidoscopy?<br>☐ Good<br>☐ Bad<br>☐ Neither	6-8					
• With the colonoscopy, you are given medicine through a needle in your arm to make you sleepy. You need someone to drive you home. You may need to take the rest of your day off from your usual activities. The day before the test, you are asked to drink a lot of liquids and to take laxatives, and no solid food is permitted.<br>Before this test was described to you, had you ever heard of colonoscopy?<br>☐ Yes<br>☐ No<br>Have you ever had a colonoscopy?<br>☐ Yes<br>☐ No<br>How would you describe your overall experience with colonoscopy?<br>☐ Good<br>☐ Bad<br>☐ Neither	9-11					

• Barium enema, or a lower gastrointestinal series, is another test to check for colon cancer. X-rays are taken of the colon after barium or barium and air are given by enema (liquid given through the rectum). The day before the test, you are asked to drink a lot of liquids and to take laxatives, and no solid food is permitted.<br>Before this test was described to you, had you ever heard of barium enema?<br>☐ Yes<br>☐ No<br>Have you ever had a barium enema?<br>☐ Yes<br>☐ No<br>How would you describe your overall experience with barium enema?<br>☐ Good<br>☐ Bad<br>☐ Neither	12-14				
• CT colonography, or virtual colonoscopy, is another test to check for colon cancer. CT scan pictures are taken of the colon after barium or barium and air are given by enema (liquid given through the rectum). The day before the test, you are asked to drink a lot of liquids and to take laxatives, and no solid food is permitted.<br>Before this test was described to you, had you ever heard of CT colonography?<br>☐ Yes<br>☐ No<br>Have you ever had a CT colonography?<br>☐ Yes<br>☐ No<br>How would you describe your overall experience with CT colonography?<br>☐ Good<br>☐ Bad<br>☐ Neither	15-17				
**Patient Knowledge**					

• Having a family member with colon cancer increases a person’s risk of getting colon cancer.	31	20					
• When colon cancer is found early, it can be cured.	28	18					
• When colon polyps (benign growth in colon) are found and removed, colon cancer can be prevented.	27	17					
• A person can have colon cancer without symptoms.	32	21					
• Men and women are equally likely to get colon cancer.	33	22					
• People under age 50 are more likely to get colon cancer than those over age 50.	34	23					
• In order to be most effective in finding cancer, a stool blood test must be done every year.	35	24					
• For people of average risk, a screening colonoscopy should be performed every 5 years if results are normal.	36	25					
**Patient Attitude**							
• Checking for colon cancer makes sense to me.	18	7					
• Checking for colon cancer is an important thing for me to do.	19	8					
• Checking for colon cancer can help to protect my health.	21	10					
• I will be just as healthy if I avoid getting checked for colon cancer.	23	12					
• The chance that I might develop colon cancer is high compared to my peers.	26	16					
• If Participant did not get checked: What is keeping you from getting checked?			7				
• If Participant did not get checked: What would help you to get checked?			8				
**Patient Anticipated Regret**							
• I am afraid of having an abnormal colon cancer screening test result.	22	11					
• I am worried that checking for colon cancer will show that I have colon cancer.	25	14					
• Checking for colon cancer is inconvenient.	29	19					
• Checking for colon cancer is embarrassing.	24	13					

• Checking for colon cancer is painful.	30 15				
**Patient Subjective Norm**<br>• When you make the decision to get checked for health, who do you want to make the decision?<br>☐ I want to make all the decisions. (Informed Consumer)<br>☐ I want to make the final decision after seriously considering my doctor's opinion.<br>☐ I want to have my doctor and I make the decision together. (Shared Decision Making)<br>☐ I want my doctor to make the final decision after seriously considering my opinion.<br>☐ I want my doctor to make all the decisions. (Paternalism)	2				
• When you make the decision to get checked for colon cancer, who do you want to make the decision?<br>☐ I want to make all the decisions. (Informed Consumer)<br>☐ I want to make the final decision after seriously considering my doctor's opinion.<br>☐ I want to have my doctor and I make the decision together. (Shared Decision Making)<br>☐ I want my doctor to make the final decision after seriously considering my opinion.<br>☐ I want my doctor to make all the decisions. (Paternalism)	37 26				
• Please tell me how influential each of the following was in you getting checked for colon cancer<br>☐ Your doctor<br>☐ Family and friends<br>☐ The Website I saw as part of this study<br>☐ Research Coordinator support while viewing website<br>☐ Anything else (Prompt 3 times)	5A				
• Which was the most influential in you getting checked for colon cancer?<br>☐ Your doctor<br>☐ Family and friends<br>☐ The Website I saw as part of this study<br>☐ Research Coordinator support while viewing website<br>☐ Other items reported ___________________________	5B				

**Patient Perceived Self Efficacy**<br>• Getting checked for colon cancer is an easy thing for me to do.	20	9						
**Patient Website Utility**								
• Using the website was time well spent.		1						
• The website was easy to use.		2						
• The website addressed my questions well.		3						
• This website helped me select a test that matches my values.		4						
• I am confident of my decision because of the website.		5						
• I would recommend the website to a family member.		6						
• Do you remember participating in the DATES study on (appointment date)?<br>☐ Yes<br>☐ No				1				
• Do you remember the website about getting checked for colon cancer?<br>☐ Yes<br>☐ No				2				
• What about the website stood out? (Let the patient answer to an open-ended question; check off which ones the patient cited.)<br>☐ Overview<br>☐ Facts about colon cancer risk<br>☐ Assessing your risk<br>☐ Screening tests for colon cancer<br>☐ Making your preference clear<br>☐ Feedback on your choice<br>☐ Summary about your choice<br>☐ Don’t remember<br>☐ Other: ___________________________________				3				
• Did the website help you recognize that a decision to get tested for colon cancer needs to be made?			18					
• Did the website prepare you to make a better decision on which colon cancer test to pick?			19					

• Did the website help you think about the pros and cons of each colon cancer test option?	20				
• Did the website help you to think about which pros and cons about colon cancer tests are most important?	21				
• Did the website help you know that the colon cancer test decision depends on what matters most to you?	22				
• Did the website help you organize your own thoughts about your colon cancer test decision?	23				
• Did the website help you think about how involved you want to be in the colon cancer test decision?	24				
• Did the website help you identify questions you want to ask your doctor about colon cancer tests?	25				
• Did the website prepare you to talk to your doctor about what matters most to you regarding colon cancer testing?	26				
• Did the website prepare you for a follow-up visit with your doctor to discuss colon cancer testing?	27				
• How helpful was the study coordinator?	28				
• Was having the coordinator present important?	29				
**Patient Preference**					
• Missing time from work.	29				
• Not having pain from the test.	30				
• Using something to clean out my colon.	31				
• Needing another person to drive me to and from the test.	32				
• Needing to get a sedative through the vein.	33				
• Getting the test done every year.	34				
• Handling my stool.	35				
• The accuracy of the test.	36				
• The need for another test if my test is positive.	37				
• My out-of-pocket cost is $50 or less.	38				

• Which test would you *want to have* to check for colon cancer? *Please check one:*<br>☐ Stool Blood Test<br>☐ Colonoscopy<br>☐ I am fine with either test<br>☐ I am not sure which test I want to do either test<br>☐ I do not want to get checked for colon cancer	38 27 15				
**Informed Decision Making**					
• Time: total duration; duration per section (for Intervention Arm, duration of the interactive risk assessment and preference clarification tool, respectively, as well); duration per page				X	
• Number of times preference clarification tool was used				X	
**Shared Decision Making**					
• Standard OPTION (12 items; scale **0-4**; total score range of 0-48 adjusted to 0-100): We will score CRC screening discussion and ONE other topic with the most decision making process (to be rated by 2 raters; if research coordinator is a rater, she should NOT rate an encounter in which she was the coordinator and should NOT rate the encounters in the practices she is primarily responsible for).			X		
• Patient-centered OPTION (12 items: NOTE, these are Questions #1-12, and do not include the DATES-specific questions #13-15; scale **0-2**; total score range of 0-24 adjusted to 0-100): We will score CRC screening discussion and ONE other topic with the most decision making process (to be rated by 2 raters; if research coordinator is a rater, she should NOT rate an encounter in which she was the coordinator and should NOT rate the encounters in the practices she is primarily responsible for).			X		
• DATES-specific (#13 of above; scale 0-2): The patient/physician explicitly refers to the COLO-DATES Green Summary Sheet			X		
• DATES-specific (#14 of above; scale 0-2): The patient/physician explicitly refers to the website			X		
• DATES-specific (#15 of above; scale 0-2): The patient/physician explicitly refers to the decision aid (risk or preference) portion of the website			X		

• Did the physician address the importance of CRC screening? Yes/No			X		
• Did the physician address the personal risk of getting CRC? Yes/No			X		
• 5A's: Ask, Advice, Agree, Assist, Arrange			X		
• Visit time: total duration; duration (%) of CRC screening discussion; time into visit that CRC screening discussion started			X		
• Topics: total number, chronic vs. acute vs. preventive			X		
• EHR vs. paper record (need to get from the practice as a general rule, rather than assessing individual visits)			X		
• Patient's need to undress or not (need to get from the practice as a general rule, rather than assessing individual visits)			X		
• Type of visit: check-up vs. opportunistic (e.g., chronic care visit) vs. appointment made specifically for the study			X		
• Who made the decision about which test to have to check for colon cancer:<br>☐ I made all the decisions<br>☐ I made the final decision after seriously considering my doctor's opinion<br>☐ My doctor and I shared responsibility for the decision<br>☐ My doctor made the final decision after seriously considering my opinions<br>☐ My doctor made all the decisions	13				
• My role in deciding which test to have to check for colon cancer was:<br>☐ Way too little<br>☐ Too little<br>☐ Just right<br>☐ Too much<br>☐ Way too much	14				
• My doctor was interested in talking to me.	1				
• My doctor seemed to care if I liked him/her.	2				
• My doctor was sincere.	3				

• My doctor wanted me to trust him/her.	4				
• My doctor was willing to listen to me.	5				
• My doctor was open to my ideas	6				
• My doctor was honest in communicating with me.	7				
• My doctor was comfortable interacting with me.	8				
• My doctor wanted to cooperate with me.	9				
• My doctor seemed nervous in my presence.	10				
• How many times did the clinician see the patient from the date of the study until the date of the test (if done) or the date of the audit (if not done)? ___________________________________					4
• If the Participant wasn't screened: In the last six months including the day I met you for the DATES Study, did your doctor recommend that you get checked for colon cancer using stool cards or having a colonoscopy?<br>☐ Yes<br>☐ Don't know<br>☐ No	6A				
**Concordance**					
• Did your doctor recommend a certain test to check for colon cancer?<br>☐ Yes<br>☐ No	11				
• If YES, what did your *doctor* finally recommend? *Please check ONE that applies:*<br>☐ Stool Blood Test<br>☐ Colonoscopy<br>☐ Other (please list):<br>☐ I DON'T REMEMBER	12				

• What influenced your answer to Question #15? *Please check ONE:*<br>☐ My doctor<br>☐ The website I just did on colon cancer screening<br>☐ ***Both*** my doctor and the website<br>☐ ***Neither*** my doctor nor the website<br>☐ Other (please lest):<br>____________________________________			16					
**Patient Intention**<br>• I intend to be checked for colon cancer in the next 6 months.<br>• If the answer to E) is “I will do it” or “I will definitely do it”: Which test do you plan to do to get checked for colon cancer?<br>☐ Stool Blood Test<br>☐ Colonoscopy<br>☐ I am fine with doing ***either*** test<br>☐ I am ***not sure*** which test I want to do<br>☐ I do ***not*** want to do either test	39	28	17	6E				
**Patient Action (CRC Screening)**<br>• Was CRC screening done?<br>☐ Yes<br>☐ No<br>• Which test was done? (Check all boxes that apply)<br>☐ Stool Blood Test 1<br>☐ Stool Blood Test 2<br>☐ Colonoscopy 1<br>☐ Colonoscopy 2<br>☐ Other<br>• (Subsequent questions in the 6-Month Chart Audit review how the stool blood test or colonoscopy was done and followed up.)								3<br><br>5<br><br>A1-7 (Stool Blood Test); B1-12 (COL)

• Did you get checked for colorectal cancer in the last 6 months using stool cards or colonoscopy?<br>☐ Yes<br>☐ No	4				
• If Participant was screened: Which screening test did you get?<br>☐ Stool Blood Test<br>☐ Colonoscopy<br>☐ ***Both*** Stool Blood Test and Colonoscopy<br>☐ ***Neither*** Stool Blood Test nor Colonoscopy (Test:________)<br>☐ Don’t remember	5C				
• If Participant was screened: Do you know the results?<br>☐ Yes<br>☐ No	5D				
• If Participant was screened: May we ask your clinician to send you a Release of Information form?<br>☐ Yes<br>☐ No	5E				
• If Participant was screened: If colonoscopy, what was the name of the physician who did the test? __________________	5F				
• If Participant was screened: If colonoscopy, where did the colonoscopy take place? ______________________________	5G				
• If Participant was screened: Date of colonoscopy ___________	5H				
• If Participant was screened: If FOBT, what lab was used? ____	5I				
• If Participant was screened: Date cards were returned _______	5J				
• If participant wasn’t screened: Do you currently have stool cards from your doctor?<br>☐ Yes<br>☐ No<br>☐ Don’t know	6B				
• If participant wasn’t screened: Do you currently have a referral to get a colonoscopy? (If the respondent says that the clinic is going to call to set up an appointment, check “Yes”)<br>☐ Yes<br>☐ No<br>☐ Don’t know	6C				

• If participant wasn't screened: Do you currently have an appointment scheduled to have a colonoscopy?<br>☐ Yes<br>☐ No<br>☐ Don't know	6D				
