# Supplementary material for: Decision Aid to Technologically Enhance Shared decision making (DATES): study protocol for a randomized controlled trial
Source: Trials. 2013 Nov 11;14:381. doi: 10.1186/1745-6215-14-381 (PMC3842677; doi:10.1186/1745-6215-14-381)
Supplement: Additional file 8 — 01. General Study Information. [file 1745-6215-14-381-S8.htm]

Print: HUM00044733 - DATES

 


|  |
| --- |
|  |

|  |  |  |  |  |  |
| --- | --- | --- | --- | --- | --- |
| |  |  |  | | --- | --- | --- | | Date: |  |  |  |  |  | | --- | --- | |  |  | |
| 01. General Study Information  **|  | | --- | | All questions marked with a red asterisk (\*) require a response. Questions without a red asterisk may or may not require a response, depending on those questions' applicability to this study. |**   |  |  |  |  |  |  |  |  |  |  | | --- | --- | --- | --- | --- | --- | --- | --- | --- | --- | | |  |  |  |  |  | | --- | --- | --- | --- | --- | | |  | | --- | | 1.1\*  Study Title: | |  | | DATES | |  | | |  |  | | --- | --- | | |  | | --- | |  | | |  | | | |  |  |  |  |  | | --- | --- | --- | --- | --- | | |  | | --- | | 1.1.1  Full Study Title: | |  | | Decision Aid to Technologically Enhance Shared Decision Making (DATES) | |  | | |  |  | | --- | --- | | |  | | --- | |  | | |  | | | |  |  |  |  | | --- | --- | --- | --- | | |  | | --- | | **1.1.2  If there are other U-M studies related to this project, enter the eResearch ID number (HUM#) or IRBMED Legacy study number. Examples of related projects include, but are not limited to:**   - Projects funded under the same grant- IRBMED Legacy study being migrated into eResearch- Previously approved Umbrella applications (such as Center Grants or approvals for release of funding)- Previously approved projects for which this is a follow up study | |  | |  | | |  |  | | --- | --- | | |  | | --- | |  | | |  | | | |  |  |  |  |  | | --- | --- | --- | --- | --- | | |  | | --- | | 1.2\*  Principal Investigator: | |  | | Masahito Jimbo    ***Note:*** If the user is not in the system, you may  Create A New User Account... | |  | | |  |  | | --- | --- | | |  | | --- | |  | | |  | | | |  |  |  |  |  |  |  |  |  |  |  |  |  |  |  |  |  |  |  |  |  |  |  |  |  |  |  |  |  |  |  |  |  |  |  |  |  |  |  |  |  |  |  |  |  |  |  |  |  |  |  |  |  |  |  |  |  |  |  |  |  |  |  |  |  |  |  |  |  |  |  |  |  |  |  |  |  |  |  |  |  |  |  |  |  |  |  |  |  |  |  |  |  |  |  |  |  |  |  |  |  |  |  |  |  |  |  |  |  |  |  |  |  |  |  |  |  |  |  |  |  |  |  |  |  |  |  |  |  |  |  |  |  |  |  |  |  |  |  |  |  |  |  |  |  |  |  |  |  |  |  |  |  |  |  |  |  |  |  |  |  |  |  |  |  |  |  |  |  |  |  |  |  |  |  |  |  |  |  |  |  |  |  |  |  |  | | --- | --- | --- | --- | --- | --- | --- | --- | --- | --- | --- | --- | --- | --- | --- | --- | --- | --- | --- | --- | --- | --- | --- | --- | --- | --- | --- | --- | --- | --- | --- | --- | --- | --- | --- | --- | --- | --- | --- | --- | --- | --- | --- | --- | --- | --- | --- | --- | --- | --- | --- | --- | --- | --- | --- | --- | --- | --- | --- | --- | --- | --- | --- | --- | --- | --- | --- | --- | --- | --- | --- | --- | --- | --- | --- | --- | --- | --- | --- | --- | --- | --- | --- | --- | --- | --- | --- | --- | --- | --- | --- | --- | --- | --- | --- | --- | --- | --- | --- | --- | --- | --- | --- | --- | --- | --- | --- | --- | --- | --- | --- | --- | --- | --- | --- | --- | --- | --- | --- | --- | --- | --- | --- | --- | --- | --- | --- | --- | --- | --- | --- | --- | --- | --- | --- | --- | --- | --- | --- | --- | --- | --- | --- | --- | --- | --- | --- | --- | --- | --- | --- | --- | --- | --- | --- | --- | --- | --- | --- | --- | --- | --- | --- | --- | --- | --- | --- | --- | --- | --- | --- | --- | --- | --- | --- | --- | --- | --- | --- | --- | --- | --- | --- | --- | --- | --- | | |  | | --- | | 1.3  Study Team Members: | |  | | |  |  |  |  |  |  |  |  |  |  |  |  |  |  |  |  |  |  |  |  |  |  |  |  |  |  |  |  |  |  |  |  |  |  |  |  |  |  |  |  |  |  |  |  |  |  |  |  |  |  |  |  |  |  |  |  |  |  |  |  |  |  |  |  |  |  |  |  |  |  |  |  |  |  |  |  |  |  |  |  |  |  |  |  |  |  |  |  |  |  |  |  |  |  |  |  |  |  |  |  |  |  |  |  |  |  |  |  |  |  |  |  |  |  |  |  |  |  |  |  |  |  |  |  |  |  |  |  |  |  |  |  |  |  |  |  |  |  |  |  |  |  |  |  |  |  |  |  |  |  |  |  |  |  |  |  |  |  |  |  |  |  |  |  |  |  |  |  |  |  |  |  |  |  |  |  |  |  |  |  |  | | --- | --- | --- | --- | --- | --- | --- | --- | --- | --- | --- | --- | --- | --- | --- | --- | --- | --- | --- | --- | --- | --- | --- | --- | --- | --- | --- | --- | --- | --- | --- | --- | --- | --- | --- | --- | --- | --- | --- | --- | --- | --- | --- | --- | --- | --- | --- | --- | --- | --- | --- | --- | --- | --- | --- | --- | --- | --- | --- | --- | --- | --- | --- | --- | --- | --- | --- | --- | --- | --- | --- | --- | --- | --- | --- | --- | --- | --- | --- | --- | --- | --- | --- | --- | --- | --- | --- | --- | --- | --- | --- | --- | --- | --- | --- | --- | --- | --- | --- | --- | --- | --- | --- | --- | --- | --- | --- | --- | --- | --- | --- | --- | --- | --- | --- | --- | --- | --- | --- | --- | --- | --- | --- | --- | --- | --- | --- | --- | --- | --- | --- | --- | --- | --- | --- | --- | --- | --- | --- | --- | --- | --- | --- | --- | --- | --- | --- | --- | --- | --- | --- | --- | --- | --- | --- | --- | --- | --- | --- | --- | --- | --- | --- | --- | --- | --- | --- | --- | --- | --- | --- | --- | --- | --- | --- | --- | --- | --- | --- | --- | --- | | | Study Team Member | Study Team Role | Appointment Dept | Appointment Selection Complete? | Student | Friend Account | Conflict of Interest | Edit Rights | Accepted Role? | PEERRS Human Subjects? | | --- | --- | --- | --- | --- | --- | --- | --- | --- | --- | | Masahito Jimbo | PI | Family Medicine | Yes | no | No | no | yes | N/A | yes | | Lawrence An | Co-Investigator | Int Med-General Medicine | Yes | no | No | no | no | Yes | yes | | Jennifer Elston Lafata | Co-Investigator |  | N/A | no | Yes | no | no | Yes | yes | | Sarah Hawley | Co-Investigator | Int Med-General Medicine | Yes | no | No | no | no | Yes | yes | | Karen Kelly-Blake | Co-Investigator |  | N/A | no | Yes | no | yes | Yes | yes | | Mack Ruffin | Co-Investigator | Family Medicine | Yes | no | No | no | no | Yes | yes | | Ananda Sen | Co-Investigator | Family Medicine | Yes | no | No | no | no | Yes | yes | | Kathleen Lawrence | Study Coordinator/Project Manager | Family Medicine | Yes | no | No | no | yes | Yes | yes | | Mary Rapai | Study Coordinator/Project Manager | Family Medicine | Yes | no | No | no | yes | Yes | yes | | Ronald Trendler | Study Coordinator/Project Manager | Family Medicine | Yes | no | No | no | yes | Yes | yes | | Donald Nease Jr | Consultant |  | N/A | no | No | no | no | Yes | yes | | Tonia Gooden | Research Staff | Family Medicine | Yes | no | No | no | yes | Yes | yes | | Yuhong Zhang | Research Staff | Family Medicine | Yes | no | No | no | yes | Yes | yes | | Judy Connelly | Administrative Staff | Family Medicine | Yes | no | No |  | yes | N/A | yes | | Katie Grode | Administrative Staff | Family Medicine | Yes | no | No |  | yes | N/A | yes | | Lauren Schleicher | Administrative Staff | Family Medicine | Yes | no | No |  | yes | N/A | yes | | Krystle Woods | Administrative Staff | Family Medicine | Yes | no | No |  | yes | N/A | yes | | | |  | | |  |  | | --- | --- | | |  | | --- | |  | | |  | | | |  |  |  |  |  | | --- | --- | --- | --- | --- | | |  | | --- | | 1.8\*  Project Summary: | |  | | Colorectal cancer screening (CRCS) is usually discussed between the patient and the primary care (PC) physician in the context of a clinic visit. However, PC physicians face a challenge in promoting CRCS in the face of multiple competing demands. Additionally, PC physicians’ tendency to recommend colonoscopy over other equally viable CRCS test options could negatively impact shared decision making (SDM) and lead to lower CRCS adherence when patients’ preferences do not match colonoscopy. An intervention that provides decision support and incorporates patient preferences in CRCS test options can aid SDM and may be effective at increasing CRCS rates across diverse populations, as recommended by a recent National Institutes of Health (NIH) State-of-the-Science Conference statement.  We have developed Colorectal Web (CW), an interactive decision aid (DA) for CRCS designed to be used prior to a clinic visit to clarify patients’ preferences and promote SDM. CW provides a unique interactive Preference Clarification Tool, which helps patients determine the CRCS test option that best matches their preferences. CW was tested in a pilot randomized controlled trial (RCT), which showed that patients using CW were more likely to undergo CRCS than those who used a standard, non-interactive website. This increase in CRCS was similar in Caucasians and African Americans; thus, CW could have higher impact in the latter group, which suffers from higher colorectal cancer (CRC) burden. To our knowledge, our pilot RCT was the first published study to show that a preference-tailored DA improved CRCS adherence. However, patients who used CW did not always complete CRCS through their preferred screening test option. We surmised that the patient-physician communication during the subsequent clinic visit affected the final choice, but the pilot RCT did not directly address this issue. Our currently proposed project will test CW's effectiveness for increasing CRCS and its facilitation of SDM in PC practices. The new CW incorporates interactive patient preference clarification and risk assessment that provide real-time information to the patient and the physician during the clinic visit, making CW more applicable to real-world PC practices. We will perform the study in two phases. Phase 1 will be usability testing of 10 participants. Phase 2 will be a 2-armed RCT (300 patients per arm) comparing the Intervention Arm using CW to the Control Arm using a non-interactive control website, Standard Web (SW), in 10 PC practices, recruiting 20 clinicians, in Metro Detroit with a large African American population.  A total of 630 participants will be recruited-- 10 participants will be recruited for usability testing, 20 physicians will be recruited to participant and 600 (300 per arm) patients will be recruited to participate. | |  | | |  |  | | --- | --- | | |  | | --- | |  | | | Additional Help | | | |  |  |  |  |  | | --- | --- | --- | --- | --- | | |  | | --- | | 1.9\*  Select the appropriate IRB: | |  | | IRBMED | |  | | |  |  | | --- | --- | | |  | | --- | |  | | |  | | | |  |  |  |  |  | | --- | --- | --- | --- | --- | | |  | | --- | | 1.10\*  *Estimated* Study Start Date (Not required for IRBMED): (mm/dd/yyyy) | |  | | 1/1/2012 | |  | | |  |  | | --- | --- | | |  | | --- | |  | | |  | | | |  |  |  |  |  | | --- | --- | --- | --- | --- | | |  | | --- | | 1.11\*  *Estimated* Duration of Study: | |  | | 4 years | |  | | |  |  | | --- | --- | | |  | | --- | |  | | |  | |  Approved |
| 01-1. Application Type   |  |  |  |  |  |  |  |  |  |  | | --- | --- | --- | --- | --- | --- | --- | --- | --- | --- | | |  |  |  |  |  | | --- | --- | --- | --- | --- | | |  | | --- | | 1-1.1\* Select the appropriate application type. | |  | | **Standard**, non-exempt, research project | |  | | |  |  | | --- | --- | | |  | | --- | |  | | |  | | |
| 01-2. Standard Study Information     |  |  |  |  |  |  |  |  |  |  | | --- | --- | --- | --- | --- | --- | --- | --- | --- | --- | | |  |  |  |  |  | | --- | --- | --- | --- | --- | | |  | | --- | | 1-2.1\*  Who initiated this study? | |  | | Investigator   **If other, please specify:** | |  | | |  |  | | --- | --- | | |  | | --- | |  | | |  | | | |  |  |  |  |  | | --- | --- | --- | --- | --- | | |  | | --- | | 1-2.2\*  Are you or any students working on this project being paid from a federally funded training grant? | |  | | Yes **No** | |  | | |  |  | | --- | --- | | |  | | --- | |  | | |  | | | |  |  |  |  |  | | --- | --- | --- | --- | --- | | |  | | --- | | 1-2.3  This study is currently associated with the following department.  To associate this research with a different department, click Select. If the department has defaulted to "student", click select to specify the department through which this application is being submitted. | |  | | Family Medicine | |  | | |  |  | | --- | --- | | |  | | --- | |  | | |  | | | |  |  |  |  |  |  |  |  |  |  |  |  |  |  |  |  |  | | --- | --- | --- | --- | --- | --- | --- | --- | --- | --- | --- | --- | --- | --- | --- | --- | --- | | |  | | --- | | 1-2.4  Will the study utilize resources from the following centers? | |  | | |  |  |  |  |  |  |  |  |  |  |  |  | | --- | --- | --- | --- | --- | --- | --- | --- | --- | --- | --- | --- | | | Select all that apply: | | --- | |  |  |  |  |  |  |  |  |  |  | | --- | --- | --- | --- | --- | --- | --- | --- | --- | --- | | There are no items to display | | | | | | | | | | | | |  | | |  |  | | --- | --- | | |  | | --- | |  | | |  | |         |  |  |  |  |  |  |  |  |  |  | | --- | --- | --- | --- | --- | --- | --- | --- | --- | --- | | |  |  |  |  |  | | --- | --- | --- | --- | --- | | |  | | --- | | 1-2.5\*  Does this study require review by the UM Health System Comprehensive Cancer Center Protocol Review Committee (PRC)? | |  | | Yes **No** | |  | | |  |  | | --- | --- | | |  | | --- | |  | | |  | |      |  |  |  |  |  |  |  |  |  |  |  |  |  |  |  |  |  |  |  |  |  |  | | --- | --- | --- | --- | --- | --- | --- | --- | --- | --- | --- | --- | --- | --- | --- | --- | --- | --- | --- | --- | --- | --- | | |  |  |  |  |  |  |  |  |  |  |  |  |  |  |  |  |  | | --- | --- | --- | --- | --- | --- | --- | --- | --- | --- | --- | --- | --- | --- | --- | --- | --- | | |  | | --- | | 1-2.5.1  Indicate needed Clinical Trials Office (CTO) resources. | |  | | |  |  |  |  |  |  |  |  |  |  |  |  | | --- | --- | --- | --- | --- | --- | --- | --- | --- | --- | --- | --- | | | Select all that apply: | | --- | |  |  |  |  |  |  |  |  |  |  | | --- | --- | --- | --- | --- | --- | --- | --- | --- | --- | | There are no items to display | | | | | | | | | | | | |  | | |  |  | | --- | --- | | |  | | --- | |  | | |  | |            |  |  |  |  |  |  |  |  |  |  | | --- | --- | --- | --- | --- | --- | --- | --- | --- | --- | | |  |  |  |  |  | | --- | --- | --- | --- | --- | | |  | | --- | | 1-2.6\*  Has the scientific merit of this study already been peer reviewed (i.e., reviewed by one or more recognized authorities on the subject)? | |  | | **Yes** No | |  | | |  |  | | --- | --- | | |  | | --- | |  | | |  | |      |  |  |  |  |  |  |  |  |  |  |  |  |  | | --- | --- | --- | --- | --- | --- | --- | --- | --- | --- | --- | --- | --- | | |  |  |  |  |  |  |  |  | | --- | --- | --- | --- | --- | --- | --- | --- | | |  |  |  |  | | --- | --- | --- | --- | | 1-2.6.1\*  List the peer-review organization(s).   |  |  |  | | --- | --- | --- | | | Peer Review Organization | | --- | | External sponsor review process (e.g. study selection) | | | |  | |  | |  | | |  |  | | --- | --- | | |  | | --- | |  | | |  | |     |  |  |  |  |  |  |  |  |  |  | | --- | --- | --- | --- | --- | --- | --- | --- | --- | --- | | |  |  |  |  |  | | --- | --- | --- | --- | --- | | |  | | --- | | 1-2.7\*  Is this a clinical trial? | |  | | Yes **No** | |  | | |  |  | | --- | --- | | |  | | --- | |  | | |  | |      |  |  |  |  |  |  |  |  |  |  |  |  |  |  |  |  |  |  |  |  |  |  |  |  |  |  |  |  |  |  |  |  |  |  |  |  |  |  |  |  |  |  |  |  |  |  |  |  | | --- | --- | --- | --- | --- | --- | --- | --- | --- | --- | --- | --- | --- | --- | --- | --- | --- | --- | --- | --- | --- | --- | --- | --- | --- | --- | --- | --- | --- | --- | --- | --- | --- | --- | --- | --- | --- | --- | --- | --- | --- | --- | --- | --- | --- | --- | --- | --- | | |  |  |  |  |  |  |  |  |  |  |  |  |  |  |  |  |  |  |  |  |  |  |  |  |  |  |  |  |  |  |  |  |  |  |  |  |  |  |  |  |  |  |  | | --- | --- | --- | --- | --- | --- | --- | --- | --- | --- | --- | --- | --- | --- | --- | --- | --- | --- | --- | --- | --- | --- | --- | --- | --- | --- | --- | --- | --- | --- | --- | --- | --- | --- | --- | --- | --- | --- | --- | --- | --- | --- | --- | | |  | | --- | | 1-2.7.1\*  Please select the trial phase | |  | | |  | Trial Phase | Description | | --- | --- | --- | |  | Phase 0 | Exploratory study to determine whether agent behaves in humans as pre-clinical testing indicated | |  | Phase I | Evaluate safety, dose range, and identify side effects in healthy volunteers or in patients with the disease of interest | |  | Phase I/II | Safety, dosage levels and efficacy of new treatment; device pilot study | |  | Phase II | Evaluate safety and efficacy of selected dosage levels and duration of treatment | |  | Phase II/III | Larger study to confirm safety and efficacy of selected dose(s) and duration of treatment | |  | Phase III | Larger, controlled trial to confirm efficacy compared to a standard treatment regimen or to no treatment; device pivotal study | |  | Phase IV | Post-market study to provide additional information on risks, benefits, and optimal use | |  | Other |  | |  |  |  |  |  |  |  |  |  |  |  | | --- | --- | --- | --- | --- | --- | --- | --- | --- | --- | --- | |  |  | | | | | | | | | |    **If other, please specify:** | |  | | |  |  | | --- | --- | | |  | | --- | |  | | |  | |       |  |  |  |  |  |  |  |  |  |  | | --- | --- | --- | --- | --- | --- | --- | --- | --- | --- | | |  |  |  |  |  | | --- | --- | --- | --- | --- | | |  | | --- | | 1-2.8\*  Would the integrity of this research study be compromised if the subject were able to view results of their research tests or medications in the Patient Portal of MyUofMHealth.org? Research results displayed to the subject in MyUofMHealth.org will include: lab results, radiology examinations and outpatient medication lists. Contracts and protocols should be assessed by the Principal Investigator for specific language regarding blinding of subjects and their research results.  *(NOTE: Additional actions are required in order to limit the subject’s view into their electronic medical record. Contact the IRB for additional information or see additional guidance for blinded studies at http://med.umich.edu/irbmed/guidance/blind.htm)* | |  | | Yes **No** | |  | | |  |  | | --- | --- | | |  | | --- | |  | | |  | | |
| Study Team Detail     |  |  |  |  |  |  |  |  |  |  |  |  |  |  |  |  |  |  |  |  | | --- | --- | --- | --- | --- | --- | --- | --- | --- | --- | --- | --- | --- | --- | --- | --- | --- | --- | --- | --- | | |  |  |  |  |  |  |  |  |  |  |  |  |  |  |  | | --- | --- | --- | --- | --- | --- | --- | --- | --- | --- | --- | --- | --- | --- | --- | | |  | | --- | | 1.4  Team Member: | |  | | Masahito Jimbo     |  |  | | --- | --- | |  |  | | Preferred email: | mjimbo@umich.edu | | Business phone | 734-998-7120 | | Business address: | Family Medicine | 1018 Fuller St SPC 5708 | 48109-5708 | | |  | | |  |  | | --- | --- | | |  | | --- | |  | | |  | | | |  |  |  |  |  | | --- | --- | --- | --- | --- | | |  | | --- | | 1.5  Function with respect to project: | |  | | PI | |  | | |  |  | | --- | --- | | |  | | --- | |  | | |  | | | |  |  |  |  |  | | --- | --- | --- | --- | --- | | |  | | --- | | 1.6  Allow this person to EDIT the application, including any supporting documents/stipulations requested during the review process: | |  | | yes | |  | | |  |  | | --- | --- | | |  | | --- | |  | | |  | | | |  |  |  |  |  | | --- | --- | --- | --- | --- | | |  | | --- | | 1.7  Include this person on all correspondences regarding this application: (Note: This will include all committee correspondence, decision outcomes, renewal notices, and adverse event submissions.) | |  | |  | |  | | |  |  | | --- | --- | | |  | | --- | |  | | |  | | | |  |  |  |  |  | | --- | --- | --- | --- | --- | | |  | | --- | | **Credentials: Required for PI, Co-Is and Faculty Advisors** | |  | |  | |  | | |  |  | | --- | --- | | |  | | --- | |  | | |  | | | |  |  |  |  |  |  |  |  |  |  | | --- | --- | --- | --- | --- | --- | --- | --- | --- | --- | | |  | | --- | | Upload or update your CV, resume, or biographical sketch. | |  | | |  |  |  |  |  | | --- | --- | --- | --- | --- | | | Name | Version | | --- | --- | | Jimbo CV 06-17-2013.doc | History | 0.07 | | | |  | | |  |  | | --- | --- | | |  | | --- | |  | | |  | | | |  |  |  |  |  | | --- | --- | --- | --- | --- | | |  | | --- | | **Conflict of Interest Detail:  Required for all roles except Administrative Staff** | |  | |  | |  | | |  |  | | --- | --- | | |  | | --- | |  | | |  | | | |  |  |  |  |  |  | | --- | --- | --- | --- | --- | --- | | |  | | --- | | C1  Do you, your spouse, domestic partner, or dependents have any outside interests or relationships to companies or entities related to this research that the IRB should consider? | | **Examples of outside interests include, but are not limited to the following:** | | - receiving compensation whose value could be affected by the study outcome- IN THE AGGREGATE, expecting to receive compensation from the sponsor of the research of $10,000 or greater in the next year- having a proprietary interest in the sponsor of the research or a product tested by this research including but not limited to, a patent, trademark, copyright, or licensing agreement, or the right to receive royalties from product commercialization- individually or collectively, having an ownership interest (equity or stock options) in the sponsor of the research or product being tested whose value cannot be readily determined through reference to public prices- individually or collectively, having an ownership interest (equity or stock options) in a company or product whose value could be affected by the study outcome- IN THE AGGREGATE, having an ownership interest (equity or stock options) in the sponsor of the research that exceeds $10,000 or 1% when the sponsor is a publicly traded entity- receiving significant payments of other sorts with an aggregate value of $10,000 or more (or payment of ANY amount to medical school or hospital employees) made directly by the sponsor of this research for unrestricted research or education, equipment, consultancy, or honorarium- holding a position of management or leadership in company or entity related to this research including, but not limited to, officer, director, or member of an advisory board.- providing consulting services or serve on a Speaker’s Bureau, either paid or unpaid, to the financial or non-financial sponsor of this study- when the sponsor is a publicly traded entity, having any ownership interest (equity or stock options) in the sponsor- expecting to receive any loans, educational support, contributions of in-kind for equipment, or any other non-compensatory payment from the sponsor of the research in the next year | | no | |  | | |  |  | | --- | --- | | |  | | --- | |  | | |  | | | |  |  |  |  |  | | --- | --- | --- | --- | --- | | |  | | --- | | C2  Please provide a detailed description of the outside interest in the box below. | |  | |  | |  | | |  |  | | --- | --- | | |  | | --- | |  | | |  | | | |  |  |  |  |  | | --- | --- | --- | --- | --- | | |  | | --- | | C2.1  Where have you submitted a disclosure of this outside interest? | |  | |  | |  | | |  |  | | --- | --- | | |  | | --- | |  | | |  | | | |  |  |  |  |  | | --- | --- | --- | --- | --- | | |  | | --- | | C2.2  Has a management plan been formalized? | |  | |  | |  | | |  |  | | --- | --- | | |  | | --- | |  | | |  | | | |  |  |  |  |  |  |  |  |  |  |  |  |  |  |  |  |  |  | | --- | --- | --- | --- | --- | --- | --- | --- | --- | --- | --- | --- | --- | --- | --- | --- | --- | --- | | |  | | --- | | C2.2.1  If yes, attach the management plan here, as well as any other applicable conflict of interest documents. | |  | | |  |  |  |  |  |  |  |  |  |  |  |  |  | | --- | --- | --- | --- | --- | --- | --- | --- | --- | --- | --- | --- | --- | | | Name | Version | | --- | --- | |  |  |  |  |  |  |  |  |  |  | | --- | --- | --- | --- | --- | --- | --- | --- | --- | --- | | There are no items to display | | | | | | | | | | | | |  | | |  |  | | --- | --- | | |  | | --- | |  | | |  | | | |  |  |  |  |  | | --- | --- | --- | --- | --- | | |  | | --- | | C2.2.2  If no, describe the financial interest in sufficient detail to permit the COI Ancillary Committee and the IRB to determine if such involvement represents a potential conflict-of-interest and/or should be disclosed to potential research subjects in the informed consent form. | |  | |  | |  | | |  |  | | --- | --- | | |  | | --- | |  | | |  | | |
| Study Team Detail     |  |  |  |  |  |  |  |  |  |  |  |  |  |  |  |  |  |  |  |  | | --- | --- | --- | --- | --- | --- | --- | --- | --- | --- | --- | --- | --- | --- | --- | --- | --- | --- | --- | --- | | |  |  |  |  |  |  |  |  |  |  |  |  |  |  |  | | --- | --- | --- | --- | --- | --- | --- | --- | --- | --- | --- | --- | --- | --- | --- | | |  | | --- | | 1.4  Team Member: | |  | | Lawrence An     |  |  | | --- | --- | |  |  | | Preferred email: | lcan@umich.edu | | Business phone | 734-763-6099 | | Business address: | UM CHCR | 2800 Plymouth Rd NCRC B16-G033N | 48109-2800 | | |  | | |  |  | | --- | --- | | |  | | --- | |  | | |  | | | |  |  |  |  |  | | --- | --- | --- | --- | --- | | |  | | --- | | 1.5  Function with respect to project: | |  | | Co-Investigator | |  | | |  |  | | --- | --- | | |  | | --- | |  | | |  | | | |  |  |  |  |  | | --- | --- | --- | --- | --- | | |  | | --- | | 1.6  Allow this person to EDIT the application, including any supporting documents/stipulations requested during the review process: | |  | | no | |  | | |  |  | | --- | --- | | |  | | --- | |  | | |  | | | |  |  |  |  |  | | --- | --- | --- | --- | --- | | |  | | --- | | 1.7  Include this person on all correspondences regarding this application: (Note: This will include all committee correspondence, decision outcomes, renewal notices, and adverse event submissions.) | |  | | yes | |  | | |  |  | | --- | --- | | |  | | --- | |  | | |  | | | |  |  |  |  |  | | --- | --- | --- | --- | --- | | |  | | --- | | **Credentials: Required for PI, Co-Is and Faculty Advisors** | |  | |  | |  | | |  |  | | --- | --- | | |  | | --- | |  | | |  | | | |  |  |  |  |  |  |  |  |  |  |  |  | | --- | --- | --- | --- | --- | --- | --- | --- | --- | --- | --- | --- | | |  | | --- | | Upload or update your CV, resume, or biographical sketch. | |  | | |  |  |  |  |  |  |  | | --- | --- | --- | --- | --- | --- | --- | | | Name | Version | | --- | --- | | AnBiosketch | History | 0.01 | | Biosketch\_An | History | 0.01 | | | |  | | |  |  | | --- | --- | | |  | | --- | |  | | |  | | | |  |  |  |  |  | | --- | --- | --- | --- | --- | | |  | | --- | | **Conflict of Interest Detail:  Required for all roles except Administrative Staff** | |  | |  | |  | | |  |  | | --- | --- | | |  | | --- | |  | | |  | | | |  |  |  |  |  |  | | --- | --- | --- | --- | --- | --- | | |  | | --- | | C1  Do you, your spouse, domestic partner, or dependents have any outside interests or relationships to companies or entities related to this research that the IRB should consider? | | **Examples of outside interests include, but are not limited to the following:** | | - receiving compensation whose value could be affected by the study outcome- IN THE AGGREGATE, expecting to receive compensation from the sponsor of the research of $10,000 or greater in the next year- having a proprietary interest in the sponsor of the research or a product tested by this research including but not limited to, a patent, trademark, copyright, or licensing agreement, or the right to receive royalties from product commercialization- individually or collectively, having an ownership interest (equity or stock options) in the sponsor of the research or product being tested whose value cannot be readily determined through reference to public prices- individually or collectively, having an ownership interest (equity or stock options) in a company or product whose value could be affected by the study outcome- IN THE AGGREGATE, having an ownership interest (equity or stock options) in the sponsor of the research that exceeds $10,000 or 1% when the sponsor is a publicly traded entity- receiving significant payments of other sorts with an aggregate value of $10,000 or more (or payment of ANY amount to medical school or hospital employees) made directly by the sponsor of this research for unrestricted research or education, equipment, consultancy, or honorarium- holding a position of management or leadership in company or entity related to this research including, but not limited to, officer, director, or member of an advisory board.- providing consulting services or serve on a Speaker’s Bureau, either paid or unpaid, to the financial or non-financial sponsor of this study- when the sponsor is a publicly traded entity, having any ownership interest (equity or stock options) in the sponsor- expecting to receive any loans, educational support, contributions of in-kind for equipment, or any other non-compensatory payment from the sponsor of the research in the next year | | no | |  | | |  |  | | --- | --- | | |  | | --- | |  | | |  | | | |  |  |  |  |  | | --- | --- | --- | --- | --- | | |  | | --- | | C2  Please provide a detailed description of the outside interest in the box below. | |  | |  | |  | | |  |  | | --- | --- | | |  | | --- | |  | | |  | | | |  |  |  |  |  | | --- | --- | --- | --- | --- | | |  | | --- | | C2.1  Where have you submitted a disclosure of this outside interest? | |  | |  | |  | | |  |  | | --- | --- | | |  | | --- | |  | | |  | | | |  |  |  |  |  | | --- | --- | --- | --- | --- | | |  | | --- | | C2.2  Has a management plan been formalized? | |  | |  | |  | | |  |  | | --- | --- | | |  | | --- | |  | | |  | | | |  |  |  |  |  |  |  |  |  |  |  |  |  |  |  |  |  |  | | --- | --- | --- | --- | --- | --- | --- | --- | --- | --- | --- | --- | --- | --- | --- | --- | --- | --- | | |  | | --- | | C2.2.1  If yes, attach the management plan here, as well as any other applicable conflict of interest documents. | |  | | |  |  |  |  |  |  |  |  |  |  |  |  |  | | --- | --- | --- | --- | --- | --- | --- | --- | --- | --- | --- | --- | --- | | | Name | Version | | --- | --- | |  |  |  |  |  |  |  |  |  |  | | --- | --- | --- | --- | --- | --- | --- | --- | --- | --- | | There are no items to display | | | | | | | | | | | | |  | | |  |  | | --- | --- | | |  | | --- | |  | | |  | | | |  |  |  |  |  | | --- | --- | --- | --- | --- | | |  | | --- | | C2.2.2  If no, describe the financial interest in sufficient detail to permit the COI Ancillary Committee and the IRB to determine if such involvement represents a potential conflict-of-interest and/or should be disclosed to potential research subjects in the informed consent form. | |  | |  | |  | | |  |  | | --- | --- | | |  | | --- | |  | | |  | | |
| Study Team Detail     |  |  |  |  |  |  |  |  |  |  |  |  |  |  |  |  |  |  |  |  | | --- | --- | --- | --- | --- | --- | --- | --- | --- | --- | --- | --- | --- | --- | --- | --- | --- | --- | --- | --- | | |  |  |  |  |  |  |  |  |  |  |  |  |  |  |  | | --- | --- | --- | --- | --- | --- | --- | --- | --- | --- | --- | --- | --- | --- | --- | | |  | | --- | | 1.4  Team Member: | |  | | Jennifer Elston Lafata     |  |  | | --- | --- | |  |  | | Preferred email: | jelstonlafat@vcu.edu | | Business phone | 804-628-3293 | | Business address: | Virginia Commonwealth University | 830 E. Main Street, 9th Floor | 23219 | | |  | | |  |  | | --- | --- | | |  | | --- | |  | | |  | | | |  |  |  |  |  | | --- | --- | --- | --- | --- | | |  | | --- | | 1.5  Function with respect to project: | |  | | Co-Investigator | |  | | |  |  | | --- | --- | | |  | | --- | |  | | |  | | | |  |  |  |  |  | | --- | --- | --- | --- | --- | | |  | | --- | | 1.6  Allow this person to EDIT the application, including any supporting documents/stipulations requested during the review process: | |  | | no | |  | | |  |  | | --- | --- | | |  | | --- | |  | | |  | | | |  |  |  |  |  | | --- | --- | --- | --- | --- | | |  | | --- | | 1.7  Include this person on all correspondences regarding this application: (Note: This will include all committee correspondence, decision outcomes, renewal notices, and adverse event submissions.) | |  | | yes | |  | | |  |  | | --- | --- | | |  | | --- | |  | | |  | | | |  |  |  |  |  | | --- | --- | --- | --- | --- | | |  | | --- | | **Credentials: Required for PI, Co-Is and Faculty Advisors** | |  | |  | |  | | |  |  | | --- | --- | | |  | | --- | |  | | |  | | | |  |  |  |  |  |  |  |  |  |  | | --- | --- | --- | --- | --- | --- | --- | --- | --- | --- | | |  | | --- | | Upload or update your CV, resume, or biographical sketch. | |  | | |  |  |  |  |  | | --- | --- | --- | --- | --- | | | Name | Version | | --- | --- | | JEL NIH biosketch crc screening observational.doc | History | 0.01 | | | |  | | |  |  | | --- | --- | | |  | | --- | |  | | |  | | | |  |  |  |  |  | | --- | --- | --- | --- | --- | | |  | | --- | | **Conflict of Interest Detail:  Required for all roles except Administrative Staff** | |  | |  | |  | | |  |  | | --- | --- | | |  | | --- | |  | | |  | | | |  |  |  |  |  |  | | --- | --- | --- | --- | --- | --- | | |  | | --- | | C1  Do you, your spouse, domestic partner, or dependents have any outside interests or relationships to companies or entities related to this research that the IRB should consider? | | **Examples of outside interests include, but are not limited to the following:** | | - receiving compensation whose value could be affected by the study outcome- IN THE AGGREGATE, expecting to receive compensation from the sponsor of the research of $10,000 or greater in the next year- having a proprietary interest in the sponsor of the research or a product tested by this research including but not limited to, a patent, trademark, copyright, or licensing agreement, or the right to receive royalties from product commercialization- individually or collectively, having an ownership interest (equity or stock options) in the sponsor of the research or product being tested whose value cannot be readily determined through reference to public prices- individually or collectively, having an ownership interest (equity or stock options) in a company or product whose value could be affected by the study outcome- IN THE AGGREGATE, having an ownership interest (equity or stock options) in the sponsor of the research that exceeds $10,000 or 1% when the sponsor is a publicly traded entity- receiving significant payments of other sorts with an aggregate value of $10,000 or more (or payment of ANY amount to medical school or hospital employees) made directly by the sponsor of this research for unrestricted research or education, equipment, consultancy, or honorarium- holding a position of management or leadership in company or entity related to this research including, but not limited to, officer, director, or member of an advisory board.- providing consulting services or serve on a Speaker’s Bureau, either paid or unpaid, to the financial or non-financial sponsor of this study- when the sponsor is a publicly traded entity, having any ownership interest (equity or stock options) in the sponsor- expecting to receive any loans, educational support, contributions of in-kind for equipment, or any other non-compensatory payment from the sponsor of the research in the next year | | no | |  | | |  |  | | --- | --- | | |  | | --- | |  | | |  | | | |  |  |  |  |  | | --- | --- | --- | --- | --- | | |  | | --- | | C2  Please provide a detailed description of the outside interest in the box below. | |  | |  | |  | | |  |  | | --- | --- | | |  | | --- | |  | | |  | | | |  |  |  |  |  | | --- | --- | --- | --- | --- | | |  | | --- | | C2.1  Where have you submitted a disclosure of this outside interest? | |  | |  | |  | | |  |  | | --- | --- | | |  | | --- | |  | | |  | | | |  |  |  |  |  | | --- | --- | --- | --- | --- | | |  | | --- | | C2.2  Has a management plan been formalized? | |  | |  | |  | | |  |  | | --- | --- | | |  | | --- | |  | | |  | | | |  |  |  |  |  |  |  |  |  |  |  |  |  |  |  |  |  |  | | --- | --- | --- | --- | --- | --- | --- | --- | --- | --- | --- | --- | --- | --- | --- | --- | --- | --- | | |  | | --- | | C2.2.1  If yes, attach the management plan here, as well as any other applicable conflict of interest documents. | |  | | |  |  |  |  |  |  |  |  |  |  |  |  |  | | --- | --- | --- | --- | --- | --- | --- | --- | --- | --- | --- | --- | --- | | | Name | Version | | --- | --- | |  |  |  |  |  |  |  |  |  |  | | --- | --- | --- | --- | --- | --- | --- | --- | --- | --- | | There are no items to display | | | | | | | | | | | | |  | | |  |  | | --- | --- | | |  | | --- | |  | | |  | | | |  |  |  |  |  | | --- | --- | --- | --- | --- | | |  | | --- | | C2.2.2  If no, describe the financial interest in sufficient detail to permit the COI Ancillary Committee and the IRB to determine if such involvement represents a potential conflict-of-interest and/or should be disclosed to potential research subjects in the informed consent form. | |  | |  | |  | | |  |  | | --- | --- | | |  | | --- | |  | | |  | | |
| Study Team Detail     |  |  |  |  |  |  |  |  |  |  |  |  |  |  |  |  |  |  |  |  | | --- | --- | --- | --- | --- | --- | --- | --- | --- | --- | --- | --- | --- | --- | --- | --- | --- | --- | --- | --- | | |  |  |  |  |  |  |  |  |  |  |  |  |  |  |  | | --- | --- | --- | --- | --- | --- | --- | --- | --- | --- | --- | --- | --- | --- | --- | | |  | | --- | | 1.4  Team Member: | |  | | Sarah Hawley     |  |  | | --- | --- | |  |  | | Preferred email: | sarahawl@umich.edu | | Business phone | 734-936-8816 | | Business address: | Int Med - General Medicine | NCRC 2800 Plymouth Rd Bldg 16/406E | 48109-2800 | | |  | | |  |  | | --- | --- | | |  | | --- | |  | | |  | | | |  |  |  |  |  | | --- | --- | --- | --- | --- | | |  | | --- | | 1.5  Function with respect to project: | |  | | Co-Investigator | |  | | |  |  | | --- | --- | | |  | | --- | |  | | |  | | | |  |  |  |  |  | | --- | --- | --- | --- | --- | | |  | | --- | | 1.6  Allow this person to EDIT the application, including any supporting documents/stipulations requested during the review process: | |  | | no | |  | | |  |  | | --- | --- | | |  | | --- | |  | | |  | | | |  |  |  |  |  | | --- | --- | --- | --- | --- | | |  | | --- | | 1.7  Include this person on all correspondences regarding this application: (Note: This will include all committee correspondence, decision outcomes, renewal notices, and adverse event submissions.) | |  | | no | |  | | |  |  | | --- | --- | | |  | | --- | |  | | |  | | | |  |  |  |  |  | | --- | --- | --- | --- | --- | | |  | | --- | | **Credentials: Required for PI, Co-Is and Faculty Advisors** | |  | |  | |  | | |  |  | | --- | --- | | |  | | --- | |  | | |  | | | |  |  |  |  |  |  |  |  |  |  | | --- | --- | --- | --- | --- | --- | --- | --- | --- | --- | | |  | | --- | | Upload or update your CV, resume, or biographical sketch. | |  | | |  |  |  |  |  | | --- | --- | --- | --- | --- | | | Name | Version | | --- | --- | | Hawley CV | History | 0.03 | | | |  | | |  |  | | --- | --- | | |  | | --- | |  | | |  | | | |  |  |  |  |  | | --- | --- | --- | --- | --- | | |  | | --- | | **Conflict of Interest Detail:  Required for all roles except Administrative Staff** | |  | |  | |  | | |  |  | | --- | --- | | |  | | --- | |  | | |  | | | |  |  |  |  |  |  | | --- | --- | --- | --- | --- | --- | | |  | | --- | | C1  Do you, your spouse, domestic partner, or dependents have any outside interests or relationships to companies or entities related to this research that the IRB should consider? | | **Examples of outside interests include, but are not limited to the following:** | | - receiving compensation whose value could be affected by the study outcome- IN THE AGGREGATE, expecting to receive compensation from the sponsor of the research of $10,000 or greater in the next year- having a proprietary interest in the sponsor of the research or a product tested by this research including but not limited to, a patent, trademark, copyright, or licensing agreement, or the right to receive royalties from product commercialization- individually or collectively, having an ownership interest (equity or stock options) in the sponsor of the research or product being tested whose value cannot be readily determined through reference to public prices- individually or collectively, having an ownership interest (equity or stock options) in a company or product whose value could be affected by the study outcome- IN THE AGGREGATE, having an ownership interest (equity or stock options) in the sponsor of the research that exceeds $10,000 or 1% when the sponsor is a publicly traded entity- receiving significant payments of other sorts with an aggregate value of $10,000 or more (or payment of ANY amount to medical school or hospital employees) made directly by the sponsor of this research for unrestricted research or education, equipment, consultancy, or honorarium- holding a position of management or leadership in company or entity related to this research including, but not limited to, officer, director, or member of an advisory board.- providing consulting services or serve on a Speaker’s Bureau, either paid or unpaid, to the financial or non-financial sponsor of this study- when the sponsor is a publicly traded entity, having any ownership interest (equity or stock options) in the sponsor- expecting to receive any loans, educational support, contributions of in-kind for equipment, or any other non-compensatory payment from the sponsor of the research in the next year | | no | |  | | |  |  | | --- | --- | | |  | | --- | |  | | |  | | | |  |  |  |  |  | | --- | --- | --- | --- | --- | | |  | | --- | | C2  Please provide a detailed description of the outside interest in the box below. | |  | |  | |  | | |  |  | | --- | --- | | |  | | --- | |  | | |  | | | |  |  |  |  |  | | --- | --- | --- | --- | --- | | |  | | --- | | C2.1  Where have you submitted a disclosure of this outside interest? | |  | |  | |  | | |  |  | | --- | --- | | |  | | --- | |  | | |  | | | |  |  |  |  |  | | --- | --- | --- | --- | --- | | |  | | --- | | C2.2  Has a management plan been formalized? | |  | |  | |  | | |  |  | | --- | --- | | |  | | --- | |  | | |  | | | |  |  |  |  |  |  |  |  |  |  |  |  |  |  |  |  |  |  | | --- | --- | --- | --- | --- | --- | --- | --- | --- | --- | --- | --- | --- | --- | --- | --- | --- | --- | | |  | | --- | | C2.2.1  If yes, attach the management plan here, as well as any other applicable conflict of interest documents. | |  | | |  |  |  |  |  |  |  |  |  |  |  |  |  | | --- | --- | --- | --- | --- | --- | --- | --- | --- | --- | --- | --- | --- | | | Name | Version | | --- | --- | |  |  |  |  |  |  |  |  |  |  | | --- | --- | --- | --- | --- | --- | --- | --- | --- | --- | | There are no items to display | | | | | | | | | | | | |  | | |  |  | | --- | --- | | |  | | --- | |  | | |  | | | |  |  |  |  |  | | --- | --- | --- | --- | --- | | |  | | --- | | C2.2.2  If no, describe the financial interest in sufficient detail to permit the COI Ancillary Committee and the IRB to determine if such involvement represents a potential conflict-of-interest and/or should be disclosed to potential research subjects in the informed consent form. | |  | |  | |  | | |  |  | | --- | --- | | |  | | --- | |  | | |  | | |
| Study Team Detail     |  |  |  |  |  |  |  |  |  |  |  |  |  |  |  |  |  |  |  |  | | --- | --- | --- | --- | --- | --- | --- | --- | --- | --- | --- | --- | --- | --- | --- | --- | --- | --- | --- | --- | | |  |  |  |  |  |  |  |  |  |  |  |  |  |  |  | | --- | --- | --- | --- | --- | --- | --- | --- | --- | --- | --- | --- | --- | --- | --- | | |  | | --- | | 1.4  Team Member: | |  | | Karen Kelly-Blake     |  |  | | --- | --- | |  |  | | Preferred email: | kellyka1@msu.edu | | Business phone | 517-353-8582 | | Business address: | Center for Ethics and Humanities in the Life Sciences | East Fee Hall | 48824 | | |  | | |  |  | | --- | --- | | |  | | --- | |  | | |  | | | |  |  |  |  |  | | --- | --- | --- | --- | --- | | |  | | --- | | 1.5  Function with respect to project: | |  | | Co-Investigator | |  | | |  |  | | --- | --- | | |  | | --- | |  | | |  | | | |  |  |  |  |  | | --- | --- | --- | --- | --- | | |  | | --- | | 1.6  Allow this person to EDIT the application, including any supporting documents/stipulations requested during the review process: | |  | | yes | |  | | |  |  | | --- | --- | | |  | | --- | |  | | |  | | | |  |  |  |  |  | | --- | --- | --- | --- | --- | | |  | | --- | | 1.7  Include this person on all correspondences regarding this application: (Note: This will include all committee correspondence, decision outcomes, renewal notices, and adverse event submissions.) | |  | | yes | |  | | |  |  | | --- | --- | | |  | | --- | |  | | |  | | | |  |  |  |  |  | | --- | --- | --- | --- | --- | | |  | | --- | | **Credentials: Required for PI, Co-Is and Faculty Advisors** | |  | |  | |  | | |  |  | | --- | --- | | |  | | --- | |  | | |  | | | |  |  |  |  |  |  |  |  |  |  | | --- | --- | --- | --- | --- | --- | --- | --- | --- | --- | | |  | | --- | | Upload or update your CV, resume, or biographical sketch. | |  | | |  |  |  |  |  | | --- | --- | --- | --- | --- | | | Name | Version | | --- | --- | | Kelly-Blake CV | History | 0.01 | | | |  | | |  |  | | --- | --- | | |  | | --- | |  | | |  | | | |  |  |  |  |  | | --- | --- | --- | --- | --- | | |  | | --- | | **Conflict of Interest Detail:  Required for all roles except Administrative Staff** | |  | |  | |  | | |  |  | | --- | --- | | |  | | --- | |  | | |  | | | |  |  |  |  |  |  | | --- | --- | --- | --- | --- | --- | | |  | | --- | | C1  Do you, your spouse, domestic partner, or dependents have any outside interests or relationships to companies or entities related to this research that the IRB should consider? | | **Examples of outside interests include, but are not limited to the following:** | | - receiving compensation whose value could be affected by the study outcome- IN THE AGGREGATE, expecting to receive compensation from the sponsor of the research of $10,000 or greater in the next year- having a proprietary interest in the sponsor of the research or a product tested by this research including but not limited to, a patent, trademark, copyright, or licensing agreement, or the right to receive royalties from product commercialization- individually or collectively, having an ownership interest (equity or stock options) in the sponsor of the research or product being tested whose value cannot be readily determined through reference to public prices- individually or collectively, having an ownership interest (equity or stock options) in a company or product whose value could be affected by the study outcome- IN THE AGGREGATE, having an ownership interest (equity or stock options) in the sponsor of the research that exceeds $10,000 or 1% when the sponsor is a publicly traded entity- receiving significant payments of other sorts with an aggregate value of $10,000 or more (or payment of ANY amount to medical school or hospital employees) made directly by the sponsor of this research for unrestricted research or education, equipment, consultancy, or honorarium- holding a position of management or leadership in company or entity related to this research including, but not limited to, officer, director, or member of an advisory board.- providing consulting services or serve on a Speaker’s Bureau, either paid or unpaid, to the financial or non-financial sponsor of this study- when the sponsor is a publicly traded entity, having any ownership interest (equity or stock options) in the sponsor- expecting to receive any loans, educational support, contributions of in-kind for equipment, or any other non-compensatory payment from the sponsor of the research in the next year | | no | |  | | |  |  | | --- | --- | | |  | | --- | |  | | |  | | | |  |  |  |  |  | | --- | --- | --- | --- | --- | | |  | | --- | | C2  Please provide a detailed description of the outside interest in the box below. | |  | |  | |  | | |  |  | | --- | --- | | |  | | --- | |  | | |  | | | |  |  |  |  |  | | --- | --- | --- | --- | --- | | |  | | --- | | C2.1  Where have you submitted a disclosure of this outside interest? | |  | |  | |  | | |  |  | | --- | --- | | |  | | --- | |  | | |  | | | |  |  |  |  |  | | --- | --- | --- | --- | --- | | |  | | --- | | C2.2  Has a management plan been formalized? | |  | |  | |  | | |  |  | | --- | --- | | |  | | --- | |  | | |  | | | |  |  |  |  |  |  |  |  |  |  |  |  |  |  |  |  |  |  | | --- | --- | --- | --- | --- | --- | --- | --- | --- | --- | --- | --- | --- | --- | --- | --- | --- | --- | | |  | | --- | | C2.2.1  If yes, attach the management plan here, as well as any other applicable conflict of interest documents. | |  | | |  |  |  |  |  |  |  |  |  |  |  |  |  | | --- | --- | --- | --- | --- | --- | --- | --- | --- | --- | --- | --- | --- | | | Name | Version | | --- | --- | |  |  |  |  |  |  |  |  |  |  | | --- | --- | --- | --- | --- | --- | --- | --- | --- | --- | | There are no items to display | | | | | | | | | | | | |  | | |  |  | | --- | --- | | |  | | --- | |  | | |  | | | |  |  |  |  |  | | --- | --- | --- | --- | --- | | |  | | --- | | C2.2.2  If no, describe the financial interest in sufficient detail to permit the COI Ancillary Committee and the IRB to determine if such involvement represents a potential conflict-of-interest and/or should be disclosed to potential research subjects in the informed consent form. | |  | |  | |  | | |  |  | | --- | --- | | |  | | --- | |  | | |  | | |
| Study Team Detail     |  |  |  |  |  |  |  |  |  |  |  |  |  |  |  |  |  |  |  |  | | --- | --- | --- | --- | --- | --- | --- | --- | --- | --- | --- | --- | --- | --- | --- | --- | --- | --- | --- | --- | | |  |  |  |  |  |  |  |  |  |  |  |  |  |  |  | | --- | --- | --- | --- | --- | --- | --- | --- | --- | --- | --- | --- | --- | --- | --- | | |  | | --- | | 1.4  Team Member: | |  | | Mack Ruffin     |  |  | | --- | --- | |  |  | | Preferred email: | mruffin@umich.edu | | Business phone | 734-998-7120 | | Business address: | Family Medicine | 1018 Fuller SPC 5708 | 48109-5708 | | |  | | |  |  | | --- | --- | | |  | | --- | |  | | |  | | | |  |  |  |  |  | | --- | --- | --- | --- | --- | | |  | | --- | | 1.5  Function with respect to project: | |  | | Co-Investigator | |  | | |  |  | | --- | --- | | |  | | --- | |  | | |  | | | |  |  |  |  |  | | --- | --- | --- | --- | --- | | |  | | --- | | 1.6  Allow this person to EDIT the application, including any supporting documents/stipulations requested during the review process: | |  | | no | |  | | |  |  | | --- | --- | | |  | | --- | |  | | |  | | | |  |  |  |  |  | | --- | --- | --- | --- | --- | | |  | | --- | | 1.7  Include this person on all correspondences regarding this application: (Note: This will include all committee correspondence, decision outcomes, renewal notices, and adverse event submissions.) | |  | | no | |  | | |  |  | | --- | --- | | |  | | --- | |  | | |  | | | |  |  |  |  |  | | --- | --- | --- | --- | --- | | |  | | --- | | **Credentials: Required for PI, Co-Is and Faculty Advisors** | |  | |  | |  | | |  |  | | --- | --- | | |  | | --- | |  | | |  | | | |  |  |  |  |  |  |  |  |  |  | | --- | --- | --- | --- | --- | --- | --- | --- | --- | --- | | |  | | --- | | Upload or update your CV, resume, or biographical sketch. | |  | | |  |  |  |  |  | | --- | --- | --- | --- | --- | | | Name | Version | | --- | --- | | MRcvFEB16jb.doc | History | 0.04 | | | |  | | |  |  | | --- | --- | | |  | | --- | |  | | |  | | | |  |  |  |  |  | | --- | --- | --- | --- | --- | | |  | | --- | | **Conflict of Interest Detail:  Required for all roles except Administrative Staff** | |  | |  | |  | | |  |  | | --- | --- | | |  | | --- | |  | | |  | | | |  |  |  |  |  |  | | --- | --- | --- | --- | --- | --- | | |  | | --- | | C1  Do you, your spouse, domestic partner, or dependents have any outside interests or relationships to companies or entities related to this research that the IRB should consider? | | **Examples of outside interests include, but are not limited to the following:** | | - receiving compensation whose value could be affected by the study outcome- IN THE AGGREGATE, expecting to receive compensation from the sponsor of the research of $10,000 or greater in the next year- having a proprietary interest in the sponsor of the research or a product tested by this research including but not limited to, a patent, trademark, copyright, or licensing agreement, or the right to receive royalties from product commercialization- individually or collectively, having an ownership interest (equity or stock options) in the sponsor of the research or product being tested whose value cannot be readily determined through reference to public prices- individually or collectively, having an ownership interest (equity or stock options) in a company or product whose value could be affected by the study outcome- IN THE AGGREGATE, having an ownership interest (equity or stock options) in the sponsor of the research that exceeds $10,000 or 1% when the sponsor is a publicly traded entity- receiving significant payments of other sorts with an aggregate value of $10,000 or more (or payment of ANY amount to medical school or hospital employees) made directly by the sponsor of this research for unrestricted research or education, equipment, consultancy, or honorarium- holding a position of management or leadership in company or entity related to this research including, but not limited to, officer, director, or member of an advisory board.- providing consulting services or serve on a Speaker’s Bureau, either paid or unpaid, to the financial or non-financial sponsor of this study- when the sponsor is a publicly traded entity, having any ownership interest (equity or stock options) in the sponsor- expecting to receive any loans, educational support, contributions of in-kind for equipment, or any other non-compensatory payment from the sponsor of the research in the next year | | no | |  | | |  |  | | --- | --- | | |  | | --- | |  | | |  | | | |  |  |  |  |  | | --- | --- | --- | --- | --- | | |  | | --- | | C2  Please provide a detailed description of the outside interest in the box below. | |  | |  | |  | | |  |  | | --- | --- | | |  | | --- | |  | | |  | | | |  |  |  |  |  | | --- | --- | --- | --- | --- | | |  | | --- | | C2.1  Where have you submitted a disclosure of this outside interest? | |  | |  | |  | | |  |  | | --- | --- | | |  | | --- | |  | | |  | | | |  |  |  |  |  | | --- | --- | --- | --- | --- | | |  | | --- | | C2.2  Has a management plan been formalized? | |  | |  | |  | | |  |  | | --- | --- | | |  | | --- | |  | | |  | | | |  |  |  |  |  |  |  |  |  |  |  |  |  |  |  |  |  |  | | --- | --- | --- | --- | --- | --- | --- | --- | --- | --- | --- | --- | --- | --- | --- | --- | --- | --- | | |  | | --- | | C2.2.1  If yes, attach the management plan here, as well as any other applicable conflict of interest documents. | |  | | |  |  |  |  |  |  |  |  |  |  |  |  |  | | --- | --- | --- | --- | --- | --- | --- | --- | --- | --- | --- | --- | --- | | | Name | Version | | --- | --- | |  |  |  |  |  |  |  |  |  |  | | --- | --- | --- | --- | --- | --- | --- | --- | --- | --- | | There are no items to display | | | | | | | | | | | | |  | | |  |  | | --- | --- | | |  | | --- | |  | | |  | | | |  |  |  |  |  | | --- | --- | --- | --- | --- | | |  | | --- | | C2.2.2  If no, describe the financial interest in sufficient detail to permit the COI Ancillary Committee and the IRB to determine if such involvement represents a potential conflict-of-interest and/or should be disclosed to potential research subjects in the informed consent form. | |  | |  | |  | | |  |  | | --- | --- | | |  | | --- | |  | | |  | | |
| Study Team Detail     |  |  |  |  |  |  |  |  |  |  |  |  |  |  |  |  |  |  |  |  | | --- | --- | --- | --- | --- | --- | --- | --- | --- | --- | --- | --- | --- | --- | --- | --- | --- | --- | --- | --- | | |  |  |  |  |  |  |  |  |  |  |  |  |  |  |  | | --- | --- | --- | --- | --- | --- | --- | --- | --- | --- | --- | --- | --- | --- | --- | | |  | | --- | | 1.4  Team Member: | |  | | Ananda Sen     |  |  | | --- | --- | |  |  | | Preferred email: | anandas@umich.edu | | Business phone | 734-998-7120 | | Business address: | Family Medicine | 1018 Fuller St | 48109-1213 | | |  | | |  |  | | --- | --- | | |  | | --- | |  | | |  | | | |  |  |  |  |  | | --- | --- | --- | --- | --- | | |  | | --- | | 1.5  Function with respect to project: | |  | | Co-Investigator | |  | | |  |  | | --- | --- | | |  | | --- | |  | | |  | | | |  |  |  |  |  | | --- | --- | --- | --- | --- | | |  | | --- | | 1.6  Allow this person to EDIT the application, including any supporting documents/stipulations requested during the review process: | |  | | no | |  | | |  |  | | --- | --- | | |  | | --- | |  | | |  | | | |  |  |  |  |  | | --- | --- | --- | --- | --- | | |  | | --- | | 1.7  Include this person on all correspondences regarding this application: (Note: This will include all committee correspondence, decision outcomes, renewal notices, and adverse event submissions.) | |  | | no | |  | | |  |  | | --- | --- | | |  | | --- | |  | | |  | | | |  |  |  |  |  | | --- | --- | --- | --- | --- | | |  | | --- | | **Credentials: Required for PI, Co-Is and Faculty Advisors** | |  | |  | |  | | |  |  | | --- | --- | | |  | | --- | |  | | |  | | | |  |  |  |  |  |  |  |  |  |  | | --- | --- | --- | --- | --- | --- | --- | --- | --- | --- | | |  | | --- | | Upload or update your CV, resume, or biographical sketch. | |  | | |  |  |  |  |  | | --- | --- | --- | --- | --- | | | Name | Version | | --- | --- | | CV Sen 8-29-12.doc | History | 0.01 | | | |  | | |  |  | | --- | --- | | |  | | --- | |  | | |  | | | |  |  |  |  |  | | --- | --- | --- | --- | --- | | |  | | --- | | **Conflict of Interest Detail:  Required for all roles except Administrative Staff** | |  | |  | |  | | |  |  | | --- | --- | | |  | | --- | |  | | |  | | | |  |  |  |  |  |  | | --- | --- | --- | --- | --- | --- | | |  | | --- | | C1  Do you, your spouse, domestic partner, or dependents have any outside interests or relationships to companies or entities related to this research that the IRB should consider? | | **Examples of outside interests include, but are not limited to the following:** | | - receiving compensation whose value could be affected by the study outcome- IN THE AGGREGATE, expecting to receive compensation from the sponsor of the research of $10,000 or greater in the next year- having a proprietary interest in the sponsor of the research or a product tested by this research including but not limited to, a patent, trademark, copyright, or licensing agreement, or the right to receive royalties from product commercialization- individually or collectively, having an ownership interest (equity or stock options) in the sponsor of the research or product being tested whose value cannot be readily determined through reference to public prices- individually or collectively, having an ownership interest (equity or stock options) in a company or product whose value could be affected by the study outcome- IN THE AGGREGATE, having an ownership interest (equity or stock options) in the sponsor of the research that exceeds $10,000 or 1% when the sponsor is a publicly traded entity- receiving significant payments of other sorts with an aggregate value of $10,000 or more (or payment of ANY amount to medical school or hospital employees) made directly by the sponsor of this research for unrestricted research or education, equipment, consultancy, or honorarium- holding a position of management or leadership in company or entity related to this research including, but not limited to, officer, director, or member of an advisory board.- providing consulting services or serve on a Speaker’s Bureau, either paid or unpaid, to the financial or non-financial sponsor of this study- when the sponsor is a publicly traded entity, having any ownership interest (equity or stock options) in the sponsor- expecting to receive any loans, educational support, contributions of in-kind for equipment, or any other non-compensatory payment from the sponsor of the research in the next year | | no | |  | | |  |  | | --- | --- | | |  | | --- | |  | | |  | | | |  |  |  |  |  | | --- | --- | --- | --- | --- | | |  | | --- | | C2  Please provide a detailed description of the outside interest in the box below. | |  | |  | |  | | |  |  | | --- | --- | | |  | | --- | |  | | |  | | | |  |  |  |  |  | | --- | --- | --- | --- | --- | | |  | | --- | | C2.1  Where have you submitted a disclosure of this outside interest? | |  | |  | |  | | |  |  | | --- | --- | | |  | | --- | |  | | |  | | | |  |  |  |  |  | | --- | --- | --- | --- | --- | | |  | | --- | | C2.2  Has a management plan been formalized? | |  | |  | |  | | |  |  | | --- | --- | | |  | | --- | |  | | |  | | | |  |  |  |  |  |  |  |  |  |  |  |  |  |  |  |  |  |  | | --- | --- | --- | --- | --- | --- | --- | --- | --- | --- | --- | --- | --- | --- | --- | --- | --- | --- | | |  | | --- | | C2.2.1  If yes, attach the management plan here, as well as any other applicable conflict of interest documents. | |  | | |  |  |  |  |  |  |  |  |  |  |  |  |  | | --- | --- | --- | --- | --- | --- | --- | --- | --- | --- | --- | --- | --- | | | Name | Version | | --- | --- | |  |  |  |  |  |  |  |  |  |  | | --- | --- | --- | --- | --- | --- | --- | --- | --- | --- | | There are no items to display | | | | | | | | | | | | |  | | |  |  | | --- | --- | | |  | | --- | |  | | |  | | | |  |  |  |  |  | | --- | --- | --- | --- | --- | | |  | | --- | | C2.2.2  If no, describe the financial interest in sufficient detail to permit the COI Ancillary Committee and the IRB to determine if such involvement represents a potential conflict-of-interest and/or should be disclosed to potential research subjects in the informed consent form. | |  | |  | |  | | |  |  | | --- | --- | | |  | | --- | |  | | |  | | |
| Study Team Detail     |  |  |  |  |  |  |  |  |  |  |  |  |  |  |  |  |  |  |  |  | | --- | --- | --- | --- | --- | --- | --- | --- | --- | --- | --- | --- | --- | --- | --- | --- | --- | --- | --- | --- | | |  |  |  |  |  |  |  |  |  |  |  |  |  |  |  | | --- | --- | --- | --- | --- | --- | --- | --- | --- | --- | --- | --- | --- | --- | --- | | |  | | --- | | 1.4  Team Member: | |  | | Kathleen Lawrence     |  |  | | --- | --- | |  |  | | Preferred email: | katlaw@umich.edu | | Business phone | 734-998-7120 | | Business address: | Family Medicine | 1018 Fuller ST Rm I1003 UHEC | 48109 | | |  | | |  |  | | --- | --- | | |  | | --- | |  | | |  | | | |  |  |  |  |  | | --- | --- | --- | --- | --- | | |  | | --- | | 1.5  Function with respect to project: | |  | | Study Coordinator/Project Manager | |  | | |  |  | | --- | --- | | |  | | --- | |  | | |  | | | |  |  |  |  |  | | --- | --- | --- | --- | --- | | |  | | --- | | 1.6  Allow this person to EDIT the application, including any supporting documents/stipulations requested during the review process: | |  | | yes | |  | | |  |  | | --- | --- | | |  | | --- | |  | | |  | | | |  |  |  |  |  | | --- | --- | --- | --- | --- | | |  | | --- | | 1.7  Include this person on all correspondences regarding this application: (Note: This will include all committee correspondence, decision outcomes, renewal notices, and adverse event submissions.) | |  | | no | |  | | |  |  | | --- | --- | | |  | | --- | |  | | |  | | | |  |  |  |  |  | | --- | --- | --- | --- | --- | | |  | | --- | | **Credentials: Required for PI, Co-Is and Faculty Advisors** | |  | |  | |  | | |  |  | | --- | --- | | |  | | --- | |  | | |  | | | |  |  |  |  |  |  |  |  |  |  |  |  |  |  |  |  |  |  | | --- | --- | --- | --- | --- | --- | --- | --- | --- | --- | --- | --- | --- | --- | --- | --- | --- | --- | | |  | | --- | | Upload or update your CV, resume, or biographical sketch. | |  | | |  |  |  |  |  |  |  |  |  |  |  |  |  | | --- | --- | --- | --- | --- | --- | --- | --- | --- | --- | --- | --- | --- | | | Name | Version | | --- | --- | |  |  |  |  |  |  |  |  |  |  | | --- | --- | --- | --- | --- | --- | --- | --- | --- | --- | | There are no items to display | | | | | | | | | | | | |  | | |  |  | | --- | --- | | |  | | --- | |  | | |  | | | |  |  |  |  |  | | --- | --- | --- | --- | --- | | |  | | --- | | **Conflict of Interest Detail:  Required for all roles except Administrative Staff** | |  | |  | |  | | |  |  | | --- | --- | | |  | | --- | |  | | |  | | | |  |  |  |  |  |  | | --- | --- | --- | --- | --- | --- | | |  | | --- | | C1  Do you, your spouse, domestic partner, or dependents have any outside interests or relationships to companies or entities related to this research that the IRB should consider? | | **Examples of outside interests include, but are not limited to the following:** | | - receiving compensation whose value could be affected by the study outcome- IN THE AGGREGATE, expecting to receive compensation from the sponsor of the research of $10,000 or greater in the next year- having a proprietary interest in the sponsor of the research or a product tested by this research including but not limited to, a patent, trademark, copyright, or licensing agreement, or the right to receive royalties from product commercialization- individually or collectively, having an ownership interest (equity or stock options) in the sponsor of the research or product being tested whose value cannot be readily determined through reference to public prices- individually or collectively, having an ownership interest (equity or stock options) in a company or product whose value could be affected by the study outcome- IN THE AGGREGATE, having an ownership interest (equity or stock options) in the sponsor of the research that exceeds $10,000 or 1% when the sponsor is a publicly traded entity- receiving significant payments of other sorts with an aggregate value of $10,000 or more (or payment of ANY amount to medical school or hospital employees) made directly by the sponsor of this research for unrestricted research or education, equipment, consultancy, or honorarium- holding a position of management or leadership in company or entity related to this research including, but not limited to, officer, director, or member of an advisory board.- providing consulting services or serve on a Speaker’s Bureau, either paid or unpaid, to the financial or non-financial sponsor of this study- when the sponsor is a publicly traded entity, having any ownership interest (equity or stock options) in the sponsor- expecting to receive any loans, educational support, contributions of in-kind for equipment, or any other non-compensatory payment from the sponsor of the research in the next year | | no | |  | | |  |  | | --- | --- | | |  | | --- | |  | | |  | | | |  |  |  |  |  | | --- | --- | --- | --- | --- | | |  | | --- | | C2  Please provide a detailed description of the outside interest in the box below. | |  | |  | |  | | |  |  | | --- | --- | | |  | | --- | |  | | |  | | | |  |  |  |  |  | | --- | --- | --- | --- | --- | | |  | | --- | | C2.1  Where have you submitted a disclosure of this outside interest? | |  | |  | |  | | |  |  | | --- | --- | | |  | | --- | |  | | |  | | | |  |  |  |  |  | | --- | --- | --- | --- | --- | | |  | | --- | | C2.2  Has a management plan been formalized? | |  | |  | |  | | |  |  | | --- | --- | | |  | | --- | |  | | |  | | | |  |  |  |  |  |  |  |  |  |  |  |  |  |  |  |  |  |  | | --- | --- | --- | --- | --- | --- | --- | --- | --- | --- | --- | --- | --- | --- | --- | --- | --- | --- | | |  | | --- | | C2.2.1  If yes, attach the management plan here, as well as any other applicable conflict of interest documents. | |  | | |  |  |  |  |  |  |  |  |  |  |  |  |  | | --- | --- | --- | --- | --- | --- | --- | --- | --- | --- | --- | --- | --- | | | Name | Version | | --- | --- | |  |  |  |  |  |  |  |  |  |  | | --- | --- | --- | --- | --- | --- | --- | --- | --- | --- | | There are no items to display | | | | | | | | | | | | |  | | |  |  | | --- | --- | | |  | | --- | |  | | |  | | | |  |  |  |  |  | | --- | --- | --- | --- | --- | | |  | | --- | | C2.2.2  If no, describe the financial interest in sufficient detail to permit the COI Ancillary Committee and the IRB to determine if such involvement represents a potential conflict-of-interest and/or should be disclosed to potential research subjects in the informed consent form. | |  | |  | |  | | |  |  | | --- | --- | | |  | | --- | |  | | |  | | |
| Study Team Detail     |  |  |  |  |  |  |  |  |  |  |  |  |  |  |  |  |  |  |  |  | | --- | --- | --- | --- | --- | --- | --- | --- | --- | --- | --- | --- | --- | --- | --- | --- | --- | --- | --- | --- | | |  |  |  |  |  |  |  |  |  |  |  |  |  |  |  | | --- | --- | --- | --- | --- | --- | --- | --- | --- | --- | --- | --- | --- | --- | --- | | |  | | --- | | 1.4  Team Member: | |  | | Mary Rapai     |  |  | | --- | --- | |  |  | | Preferred email: | mrapai@umich.edu | | Business phone | 734-998-7120 | | Business address: | Family Medicine | Room I3001, 1018 Fuller St | 48104-1213 | | |  | | |  |  | | --- | --- | | |  | | --- | |  | | |  | | | |  |  |  |  |  | | --- | --- | --- | --- | --- | | |  | | --- | | 1.5  Function with respect to project: | |  | | Study Coordinator/Project Manager | |  | | |  |  | | --- | --- | | |  | | --- | |  | | |  | | | |  |  |  |  |  | | --- | --- | --- | --- | --- | | |  | | --- | | 1.6  Allow this person to EDIT the application, including any supporting documents/stipulations requested during the review process: | |  | | yes | |  | | |  |  | | --- | --- | | |  | | --- | |  | | |  | | | |  |  |  |  |  | | --- | --- | --- | --- | --- | | |  | | --- | | 1.7  Include this person on all correspondences regarding this application: (Note: This will include all committee correspondence, decision outcomes, renewal notices, and adverse event submissions.) | |  | | yes | |  | | |  |  | | --- | --- | | |  | | --- | |  | | |  | | | |  |  |  |  |  | | --- | --- | --- | --- | --- | | |  | | --- | | **Credentials: Required for PI, Co-Is and Faculty Advisors** | |  | |  | |  | | |  |  | | --- | --- | | |  | | --- | |  | | |  | | | |  |  |  |  |  |  |  |  |  |  | | --- | --- | --- | --- | --- | --- | --- | --- | --- | --- | | |  | | --- | | Upload or update your CV, resume, or biographical sketch. | |  | | |  |  |  |  |  | | --- | --- | --- | --- | --- | | | Name | Version | | --- | --- | | resume | History | 0.02 | | | |  | | |  |  | | --- | --- | | |  | | --- | |  | | |  | | | |  |  |  |  |  | | --- | --- | --- | --- | --- | | |  | | --- | | **Conflict of Interest Detail:  Required for all roles except Administrative Staff** | |  | |  | |  | | |  |  | | --- | --- | | |  | | --- | |  | | |  | | | |  |  |  |  |  |  | | --- | --- | --- | --- | --- | --- | | |  | | --- | | C1  Do you, your spouse, domestic partner, or dependents have any outside interests or relationships to companies or entities related to this research that the IRB should consider? | | **Examples of outside interests include, but are not limited to the following:** | | - receiving compensation whose value could be affected by the study outcome- IN THE AGGREGATE, expecting to receive compensation from the sponsor of the research of $10,000 or greater in the next year- having a proprietary interest in the sponsor of the research or a product tested by this research including but not limited to, a patent, trademark, copyright, or licensing agreement, or the right to receive royalties from product commercialization- individually or collectively, having an ownership interest (equity or stock options) in the sponsor of the research or product being tested whose value cannot be readily determined through reference to public prices- individually or collectively, having an ownership interest (equity or stock options) in a company or product whose value could be affected by the study outcome- IN THE AGGREGATE, having an ownership interest (equity or stock options) in the sponsor of the research that exceeds $10,000 or 1% when the sponsor is a publicly traded entity- receiving significant payments of other sorts with an aggregate value of $10,000 or more (or payment of ANY amount to medical school or hospital employees) made directly by the sponsor of this research for unrestricted research or education, equipment, consultancy, or honorarium- holding a position of management or leadership in company or entity related to this research including, but not limited to, officer, director, or member of an advisory board.- providing consulting services or serve on a Speaker’s Bureau, either paid or unpaid, to the financial or non-financial sponsor of this study- when the sponsor is a publicly traded entity, having any ownership interest (equity or stock options) in the sponsor- expecting to receive any loans, educational support, contributions of in-kind for equipment, or any other non-compensatory payment from the sponsor of the research in the next year | | no | |  | | |  |  | | --- | --- | | |  | | --- | |  | | |  | | | |  |  |  |  |  | | --- | --- | --- | --- | --- | | |  | | --- | | C2  Please provide a detailed description of the outside interest in the box below. | |  | |  | |  | | |  |  | | --- | --- | | |  | | --- | |  | | |  | | | |  |  |  |  |  | | --- | --- | --- | --- | --- | | |  | | --- | | C2.1  Where have you submitted a disclosure of this outside interest? | |  | |  | |  | | |  |  | | --- | --- | | |  | | --- | |  | | |  | | | |  |  |  |  |  | | --- | --- | --- | --- | --- | | |  | | --- | | C2.2  Has a management plan been formalized? | |  | |  | |  | | |  |  | | --- | --- | | |  | | --- | |  | | |  | | | |  |  |  |  |  |  |  |  |  |  |  |  |  |  |  |  |  |  | | --- | --- | --- | --- | --- | --- | --- | --- | --- | --- | --- | --- | --- | --- | --- | --- | --- | --- | | |  | | --- | | C2.2.1  If yes, attach the management plan here, as well as any other applicable conflict of interest documents. | |  | | |  |  |  |  |  |  |  |  |  |  |  |  |  | | --- | --- | --- | --- | --- | --- | --- | --- | --- | --- | --- | --- | --- | | | Name | Version | | --- | --- | |  |  |  |  |  |  |  |  |  |  | | --- | --- | --- | --- | --- | --- | --- | --- | --- | --- | | There are no items to display | | | | | | | | | | | | |  | | |  |  | | --- | --- | | |  | | --- | |  | | |  | | | |  |  |  |  |  | | --- | --- | --- | --- | --- | | |  | | --- | | C2.2.2  If no, describe the financial interest in sufficient detail to permit the COI Ancillary Committee and the IRB to determine if such involvement represents a potential conflict-of-interest and/or should be disclosed to potential research subjects in the informed consent form. | |  | |  | |  | | |  |  | | --- | --- | | |  | | --- | |  | | |  | | |
| Study Team Detail     |  |  |  |  |  |  |  |  |  |  |  |  |  |  |  |  |  |  |  |  | | --- | --- | --- | --- | --- | --- | --- | --- | --- | --- | --- | --- | --- | --- | --- | --- | --- | --- | --- | --- | | |  |  |  |  |  |  |  |  |  |  |  |  |  |  |  | | --- | --- | --- | --- | --- | --- | --- | --- | --- | --- | --- | --- | --- | --- | --- | | |  | | --- | | 1.4  Team Member: | |  | | Ronald Trendler     |  |  | | --- | --- | |  |  | | Preferred email: | rtrendle@umich.edu | | Business phone | 734-998-7120 | | Business address: | Family Medicine | 1018 Fuller St | 48109 | | |  | | |  |  | | --- | --- | | |  | | --- | |  | | |  | | | |  |  |  |  |  | | --- | --- | --- | --- | --- | | |  | | --- | | 1.5  Function with respect to project: | |  | | Study Coordinator/Project Manager | |  | | |  |  | | --- | --- | | |  | | --- | |  | | |  | | | |  |  |  |  |  | | --- | --- | --- | --- | --- | | |  | | --- | | 1.6  Allow this person to EDIT the application, including any supporting documents/stipulations requested during the review process: | |  | | yes | |  | | |  |  | | --- | --- | | |  | | --- | |  | | |  | | | |  |  |  |  |  | | --- | --- | --- | --- | --- | | |  | | --- | | 1.7  Include this person on all correspondences regarding this application: (Note: This will include all committee correspondence, decision outcomes, renewal notices, and adverse event submissions.) | |  | | yes | |  | | |  |  | | --- | --- | | |  | | --- | |  | | |  | | | |  |  |  |  |  | | --- | --- | --- | --- | --- | | |  | | --- | | **Credentials: Required for PI, Co-Is and Faculty Advisors** | |  | |  | |  | | |  |  | | --- | --- | | |  | | --- | |  | | |  | | | |  |  |  |  |  |  |  |  |  |  |  |  |  |  |  |  |  |  | | --- | --- | --- | --- | --- | --- | --- | --- | --- | --- | --- | --- | --- | --- | --- | --- | --- | --- | | |  | | --- | | Upload or update your CV, resume, or biographical sketch. | |  | | |  |  |  |  |  |  |  |  |  |  |  |  |  | | --- | --- | --- | --- | --- | --- | --- | --- | --- | --- | --- | --- | --- | | | Name | Version | | --- | --- | |  |  |  |  |  |  |  |  |  |  | | --- | --- | --- | --- | --- | --- | --- | --- | --- | --- | | There are no items to display | | | | | | | | | | | | |  | | |  |  | | --- | --- | | |  | | --- | |  | | |  | | | |  |  |  |  |  | | --- | --- | --- | --- | --- | | |  | | --- | | **Conflict of Interest Detail:  Required for all roles except Administrative Staff** | |  | |  | |  | | |  |  | | --- | --- | | |  | | --- | |  | | |  | | | |  |  |  |  |  |  | | --- | --- | --- | --- | --- | --- | | |  | | --- | | C1  Do you, your spouse, domestic partner, or dependents have any outside interests or relationships to companies or entities related to this research that the IRB should consider? | | **Examples of outside interests include, but are not limited to the following:** | | - receiving compensation whose value could be affected by the study outcome- IN THE AGGREGATE, expecting to receive compensation from the sponsor of the research of $10,000 or greater in the next year- having a proprietary interest in the sponsor of the research or a product tested by this research including but not limited to, a patent, trademark, copyright, or licensing agreement, or the right to receive royalties from product commercialization- individually or collectively, having an ownership interest (equity or stock options) in the sponsor of the research or product being tested whose value cannot be readily determined through reference to public prices- individually or collectively, having an ownership interest (equity or stock options) in a company or product whose value could be affected by the study outcome- IN THE AGGREGATE, having an ownership interest (equity or stock options) in the sponsor of the research that exceeds $10,000 or 1% when the sponsor is a publicly traded entity- receiving significant payments of other sorts with an aggregate value of $10,000 or more (or payment of ANY amount to medical school or hospital employees) made directly by the sponsor of this research for unrestricted research or education, equipment, consultancy, or honorarium- holding a position of management or leadership in company or entity related to this research including, but not limited to, officer, director, or member of an advisory board.- providing consulting services or serve on a Speaker’s Bureau, either paid or unpaid, to the financial or non-financial sponsor of this study- when the sponsor is a publicly traded entity, having any ownership interest (equity or stock options) in the sponsor- expecting to receive any loans, educational support, contributions of in-kind for equipment, or any other non-compensatory payment from the sponsor of the research in the next year | | no | |  | | |  |  | | --- | --- | | |  | | --- | |  | | |  | | | |  |  |  |  |  | | --- | --- | --- | --- | --- | | |  | | --- | | C2  Please provide a detailed description of the outside interest in the box below. | |  | |  | |  | | |  |  | | --- | --- | | |  | | --- | |  | | |  | | | |  |  |  |  |  | | --- | --- | --- | --- | --- | | |  | | --- | | C2.1  Where have you submitted a disclosure of this outside interest? | |  | |  | |  | | |  |  | | --- | --- | | |  | | --- | |  | | |  | | | |  |  |  |  |  | | --- | --- | --- | --- | --- | | |  | | --- | | C2.2  Has a management plan been formalized? | |  | |  | |  | | |  |  | | --- | --- | | |  | | --- | |  | | |  | | | |  |  |  |  |  |  |  |  |  |  |  |  |  |  |  |  |  |  | | --- | --- | --- | --- | --- | --- | --- | --- | --- | --- | --- | --- | --- | --- | --- | --- | --- | --- | | |  | | --- | | C2.2.1  If yes, attach the management plan here, as well as any other applicable conflict of interest documents. | |  | | |  |  |  |  |  |  |  |  |  |  |  |  |  | | --- | --- | --- | --- | --- | --- | --- | --- | --- | --- | --- | --- | --- | | | Name | Version | | --- | --- | |  |  |  |  |  |  |  |  |  |  | | --- | --- | --- | --- | --- | --- | --- | --- | --- | --- | | There are no items to display | | | | | | | | | | | | |  | | |  |  | | --- | --- | | |  | | --- | |  | | |  | | | |  |  |  |  |  | | --- | --- | --- | --- | --- | | |  | | --- | | C2.2.2  If no, describe the financial interest in sufficient detail to permit the COI Ancillary Committee and the IRB to determine if such involvement represents a potential conflict-of-interest and/or should be disclosed to potential research subjects in the informed consent form. | |  | |  | |  | | |  |  | | --- | --- | | |  | | --- | |  | | |  | | |
| Study Team Detail     |  |  |  |  |  |  |  |  |  |  |  |  |  |  |  |  |  |  |  |  | | --- | --- | --- | --- | --- | --- | --- | --- | --- | --- | --- | --- | --- | --- | --- | --- | --- | --- | --- | --- | | |  |  |  |  |  |  |  |  |  |  |  |  |  |  |  | | --- | --- | --- | --- | --- | --- | --- | --- | --- | --- | --- | --- | --- | --- | --- | | |  | | --- | | 1.4  Team Member: | |  | | Donald Nease Jr     |  |  | | --- | --- | |  |  | | Preferred email: | dnease@umich.edu | | Business phone |  | | Business address: | Family Medicine | 1018 Fuller St SPC 5708 | 48109-5708 | | |  | | |  |  | | --- | --- | | |  | | --- | |  | | |  | | | |  |  |  |  |  | | --- | --- | --- | --- | --- | | |  | | --- | | 1.5  Function with respect to project: | |  | | Consultant | |  | | |  |  | | --- | --- | | |  | | --- | |  | | |  | | | |  |  |  |  |  | | --- | --- | --- | --- | --- | | |  | | --- | | 1.6  Allow this person to EDIT the application, including any supporting documents/stipulations requested during the review process: | |  | | no | |  | | |  |  | | --- | --- | | |  | | --- | |  | | |  | | | |  |  |  |  |  | | --- | --- | --- | --- | --- | | |  | | --- | | 1.7  Include this person on all correspondences regarding this application: (Note: This will include all committee correspondence, decision outcomes, renewal notices, and adverse event submissions.) | |  | | no | |  | | |  |  | | --- | --- | | |  | | --- | |  | | |  | | | |  |  |  |  |  | | --- | --- | --- | --- | --- | | |  | | --- | | **Credentials: Required for PI, Co-Is and Faculty Advisors** | |  | |  | |  | | |  |  | | --- | --- | | |  | | --- | |  | | |  | | | |  |  |  |  |  |  |  |  |  |  | | --- | --- | --- | --- | --- | --- | --- | --- | --- | --- | | |  | | --- | | Upload or update your CV, resume, or biographical sketch. | |  | | |  |  |  |  |  | | --- | --- | --- | --- | --- | | | Name | Version | | --- | --- | | Nease cv | History | 0.04 | | | |  | | |  |  | | --- | --- | | |  | | --- | |  | | |  | | | |  |  |  |  |  | | --- | --- | --- | --- | --- | | |  | | --- | | **Conflict of Interest Detail:  Required for all roles except Administrative Staff** | |  | |  | |  | | |  |  | | --- | --- | | |  | | --- | |  | | |  | | | |  |  |  |  |  |  | | --- | --- | --- | --- | --- | --- | | |  | | --- | | C1  Do you, your spouse, domestic partner, or dependents have any outside interests or relationships to companies or entities related to this research that the IRB should consider? | | **Examples of outside interests include, but are not limited to the following:** | | - receiving compensation whose value could be affected by the study outcome- IN THE AGGREGATE, expecting to receive compensation from the sponsor of the research of $10,000 or greater in the next year- having a proprietary interest in the sponsor of the research or a product tested by this research including but not limited to, a patent, trademark, copyright, or licensing agreement, or the right to receive royalties from product commercialization- individually or collectively, having an ownership interest (equity or stock options) in the sponsor of the research or product being tested whose value cannot be readily determined through reference to public prices- individually or collectively, having an ownership interest (equity or stock options) in a company or product whose value could be affected by the study outcome- IN THE AGGREGATE, having an ownership interest (equity or stock options) in the sponsor of the research that exceeds $10,000 or 1% when the sponsor is a publicly traded entity- receiving significant payments of other sorts with an aggregate value of $10,000 or more (or payment of ANY amount to medical school or hospital employees) made directly by the sponsor of this research for unrestricted research or education, equipment, consultancy, or honorarium- holding a position of management or leadership in company or entity related to this research including, but not limited to, officer, director, or member of an advisory board.- providing consulting services or serve on a Speaker’s Bureau, either paid or unpaid, to the financial or non-financial sponsor of this study- when the sponsor is a publicly traded entity, having any ownership interest (equity or stock options) in the sponsor- expecting to receive any loans, educational support, contributions of in-kind for equipment, or any other non-compensatory payment from the sponsor of the research in the next year | | no | |  | | |  |  | | --- | --- | | |  | | --- | |  | | |  | | | |  |  |  |  |  | | --- | --- | --- | --- | --- | | |  | | --- | | C2  Please provide a detailed description of the outside interest in the box below. | |  | |  | |  | | |  |  | | --- | --- | | |  | | --- | |  | | |  | | | |  |  |  |  |  | | --- | --- | --- | --- | --- | | |  | | --- | | C2.1  Where have you submitted a disclosure of this outside interest? | |  | |  | |  | | |  |  | | --- | --- | | |  | | --- | |  | | |  | | | |  |  |  |  |  | | --- | --- | --- | --- | --- | | |  | | --- | | C2.2  Has a management plan been formalized? | |  | |  | |  | | |  |  | | --- | --- | | |  | | --- | |  | | |  | | | |  |  |  |  |  |  |  |  |  |  |  |  |  |  |  |  |  |  | | --- | --- | --- | --- | --- | --- | --- | --- | --- | --- | --- | --- | --- | --- | --- | --- | --- | --- | | |  | | --- | | C2.2.1  If yes, attach the management plan here, as well as any other applicable conflict of interest documents. | |  | | |  |  |  |  |  |  |  |  |  |  |  |  |  | | --- | --- | --- | --- | --- | --- | --- | --- | --- | --- | --- | --- | --- | | | Name | Version | | --- | --- | |  |  |  |  |  |  |  |  |  |  | | --- | --- | --- | --- | --- | --- | --- | --- | --- | --- | | There are no items to display | | | | | | | | | | | | |  | | |  |  | | --- | --- | | |  | | --- | |  | | |  | | | |  |  |  |  |  | | --- | --- | --- | --- | --- | | |  | | --- | | C2.2.2  If no, describe the financial interest in sufficient detail to permit the COI Ancillary Committee and the IRB to determine if such involvement represents a potential conflict-of-interest and/or should be disclosed to potential research subjects in the informed consent form. | |  | |  | |  | | |  |  | | --- | --- | | |  | | --- | |  | | |  | | |
| Study Team Detail     |  |  |  |  |  |  |  |  |  |  |  |  |  |  |  |  |  |  |  |  | | --- | --- | --- | --- | --- | --- | --- | --- | --- | --- | --- | --- | --- | --- | --- | --- | --- | --- | --- | --- | | |  |  |  |  |  |  |  |  |  |  |  |  |  |  |  | | --- | --- | --- | --- | --- | --- | --- | --- | --- | --- | --- | --- | --- | --- | --- | | |  | | --- | | 1.4  Team Member: | |  | | Tonia Gooden     |  |  | | --- | --- | |  |  | | Preferred email: | tagooden@umich.edu | | Business phone |  | | Business address: |  |  | 48109 | | |  | | |  |  | | --- | --- | | |  | | --- | |  | | |  | | | |  |  |  |  |  | | --- | --- | --- | --- | --- | | |  | | --- | | 1.5  Function with respect to project: | |  | | Research Staff | |  | | |  |  | | --- | --- | | |  | | --- | |  | | |  | | | |  |  |  |  |  | | --- | --- | --- | --- | --- | | |  | | --- | | 1.6  Allow this person to EDIT the application, including any supporting documents/stipulations requested during the review process: | |  | | yes | |  | | |  |  | | --- | --- | | |  | | --- | |  | | |  | | | |  |  |  |  |  | | --- | --- | --- | --- | --- | | |  | | --- | | 1.7  Include this person on all correspondences regarding this application: (Note: This will include all committee correspondence, decision outcomes, renewal notices, and adverse event submissions.) | |  | | no | |  | | |  |  | | --- | --- | | |  | | --- | |  | | |  | | | |  |  |  |  |  | | --- | --- | --- | --- | --- | | |  | | --- | | **Credentials: Required for PI, Co-Is and Faculty Advisors** | |  | |  | |  | | |  |  | | --- | --- | | |  | | --- | |  | | |  | | | |  |  |  |  |  |  |  |  |  |  |  |  |  |  |  |  |  |  | | --- | --- | --- | --- | --- | --- | --- | --- | --- | --- | --- | --- | --- | --- | --- | --- | --- | --- | | |  | | --- | | Upload or update your CV, resume, or biographical sketch. | |  | | |  |  |  |  |  |  |  |  |  |  |  |  |  | | --- | --- | --- | --- | --- | --- | --- | --- | --- | --- | --- | --- | --- | | | Name | Version | | --- | --- | |  |  |  |  |  |  |  |  |  |  | | --- | --- | --- | --- | --- | --- | --- | --- | --- | --- | | There are no items to display | | | | | | | | | | | | |  | | |  |  | | --- | --- | | |  | | --- | |  | | |  | | | |  |  |  |  |  | | --- | --- | --- | --- | --- | | |  | | --- | | **Conflict of Interest Detail:  Required for all roles except Administrative Staff** | |  | |  | |  | | |  |  | | --- | --- | | |  | | --- | |  | | |  | | | |  |  |  |  |  |  | | --- | --- | --- | --- | --- | --- | | |  | | --- | | C1  Do you, your spouse, domestic partner, or dependents have any outside interests or relationships to companies or entities related to this research that the IRB should consider? | | **Examples of outside interests include, but are not limited to the following:** | | - receiving compensation whose value could be affected by the study outcome- IN THE AGGREGATE, expecting to receive compensation from the sponsor of the research of $10,000 or greater in the next year- having a proprietary interest in the sponsor of the research or a product tested by this research including but not limited to, a patent, trademark, copyright, or licensing agreement, or the right to receive royalties from product commercialization- individually or collectively, having an ownership interest (equity or stock options) in the sponsor of the research or product being tested whose value cannot be readily determined through reference to public prices- individually or collectively, having an ownership interest (equity or stock options) in a company or product whose value could be affected by the study outcome- IN THE AGGREGATE, having an ownership interest (equity or stock options) in the sponsor of the research that exceeds $10,000 or 1% when the sponsor is a publicly traded entity- receiving significant payments of other sorts with an aggregate value of $10,000 or more (or payment of ANY amount to medical school or hospital employees) made directly by the sponsor of this research for unrestricted research or education, equipment, consultancy, or honorarium- holding a position of management or leadership in company or entity related to this research including, but not limited to, officer, director, or member of an advisory board.- providing consulting services or serve on a Speaker’s Bureau, either paid or unpaid, to the financial or non-financial sponsor of this study- when the sponsor is a publicly traded entity, having any ownership interest (equity or stock options) in the sponsor- expecting to receive any loans, educational support, contributions of in-kind for equipment, or any other non-compensatory payment from the sponsor of the research in the next year | | no | |  | | |  |  | | --- | --- | | |  | | --- | |  | | |  | | | |  |  |  |  |  | | --- | --- | --- | --- | --- | | |  | | --- | | C2  Please provide a detailed description of the outside interest in the box below. | |  | |  | |  | | |  |  | | --- | --- | | |  | | --- | |  | | |  | | | |  |  |  |  |  | | --- | --- | --- | --- | --- | | |  | | --- | | C2.1  Where have you submitted a disclosure of this outside interest? | |  | |  | |  | | |  |  | | --- | --- | | |  | | --- | |  | | |  | | | |  |  |  |  |  | | --- | --- | --- | --- | --- | | |  | | --- | | C2.2  Has a management plan been formalized? | |  | |  | |  | | |  |  | | --- | --- | | |  | | --- | |  | | |  | | | |  |  |  |  |  |  |  |  |  |  |  |  |  |  |  |  |  |  | | --- | --- | --- | --- | --- | --- | --- | --- | --- | --- | --- | --- | --- | --- | --- | --- | --- | --- | | |  | | --- | | C2.2.1  If yes, attach the management plan here, as well as any other applicable conflict of interest documents. | |  | | |  |  |  |  |  |  |  |  |  |  |  |  |  | | --- | --- | --- | --- | --- | --- | --- | --- | --- | --- | --- | --- | --- | | | Name | Version | | --- | --- | |  |  |  |  |  |  |  |  |  |  | | --- | --- | --- | --- | --- | --- | --- | --- | --- | --- | | There are no items to display | | | | | | | | | | | | |  | | |  |  | | --- | --- | | |  | | --- | |  | | |  | | | |  |  |  |  |  | | --- | --- | --- | --- | --- | | |  | | --- | | C2.2.2  If no, describe the financial interest in sufficient detail to permit the COI Ancillary Committee and the IRB to determine if such involvement represents a potential conflict-of-interest and/or should be disclosed to potential research subjects in the informed consent form. | |  | |  | |  | | |  |  | | --- | --- | | |  | | --- | |  | | |  | | |
| Study Team Detail     |  |  |  |  |  |  |  |  |  |  |  |  |  |  |  |  |  |  |  |  | | --- | --- | --- | --- | --- | --- | --- | --- | --- | --- | --- | --- | --- | --- | --- | --- | --- | --- | --- | --- | | |  |  |  |  |  |  |  |  |  |  |  |  |  |  |  | | --- | --- | --- | --- | --- | --- | --- | --- | --- | --- | --- | --- | --- | --- | --- | | |  | | --- | | 1.4  Team Member: | |  | | Yuhong Zhang     |  |  | | --- | --- | |  |  | | Preferred email: | yuhzhang@umich.edu | | Business phone | 734-998-7120 | | Business address: | Family Medicine | 1018 Fuller St | 48104-1213 | | |  | | |  |  | | --- | --- | | |  | | --- | |  | | |  | | | |  |  |  |  |  | | --- | --- | --- | --- | --- | | |  | | --- | | 1.5  Function with respect to project: | |  | | Research Staff | |  | | |  |  | | --- | --- | | |  | | --- | |  | | |  | | | |  |  |  |  |  | | --- | --- | --- | --- | --- | | |  | | --- | | 1.6  Allow this person to EDIT the application, including any supporting documents/stipulations requested during the review process: | |  | | yes | |  | | |  |  | | --- | --- | | |  | | --- | |  | | |  | | | |  |  |  |  |  | | --- | --- | --- | --- | --- | | |  | | --- | | 1.7  Include this person on all correspondences regarding this application: (Note: This will include all committee correspondence, decision outcomes, renewal notices, and adverse event submissions.) | |  | | yes | |  | | |  |  | | --- | --- | | |  | | --- | |  | | |  | | | |  |  |  |  |  | | --- | --- | --- | --- | --- | | |  | | --- | | **Credentials: Required for PI, Co-Is and Faculty Advisors** | |  | |  | |  | | |  |  | | --- | --- | | |  | | --- | |  | | |  | | | |  |  |  |  |  |  |  |  |  |  |  |  |  |  |  |  |  |  | | --- | --- | --- | --- | --- | --- | --- | --- | --- | --- | --- | --- | --- | --- | --- | --- | --- | --- | | |  | | --- | | Upload or update your CV, resume, or biographical sketch. | |  | | |  |  |  |  |  |  |  |  |  |  |  |  |  | | --- | --- | --- | --- | --- | --- | --- | --- | --- | --- | --- | --- | --- | | | Name | Version | | --- | --- | |  |  |  |  |  |  |  |  |  |  | | --- | --- | --- | --- | --- | --- | --- | --- | --- | --- | | There are no items to display | | | | | | | | | | | | |  | | |  |  | | --- | --- | | |  | | --- | |  | | |  | | | |  |  |  |  |  | | --- | --- | --- | --- | --- | | |  | | --- | | **Conflict of Interest Detail:  Required for all roles except Administrative Staff** | |  | |  | |  | | |  |  | | --- | --- | | |  | | --- | |  | | |  | | | |  |  |  |  |  |  | | --- | --- | --- | --- | --- | --- | | |  | | --- | | C1  Do you, your spouse, domestic partner, or dependents have any outside interests or relationships to companies or entities related to this research that the IRB should consider? | | **Examples of outside interests include, but are not limited to the following:** | | - receiving compensation whose value could be affected by the study outcome- IN THE AGGREGATE, expecting to receive compensation from the sponsor of the research of $10,000 or greater in the next year- having a proprietary interest in the sponsor of the research or a product tested by this research including but not limited to, a patent, trademark, copyright, or licensing agreement, or the right to receive royalties from product commercialization- individually or collectively, having an ownership interest (equity or stock options) in the sponsor of the research or product being tested whose value cannot be readily determined through reference to public prices- individually or collectively, having an ownership interest (equity or stock options) in a company or product whose value could be affected by the study outcome- IN THE AGGREGATE, having an ownership interest (equity or stock options) in the sponsor of the research that exceeds $10,000 or 1% when the sponsor is a publicly traded entity- receiving significant payments of other sorts with an aggregate value of $10,000 or more (or payment of ANY amount to medical school or hospital employees) made directly by the sponsor of this research for unrestricted research or education, equipment, consultancy, or honorarium- holding a position of management or leadership in company or entity related to this research including, but not limited to, officer, director, or member of an advisory board.- providing consulting services or serve on a Speaker’s Bureau, either paid or unpaid, to the financial or non-financial sponsor of this study- when the sponsor is a publicly traded entity, having any ownership interest (equity or stock options) in the sponsor- expecting to receive any loans, educational support, contributions of in-kind for equipment, or any other non-compensatory payment from the sponsor of the research in the next year | | no | |  | | |  |  | | --- | --- | | |  | | --- | |  | | |  | | | |  |  |  |  |  | | --- | --- | --- | --- | --- | | |  | | --- | | C2  Please provide a detailed description of the outside interest in the box below. | |  | |  | |  | | |  |  | | --- | --- | | |  | | --- | |  | | |  | | | |  |  |  |  |  | | --- | --- | --- | --- | --- | | |  | | --- | | C2.1  Where have you submitted a disclosure of this outside interest? | |  | |  | |  | | |  |  | | --- | --- | | |  | | --- | |  | | |  | | | |  |  |  |  |  | | --- | --- | --- | --- | --- | | |  | | --- | | C2.2  Has a management plan been formalized? | |  | |  | |  | | |  |  | | --- | --- | | |  | | --- | |  | | |  | | | |  |  |  |  |  |  |  |  |  |  |  |  |  |  |  |  |  |  | | --- | --- | --- | --- | --- | --- | --- | --- | --- | --- | --- | --- | --- | --- | --- | --- | --- | --- | | |  | | --- | | C2.2.1  If yes, attach the management plan here, as well as any other applicable conflict of interest documents. | |  | | |  |  |  |  |  |  |  |  |  |  |  |  |  | | --- | --- | --- | --- | --- | --- | --- | --- | --- | --- | --- | --- | --- | | | Name | Version | | --- | --- | |  |  |  |  |  |  |  |  |  |  | | --- | --- | --- | --- | --- | --- | --- | --- | --- | --- | | There are no items to display | | | | | | | | | | | | |  | | |  |  | | --- | --- | | |  | | --- | |  | | |  | | | |  |  |  |  |  | | --- | --- | --- | --- | --- | | |  | | --- | | C2.2.2  If no, describe the financial interest in sufficient detail to permit the COI Ancillary Committee and the IRB to determine if such involvement represents a potential conflict-of-interest and/or should be disclosed to potential research subjects in the informed consent form. | |  | |  | |  | | |  |  | | --- | --- | | |  | | --- | |  | | |  | | |
| Study Team Detail     |  |  |  |  |  |  |  |  |  |  |  |  |  |  |  |  |  |  |  |  | | --- | --- | --- | --- | --- | --- | --- | --- | --- | --- | --- | --- | --- | --- | --- | --- | --- | --- | --- | --- | | |  |  |  |  |  |  |  |  |  |  |  |  |  |  |  | | --- | --- | --- | --- | --- | --- | --- | --- | --- | --- | --- | --- | --- | --- | --- | | |  | | --- | | 1.4  Team Member: | |  | | Judy Connelly     |  |  | | --- | --- | |  |  | | Preferred email: | jconnell@umich.edu | | Business phone | 734-998-7120 | | Business address: | Family Medicine | 1018 Fuller SPC 5708 | 48109-5708 | | |  | | |  |  | | --- | --- | | |  | | --- | |  | | |  | | | |  |  |  |  |  | | --- | --- | --- | --- | --- | | |  | | --- | | 1.5  Function with respect to project: | |  | | Administrative Staff | |  | | |  |  | | --- | --- | | |  | | --- | |  | | |  | | | |  |  |  |  |  | | --- | --- | --- | --- | --- | | |  | | --- | | 1.6  Allow this person to EDIT the application, including any supporting documents/stipulations requested during the review process: | |  | | yes | |  | | |  |  | | --- | --- | | |  | | --- | |  | | |  | | | |  |  |  |  |  | | --- | --- | --- | --- | --- | | |  | | --- | | 1.7  Include this person on all correspondences regarding this application: (Note: This will include all committee correspondence, decision outcomes, renewal notices, and adverse event submissions.) | |  | | yes | |  | | |  |  | | --- | --- | | |  | | --- | |  | | |  | | | |  |  |  |  |  | | --- | --- | --- | --- | --- | | |  | | --- | | **Credentials: Required for PI, Co-Is and Faculty Advisors** | |  | |  | |  | | |  |  | | --- | --- | | |  | | --- | |  | | |  | | | |  |  |  |  |  |  |  |  |  |  | | --- | --- | --- | --- | --- | --- | --- | --- | --- | --- | | |  | | --- | | Upload or update your CV, resume, or biographical sketch. | |  | | |  |  |  |  |  | | --- | --- | --- | --- | --- | | | Name | Version | | --- | --- | | Judy Connelly.docx | History | 0.01 | | | |  | | |  |  | | --- | --- | | |  | | --- | |  | | |  | | | |  |  |  |  |  | | --- | --- | --- | --- | --- | | |  | | --- | | **Conflict of Interest Detail:  Required for all roles except Administrative Staff** | |  | |  | |  | | |  |  | | --- | --- | | |  | | --- | |  | | |  | | | |  |  |  |  |  |  | | --- | --- | --- | --- | --- | --- | | |  | | --- | | C1  Do you, your spouse, domestic partner, or dependents have any outside interests or relationships to companies or entities related to this research that the IRB should consider? | | **Examples of outside interests include, but are not limited to the following:** | | - receiving compensation whose value could be affected by the study outcome- IN THE AGGREGATE, expecting to receive compensation from the sponsor of the research of $10,000 or greater in the next year- having a proprietary interest in the sponsor of the research or a product tested by this research including but not limited to, a patent, trademark, copyright, or licensing agreement, or the right to receive royalties from product commercialization- individually or collectively, having an ownership interest (equity or stock options) in the sponsor of the research or product being tested whose value cannot be readily determined through reference to public prices- individually or collectively, having an ownership interest (equity or stock options) in a company or product whose value could be affected by the study outcome- IN THE AGGREGATE, having an ownership interest (equity or stock options) in the sponsor of the research that exceeds $10,000 or 1% when the sponsor is a publicly traded entity- receiving significant payments of other sorts with an aggregate value of $10,000 or more (or payment of ANY amount to medical school or hospital employees) made directly by the sponsor of this research for unrestricted research or education, equipment, consultancy, or honorarium- holding a position of management or leadership in company or entity related to this research including, but not limited to, officer, director, or member of an advisory board.- providing consulting services or serve on a Speaker’s Bureau, either paid or unpaid, to the financial or non-financial sponsor of this study- when the sponsor is a publicly traded entity, having any ownership interest (equity or stock options) in the sponsor- expecting to receive any loans, educational support, contributions of in-kind for equipment, or any other non-compensatory payment from the sponsor of the research in the next year | |  | |  | | |  |  | | --- | --- | | |  | | --- | |  | | |  | | | |  |  |  |  |  | | --- | --- | --- | --- | --- | | |  | | --- | | C2  Please provide a detailed description of the outside interest in the box below. | |  | |  | |  | | |  |  | | --- | --- | | |  | | --- | |  | | |  | | | |  |  |  |  |  | | --- | --- | --- | --- | --- | | |  | | --- | | C2.1  Where have you submitted a disclosure of this outside interest? | |  | |  | |  | | |  |  | | --- | --- | | |  | | --- | |  | | |  | | | |  |  |  |  |  | | --- | --- | --- | --- | --- | | |  | | --- | | C2.2  Has a management plan been formalized? | |  | |  | |  | | |  |  | | --- | --- | | |  | | --- | |  | | |  | | | |  |  |  |  |  |  |  |  |  |  |  |  |  |  |  |  |  |  | | --- | --- | --- | --- | --- | --- | --- | --- | --- | --- | --- | --- | --- | --- | --- | --- | --- | --- | | |  | | --- | | C2.2.1  If yes, attach the management plan here, as well as any other applicable conflict of interest documents. | |  | | |  |  |  |  |  |  |  |  |  |  |  |  |  | | --- | --- | --- | --- | --- | --- | --- | --- | --- | --- | --- | --- | --- | | | Name | Version | | --- | --- | |  |  |  |  |  |  |  |  |  |  | | --- | --- | --- | --- | --- | --- | --- | --- | --- | --- | | There are no items to display | | | | | | | | | | | | |  | | |  |  | | --- | --- | | |  | | --- | |  | | |  | | | |  |  |  |  |  | | --- | --- | --- | --- | --- | | |  | | --- | | C2.2.2  If no, describe the financial interest in sufficient detail to permit the COI Ancillary Committee and the IRB to determine if such involvement represents a potential conflict-of-interest and/or should be disclosed to potential research subjects in the informed consent form. | |  | |  | |  | | |  |  | | --- | --- | | |  | | --- | |  | | |  | | |
| Study Team Detail     |  |  |  |  |  |  |  |  |  |  |  |  |  |  |  |  |  |  |  |  | | --- | --- | --- | --- | --- | --- | --- | --- | --- | --- | --- | --- | --- | --- | --- | --- | --- | --- | --- | --- | | |  |  |  |  |  |  |  |  |  |  |  |  |  |  |  | | --- | --- | --- | --- | --- | --- | --- | --- | --- | --- | --- | --- | --- | --- | --- | | |  | | --- | | 1.4  Team Member: | |  | | Katie Grode     |  |  | | --- | --- | |  |  | | Preferred email: | kgrode@umich.edu | | Business phone | 734-998-7120 | | Business address: | 1018 Fuller Street |  | 48109-1213 | | |  | | |  |  | | --- | --- | | |  | | --- | |  | | |  | | | |  |  |  |  |  | | --- | --- | --- | --- | --- | | |  | | --- | | 1.5  Function with respect to project: | |  | | Administrative Staff | |  | | |  |  | | --- | --- | | |  | | --- | |  | | |  | | | |  |  |  |  |  | | --- | --- | --- | --- | --- | | |  | | --- | | 1.6  Allow this person to EDIT the application, including any supporting documents/stipulations requested during the review process: | |  | | yes | |  | | |  |  | | --- | --- | | |  | | --- | |  | | |  | | | |  |  |  |  |  | | --- | --- | --- | --- | --- | | |  | | --- | | 1.7  Include this person on all correspondences regarding this application: (Note: This will include all committee correspondence, decision outcomes, renewal notices, and adverse event submissions.) | |  | | yes | |  | | |  |  | | --- | --- | | |  | | --- | |  | | |  | | | |  |  |  |  |  | | --- | --- | --- | --- | --- | | |  | | --- | | **Credentials: Required for PI, Co-Is and Faculty Advisors** | |  | |  | |  | | |  |  | | --- | --- | | |  | | --- | |  | | |  | | | |  |  |  |  |  |  |  |  |  |  |  |  |  |  |  |  |  |  | | --- | --- | --- | --- | --- | --- | --- | --- | --- | --- | --- | --- | --- | --- | --- | --- | --- | --- | | |  | | --- | | Upload or update your CV, resume, or biographical sketch. | |  | | |  |  |  |  |  |  |  |  |  |  |  |  |  | | --- | --- | --- | --- | --- | --- | --- | --- | --- | --- | --- | --- | --- | | | Name | Version | | --- | --- | |  |  |  |  |  |  |  |  |  |  | | --- | --- | --- | --- | --- | --- | --- | --- | --- | --- | | There are no items to display | | | | | | | | | | | | |  | | |  |  | | --- | --- | | |  | | --- | |  | | |  | | | |  |  |  |  |  | | --- | --- | --- | --- | --- | | |  | | --- | | **Conflict of Interest Detail:  Required for all roles except Administrative Staff** | |  | |  | |  | | |  |  | | --- | --- | | |  | | --- | |  | | |  | | | |  |  |  |  |  |  | | --- | --- | --- | --- | --- | --- | | |  | | --- | | C1  Do you, your spouse, domestic partner, or dependents have any outside interests or relationships to companies or entities related to this research that the IRB should consider? | | **Examples of outside interests include, but are not limited to the following:** | | - receiving compensation whose value could be affected by the study outcome- IN THE AGGREGATE, expecting to receive compensation from the sponsor of the research of $10,000 or greater in the next year- having a proprietary interest in the sponsor of the research or a product tested by this research including but not limited to, a patent, trademark, copyright, or licensing agreement, or the right to receive royalties from product commercialization- individually or collectively, having an ownership interest (equity or stock options) in the sponsor of the research or product being tested whose value cannot be readily determined through reference to public prices- individually or collectively, having an ownership interest (equity or stock options) in a company or product whose value could be affected by the study outcome- IN THE AGGREGATE, having an ownership interest (equity or stock options) in the sponsor of the research that exceeds $10,000 or 1% when the sponsor is a publicly traded entity- receiving significant payments of other sorts with an aggregate value of $10,000 or more (or payment of ANY amount to medical school or hospital employees) made directly by the sponsor of this research for unrestricted research or education, equipment, consultancy, or honorarium- holding a position of management or leadership in company or entity related to this research including, but not limited to, officer, director, or member of an advisory board.- providing consulting services or serve on a Speaker’s Bureau, either paid or unpaid, to the financial or non-financial sponsor of this study- when the sponsor is a publicly traded entity, having any ownership interest (equity or stock options) in the sponsor- expecting to receive any loans, educational support, contributions of in-kind for equipment, or any other non-compensatory payment from the sponsor of the research in the next year | |  | |  | | |  |  | | --- | --- | | |  | | --- | |  | | |  | | | |  |  |  |  |  | | --- | --- | --- | --- | --- | | |  | | --- | | C2  Please provide a detailed description of the outside interest in the box below. | |  | |  | |  | | |  |  | | --- | --- | | |  | | --- | |  | | |  | | | |  |  |  |  |  | | --- | --- | --- | --- | --- | | |  | | --- | | C2.1  Where have you submitted a disclosure of this outside interest? | |  | |  | |  | | |  |  | | --- | --- | | |  | | --- | |  | | |  | | | |  |  |  |  |  | | --- | --- | --- | --- | --- | | |  | | --- | | C2.2  Has a management plan been formalized? | |  | |  | |  | | |  |  | | --- | --- | | |  | | --- | |  | | |  | | | |  |  |  |  |  |  |  |  |  |  |  |  |  |  |  |  |  |  | | --- | --- | --- | --- | --- | --- | --- | --- | --- | --- | --- | --- | --- | --- | --- | --- | --- | --- | | |  | | --- | | C2.2.1  If yes, attach the management plan here, as well as any other applicable conflict of interest documents. | |  | | |  |  |  |  |  |  |  |  |  |  |  |  |  | | --- | --- | --- | --- | --- | --- | --- | --- | --- | --- | --- | --- | --- | | | Name | Version | | --- | --- | |  |  |  |  |  |  |  |  |  |  | | --- | --- | --- | --- | --- | --- | --- | --- | --- | --- | | There are no items to display | | | | | | | | | | | | |  | | |  |  | | --- | --- | | |  | | --- | |  | | |  | | | |  |  |  |  |  | | --- | --- | --- | --- | --- | | |  | | --- | | C2.2.2  If no, describe the financial interest in sufficient detail to permit the COI Ancillary Committee and the IRB to determine if such involvement represents a potential conflict-of-interest and/or should be disclosed to potential research subjects in the informed consent form. | |  | |  | |  | | |  |  | | --- | --- | | |  | | --- | |  | | |  | | |
| Study Team Detail     |  |  |  |  |  |  |  |  |  |  |  |  |  |  |  |  |  |  |  |  | | --- | --- | --- | --- | --- | --- | --- | --- | --- | --- | --- | --- | --- | --- | --- | --- | --- | --- | --- | --- | | |  |  |  |  |  |  |  |  |  |  |  |  |  |  |  | | --- | --- | --- | --- | --- | --- | --- | --- | --- | --- | --- | --- | --- | --- | --- | | |  | | --- | | 1.4  Team Member: | |  | | Lauren Schleicher     |  |  | | --- | --- | |  |  | | Preferred email: | lschlei@med.umich.edu | | Business phone | 734-998-7120 | | Business address: | Family Medicine | 1018 Fuller Street | 48109 | | |  | | |  |  | | --- | --- | | |  | | --- | |  | | |  | | | |  |  |  |  |  | | --- | --- | --- | --- | --- | | |  | | --- | | 1.5  Function with respect to project: | |  | | Administrative Staff | |  | | |  |  | | --- | --- | | |  | | --- | |  | | |  | | | |  |  |  |  |  | | --- | --- | --- | --- | --- | | |  | | --- | | 1.6  Allow this person to EDIT the application, including any supporting documents/stipulations requested during the review process: | |  | | yes | |  | | |  |  | | --- | --- | | |  | | --- | |  | | |  | | | |  |  |  |  |  | | --- | --- | --- | --- | --- | | |  | | --- | | 1.7  Include this person on all correspondences regarding this application: (Note: This will include all committee correspondence, decision outcomes, renewal notices, and adverse event submissions.) | |  | | no | |  | | |  |  | | --- | --- | | |  | | --- | |  | | |  | | | |  |  |  |  |  | | --- | --- | --- | --- | --- | | |  | | --- | | **Credentials: Required for PI, Co-Is and Faculty Advisors** | |  | |  | |  | | |  |  | | --- | --- | | |  | | --- | |  | | |  | | | |  |  |  |  |  |  |  |  |  |  |  |  |  |  |  |  |  |  | | --- | --- | --- | --- | --- | --- | --- | --- | --- | --- | --- | --- | --- | --- | --- | --- | --- | --- | | |  | | --- | | Upload or update your CV, resume, or biographical sketch. | |  | | |  |  |  |  |  |  |  |  |  |  |  |  |  | | --- | --- | --- | --- | --- | --- | --- | --- | --- | --- | --- | --- | --- | | | Name | Version | | --- | --- | |  |  |  |  |  |  |  |  |  |  | | --- | --- | --- | --- | --- | --- | --- | --- | --- | --- | | There are no items to display | | | | | | | | | | | | |  | | |  |  | | --- | --- | | |  | | --- | |  | | |  | | | |  |  |  |  |  | | --- | --- | --- | --- | --- | | |  | | --- | | **Conflict of Interest Detail:  Required for all roles except Administrative Staff** | |  | |  | |  | | |  |  | | --- | --- | | |  | | --- | |  | | |  | | | |  |  |  |  |  |  | | --- | --- | --- | --- | --- | --- | | |  | | --- | | C1  Do you, your spouse, domestic partner, or dependents have any outside interests or relationships to companies or entities related to this research that the IRB should consider? | | **Examples of outside interests include, but are not limited to the following:** | | - receiving compensation whose value could be affected by the study outcome- IN THE AGGREGATE, expecting to receive compensation from the sponsor of the research of $10,000 or greater in the next year- having a proprietary interest in the sponsor of the research or a product tested by this research including but not limited to, a patent, trademark, copyright, or licensing agreement, or the right to receive royalties from product commercialization- individually or collectively, having an ownership interest (equity or stock options) in the sponsor of the research or product being tested whose value cannot be readily determined through reference to public prices- individually or collectively, having an ownership interest (equity or stock options) in a company or product whose value could be affected by the study outcome- IN THE AGGREGATE, having an ownership interest (equity or stock options) in the sponsor of the research that exceeds $10,000 or 1% when the sponsor is a publicly traded entity- receiving significant payments of other sorts with an aggregate value of $10,000 or more (or payment of ANY amount to medical school or hospital employees) made directly by the sponsor of this research for unrestricted research or education, equipment, consultancy, or honorarium- holding a position of management or leadership in company or entity related to this research including, but not limited to, officer, director, or member of an advisory board.- providing consulting services or serve on a Speaker’s Bureau, either paid or unpaid, to the financial or non-financial sponsor of this study- when the sponsor is a publicly traded entity, having any ownership interest (equity or stock options) in the sponsor- expecting to receive any loans, educational support, contributions of in-kind for equipment, or any other non-compensatory payment from the sponsor of the research in the next year | |  | |  | | |  |  | | --- | --- | | |  | | --- | |  | | |  | | | |  |  |  |  |  | | --- | --- | --- | --- | --- | | |  | | --- | | C2  Please provide a detailed description of the outside interest in the box below. | |  | |  | |  | | |  |  | | --- | --- | | |  | | --- | |  | | |  | | | |  |  |  |  |  | | --- | --- | --- | --- | --- | | |  | | --- | | C2.1  Where have you submitted a disclosure of this outside interest? | |  | |  | |  | | |  |  | | --- | --- | | |  | | --- | |  | | |  | | | |  |  |  |  |  | | --- | --- | --- | --- | --- | | |  | | --- | | C2.2  Has a management plan been formalized? | |  | |  | |  | | |  |  | | --- | --- | | |  | | --- | |  | | |  | | | |  |  |  |  |  |  |  |  |  |  |  |  |  |  |  |  |  |  | | --- | --- | --- | --- | --- | --- | --- | --- | --- | --- | --- | --- | --- | --- | --- | --- | --- | --- | | |  | | --- | | C2.2.1  If yes, attach the management plan here, as well as any other applicable conflict of interest documents. | |  | | |  |  |  |  |  |  |  |  |  |  |  |  |  | | --- | --- | --- | --- | --- | --- | --- | --- | --- | --- | --- | --- | --- | | | Name | Version | | --- | --- | |  |  |  |  |  |  |  |  |  |  | | --- | --- | --- | --- | --- | --- | --- | --- | --- | --- | | There are no items to display | | | | | | | | | | | | |  | | |  |  | | --- | --- | | |  | | --- | |  | | |  | | | |  |  |  |  |  | | --- | --- | --- | --- | --- | | |  | | --- | | C2.2.2  If no, describe the financial interest in sufficient detail to permit the COI Ancillary Committee and the IRB to determine if such involvement represents a potential conflict-of-interest and/or should be disclosed to potential research subjects in the informed consent form. | |  | |  | |  | | |  |  | | --- | --- | | |  | | --- | |  | | |  | | |
| Study Team Detail     |  |  |  |  |  |  |  |  |  |  |  |  |  |  |  |  |  |  |  |  | | --- | --- | --- | --- | --- | --- | --- | --- | --- | --- | --- | --- | --- | --- | --- | --- | --- | --- | --- | --- | | |  |  |  |  |  |  |  |  |  |  |  |  |  |  |  | | --- | --- | --- | --- | --- | --- | --- | --- | --- | --- | --- | --- | --- | --- | --- | | |  | | --- | | 1.4  Team Member: | |  | | Krystle Woods     |  |  | | --- | --- | |  |  | | Preferred email: | krystlew@med.umich.edu | | Business phone | 734-998-7120 | | Business address: | Family Medicine | 1018 Fuller Street | 48109 | | |  | | |  |  | | --- | --- | | |  | | --- | |  | | |  | | | |  |  |  |  |  | | --- | --- | --- | --- | --- | | |  | | --- | | 1.5  Function with respect to project: | |  | | Administrative Staff | |  | | |  |  | | --- | --- | | |  | | --- | |  | | |  | | | |  |  |  |  |  | | --- | --- | --- | --- | --- | | |  | | --- | | 1.6  Allow this person to EDIT the application, including any supporting documents/stipulations requested during the review process: | |  | | yes | |  | | |  |  | | --- | --- | | |  | | --- | |  | | |  | | | |  |  |  |  |  | | --- | --- | --- | --- | --- | | |  | | --- | | 1.7  Include this person on all correspondences regarding this application: (Note: This will include all committee correspondence, decision outcomes, renewal notices, and adverse event submissions.) | |  | | no | |  | | |  |  | | --- | --- | | |  | | --- | |  | | |  | | | |  |  |  |  |  | | --- | --- | --- | --- | --- | | |  | | --- | | **Credentials: Required for PI, Co-Is and Faculty Advisors** | |  | |  | |  | | |  |  | | --- | --- | | |  | | --- | |  | | |  | | | |  |  |  |  |  |  |  |  |  |  |  |  |  |  |  |  |  |  | | --- | --- | --- | --- | --- | --- | --- | --- | --- | --- | --- | --- | --- | --- | --- | --- | --- | --- | | |  | | --- | | Upload or update your CV, resume, or biographical sketch. | |  | | |  |  |  |  |  |  |  |  |  |  |  |  |  | | --- | --- | --- | --- | --- | --- | --- | --- | --- | --- | --- | --- | --- | | | Name | Version | | --- | --- | |  |  |  |  |  |  |  |  |  |  | | --- | --- | --- | --- | --- | --- | --- | --- | --- | --- | | There are no items to display | | | | | | | | | | | | |  | | |  |  | | --- | --- | | |  | | --- | |  | | |  | | | |  |  |  |  |  | | --- | --- | --- | --- | --- | | |  | | --- | | **Conflict of Interest Detail:  Required for all roles except Administrative Staff** | |  | |  | |  | | |  |  | | --- | --- | | |  | | --- | |  | | |  | | | |  |  |  |  |  |  | | --- | --- | --- | --- | --- | --- | | |  | | --- | | C1  Do you, your spouse, domestic partner, or dependents have any outside interests or relationships to companies or entities related to this research that the IRB should consider? | | **Examples of outside interests include, but are not limited to the following:** | | - receiving compensation whose value could be affected by the study outcome- IN THE AGGREGATE, expecting to receive compensation from the sponsor of the research of $10,000 or greater in the next year- having a proprietary interest in the sponsor of the research or a product tested by this research including but not limited to, a patent, trademark, copyright, or licensing agreement, or the right to receive royalties from product commercialization- individually or collectively, having an ownership interest (equity or stock options) in the sponsor of the research or product being tested whose value cannot be readily determined through reference to public prices- individually or collectively, having an ownership interest (equity or stock options) in a company or product whose value could be affected by the study outcome- IN THE AGGREGATE, having an ownership interest (equity or stock options) in the sponsor of the research that exceeds $10,000 or 1% when the sponsor is a publicly traded entity- receiving significant payments of other sorts with an aggregate value of $10,000 or more (or payment of ANY amount to medical school or hospital employees) made directly by the sponsor of this research for unrestricted research or education, equipment, consultancy, or honorarium- holding a position of management or leadership in company or entity related to this research including, but not limited to, officer, director, or member of an advisory board.- providing consulting services or serve on a Speaker’s Bureau, either paid or unpaid, to the financial or non-financial sponsor of this study- when the sponsor is a publicly traded entity, having any ownership interest (equity or stock options) in the sponsor- expecting to receive any loans, educational support, contributions of in-kind for equipment, or any other non-compensatory payment from the sponsor of the research in the next year | |  | |  | | |  |  | | --- | --- | | |  | | --- | |  | | |  | | | |  |  |  |  |  | | --- | --- | --- | --- | --- | | |  | | --- | | C2  Please provide a detailed description of the outside interest in the box below. | |  | |  | |  | | |  |  | | --- | --- | | |  | | --- | |  | | |  | | | |  |  |  |  |  | | --- | --- | --- | --- | --- | | |  | | --- | | C2.1  Where have you submitted a disclosure of this outside interest? | |  | |  | |  | | |  |  | | --- | --- | | |  | | --- | |  | | |  | | | |  |  |  |  |  | | --- | --- | --- | --- | --- | | |  | | --- | | C2.2  Has a management plan been formalized? | |  | |  | |  | | |  |  | | --- | --- | | |  | | --- | |  | | |  | | | |  |  |  |  |  |  |  |  |  |  |  |  |  |  |  |  |  |  | | --- | --- | --- | --- | --- | --- | --- | --- | --- | --- | --- | --- | --- | --- | --- | --- | --- | --- | | |  | | --- | | C2.2.1  If yes, attach the management plan here, as well as any other applicable conflict of interest documents. | |  | | |  |  |  |  |  |  |  |  |  |  |  |  |  | | --- | --- | --- | --- | --- | --- | --- | --- | --- | --- | --- | --- | --- | | | Name | Version | | --- | --- | |  |  |  |  |  |  |  |  |  |  | | --- | --- | --- | --- | --- | --- | --- | --- | --- | --- | | There are no items to display | | | | | | | | | | | | |  | | |  |  | | --- | --- | | |  | | --- | |  | | |  | | | |  |  |  |  |  | | --- | --- | --- | --- | --- | | |  | | --- | | C2.2.2  If no, describe the financial interest in sufficient detail to permit the COI Ancillary Committee and the IRB to determine if such involvement represents a potential conflict-of-interest and/or should be disclosed to potential research subjects in the informed consent form. | |  | |  | |  | | |  |  | | --- | --- | | |  | | --- | |  | | |  | | |

|  |  |  |
| --- | --- | --- |
| |  |  | | --- | --- | |  |  | |
| 02. Sponsor/Support Information  **The following sections request details about the current or pending sponsorship/support of this study. Consider all of the choices below and complete the appropriate sections.  \* Note: At least one of the following sections must be answered. Multiple sponsors or sources of support must be added one at a time.**     |  |  |  |  |  |  |  |  |  |  |  |  |  |  |  |  |  |  |  |  |  |  |  |  |  |  |  |  |  | | --- | --- | --- | --- | --- | --- | --- | --- | --- | --- | --- | --- | --- | --- | --- | --- | --- | --- | --- | --- | --- | --- | --- | --- | --- | --- | --- | --- | --- | | |  |  |  |  |  |  |  |  |  |  |  |  |  |  |  |  |  |  |  |  |  |  |  |  | | --- | --- | --- | --- | --- | --- | --- | --- | --- | --- | --- | --- | --- | --- | --- | --- | --- | --- | --- | --- | --- | --- | --- | --- | | |  | | --- | | 2.1  External Sponsor(s)/Support: | |  | | |  |  |  |  |  |  |  |  |  |  |  |  |  |  |  |  |  |  |  | | --- | --- | --- | --- | --- | --- | --- | --- | --- | --- | --- | --- | --- | --- | --- | --- | --- | --- | --- | | |  | Type | Name | Other Direct Sponsor/Support | Support Type | Has PAF? | | --- | --- | --- | --- | --- | --- | | View | Government - Federal without Stimulus Plan (American Recovery and Reinvestment Act) funding |  | National Cancer Institute | Financial | yes | | View | Government - Federal without Stimulus Plan (American Recovery and Reinvestment Act) funding |  | NIH-National Cancer Institute | Financial | yes | | | |  | | |  |  | | --- | --- | | |  | | --- | |  | | |  | | | |  |  |  |  |  |  |  |  |  |  |  |  |  |  |  |  |  |  |  |  |  | | --- | --- | --- | --- | --- | --- | --- | --- | --- | --- | --- | --- | --- | --- | --- | --- | --- | --- | --- | --- | --- | | |  | | --- | | 2.5  Internal UM Sponsor(s)/Support: [Including department or PI discretionary funding] | |  | | |  |  |  |  |  |  |  |  |  |  |  |  |  |  |  |  | | --- | --- | --- | --- | --- | --- | --- | --- | --- | --- | --- | --- | --- | --- | --- | --- | | |  | Type | Department Sponsor | Support Type | | --- | --- | --- | --- | |  |  |  |  |  |  |  |  |  |  |  | | --- | --- | --- | --- | --- | --- | --- | --- | --- | --- | --- | |  | There are no items to display | | | | | | | | | | | | |  | | |  |  | | --- | --- | | |  | | --- | |  | | |  | | | |  |  |  |  |  | | --- | --- | --- | --- | --- | | |  | | --- | | 2.8  Check here if the proposed study does not require external or internal sponsorship or support: | |  | |  | |  | | |  |  | | --- | --- | | |  | | --- | |  | | |  | | |
| External Sponsor Detail     |  |  |  |  |  |  |  |  |  |  | | --- | --- | --- | --- | --- | --- | --- | --- | --- | --- | | |  |  |  |  |  | | --- | --- | --- | --- | --- | | |  | | --- | | 2.2\*  Direct Sponsor/Support: | |  | | **If the Direct Sponsor/Support does not appear in the Select list, enter the name of the Direct Sponsor/Support below:**  National Cancer Institute | |  | | |  |  | | --- | --- | | |  | | --- | |  | | |  | | | |  |  |  |  |  | | --- | --- | --- | --- | --- | | |  | | --- | | 2.2.1\*  Sponsor Type: | |  | | Government - Federal without Stimulus Plan (American Recovery and Reinvestment Act) funding   **If other, please specify:** | |  | | |  |  | | --- | --- | | |  | | --- | |  | | |  | | | |  |  |  |  |  | | --- | --- | --- | --- | --- | | |  | | --- | | 2.2.2\*  Support Type: | |  | | Financial | |  | | |  |  | | --- | --- | | |  | | --- | |  | | |  | | | |  |  |  |  |  | | --- | --- | --- | --- | --- | | |  | | --- | | 2.2.3\*  Is the support confirmed? | |  | | **Yes** No | |  | | |  |  | | --- | --- | | |  | | --- | |  | | |  | | | |  |  |  |  |  | | --- | --- | --- | --- | --- | | |  | | --- | | 2.2.4\*  Is there an existing Proposal Approval Form (PAF) for this IRB Application | |  | | **Yes** No | |  | | |  |  | | --- | --- | | |  | | --- | |  | | |  | |      |  |  |  |  |  |  |  |  |  |  |  |  |  | | --- | --- | --- | --- | --- | --- | --- | --- | --- | --- | --- | --- | --- | | |  |  |  |  |  |  |  |  | | --- | --- | --- | --- | --- | --- | --- | --- | | |  | | --- | | 2.2.5\*  Please select the PAF(s) associated with this study. Clicking the Add button will allow for the selection of a PAF based on selected criteria. After the PAF(s) has been associated with the human subjects research application, clicking on the PAF link will access the Proposal Management system and will display the current PAF information. Access to the PAF is based on account information in the Proposal Management system. | |  | | |  |  |  | | --- | --- | --- | | | Proposal ID | | --- | | 10-PAF07295 | | | |  | | |  |  | | --- | --- | | |  | | --- | |  | | |  | |       |  |  |  |  |  |  |  |  |  |  | | --- | --- | --- | --- | --- | --- | --- | --- | --- | --- | | |  |  |  |  |  | | --- | --- | --- | --- | --- | | |  | | --- | | 2.2.6  Previously entered DRDA #: | |  | |  | |  | | |  |  | | --- | --- | | |  | | --- | |  | | |  | |     |  |  |  |  |  |  |  |  |  |  | | --- | --- | --- | --- | --- | --- | --- | --- | --- | --- | | |  |  |  |  |  | | --- | --- | --- | --- | --- | | |  | | --- | | 2.3\*  Is this a subcontract to UM? | |  | | Yes **No** | |  | | |  |  | | --- | --- | | |  | | --- | |  | | |  | |      |  |  |  |  |  |  |  |  |  |  | | --- | --- | --- | --- | --- | --- | --- | --- | --- | --- | | |  |  |  |  |  | | --- | --- | --- | --- | --- | | |  | | --- | | 2.3.1\*  Indicate the Prime Sponsor/Support (the original source of funding): | |  | | **If the Prime Sponsor/Support does not appear in the Select list, enter the name of the Prime Sponsor below:** | |  | | |  |  | | --- | --- | | |  | | --- | |  | | |  | | | |  |  |  |  |  | | --- | --- | --- | --- | --- | | |  | | --- | | 2.3.2\*  Prime Sponsor Type: | |  | |  | |  | | |  |  | | --- | --- | | |  | | --- | |  | | |  | | | |  |  |  |  |  | | --- | --- | --- | --- | --- | | |  | | --- | | 2.3.3\*  Prime Support Type: | |  | |  | |  | | |  |  | | --- | --- | | |  | | --- | |  | | |  | | | |  |  |  |  |  | | --- | --- | --- | --- | --- | | |  | | --- | | 2.3.4\*  Is the Prime Support confirmed? | |  | | Yes No | |  | | |  |  | | --- | --- | | |  | | --- | |  | | |  | |       |  |  |  |  |  |  |  |  |  |  |  |  |  |  |  |  |  |  |  |  |  |  |  | | --- | --- | --- | --- | --- | --- | --- | --- | --- | --- | --- | --- | --- | --- | --- | --- | --- | --- | --- | --- | --- | --- | --- | | |  |  |  |  |  |  |  |  |  |  |  |  |  |  |  |  |  |  | | --- | --- | --- | --- | --- | --- | --- | --- | --- | --- | --- | --- | --- | --- | --- | --- | --- | --- | | |  | | --- | | 2.4  Upload all of the following documents that apply:  - Grant (\*required for "Government - Federal" sponsor types)- Contract application (\*required for "Government - Federal" sponsor types)- Sponsor budget (\*required for use of MCRU resources)- Sponsor application- Most recent competing renewal application | |  | | |  |  |  |  |  |  |  |  |  |  |  |  |  | | --- | --- | --- | --- | --- | --- | --- | --- | --- | --- | --- | --- | --- | | | Name | Version | | --- | --- | |  |  |  |  |  |  |  |  |  |  | | --- | --- | --- | --- | --- | --- | --- | --- | --- | --- | | There are no items to display | | | | | | | | | | |   **Note:** Study Teams are encouraged to scan and upload documents. See Help for a list of sites with scanning facilities. | |  | | |  |  | | --- | --- | | |  | | --- | |  | | |  | | |
| External Sponsor Detail     |  |  |  |  |  |  |  |  |  |  | | --- | --- | --- | --- | --- | --- | --- | --- | --- | --- | | |  |  |  |  |  | | --- | --- | --- | --- | --- | | |  | | --- | | 2.2\*  Direct Sponsor/Support: | |  | | **If the Direct Sponsor/Support does not appear in the Select list, enter the name of the Direct Sponsor/Support below:**  NIH-National Cancer Institute | |  | | |  |  | | --- | --- | | |  | | --- | |  | | |  | | | |  |  |  |  |  | | --- | --- | --- | --- | --- | | |  | | --- | | 2.2.1\*  Sponsor Type: | |  | | Government - Federal without Stimulus Plan (American Recovery and Reinvestment Act) funding   **If other, please specify:** | |  | | |  |  | | --- | --- | | |  | | --- | |  | | |  | | | |  |  |  |  |  | | --- | --- | --- | --- | --- | | |  | | --- | | 2.2.2\*  Support Type: | |  | | Financial | |  | | |  |  | | --- | --- | | |  | | --- | |  | | |  | | | |  |  |  |  |  | | --- | --- | --- | --- | --- | | |  | | --- | | 2.2.3\*  Is the support confirmed? | |  | | **Yes** No | |  | | |  |  | | --- | --- | | |  | | --- | |  | | |  | | | |  |  |  |  |  | | --- | --- | --- | --- | --- | | |  | | --- | | 2.2.4\*  Is there an existing Proposal Approval Form (PAF) for this IRB Application | |  | | **Yes** No | |  | | |  |  | | --- | --- | | |  | | --- | |  | | |  | |      |  |  |  |  |  |  |  |  |  |  |  |  |  | | --- | --- | --- | --- | --- | --- | --- | --- | --- | --- | --- | --- | --- | | |  |  |  |  |  |  |  |  | | --- | --- | --- | --- | --- | --- | --- | --- | | |  | | --- | | 2.2.5\*  Please select the PAF(s) associated with this study. Clicking the Add button will allow for the selection of a PAF based on selected criteria. After the PAF(s) has been associated with the human subjects research application, clicking on the PAF link will access the Proposal Management system and will display the current PAF information. Access to the PAF is based on account information in the Proposal Management system. | |  | | |  |  |  | | --- | --- | --- | | | Proposal ID | | --- | | 12-PAF04903 | | | |  | | |  |  | | --- | --- | | |  | | --- | |  | | |  | |       |  |  |  |  |  |  |  |  |  |  | | --- | --- | --- | --- | --- | --- | --- | --- | --- | --- | | |  |  |  |  |  | | --- | --- | --- | --- | --- | | |  | | --- | | 2.2.6  Previously entered DRDA #: | |  | |  | |  | | |  |  | | --- | --- | | |  | | --- | |  | | |  | |     |  |  |  |  |  |  |  |  |  |  | | --- | --- | --- | --- | --- | --- | --- | --- | --- | --- | | |  |  |  |  |  | | --- | --- | --- | --- | --- | | |  | | --- | | 2.3\*  Is this a subcontract to UM? | |  | | Yes **No** | |  | | |  |  | | --- | --- | | |  | | --- | |  | | |  | |      |  |  |  |  |  |  |  |  |  |  | | --- | --- | --- | --- | --- | --- | --- | --- | --- | --- | | |  |  |  |  |  | | --- | --- | --- | --- | --- | | |  | | --- | | 2.3.1\*  Indicate the Prime Sponsor/Support (the original source of funding): | |  | | **If the Prime Sponsor/Support does not appear in the Select list, enter the name of the Prime Sponsor below:** | |  | | |  |  | | --- | --- | | |  | | --- | |  | | |  | | | |  |  |  |  |  | | --- | --- | --- | --- | --- | | |  | | --- | | 2.3.2\*  Prime Sponsor Type: | |  | |  | |  | | |  |  | | --- | --- | | |  | | --- | |  | | |  | | | |  |  |  |  |  | | --- | --- | --- | --- | --- | | |  | | --- | | 2.3.3\*  Prime Support Type: | |  | |  | |  | | |  |  | | --- | --- | | |  | | --- | |  | | |  | | | |  |  |  |  |  | | --- | --- | --- | --- | --- | | |  | | --- | | 2.3.4\*  Is the Prime Support confirmed? | |  | | Yes No | |  | | |  |  | | --- | --- | | |  | | --- | |  | | |  | |       |  |  |  |  |  |  |  |  |  |  |  |  |  |  |  |  |  |  |  |  |  |  |  | | --- | --- | --- | --- | --- | --- | --- | --- | --- | --- | --- | --- | --- | --- | --- | --- | --- | --- | --- | --- | --- | --- | --- | | |  |  |  |  |  |  |  |  |  |  |  |  |  |  |  |  |  |  | | --- | --- | --- | --- | --- | --- | --- | --- | --- | --- | --- | --- | --- | --- | --- | --- | --- | --- | | |  | | --- | | 2.4  Upload all of the following documents that apply:  - Grant (\*required for "Government - Federal" sponsor types)- Contract application (\*required for "Government - Federal" sponsor types)- Sponsor budget (\*required for use of MCRU resources)- Sponsor application- Most recent competing renewal application | |  | | |  |  |  |  |  |  |  |  |  |  |  |  |  | | --- | --- | --- | --- | --- | --- | --- | --- | --- | --- | --- | --- | --- | | | Name | Version | | --- | --- | |  |  |  |  |  |  |  |  |  |  | | --- | --- | --- | --- | --- | --- | --- | --- | --- | --- | | There are no items to display | | | | | | | | | | |   **Note:** Study Teams are encouraged to scan and upload documents. See Help for a list of sites with scanning facilities. | |  | | |  |  | | --- | --- | | |  | | --- | |  | | |  | | |

|  |  |  |
| --- | --- | --- |
| |  |  | | --- | --- | |  |  | |
| 03. UM Study Functions     |  |  |  |  |  |  |  |  |  |  |  |  |  |  |  |  |  |  |  |  | | --- | --- | --- | --- | --- | --- | --- | --- | --- | --- | --- | --- | --- | --- | --- | --- | --- | --- | --- | --- | | |  |  |  |  |  |  |  |  |  |  |  |  |  |  |  | | --- | --- | --- | --- | --- | --- | --- | --- | --- | --- | --- | --- | --- | --- | --- | | |  | | --- | | 3.1\*  Indicate all functions that will be performed at University of Michigan locations. | |  | | |  |  |  |  |  |  |  |  |  |  | | --- | --- | --- | --- | --- | --- | --- | --- | --- | --- | | | Select all that apply: | | --- | | Recruitment (including screening) | | Interaction (e.g., information gathering, survey, interview, focus groups, etc.) | | Intervention (e.g., use of drug or device, medical procedures, educational intervention, group intervention, social/psychological intervention etc.) | | Observation of behavior (direct or indirect) | | Qualitative research (e.g., 'member checking', open-ended questions, etc.) | | Secondary data collection (e.g., medical chart review, data abstraction from existing records, etc.) | | Primary or secondary analysis (data/specimen) | | Storage (data/specimen) | |  If other, please specify. | |  | | |  |  | | --- | --- | | |  | | --- | |  | | |  | |      |  |  |  |  |  |  |  |  |  |  | | --- | --- | --- | --- | --- | --- | --- | --- | --- | --- | | |  |  |  |  |  | | --- | --- | --- | --- | --- | | |  | | --- | | 3.1.1\*  As the Operations, Coordinating or Lead Center describe the mechanisms in place to ensure that management, data analysis, and Data Safety and Monitoring systems are adequate for each site. | |  | |  | |  | | |  |  | | --- | --- | | |  | | --- | |  | | |  | | | |  |  |  |  |  | | --- | --- | --- | --- | --- | | |  | | --- | | 3.1.2\*  Describe the plan for communicating interim results (e.g. adverse events, unanticipated events or interim data): | |  | |  | |  | | |  |  | | --- | --- | | |  | | --- | |  | | |  | | | |  |  |  |  |  | | --- | --- | --- | --- | --- | | |  | | --- | | 3.1.3\*  Describe the plan for communicating any protocol modification by the site(s): | |  | |  | |  | | |  |  | | --- | --- | | |  | | --- | |  | | |  | | |
| 03-1. Performance Sites     |  |  |  |  |  |  |  |  |  |  |  |  |  |  |  |  |  |  |  |  |  |  |  |  |  |  |  |  |  |  |  |  |  |  |  |  |  |  |  |  |  |  |  |  |  |  |  |  |  |  |  |  |  |  |  |  |  |  |  |  |  |  |  |  |  |  |  |  |  |  |  |  |  |  |  | | --- | --- | --- | --- | --- | --- | --- | --- | --- | --- | --- | --- | --- | --- | --- | --- | --- | --- | --- | --- | --- | --- | --- | --- | --- | --- | --- | --- | --- | --- | --- | --- | --- | --- | --- | --- | --- | --- | --- | --- | --- | --- | --- | --- | --- | --- | --- | --- | --- | --- | --- | --- | --- | --- | --- | --- | --- | --- | --- | --- | --- | --- | --- | --- | --- | --- | --- | --- | --- | --- | --- | --- | --- | --- | --- | | |  |  |  |  |  |  |  |  |  |  |  |  |  |  |  |  |  |  |  |  |  |  |  |  |  |  |  |  |  |  |  |  |  |  |  |  |  |  |  |  |  |  |  |  |  |  |  |  |  |  |  |  |  |  |  |  |  |  |  |  |  |  |  |  |  |  |  |  |  |  | | --- | --- | --- | --- | --- | --- | --- | --- | --- | --- | --- | --- | --- | --- | --- | --- | --- | --- | --- | --- | --- | --- | --- | --- | --- | --- | --- | --- | --- | --- | --- | --- | --- | --- | --- | --- | --- | --- | --- | --- | --- | --- | --- | --- | --- | --- | --- | --- | --- | --- | --- | --- | --- | --- | --- | --- | --- | --- | --- | --- | --- | --- | --- | --- | --- | --- | --- | --- | --- | --- | | |  | | --- | | 3-1.1\*  Performance Sites: | |  | | |  |  |  |  |  |  |  |  |  |  |  |  |  |  |  |  |  |  |  |  |  |  |  |  |  |  |  |  |  |  |  |  |  |  |  |  |  |  |  |  |  |  |  |  |  |  |  |  |  |  |  |  |  |  |  |  |  |  |  |  |  |  |  |  |  | | --- | --- | --- | --- | --- | --- | --- | --- | --- | --- | --- | --- | --- | --- | --- | --- | --- | --- | --- | --- | --- | --- | --- | --- | --- | --- | --- | --- | --- | --- | --- | --- | --- | --- | --- | --- | --- | --- | --- | --- | --- | --- | --- | --- | --- | --- | --- | --- | --- | --- | --- | --- | --- | --- | --- | --- | --- | --- | --- | --- | --- | --- | --- | --- | --- | | | Location | Country | "Engaged" in the research? | Site Function | | --- | --- | --- | --- | | Andrew Thomas, MD PC | USA | no | Other,Secondary data collection,Recruitment | | Children & Family Medical Clinic | USA | no | Other,Secondary data collection,Recruitment | | David Williams, MD PC | USA | no | Other,Secondary data collection,Recruitment | | Downriver Internists PC | USA | no | Other,Secondary data collection,Recruitment | | Dua Family Practice | USA | no | Secondary data collection,Recruitment | | George C. Hawrot, MD | USA | no | Other,Secondary data collection,Recruitment | | IPC-Livonia Internal Medicine | USA | no | Other,Secondary data collection,Recruitment | | Lonnie Joe Jr., MD | USA | no | Other,Secondary data collection,Recruitment | | Mark W. Sawka, MD | USA | no | Other,Secondary data collection,Recruitment | | Michigan State university | USA | yes | Qualitative research,Intervention,Storage,Interaction,Analysis,Recruitment | | Morang Chester Clinic PC | USA | no | Other,Secondary data collection,Recruitment | | Rice Lanzilote Dos and Egan MD | USA | no | Other,Secondary data collection,Recruitment | | University of Michigan | USA | yes | Qualitative research,Intervention,Storage,Other,Interaction,Analysis,Secondary data collection,Observation,Recruitment | | University of Michigan Family Medicine Clinics | USA | yes | Intervention,Interaction,Secondary data collection,Recruitment | | up to 10 yet to be recruited practices in the Metro Detroit area | USA | no | Other,Secondary data collection,Recruitment | | | |  | | |  |  | | --- | --- | | |  | | --- | | Based on the information provided in 3.1, "University of Michigan" is listed below as a performance site. Click ADD to list non-UM locations (both domestic and foreign) where UM-related research activities are conducted, including:  - Subject recruitment- Interaction- Intervention- Study coordination- Data/specimen collection- Data/specimen analysis (e.g., radiology, lab, etc.)- Transcription services- Translation services- Other contracted services       Include all locations to which identifiable private information may be sent. This section may or may not apply to certain industry/corporate sponsor locations -- see Help for important instructions.    Research conducted in other states and in other countries may be subject to different requirements -- see Help for important instructions and consult with the Office of the General Counsel or the Health System Legal Office for consideration of human research regulations outside of Michigan. | | |  | | |
| Performance Site Detail     |  |  |  |  |  |  |  |  |  |  | | --- | --- | --- | --- | --- | --- | --- | --- | --- | --- | | |  |  |  |  |  | | --- | --- | --- | --- | --- | | |  | | --- | | 3-1.2\*  Location or Institution: | |  | | Andrew Thomas, MD PC | |  | | |  |  | | --- | --- | | |  | | --- | |  | | |  | | | |  |  |  |  |  |  |  |  |  |  |  | | --- | --- | --- | --- | --- | --- | --- | --- | --- | --- | --- | | |  | | --- | | 3-1.3  Address: | |  | | |  |  | | --- | --- | | City | Detroit | | State | MI | | Country**\*** | USA | | |  | | |  |  | | --- | --- | | |  | | --- | |  | | |  | | | |  |  |  |  |  |  |  |  |  |  | | --- | --- | --- | --- | --- | --- | --- | --- | --- | --- | | |  | | --- | | 3-1.4\*  Function of this location with respect to this study: | |  | | |  |  |  |  |  | | --- | --- | --- | --- | --- | | | Select all that apply: | | --- | | Recruitment (including screening) | | Secondary data collection (e.g., medical chart review, data abstraction from existing records, etc.) | | Other | |    **If other, please specify:**  Individual practices will identify patients and their practice physicians will review and exclude anyone who should not be contacted/invited to participate in the study | |  | | |  |  | | --- | --- | | |  | | --- | |  | | |  | | | |  |  |  |  |  | | --- | --- | --- | --- | --- | | |  | | --- | | 3-1.5\*  Will this site be "engaged" in the conduct of the research? | |  | | Yes **No** | |  | | |  |  | | --- | --- | | |  | | --- | |  | | |  | | | |  |  |  |  |  | | --- | --- | --- | --- | --- | | |  | | --- | | 3-1.6  If known, provide the Federalwide Assurance (FWA) number for this location. | |  | |  | |  | | |  |  | | --- | --- | | |  | | --- | |  | | |  | | | |  |  |  |  |  | | --- | --- | --- | --- | --- | | |  | | --- | | 3-1.7  If applicable, indicate what organization, agency or government office has reviewed this research and provided its approval (e.g., IRB, ethics committee, school district office, prison official, nursing home administrator). | |  | |  | |  | | |  |  | | --- | --- | | |  | | --- | |  | | |  | | | |  |  |  |  |  |  |  |  |  |  |  |  | | --- | --- | --- | --- | --- | --- | --- | --- | --- | --- | --- | --- | | |  | | --- | | 3-1.8  Upload any location site approval documentation here: | |  | | |  |  |  |  |  |  |  | | --- | --- | --- | --- | --- | --- | --- | | | Name | Version | | --- | --- | | Thomas\_DSA | History | 0.01 | | Thomas\_Support\_Letter\_8.28.12.jpg | History | 0.01 | | | |  | | |  |  | | --- | --- | | |  | | --- | |  | | |  | | |
| Performance Site Detail     |  |  |  |  |  |  |  |  |  |  | | --- | --- | --- | --- | --- | --- | --- | --- | --- | --- | | |  |  |  |  |  | | --- | --- | --- | --- | --- | | |  | | --- | | 3-1.2\*  Location or Institution: | |  | | Children & Family Medical Clinic | |  | | |  |  | | --- | --- | | |  | | --- | |  | | |  | | | |  |  |  |  |  |  |  |  |  |  |  | | --- | --- | --- | --- | --- | --- | --- | --- | --- | --- | --- | | |  | | --- | | 3-1.3  Address: | |  | | |  |  | | --- | --- | | City | Allen Park | | State | MI | | Country**\*** | USA | | |  | | |  |  | | --- | --- | | |  | | --- | |  | | |  | | | |  |  |  |  |  |  |  |  |  |  | | --- | --- | --- | --- | --- | --- | --- | --- | --- | --- | | |  | | --- | | 3-1.4\*  Function of this location with respect to this study: | |  | | |  |  |  |  |  | | --- | --- | --- | --- | --- | | | Select all that apply: | | --- | | Recruitment (including screening) | | Secondary data collection (e.g., medical chart review, data abstraction from existing records, etc.) | | Other | |    **If other, please specify:**  Individual practices will identify patients and their practice physicians will review and exclude anyone who should not be contacted/invited to participate in the study | |  | | |  |  | | --- | --- | | |  | | --- | |  | | |  | | | |  |  |  |  |  | | --- | --- | --- | --- | --- | | |  | | --- | | 3-1.5\*  Will this site be "engaged" in the conduct of the research? | |  | | Yes **No** | |  | | |  |  | | --- | --- | | |  | | --- | |  | | |  | | | |  |  |  |  |  | | --- | --- | --- | --- | --- | | |  | | --- | | 3-1.6  If known, provide the Federalwide Assurance (FWA) number for this location. | |  | |  | |  | | |  |  | | --- | --- | | |  | | --- | |  | | |  | | | |  |  |  |  |  | | --- | --- | --- | --- | --- | | |  | | --- | | 3-1.7  If applicable, indicate what organization, agency or government office has reviewed this research and provided its approval (e.g., IRB, ethics committee, school district office, prison official, nursing home administrator). | |  | | UMIRB | |  | | |  |  | | --- | --- | | |  | | --- | |  | | |  | | | |  |  |  |  |  |  |  |  |  |  | | --- | --- | --- | --- | --- | --- | --- | --- | --- | --- | | |  | | --- | | 3-1.8  Upload any location site approval documentation here: | |  | | |  |  |  |  |  | | --- | --- | --- | --- | --- | | | Name | Version | | --- | --- | | DuaAllenPkDSA.pdf | History | 0.01 | | | |  | | |  |  | | --- | --- | | |  | | --- | |  | | |  | | |
| Performance Site Detail     |  |  |  |  |  |  |  |  |  |  | | --- | --- | --- | --- | --- | --- | --- | --- | --- | --- | | |  |  |  |  |  | | --- | --- | --- | --- | --- | | |  | | --- | | 3-1.2\*  Location or Institution: | |  | | David Williams, MD PC | |  | | |  |  | | --- | --- | | |  | | --- | |  | | |  | | | |  |  |  |  |  |  |  |  |  |  |  | | --- | --- | --- | --- | --- | --- | --- | --- | --- | --- | --- | | |  | | --- | | 3-1.3  Address: | |  | | |  |  | | --- | --- | | City | Detroit | | State | MI | | Country**\*** | USA | | |  | | |  |  | | --- | --- | | |  | | --- | |  | | |  | | | |  |  |  |  |  |  |  |  |  |  | | --- | --- | --- | --- | --- | --- | --- | --- | --- | --- | | |  | | --- | | 3-1.4\*  Function of this location with respect to this study: | |  | | |  |  |  |  |  | | --- | --- | --- | --- | --- | | | Select all that apply: | | --- | | Recruitment (including screening) | | Secondary data collection (e.g., medical chart review, data abstraction from existing records, etc.) | | Other | |    **If other, please specify:**  Individual practices will identify patients and their practice physicians will review and exclude anyone who should not be contacted/invited to participate in the study | |  | | |  |  | | --- | --- | | |  | | --- | |  | | |  | | | |  |  |  |  |  | | --- | --- | --- | --- | --- | | |  | | --- | | 3-1.5\*  Will this site be "engaged" in the conduct of the research? | |  | | Yes **No** | |  | | |  |  | | --- | --- | | |  | | --- | |  | | |  | | | |  |  |  |  |  | | --- | --- | --- | --- | --- | | |  | | --- | | 3-1.6  If known, provide the Federalwide Assurance (FWA) number for this location. | |  | |  | |  | | |  |  | | --- | --- | | |  | | --- | |  | | |  | | | |  |  |  |  |  | | --- | --- | --- | --- | --- | | |  | | --- | | 3-1.7  If applicable, indicate what organization, agency or government office has reviewed this research and provided its approval (e.g., IRB, ethics committee, school district office, prison official, nursing home administrator). | |  | |  | |  | | |  |  | | --- | --- | | |  | | --- | |  | | |  | | | |  |  |  |  |  |  |  |  |  |  | | --- | --- | --- | --- | --- | --- | --- | --- | --- | --- | | |  | | --- | | 3-1.8  Upload any location site approval documentation here: | |  | | |  |  |  |  |  | | --- | --- | --- | --- | --- | | | Name | Version | | --- | --- | | Williams\_DSA | History | 0.01 | | | |  | | |  |  | | --- | --- | | |  | | --- | |  | | |  | | |
| Performance Site Detail     |  |  |  |  |  |  |  |  |  |  | | --- | --- | --- | --- | --- | --- | --- | --- | --- | --- | | |  |  |  |  |  | | --- | --- | --- | --- | --- | | |  | | --- | | 3-1.2\*  Location or Institution: | |  | | Downriver Internists PC | |  | | |  |  | | --- | --- | | |  | | --- | |  | | |  | | | |  |  |  |  |  |  |  |  |  |  |  | | --- | --- | --- | --- | --- | --- | --- | --- | --- | --- | --- | | |  | | --- | | 3-1.3  Address: | |  | | |  |  | | --- | --- | | City | Allen Park | | State | MI | | Country**\*** | USA | | |  | | |  |  | | --- | --- | | |  | | --- | |  | | |  | | | |  |  |  |  |  |  |  |  |  |  | | --- | --- | --- | --- | --- | --- | --- | --- | --- | --- | | |  | | --- | | 3-1.4\*  Function of this location with respect to this study: | |  | | |  |  |  |  |  | | --- | --- | --- | --- | --- | | | Select all that apply: | | --- | | Recruitment (including screening) | | Secondary data collection (e.g., medical chart review, data abstraction from existing records, etc.) | | Other | |    **If other, please specify:**  Individual practices will identify patients and their practice physicians will review and exclude anyone who should not be contacted/invited to participate in the study | |  | | |  |  | | --- | --- | | |  | | --- | |  | | |  | | | |  |  |  |  |  | | --- | --- | --- | --- | --- | | |  | | --- | | 3-1.5\*  Will this site be "engaged" in the conduct of the research? | |  | | Yes **No** | |  | | |  |  | | --- | --- | | |  | | --- | |  | | |  | | | |  |  |  |  |  | | --- | --- | --- | --- | --- | | |  | | --- | | 3-1.6  If known, provide the Federalwide Assurance (FWA) number for this location. | |  | |  | |  | | |  |  | | --- | --- | | |  | | --- | |  | | |  | | | |  |  |  |  |  | | --- | --- | --- | --- | --- | | |  | | --- | | 3-1.7  If applicable, indicate what organization, agency or government office has reviewed this research and provided its approval (e.g., IRB, ethics committee, school district office, prison official, nursing home administrator). | |  | | UMIRB | |  | | |  |  | | --- | --- | | |  | | --- | |  | | |  | | | |  |  |  |  |  |  |  |  |  |  | | --- | --- | --- | --- | --- | --- | --- | --- | --- | --- | | |  | | --- | | 3-1.8  Upload any location site approval documentation here: | |  | | |  |  |  |  |  | | --- | --- | --- | --- | --- | | | Name | Version | | --- | --- | | DOWNRIVERDSA.pdf | History | 0.01 | | | |  | | |  |  | | --- | --- | | |  | | --- | |  | | |  | | |
| Performance Site Detail     |  |  |  |  |  |  |  |  |  |  | | --- | --- | --- | --- | --- | --- | --- | --- | --- | --- | | |  |  |  |  |  | | --- | --- | --- | --- | --- | | |  | | --- | | 3-1.2\*  Location or Institution: | |  | | Dua Family Practice | |  | | |  |  | | --- | --- | | |  | | --- | |  | | |  | | | |  |  |  |  |  |  |  |  |  |  |  | | --- | --- | --- | --- | --- | --- | --- | --- | --- | --- | --- | | |  | | --- | | 3-1.3  Address: | |  | | |  |  | | --- | --- | | City | Canton | | State | MI | | Country**\*** | USA | | |  | | |  |  | | --- | --- | | |  | | --- | |  | | |  | | | |  |  |  |  |  |  |  |  |  | | --- | --- | --- | --- | --- | --- | --- | --- | --- | | |  | | --- | | 3-1.4\*  Function of this location with respect to this study: | |  | | |  |  |  |  | | --- | --- | --- | --- | | | Select all that apply: | | --- | | Recruitment (including screening) | | Secondary data collection (e.g., medical chart review, data abstraction from existing records, etc.) | |    **If other, please specify:**  Individual practices will identify patients and their practice physicians will review and exclude anyone who should not be contacted/invited to participate in the study | |  | | |  |  | | --- | --- | | |  | | --- | |  | | |  | | | |  |  |  |  |  | | --- | --- | --- | --- | --- | | |  | | --- | | 3-1.5\*  Will this site be "engaged" in the conduct of the research? | |  | | Yes **No** | |  | | |  |  | | --- | --- | | |  | | --- | |  | | |  | | | |  |  |  |  |  | | --- | --- | --- | --- | --- | | |  | | --- | | 3-1.6  If known, provide the Federalwide Assurance (FWA) number for this location. | |  | |  | |  | | |  |  | | --- | --- | | |  | | --- | |  | | |  | | | |  |  |  |  |  | | --- | --- | --- | --- | --- | | |  | | --- | | 3-1.7  If applicable, indicate what organization, agency or government office has reviewed this research and provided its approval (e.g., IRB, ethics committee, school district office, prison official, nursing home administrator). | |  | | UMIRB | |  | | |  |  | | --- | --- | | |  | | --- | |  | | |  | | | |  |  |  |  |  |  |  |  |  |  | | --- | --- | --- | --- | --- | --- | --- | --- | --- | --- | | |  | | --- | | 3-1.8  Upload any location site approval documentation here: | |  | | |  |  |  |  |  | | --- | --- | --- | --- | --- | | | Name | Version | | --- | --- | | DuaCantonDSA.pdf | History | 0.01 | | | |  | | |  |  | | --- | --- | | |  | | --- | |  | | |  | | |
| Performance Site Detail     |  |  |  |  |  |  |  |  |  |  | | --- | --- | --- | --- | --- | --- | --- | --- | --- | --- | | |  |  |  |  |  | | --- | --- | --- | --- | --- | | |  | | --- | | 3-1.2\*  Location or Institution: | |  | | George C. Hawrot, MD | |  | | |  |  | | --- | --- | | |  | | --- | |  | | |  | | | |  |  |  |  |  |  |  |  |  |  |  | | --- | --- | --- | --- | --- | --- | --- | --- | --- | --- | --- | | |  | | --- | | 3-1.3  Address: | |  | | |  |  | | --- | --- | | City | Dearborn | | State | MI | | Country**\*** | USA | | |  | | |  |  | | --- | --- | | |  | | --- | |  | | |  | | | |  |  |  |  |  |  |  |  |  |  | | --- | --- | --- | --- | --- | --- | --- | --- | --- | --- | | |  | | --- | | 3-1.4\*  Function of this location with respect to this study: | |  | | |  |  |  |  |  | | --- | --- | --- | --- | --- | | | Select all that apply: | | --- | | Recruitment (including screening) | | Secondary data collection (e.g., medical chart review, data abstraction from existing records, etc.) | | Other | |    **If other, please specify:**  Individual practices will identify patients and their practice physicians will review and exclude anyone who should not be contacted/invited to participate in the study | |  | | |  |  | | --- | --- | | |  | | --- | |  | | |  | | | |  |  |  |  |  | | --- | --- | --- | --- | --- | | |  | | --- | | 3-1.5\*  Will this site be "engaged" in the conduct of the research? | |  | | Yes **No** | |  | | |  |  | | --- | --- | | |  | | --- | |  | | |  | | | |  |  |  |  |  | | --- | --- | --- | --- | --- | | |  | | --- | | 3-1.6  If known, provide the Federalwide Assurance (FWA) number for this location. | |  | |  | |  | | |  |  | | --- | --- | | |  | | --- | |  | | |  | | | |  |  |  |  |  | | --- | --- | --- | --- | --- | | |  | | --- | | 3-1.7  If applicable, indicate what organization, agency or government office has reviewed this research and provided its approval (e.g., IRB, ethics committee, school district office, prison official, nursing home administrator). | |  | | UMIRB | |  | | |  |  | | --- | --- | | |  | | --- | |  | | |  | | | |  |  |  |  |  |  |  |  |  |  | | --- | --- | --- | --- | --- | --- | --- | --- | --- | --- | | |  | | --- | | 3-1.8  Upload any location site approval documentation here: | |  | | |  |  |  |  |  | | --- | --- | --- | --- | --- | | | Name | Version | | --- | --- | | Hawrot DSA | History | 0.02 | | | |  | | |  |  | | --- | --- | | |  | | --- | |  | | |  | | |
| Performance Site Detail     |  |  |  |  |  |  |  |  |  |  | | --- | --- | --- | --- | --- | --- | --- | --- | --- | --- | | |  |  |  |  |  | | --- | --- | --- | --- | --- | | |  | | --- | | 3-1.2\*  Location or Institution: | |  | | IPC-Livonia Internal Medicine | |  | | |  |  | | --- | --- | | |  | | --- | |  | | |  | | | |  |  |  |  |  |  |  |  |  |  |  | | --- | --- | --- | --- | --- | --- | --- | --- | --- | --- | --- | | |  | | --- | | 3-1.3  Address: | |  | | |  |  | | --- | --- | | City | Livonia | | State | MI | | Country**\*** | USA | | |  | | |  |  | | --- | --- | | |  | | --- | |  | | |  | | | |  |  |  |  |  |  |  |  |  |  | | --- | --- | --- | --- | --- | --- | --- | --- | --- | --- | | |  | | --- | | 3-1.4\*  Function of this location with respect to this study: | |  | | |  |  |  |  |  | | --- | --- | --- | --- | --- | | | Select all that apply: | | --- | | Recruitment (including screening) | | Secondary data collection (e.g., medical chart review, data abstraction from existing records, etc.) | | Other | |    **If other, please specify:**  Individual practices will identify patients and their practice physicians will review and exclude anyone who should not be contacted/invited to participate in the study | |  | | |  |  | | --- | --- | | |  | | --- | |  | | |  | | | |  |  |  |  |  | | --- | --- | --- | --- | --- | | |  | | --- | | 3-1.5\*  Will this site be "engaged" in the conduct of the research? | |  | | Yes **No** | |  | | |  |  | | --- | --- | | |  | | --- | |  | | |  | | | |  |  |  |  |  | | --- | --- | --- | --- | --- | | |  | | --- | | 3-1.6  If known, provide the Federalwide Assurance (FWA) number for this location. | |  | |  | |  | | |  |  | | --- | --- | | |  | | --- | |  | | |  | | | |  |  |  |  |  | | --- | --- | --- | --- | --- | | |  | | --- | | 3-1.7  If applicable, indicate what organization, agency or government office has reviewed this research and provided its approval (e.g., IRB, ethics committee, school district office, prison official, nursing home administrator). | |  | | UMIRB | |  | | |  |  | | --- | --- | | |  | | --- | |  | | |  | | | |  |  |  |  |  |  |  |  |  |  | | --- | --- | --- | --- | --- | --- | --- | --- | --- | --- | | |  | | --- | | 3-1.8  Upload any location site approval documentation here: | |  | | |  |  |  |  |  | | --- | --- | --- | --- | --- | | | Name | Version | | --- | --- | | LIVONIADSA.pdf | History | 0.01 | | | |  | | |  |  | | --- | --- | | |  | | --- | |  | | |  | | |
| Performance Site Detail     |  |  |  |  |  |  |  |  |  |  | | --- | --- | --- | --- | --- | --- | --- | --- | --- | --- | | |  |  |  |  |  | | --- | --- | --- | --- | --- | | |  | | --- | | 3-1.2\*  Location or Institution: | |  | | Lonnie Joe Jr., MD | |  | | |  |  | | --- | --- | | |  | | --- | |  | | |  | | | |  |  |  |  |  |  |  |  |  |  |  | | --- | --- | --- | --- | --- | --- | --- | --- | --- | --- | --- | | |  | | --- | | 3-1.3  Address: | |  | | |  |  | | --- | --- | | City | Southfield | | State | MI | | Country**\*** | USA | | |  | | |  |  | | --- | --- | | |  | | --- | |  | | |  | | | |  |  |  |  |  |  |  |  |  |  | | --- | --- | --- | --- | --- | --- | --- | --- | --- | --- | | |  | | --- | | 3-1.4\*  Function of this location with respect to this study: | |  | | |  |  |  |  |  | | --- | --- | --- | --- | --- | | | Select all that apply: | | --- | | Recruitment (including screening) | | Secondary data collection (e.g., medical chart review, data abstraction from existing records, etc.) | | Other | |    **If other, please specify:**  Individual practices will identify patients and their practice physicians will review and exclude anyone who should not be contacted/invited to participate in the study | |  | | |  |  | | --- | --- | | |  | | --- | |  | | |  | | | |  |  |  |  |  | | --- | --- | --- | --- | --- | | |  | | --- | | 3-1.5\*  Will this site be "engaged" in the conduct of the research? | |  | | Yes **No** | |  | | |  |  | | --- | --- | | |  | | --- | |  | | |  | | | |  |  |  |  |  | | --- | --- | --- | --- | --- | | |  | | --- | | 3-1.6  If known, provide the Federalwide Assurance (FWA) number for this location. | |  | |  | |  | | |  |  | | --- | --- | | |  | | --- | |  | | |  | | | |  |  |  |  |  | | --- | --- | --- | --- | --- | | |  | | --- | | 3-1.7  If applicable, indicate what organization, agency or government office has reviewed this research and provided its approval (e.g., IRB, ethics committee, school district office, prison official, nursing home administrator). | |  | |  | |  | | |  |  | | --- | --- | | |  | | --- | |  | | |  | | | |  |  |  |  |  |  |  |  |  |  | | --- | --- | --- | --- | --- | --- | --- | --- | --- | --- | | |  | | --- | | 3-1.8  Upload any location site approval documentation here: | |  | | |  |  |  |  |  | | --- | --- | --- | --- | --- | | | Name | Version | | --- | --- | | Joe\_DSA\_9.24.pdf | History | 0.01 | | | |  | | |  |  | | --- | --- | | |  | | --- | |  | | |  | | |
| Performance Site Detail     |  |  |  |  |  |  |  |  |  |  | | --- | --- | --- | --- | --- | --- | --- | --- | --- | --- | | |  |  |  |  |  | | --- | --- | --- | --- | --- | | |  | | --- | | 3-1.2\*  Location or Institution: | |  | | Mark W. Sawka, MD | |  | | |  |  | | --- | --- | | |  | | --- | |  | | |  | | | |  |  |  |  |  |  |  |  |  |  |  | | --- | --- | --- | --- | --- | --- | --- | --- | --- | --- | --- | | |  | | --- | | 3-1.3  Address: | |  | | |  |  | | --- | --- | | City | Woodhaven | | State | MI | | Country**\*** | USA | | |  | | |  |  | | --- | --- | | |  | | --- | |  | | |  | | | |  |  |  |  |  |  |  |  |  |  | | --- | --- | --- | --- | --- | --- | --- | --- | --- | --- | | |  | | --- | | 3-1.4\*  Function of this location with respect to this study: | |  | | |  |  |  |  |  | | --- | --- | --- | --- | --- | | | Select all that apply: | | --- | | Recruitment (including screening) | | Secondary data collection (e.g., medical chart review, data abstraction from existing records, etc.) | | Other | |    **If other, please specify:**  Individual practices will identify patients and their practice physicians will review and exclude anyone who should not be contacted/invited to participate in the study | |  | | |  |  | | --- | --- | | |  | | --- | |  | | |  | | | |  |  |  |  |  | | --- | --- | --- | --- | --- | | |  | | --- | | 3-1.5\*  Will this site be "engaged" in the conduct of the research? | |  | | Yes **No** | |  | | |  |  | | --- | --- | | |  | | --- | |  | | |  | | | |  |  |  |  |  | | --- | --- | --- | --- | --- | | |  | | --- | | 3-1.6  If known, provide the Federalwide Assurance (FWA) number for this location. | |  | |  | |  | | |  |  | | --- | --- | | |  | | --- | |  | | |  | | | |  |  |  |  |  | | --- | --- | --- | --- | --- | | |  | | --- | | 3-1.7  If applicable, indicate what organization, agency or government office has reviewed this research and provided its approval (e.g., IRB, ethics committee, school district office, prison official, nursing home administrator). | |  | | UMIRB | |  | | |  |  | | --- | --- | | |  | | --- | |  | | |  | | | |  |  |  |  |  |  |  |  |  |  | | --- | --- | --- | --- | --- | --- | --- | --- | --- | --- | | |  | | --- | | 3-1.8  Upload any location site approval documentation here: | |  | | |  |  |  |  |  | | --- | --- | --- | --- | --- | | | Name | Version | | --- | --- | | SAWKADSA.pdf | History | 0.01 | | | |  | | |  |  | | --- | --- | | |  | | --- | |  | | |  | | |
| Performance Site Detail     |  |  |  |  |  |  |  |  |  |  | | --- | --- | --- | --- | --- | --- | --- | --- | --- | --- | | |  |  |  |  |  | | --- | --- | --- | --- | --- | | |  | | --- | | 3-1.2\*  Location or Institution: | |  | | Michigan State university | |  | | |  |  | | --- | --- | | |  | | --- | |  | | |  | | | |  |  |  |  |  |  |  |  |  |  |  | | --- | --- | --- | --- | --- | --- | --- | --- | --- | --- | --- | | |  | | --- | | 3-1.3  Address: | |  | | |  |  | | --- | --- | | City |  | | State | MI | | Country**\*** | USA | | |  | | |  |  | | --- | --- | | |  | | --- | |  | | |  | | | |  |  |  |  |  |  |  |  |  |  |  |  |  | | --- | --- | --- | --- | --- | --- | --- | --- | --- | --- | --- | --- | --- | | |  | | --- | | 3-1.4\*  Function of this location with respect to this study: | |  | | |  |  |  |  |  |  |  |  | | --- | --- | --- | --- | --- | --- | --- | --- | | | Select all that apply: | | --- | | Recruitment (including screening) | | Interaction (e.g., information gathering, survey, interview, focus groups, etc.) | | Intervention (e.g., use of drug or device, medical procedures, educational intervention, group intervention, social/psychological intervention etc.) | | Qualitative research (e.g., 'member checking', open-ended questions, etc.) | | Primary or secondary analysis (data/specimen) | | Storage (data/specimen) | |    **If other, please specify:** | |  | | |  |  | | --- | --- | | |  | | --- | |  | | |  | | | |  |  |  |  |  | | --- | --- | --- | --- | --- | | |  | | --- | | 3-1.5\*  Will this site be "engaged" in the conduct of the research? | |  | | **Yes** No | |  | | |  |  | | --- | --- | | |  | | --- | |  | | |  | | | |  |  |  |  |  | | --- | --- | --- | --- | --- | | |  | | --- | | 3-1.6  If known, provide the Federalwide Assurance (FWA) number for this location. | |  | |  | |  | | |  |  | | --- | --- | | |  | | --- | |  | | |  | | | |  |  |  |  |  | | --- | --- | --- | --- | --- | | |  | | --- | | 3-1.7  If applicable, indicate what organization, agency or government office has reviewed this research and provided its approval (e.g., IRB, ethics committee, school district office, prison official, nursing home administrator). | |  | | MSU's IRB | |  | | |  |  | | --- | --- | | |  | | --- | |  | | |  | | | |  |  |  |  |  |  |  |  |  |  | | --- | --- | --- | --- | --- | --- | --- | --- | --- | --- | | |  | | --- | | 3-1.8  Upload any location site approval documentation here: | |  | | |  |  |  |  |  | | --- | --- | --- | --- | --- | | | Name | Version | | --- | --- | | MSU's IRB Approval | History | 0.01 | | | |  | | |  |  | | --- | --- | | |  | | --- | |  | | |  | | |
| Performance Site Detail     |  |  |  |  |  |  |  |  |  |  | | --- | --- | --- | --- | --- | --- | --- | --- | --- | --- | | |  |  |  |  |  | | --- | --- | --- | --- | --- | | |  | | --- | | 3-1.2\*  Location or Institution: | |  | | Morang Chester Clinic PC | |  | | |  |  | | --- | --- | | |  | | --- | |  | | |  | | | |  |  |  |  |  |  |  |  |  |  |  | | --- | --- | --- | --- | --- | --- | --- | --- | --- | --- | --- | | |  | | --- | | 3-1.3  Address: | |  | | |  |  | | --- | --- | | City | Detroit | | State | MI | | Country**\*** | USA | | |  | | |  |  | | --- | --- | | |  | | --- | |  | | |  | | | |  |  |  |  |  |  |  |  |  |  | | --- | --- | --- | --- | --- | --- | --- | --- | --- | --- | | |  | | --- | | 3-1.4\*  Function of this location with respect to this study: | |  | | |  |  |  |  |  | | --- | --- | --- | --- | --- | | | Select all that apply: | | --- | | Recruitment (including screening) | | Secondary data collection (e.g., medical chart review, data abstraction from existing records, etc.) | | Other | |    **If other, please specify:**  Individual practices will identify patients and their practice physicians will review and exclude anyone who should not be contacted/invited to participate in the study | |  | | |  |  | | --- | --- | | |  | | --- | |  | | |  | | | |  |  |  |  |  | | --- | --- | --- | --- | --- | | |  | | --- | | 3-1.5\*  Will this site be "engaged" in the conduct of the research? | |  | | Yes **No** | |  | | |  |  | | --- | --- | | |  | | --- | |  | | |  | | | |  |  |  |  |  | | --- | --- | --- | --- | --- | | |  | | --- | | 3-1.6  If known, provide the Federalwide Assurance (FWA) number for this location. | |  | |  | |  | | |  |  | | --- | --- | | |  | | --- | |  | | |  | | | |  |  |  |  |  | | --- | --- | --- | --- | --- | | |  | | --- | | 3-1.7  If applicable, indicate what organization, agency or government office has reviewed this research and provided its approval (e.g., IRB, ethics committee, school district office, prison official, nursing home administrator). | |  | | UMIRB | |  | | |  |  | | --- | --- | | |  | | --- | |  | | |  | | | |  |  |  |  |  |  |  |  |  |  | | --- | --- | --- | --- | --- | --- | --- | --- | --- | --- | | |  | | --- | | 3-1.8  Upload any location site approval documentation here: | |  | | |  |  |  |  |  | | --- | --- | --- | --- | --- | | | Name | Version | | --- | --- | | BRAVERDSA.pdf | History | 0.01 | | | |  | | |  |  | | --- | --- | | |  | | --- | |  | | |  | | |
| Performance Site Detail     |  |  |  |  |  |  |  |  |  |  | | --- | --- | --- | --- | --- | --- | --- | --- | --- | --- | | |  |  |  |  |  | | --- | --- | --- | --- | --- | | |  | | --- | | 3-1.2\*  Location or Institution: | |  | | Rice Lanzilote Dos and Egan MD | |  | | |  |  | | --- | --- | | |  | | --- | |  | | |  | | | |  |  |  |  |  |  |  |  |  |  |  | | --- | --- | --- | --- | --- | --- | --- | --- | --- | --- | --- | | |  | | --- | | 3-1.3  Address: | |  | | |  |  | | --- | --- | | City | Detroit | | State | MI | | Country**\*** | USA | | |  | | |  |  | | --- | --- | | |  | | --- | |  | | |  | | | |  |  |  |  |  |  |  |  |  |  | | --- | --- | --- | --- | --- | --- | --- | --- | --- | --- | | |  | | --- | | 3-1.4\*  Function of this location with respect to this study: | |  | | |  |  |  |  |  | | --- | --- | --- | --- | --- | | | Select all that apply: | | --- | | Recruitment (including screening) | | Secondary data collection (e.g., medical chart review, data abstraction from existing records, etc.) | | Other | |    **If other, please specify:**  Individual practices will identify patients and their practice physicians will review and exclude anyone who should not be contacted/invited to participate in the study | |  | | |  |  | | --- | --- | | |  | | --- | |  | | |  | | | |  |  |  |  |  | | --- | --- | --- | --- | --- | | |  | | --- | | 3-1.5\*  Will this site be "engaged" in the conduct of the research? | |  | | Yes **No** | |  | | |  |  | | --- | --- | | |  | | --- | |  | | |  | | | |  |  |  |  |  | | --- | --- | --- | --- | --- | | |  | | --- | | 3-1.6  If known, provide the Federalwide Assurance (FWA) number for this location. | |  | |  | |  | | |  |  | | --- | --- | | |  | | --- | |  | | |  | | | |  |  |  |  |  | | --- | --- | --- | --- | --- | | |  | | --- | | 3-1.7  If applicable, indicate what organization, agency or government office has reviewed this research and provided its approval (e.g., IRB, ethics committee, school district office, prison official, nursing home administrator). | |  | |  | |  | | |  |  | | --- | --- | | |  | | --- | |  | | |  | | | |  |  |  |  |  |  |  |  |  |  | | --- | --- | --- | --- | --- | --- | --- | --- | --- | --- | | |  | | --- | | 3-1.8  Upload any location site approval documentation here: | |  | | |  |  |  |  |  | | --- | --- | --- | --- | --- | | | Name | Version | | --- | --- | | Rice\_DSA\_9.4.jpg | History | 0.01 | | | |  | | |  |  | | --- | --- | | |  | | --- | |  | | |  | | |
| Performance Site Detail     |  |  |  |  |  |  |  |  |  |  | | --- | --- | --- | --- | --- | --- | --- | --- | --- | --- | | |  |  |  |  |  | | --- | --- | --- | --- | --- | | |  | | --- | | 3-1.2\*  Location or Institution: | |  | | University of Michigan | |  | | |  |  | | --- | --- | | |  | | --- | |  | | |  | | | |  |  |  |  |  |  |  |  |  |  |  | | --- | --- | --- | --- | --- | --- | --- | --- | --- | --- | --- | | |  | | --- | | 3-1.3  Address: | |  | | |  |  | | --- | --- | | City |  | | State |  | | Country**\*** | USA | | |  | | |  |  | | --- | --- | | |  | | --- | |  | | |  | | | |  |  |  |  |  |  |  |  |  |  |  |  |  |  |  |  | | --- | --- | --- | --- | --- | --- | --- | --- | --- | --- | --- | --- | --- | --- | --- | --- | | |  | | --- | | 3-1.4\*  Function of this location with respect to this study: | |  | | |  |  |  |  |  |  |  |  |  |  |  | | --- | --- | --- | --- | --- | --- | --- | --- | --- | --- | --- | | | Select all that apply: | | --- | | Recruitment (including screening) | | Interaction (e.g., information gathering, survey, interview, focus groups, etc.) | | Intervention (e.g., use of drug or device, medical procedures, educational intervention, group intervention, social/psychological intervention etc.) | | Observation of behavior (direct or indirect) | | Qualitative research (e.g., 'member checking', open-ended questions, etc.) | | Secondary data collection (e.g., medical chart review, data abstraction from existing records, etc.) | | Primary or secondary analysis (data/specimen) | | Storage (data/specimen) | | Other | |    **If other, please specify:**  Individual practices will identify patients and their practice physicians will review and exclude anyone who should not be contacted/invited to participate in the study | |  | | |  |  | | --- | --- | | |  | | --- | |  | | |  | | | |  |  |  |  |  | | --- | --- | --- | --- | --- | | |  | | --- | | 3-1.5\*  Will this site be "engaged" in the conduct of the research? | |  | | **Yes** No | |  | | |  |  | | --- | --- | | |  | | --- | |  | | |  | | | |  |  |  |  |  | | --- | --- | --- | --- | --- | | |  | | --- | | 3-1.6  If known, provide the Federalwide Assurance (FWA) number for this location. | |  | | FWA00004969 | |  | | |  |  | | --- | --- | | |  | | --- | |  | | |  | | | |  |  |  |  |  | | --- | --- | --- | --- | --- | | |  | | --- | | 3-1.7  If applicable, indicate what organization, agency or government office has reviewed this research and provided its approval (e.g., IRB, ethics committee, school district office, prison official, nursing home administrator). | |  | | IRB | |  | | |  |  | | --- | --- | | |  | | --- | |  | | |  | | | |  |  |  |  |  |  |  |  |  |  |  |  |  |  |  |  |  |  | | --- | --- | --- | --- | --- | --- | --- | --- | --- | --- | --- | --- | --- | --- | --- | --- | --- | --- | | |  | | --- | | 3-1.8  Upload any location site approval documentation here: | |  | | |  |  |  |  |  |  |  |  |  |  |  |  |  | | --- | --- | --- | --- | --- | --- | --- | --- | --- | --- | --- | --- | --- | | | Name | Version | | --- | --- | |  |  |  |  |  |  |  |  |  |  | | --- | --- | --- | --- | --- | --- | --- | --- | --- | --- | | There are no items to display | | | | | | | | | | | | |  | | |  |  | | --- | --- | | |  | | --- | |  | | |  | | |
| Performance Site Detail     |  |  |  |  |  |  |  |  |  |  | | --- | --- | --- | --- | --- | --- | --- | --- | --- | --- | | |  |  |  |  |  | | --- | --- | --- | --- | --- | | |  | | --- | | 3-1.2\*  Location or Institution: | |  | | University of Michigan Family Medicine Clinics | |  | | |  |  | | --- | --- | | |  | | --- | |  | | |  | | | |  |  |  |  |  |  |  |  |  |  |  | | --- | --- | --- | --- | --- | --- | --- | --- | --- | --- | --- | | |  | | --- | | 3-1.3  Address: | |  | | |  |  | | --- | --- | | City |  | | State | MI | | Country**\*** | USA | | |  | | |  |  | | --- | --- | | |  | | --- | |  | | |  | | | |  |  |  |  |  |  |  |  |  |  |  | | --- | --- | --- | --- | --- | --- | --- | --- | --- | --- | --- | | |  | | --- | | 3-1.4\*  Function of this location with respect to this study: | |  | | |  |  |  |  |  |  | | --- | --- | --- | --- | --- | --- | | | Select all that apply: | | --- | | Recruitment (including screening) | | Interaction (e.g., information gathering, survey, interview, focus groups, etc.) | | Intervention (e.g., use of drug or device, medical procedures, educational intervention, group intervention, social/psychological intervention etc.) | | Secondary data collection (e.g., medical chart review, data abstraction from existing records, etc.) | |    **If other, please specify:** | |  | | |  |  | | --- | --- | | |  | | --- | |  | | |  | | | |  |  |  |  |  | | --- | --- | --- | --- | --- | | |  | | --- | | 3-1.5\*  Will this site be "engaged" in the conduct of the research? | |  | | **Yes** No | |  | | |  |  | | --- | --- | | |  | | --- | |  | | |  | | | |  |  |  |  |  | | --- | --- | --- | --- | --- | | |  | | --- | | 3-1.6  If known, provide the Federalwide Assurance (FWA) number for this location. | |  | |  | |  | | |  |  | | --- | --- | | |  | | --- | |  | | |  | | | |  |  |  |  |  | | --- | --- | --- | --- | --- | | |  | | --- | | 3-1.7  If applicable, indicate what organization, agency or government office has reviewed this research and provided its approval (e.g., IRB, ethics committee, school district office, prison official, nursing home administrator). | |  | | UM, IBRMED | |  | | |  |  | | --- | --- | | |  | | --- | |  | | |  | | | |  |  |  |  |  |  |  |  |  |  | | --- | --- | --- | --- | --- | --- | --- | --- | --- | --- | | |  | | --- | | 3-1.8  Upload any location site approval documentation here: | |  | | |  |  |  |  |  | | --- | --- | --- | --- | --- | | | Name | Version | | --- | --- | | UM Family Medicine Approval to Conduct Research | History | 0.01 | | | |  | | |  |  | | --- | --- | | |  | | --- | |  | | |  | | |
| Performance Site Detail     |  |  |  |  |  |  |  |  |  |  | | --- | --- | --- | --- | --- | --- | --- | --- | --- | --- | | |  |  |  |  |  | | --- | --- | --- | --- | --- | | |  | | --- | | 3-1.2\*  Location or Institution: | |  | | up to 10 yet to be recruited practices in the Metro Detroit area | |  | | |  |  | | --- | --- | | |  | | --- | |  | | |  | | | |  |  |  |  |  |  |  |  |  |  |  | | --- | --- | --- | --- | --- | --- | --- | --- | --- | --- | --- | | |  | | --- | | 3-1.3  Address: | |  | | |  |  | | --- | --- | | City |  | | State | MI | | Country**\*** | USA | | |  | | |  |  | | --- | --- | | |  | | --- | |  | | |  | | | |  |  |  |  |  |  |  |  |  |  | | --- | --- | --- | --- | --- | --- | --- | --- | --- | --- | | |  | | --- | | 3-1.4\*  Function of this location with respect to this study: | |  | | |  |  |  |  |  | | --- | --- | --- | --- | --- | | | Select all that apply: | | --- | | Recruitment (including screening) | | Secondary data collection (e.g., medical chart review, data abstraction from existing records, etc.) | | Other | |    **If other, please specify:**  Individual practices will identify patients and their practice physicians will review and exclude anyone who should not be contacted/invited to participate in the study | |  | | |  |  | | --- | --- | | |  | | --- | |  | | |  | | | |  |  |  |  |  | | --- | --- | --- | --- | --- | | |  | | --- | | 3-1.5\*  Will this site be "engaged" in the conduct of the research? | |  | | Yes **No** | |  | | |  |  | | --- | --- | | |  | | --- | |  | | |  | | | |  |  |  |  |  | | --- | --- | --- | --- | --- | | |  | | --- | | 3-1.6  If known, provide the Federalwide Assurance (FWA) number for this location. | |  | |  | |  | | |  |  | | --- | --- | | |  | | --- | |  | | |  | | | |  |  |  |  |  | | --- | --- | --- | --- | --- | | |  | | --- | | 3-1.7  If applicable, indicate what organization, agency or government office has reviewed this research and provided its approval (e.g., IRB, ethics committee, school district office, prison official, nursing home administrator). | |  | |  | |  | | |  |  | | --- | --- | | |  | | --- | |  | | |  | | | |  |  |  |  |  |  |  |  |  |  |  |  |  |  |  |  |  |  | | --- | --- | --- | --- | --- | --- | --- | --- | --- | --- | --- | --- | --- | --- | --- | --- | --- | --- | | |  | | --- | | 3-1.8  Upload any location site approval documentation here: | |  | | |  |  |  |  |  |  |  |  |  |  |  |  |  | | --- | --- | --- | --- | --- | --- | --- | --- | --- | --- | --- | --- | --- | | | Name | Version | | --- | --- | |  |  |  |  |  |  |  |  |  |  | | --- | --- | --- | --- | --- | --- | --- | --- | --- | --- | | There are no items to display | | | | | | | | | | | | |  | | |  |  | | --- | --- | | |  | | --- | |  | | |  | | |

|  |  |  |
| --- | --- | --- |
| |  |  | | --- | --- | |  |  | |
| 05. Research Design     |  |  |  |  |  |  |  |  |  |  | | --- | --- | --- | --- | --- | --- | --- | --- | --- | --- | | |  |  |  |  |  | | --- | --- | --- | --- | --- | | |  | | --- | | 5.1\*  Is there a stand-alone scientific protocol document and/or research plan associated with this application? | |  | | **Yes** No | |  | | |  |  | | --- | --- | | |  | | --- | |  | | |  | |      |  |  |  |  |  |  |  |  |  |  |  |  |  |  |  |  |  | | --- | --- | --- | --- | --- | --- | --- | --- | --- | --- | --- | --- | --- | --- | --- | --- | --- | | |  |  |  |  |  |  |  |  |  |  |  |  | | --- | --- | --- | --- | --- | --- | --- | --- | --- | --- | --- | --- | | |  | | --- | | 5.1.1\*  Click ADD to attach the document(s) electronically. | |  | | |  |  |  |  |  |  |  | | --- | --- | --- | --- | --- | --- | --- | | | Name | Version | | --- | --- | | CLEAN\_DATESprotocol\_v-1.1.6\_6.25.13.doc | History | 0.10 | | TRACK\_DATESprotocol\_v-1.1.6\_6.25.13.doc | History | 0.07 | | | |  | | |  |  | | --- | --- | | |  | | --- | |  | | |  | | | |  |  |  |  |  |  |  |  |  |  |  |  |  |  |  |  |  |  |  |  | | --- | --- | --- | --- | --- | --- | --- | --- | --- | --- | --- | --- | --- | --- | --- | --- | --- | --- | --- | --- | | |  | | --- | | 5.1.2\*  Indicate the section where each of the following are covered in the attached protocol: | |  | | |  |  |  | | --- | --- | --- | | **Objective** |  | In specific aims | | **Specific Aim/Hypothesis** |  | In specific aims | | **Background Information** |  | In specific aims | | **Methodology** |  | Phase 1, 2, 3 | | **Statistical Design** |  | Phase 1, Overview, Specific Aims | | |  | | |  |  | | --- | --- | | |  | | --- | |  | | |  | | | |  |  |  |  |  | | --- | --- | --- | --- | --- | | |  | | --- | | 5.1.3\*  Study team Experience: Briefly outline the experience and competence of the study team to pursue the proposed study. | |  | | Jimbo: The goal of the proposed research is to improve the rate of colorectal cancer (CRC) screening by using colorectal Web (CW), an innovative, interactive decision aid that helps patients understand their personal risk regarding CRC, clarify their preferences, and choose a CRC screening test option that best matches their risks and preferences. Specifically, we plan to conduct a randomized controlled trial comparing rates of CRC screening completion and shifts in patient behavioral factors, such as intent, between the CW Group using CW and the Control Group using a non-interactive standard web. We will also evaluate how the decision aid and its influence on patients affect patient/physician interaction and shared decision making. Dr. Jimbo has the expertise, leadership, and motivation necessary to successfully carry out the proposed work. Having worked as a family physician in both urban and rural underserved areas, Dr. Jimbo has first-hand knowledge of how competing patient issues can crowd out important preventive service discussions, such as CRC screening, from the patient's clinic visit. Initially trained in basic laboratory research, Dr. Jimbo has obtained expertise in behavioral intervention and research through formal training in public health at the University of North Carolina and hands-on research on patient and physician-targeted behavioral interventions to improve follow-up of abnormal CRC screening results at Thomas Jefferson University. As Principal Investigator in several university- and foundation- funded grants, Dr. Jimbo laid the groundwork for the proposed research by improving CW for real-world usage and establishing the feasibility of administering and evaluating the proposed behavioral intervention in diverse settings, including the practices in the Great Lakes Research Into Practice Network (GRIN), the setting for the proposed study. During this time, Dr. Jimbo has worked closely with all of the co-investigators, each of whom brings distinct and complementary expertise to the proposed project. Dr. Jimbo successfully administered the projects including institutional review board approval, staffing, data protection, and budget. Through these previous efforts, Dr. Jimbo has developed a protocol of regular communication among the research members, patient recruitment plan, data protection, and analysis. In summary, Dr. Jimbo has a demonstrated record of successful and productive research projects in an area of significant impact, CRC screening, and his expertise and experience have prepared him to lead the proposed project.   Ruffin: Dr. Ruffin has 20 years of experience as a NIH-funded investigator focused in the area of cancer prevention and early detection. He was responsible for the development and testing of the original Colorectal Web (CW) as highlighted in his most relevant publications. As part of his K24 Mid-Career Award from NCI, Dr. Ruffin has been a mentor to Dr. Jimbo. He has worked closely with Dr. Jimbo on the further refinements of CW by adding the interactive risk assessment, testing the feasibility of implementing CW in practice, and developing the original study application and revision. In this study, Dr. Ruffin will serve as the senior investigator and mentor to Dr. Jimbo. Dr. Ruffin has experience conducting studies in community-based practice setting, including Detroit, as highlighted by his recent and best publications. He will meet weekly with Dr. Jimbo to assure that the study is progressing. He will work with Dr. Jimbo in all aspects of the study, including hiring and training staff, interfacing with community practice, human subject issues, data capture and synthesis, and preparing presentations, and publication. Dr. Ruffin will be trained in the scoring of the audiotapes using the Observing Patient Involvement (OPTION) score. He will review and score the audiotapes of patient encounters.    Hawley: Dr. Hawley is a social/behavioral scientist trained in health services research with a focus on cancer prevention and control. Her research focuses specifically on how physician patient communication and patients' preferences impact cancer screening and treatment decisions. Dr. Hawley has specific expertise in evaluating factors associated with use of cancer screening and treatment services. Dr. Hawley has developed and tested interventions that integrate preference elicitation exercises to help patients with cancer related decision making in real time. In this capacity, Dr. Hawley has considerable experience in implementation and evaluation of cancer screening interventions in randomized controlled trials. Specifically, Dr. Hawley directs two large RCTs of a preference-tailored intervention for improving CRC screening uptake, using conjoint analysis to elicit patients' preferences. Dr. Hawley brings her skills in health services research and intervention methodology, cancer screening, and physician- patient decision making to the proposed project. The evaluation of an intervention directed toward improving uptake of CRC screening, by incorporating patients' preferences and risk assessments is both innovative and significant. The results of this project will have important implications for understanding how addressing these factors can improve shared decision making and, ultimately, screening adherence.  Strecher: Dr. Strecher has been involved in the development, evaluation, and testing of Colorectal Web (CW) since the inception. He has collaborated with the research team in further refinement of CW and the design of this current study proposal. Dr. Strecher will provide scientific expertise regarding health communications and behavior change theory, study design and intervention development, especially in the area of individually tailored messages. He will work closely with the study investigators to assure that the messages and communications strategies reflect the best practices of theory and empirical research. Dr. Strecher will also assist in the data analysis, presentations, and manuscript development of the research results. Dr. Strecher will attend senior staff meetings and required project meetings at the NIH.  Sen: Dr. Sen is a biostatistician in the Department of Family Medicine. His role on the proposed study is that of co-investigator with biostatistical expertise. Dr. Sen has significant prior experience on working on several projects with the PI. Dr. Sen also works closely with several investigators in the Department of Family Medicine as well as the Cancer Center at the University of Michigan. He will be available to support the study by contributing expertise to the overall development and testing of the study design and analyses, and interpretation of findings. Dr. Sen will further participate in the development, review and publication of related papers and abstracts and be able to present the findings in meetings and conferences.  Dr. Karen Kelly-Blake is a medical anthropologist with extensive experience analyzing patient understanding of content in decision aids. In this study, she will be responsible for coding the transcripts and qualitative data analysis, described in more detail in the Specific Aims section of the protocol. She will not have any interaction with the participants. Dr. Kelly-Blake has applied for a minority supplement (please see attached in section 44). If funded, she will become a Co-I on this project.  Dr. Lafata is professor with the department of Social and Behavioral Health at Virginia Commonwealth university in Richmond VA. Her training is in health services research and economics. Her research currently centers on patient decision-making regarding colorectal cancer control and prevention as well as patient decision making regarding diabetes management. She served as PI on an NIH-funded study, the Preventive Health Study(PHS), that used direct observation and audio-recordings of 500 patient-physician interactions during annual “check-ups,” linked with patient survey and claims data to evaluate colorectal cancer screening decision making in primary care. She also has served as a co-investigator on a number of practice-based trials evaluating interventions designed to improve colorectal cancer screening in primary care. She has participated in NIH-sponsored studies evaluating the appropriateness of using claims data to study the care delivered to cancer survivors and an AHRQ-funded study to evaluate the use of claims data to detect medication errors in the outpatient setting. Through these and other efforts, she has extensive experience working with delivery and financing organizations to develop, implement and evaluate interventions designed to improve cancer control and routine chronic care management.  Larry An, MD, is a general internist and Associate Professor of Internal Medicine at the University of Michigan. He is the Director of the Center for Health Communications Research and Scientific Director of the Cancer Survivorship Program. Dr. An did his medical training and internal medicine residency at the University of Michigan before completing fellowships with the Robert Wood Johnson Clinical Scholars program and the Agency for Health Care Policy and Research. For 11 years prior to joining the University of Michigan faculty, Dr. An was on faculty at the University of Minnesota where he worked closely with health plans, health care provider organizations, and the state health department. Dr. An's research teams have developed and tested several web-based tailored interventions for health behavior change. His research has focused primarily on tobacco control in the areas of clinical guideline implementation and health systems change, design and evaluation of statewide tobacco cessation services, and development of innovative web-assisted tobacco interventions, with an emphasis on youth. | |  | | |  |  | | --- | --- | | |  | | --- | |  | | |  | |     |  |  |  |  |  |  |  |  |  |  | | --- | --- | --- | --- | --- | --- | --- | --- | --- | --- | | |  |  |  |  |  | | --- | --- | --- | --- | --- | | |  | | --- | | 5.2\*  Will the involvement of ANY subjects in this study be limited to analysis of their existing data or specimens? | |  | | Yes **No** | |  | | |  |  | | --- | --- | | |  | | --- | |  | | |  | |      |  |  |  |  |  |  |  |  |  |  | | --- | --- | --- | --- | --- | --- | --- | --- | --- | --- | | |  |  |  |  |  | | --- | --- | --- | --- | --- | | |  | | --- | | 5.2.1\*  How many subjects are represented in the data or specimens to be analyzed? | |  | | *(do not enter commas, dots, or special characters)* | |  | | |  |  | | --- | --- | | |  | | --- | |  | | |  | |     |  |  |  |  |  |  |  |  |  |  | | --- | --- | --- | --- | --- | --- | --- | --- | --- | --- | | |  |  |  |  |  | | --- | --- | --- | --- | --- | | |  | | --- | | 5.3\*  Will the study involve recruitment and/or participation of subjects in order to produce new data (e.g., surveys, interaction, intervention)?  *[Require sections 8-2 and 11-3]* | |  | | **Yes** No | |  | | |  |  | | --- | --- | | |  | | --- | |  | | |  | | | |  |  |  |  |  | | --- | --- | --- | --- | --- | | |  | | --- | | 5.4\*  List the inclusion and exclusion criteria for this study population and/or data set. (If covered in attached protocol, indicate section) | |  | | See Inclusion/Exclusion Section in attached protcol | |  | | |  |  | | --- | --- | | |  | | --- | |  | | |  | | | |  |  |  |  |  | | --- | --- | --- | --- | --- | | |  | | --- | | 5.5  Identify any racial, ethnic, or gender group(s) that will be specifically excluded from participation in this research study and provide a compelling justification for such exclusion: | |  | |  | |  | | |  |  | | --- | --- | | |  | | --- | |  | | |  | | | |  |  |  |  |  |  |  |  |  |  |  | | --- | --- | --- | --- | --- | --- | --- | --- | --- | --- | --- | | |  | | --- | | 5.6\*  Indicate the age range (in years) of the subject population in this study. | |  | | |  |  |  | | --- | --- | --- | | **Minimum Age:** |  | 50 | | **Maximum Age:** |  | 75 If no upper limit, enter "999" | | |  | | |  |  | | --- | --- | | |  | | --- | |  | | |  | | |

|  |  |  |
| --- | --- | --- |
| |  |  | | --- | --- | |  |  | |
| 06. Benefits and Risks   |  |  |  |  |  |  |  |  |  |  | | --- | --- | --- | --- | --- | --- | --- | --- | --- | --- | | |  |  |  |  |  | | --- | --- | --- | --- | --- | | |  | | --- | | 6.1 \*  Describe the potential benefits of this research to society. | |  | | What we learn may one day help more people complete the colon cancer screening test that is right for them. | |  | | |  |  | | --- | --- | | |  | | --- | |  | | |  | | | |  |  |  |  |  | | --- | --- | --- | --- | --- | | |  | | --- | | 6.2 \*  Will results of the research be communicated back to the subjects? | |  | | Yes **No** | |  | | |  |  | | --- | --- | | |  | | --- | |  | | |  | |      |  |  |  |  |  |  |  |  |  |  | | --- | --- | --- | --- | --- | --- | --- | --- | --- | --- | | |  |  |  |  |  | | --- | --- | --- | --- | --- | | |  | | --- | | 6.2.1 \*  Explain the plan and process. | |  | |  | |  | | |  |  | | --- | --- | | |  | | --- | |  | | |  | |     |  |  |  |  |  |  |  |  |  |  | | --- | --- | --- | --- | --- | --- | --- | --- | --- | --- | | |  |  |  |  |  | | --- | --- | --- | --- | --- | | |  | | --- | | 6.3 \*  Describe any direct risks to the public or community, which could result from this research? | |  | | None | |  | | |  |  | | --- | --- | | |  | | --- | |  | | |  | |  |  |  |  |  |  |  |  |  |  |  | | --- | --- | --- | --- | --- | --- | --- | --- | --- | --- | | |  |  |  |  |  | | --- | --- | --- | --- | --- | | |  | | --- | | 6.4 \*  Does this project involve study arms that have differing levels of benefit or risks to subjects? | | Yes **No** | |  | |  | | |  |  | | --- | --- | | |  | | --- | |  | | |  | |  |  |  |  |  |  |  |  |  |  |  |  |  |  |  |  |  |  |  |  | | --- | --- | --- | --- | --- | --- | --- | --- | --- | --- | --- | --- | --- | --- | --- | --- | --- | --- | --- | | |  |  |  |  |  |  |  |  |  |  |  |  |  |  | | --- | --- | --- | --- | --- | --- | --- | --- | --- | --- | --- | --- | --- | --- | | |  | | --- | | **6.5 \*  Benefits and Risks:** | | *Click "Add" to begin entering the benefit and risk level detail information associated with this study.* | | |  |  |  |  |  |  |  |  |  | | --- | --- | --- | --- | --- | --- | --- | --- | --- | | |  | Name | Risk Level | Direct Benefit | | --- | --- | --- | --- | | View | HUM00053273 | No more than minimal risk | no | | | |  | | |  |  | | --- | --- | | |  | | --- | |  | | |  | | |
| Benefits and Risk Level Detail  **If a study involves multiple arms or phases that pose different levels of risk or direct benefits to subjects, then create an entry for each arm or phase using the "OK and Add Another" option at the bottom of this page.  Only one entry is necessary if the risk level and the direct benefit to subjects is the same for the entire project, even if the study involves multiple arms or phases.**       |  |  |  |  |  |  |  |  |  |  | | --- | --- | --- | --- | --- | --- | --- | --- | --- | --- | | |  |  |  |  |  | | --- | --- | --- | --- | --- | | |  | | --- | | 6.5.1 \* Name of Arm (experimental group, study wave, etc.) | |  | | HUM00053273 | |  | | |  |  | | --- | --- | | |  | | --- | |  | | |  | |  |  |  |  |  |  |  |  |  |  |  | | --- | --- | --- | --- | --- | --- | --- | --- | --- | --- | | |  |  |  |  |  | | --- | --- | --- | --- | --- | | |  | | --- | | 6.5.2 \*  Description of Arm (experimental group, study wave, etc.) | |  | |  | |  | | |  |  | | --- | --- | | |  | | --- | |  | | |  | |     |  |  |  |  |  |  |  |  |  |  | | --- | --- | --- | --- | --- | --- | --- | --- | --- | --- | | |  |  |  |  |  | | --- | --- | --- | --- | --- | | |  | | --- | | 6.6 \* Are there potential direct benefits of this research to the subjects? | |  | | Yes **No** | |  | | |  |  | | --- | --- | | |  | | --- | |  | | |  | |      |  |  |  |  |  |  |  |  |  |  | | --- | --- | --- | --- | --- | --- | --- | --- | --- | --- | | |  |  |  |  |  | | --- | --- | --- | --- | --- | | |  | | --- | | 6.6.1 \*  Describe the potential direct benefits. | |  | |  | |  | | |  |  | | --- | --- | | |  | | --- | |  | | |  | |     |  |  |  |  |  |  |  |  |  |  | | --- | --- | --- | --- | --- | --- | --- | --- | --- | --- | | |  |  |  |  |  | | --- | --- | --- | --- | --- | | |  | | --- | | 6.7 \*  Provide a description of the foreseeable risks to subjects.  For studies involving multiple arms or phases, enter the risks for this arm or phase only. Provide a description of the foreseeable risks to the subjects.  **For EACH identified risk, include:**  - **Likelihood of the risk,**- **Seriousness to the subject; and**- **What measures will be taken to minimize the risk (for example, study design includes the substitution of procedures already being performed on the subject for diagnostic or treatment purposes, or in a study of Post-Traumatic Stress Disorder, the investigator takes steps to identify, manage, or refer as appropriate, subjects for whom the study may evoke very difficult emotions)**  **If possible, please use the following categories to assess the likelihood:**    - **"Common" (i.e., approximate incidence > 25%)**- **"Likely" (i.e., approximate incidence of 10-25%)**- **"Infrequent" (i.e., approximate incidence of 1-10%)**- **"Rare" (i.e., approximate incidence < 1%):** | | Breach of confidentiality The researchers have implemented multiple systems that will protect confidentiality including research documents housed on secured server, never on a laptop, and secured transmission and storage of audio recordings. Due to these safe guards, the risk of the breach of confidentiality is extremely low. | |  | |  | | |  |  | | --- | --- | | |  | | --- | |  | | |  | | | |  |  |  |  |  | | --- | --- | --- | --- | --- | | |  | | --- | | 6.8 \*  What is the level of risk of harm to the subjects, resulting from this arm of the research?  For studies involving multiple arms or phases, enter the level of risk for this arm or phase only. | | No more than minimal risk | |  | |  | | |  |  | | --- | --- | | |  | | --- | |  | | |  | | | |  |  |  |  |  | | --- | --- | --- | --- | --- | | |  | | --- | | 6.9 \*  Discuss why the risks to the subjects are reasonable in relation to the anticipated benefits. | | The researchers have put in place all the safeguards possible to protect patient confidentiality thus reducing the risk of breach of confindentiality to a minimum in turn for the minimum benefits the particpation may receive for participating in the study. | |  | |  | | |  |  | | --- | --- | | |  | | --- | |  | | |  | | |

|  |  |  |
| --- | --- | --- |
| |  |  | | --- | --- | |  |  | |
| 07. Special Considerations     |  |  |  |  |  |  |  |  |  |  | | --- | --- | --- | --- | --- | --- | --- | --- | --- | --- | | |  |  |  |  |  | | --- | --- | --- | --- | --- | | |  | | --- | | 7.1\*  Does this study involve human tissue or biological specimens (use, collection, or secondary analysis)  (e.g. blood, urine, bone marrow, skin, etc.)?  [Require Section 18] | |  | | Yes **No** | |  | | |  |  | | --- | --- | | |  | | --- | |  | | |  | |      |  |  |  |  |  |  |  |  |  |  | | --- | --- | --- | --- | --- | --- | --- | --- | --- | --- | | |  |  |  |  |  | | --- | --- | --- | --- | --- | | |  | | --- | | 7.1.1\*  Will genetic analysis be performed on any specimens acquired in conjunction with this study?  [Require Section 20] | |  | | Yes No | |  | | |  |  | | --- | --- | | |  | | --- | |  | | |  | |     |  |  |  |  |  |  |  |  |  |  | | --- | --- | --- | --- | --- | --- | --- | --- | --- | --- | | |  |  |  |  |  | | --- | --- | --- | --- | --- | | |  | | --- | | 7.2\*  Does this study involve the secondary analysis of a pre-existing data set, including data associated with any specimens identified in response to question 7.1?  [Require Section 24] | |  | | **Yes** No | |  | | |  |  | | --- | --- | | |  | | --- | |  | | |  | | | |  |  |  |  |  | | --- | --- | --- | --- | --- | | |  | | --- | | 7.3\*  Will the research involve the access, collection, use, maintenance, or disclosure of University of Michigan protected health information (PHI)? PHI is:  - information about a subjects past, present, or future physical or mental health, the provision of healthcare to a subject, or payment for the provision of healthcare to a subject; AND - maintained by a University of Michigan school, department, division, or other unit that is part of the University's HIPAA-covered component (e.g. healthcare provider, healthcare plan, or healthcare clearinghouse).  [Require Section 25] | |  | | Yes **No** | |  | | |  |  | | --- | --- | | |  | | --- | |  | | |  | | |
| 07-1. Special Considerations - Continued     |  |  |  |  |  |  |  |  |  |  | | --- | --- | --- | --- | --- | --- | --- | --- | --- | --- | | |  |  |  |  |  | | --- | --- | --- | --- | --- | | |  | | --- | | 7-1.1\*  Will subjects receive payment or other incentives for their participation in the study? [Require Section 13] | |  | | **Yes** No | |  | | |  |  | | --- | --- | | |  | | --- | |  | | |  | | | |  |  |  |  |  | | --- | --- | --- | --- | --- | | |  | | --- | | 7-1.2\*  Will subjects undergo healthcare-related treatments or procedures (standard of care and/or research) as part of the study? [Require Section 14] | |  | | Yes **No** | |  | | |  |  | | --- | --- | | |  | | --- | |  | | |  | | | |  |  |  |  |  | | --- | --- | --- | --- | --- | | |  | | --- | | 7-1.3\*  Does this study involve the deception of subjects? [Require Section 27] | |  | | Yes **No** | |  | | |  |  | | --- | --- | | |  | | --- | |  | | |  | | | |  |  |  |  |  | | --- | --- | --- | --- | --- | | |  | | --- | | 7-1.4\*  Excluding routine email correspondence, does this study involve the use of the Internet or email as an integral part of the research design or will sensitive information be transmitted by e-mail? [Require Section 28] | |  | | **Yes** No | |  | | |  |  | | --- | --- | | |  | | --- | |  | | |  | | | |  |  |  |  |  | | --- | --- | --- | --- | --- | | |  | | --- | | 7-1.5\*  Will the study collect data using surveys, interviews, or focus groups? [Require Section 29] | |  | | **Yes** No | |  | | |  |  | | --- | --- | | |  | | --- | |  | | |  | | | |  |  |  |  |  | | --- | --- | --- | --- | --- | | |  | | --- | | 7-1.6\*  Does this study require subjects to listen to an audio recording or view images? [Require Section 31] | |  | | Yes **No** | |  | | |  |  | | --- | --- | | |  | | --- | |  | | |  | | | |  |  |  |  | | --- | --- | --- | --- | | |  | | --- | | 7-1.7\*  Will any drugs, biologics, nutritional (e.g., herbal or alternative medication) supplements or other material be administered, implanted, or applied to the subjects as the object of the study? [Require Section 15] | |  | | Yes **No** |    **PLEASE NOTE:** Since drugs are being used in this study, a MiChart order may need to be created; please allow sufficient time for the MiChart orderset to be created before starting your study. | |  | | |  |  | | --- | --- | | |  | | --- | |  | | |  | |
| |  |  |  |  |  | | --- | --- | --- | --- | --- | | |  | | --- | | 7-1.8\*  Will the study involve a placebo (drug, device, procedure, intervention, surgery, etc.) control group? [Require Section 17] | |  | | Yes **No** | |  | | |  |  | | --- | --- | | |  | | --- | |  | | |  | |

|  |  |  |  |  |  |  |  |  |  |
| --- | --- | --- | --- | --- | --- | --- | --- | --- | --- |
| |  |  |  |  |  | | --- | --- | --- | --- | --- | | |  | | --- | | 7-1.8.1\*  Is the placebo for a drug? [Require Section 15] | |  | | Yes No | |  | | |  |  | | --- | --- | | |  | | --- | |  | | |  | |

 

|  |  |  |  |  |  |  |  |  |  |
| --- | --- | --- | --- | --- | --- | --- | --- | --- | --- |
| |  |  |  |  |  | | --- | --- | --- | --- | --- | | |  | | --- | | 7-1.9\*  Will the study involve human embryonic stem cells (hESCs) or induced pluripotent stem cells? [Require Section 19] | |  | | Yes **No** | |  | | |  |  | | --- | --- | | |  | | --- | |  | | |  | |
| |  |  |  |  |  | | --- | --- | --- | --- | --- | | |  | | --- | | 7-1.10\*  Will the study have a Data and Safety Monitoring Plan (DSMP)? **[Require Section 32]** | |  | | **Yes** No | |  | | |  |  | | --- | --- | | |  | | --- | |  | | |  | |

     | 7-2. Special Consideration - Continued     |  |  |  |  |  |  |  |  |  |  | | --- | --- | --- | --- | --- | --- | --- | --- | --- | --- | | |  |  |  |  |  | | --- | --- | --- | --- | --- | | |  | | --- | | 7-2.1\*  Will any devices be used, administered, implanted, or applied to the subjects, or will human specimens be used to test in vitro diagnostic devices? *[Non-IRB HSBS Applications Require Section 16]* | |  | | Yes **No** | |  | | |  |  | | --- | --- | | |  | | --- | |  | | |  | |        |  |  |  |  |  |  |  |  |  |  | | --- | --- | --- | --- | --- | --- | --- | --- | --- | --- | | |  |  |  |  |  | | --- | --- | --- | --- | --- | | |  | | --- | | 7-2.1.1\*  Describe all devices that are the OBJECT of the study, or ARE RELEVANT to the study. If this study is designed to test the safety or efficacy of any of these devices, then this project is FDA-regulated and must be reviewed by IRBMED. | |  | |  | |  | | |  |  | | --- | --- | | |  | | --- | |  | | |  | |      |  |  |  |  |  |  |  |  |  |  | | --- | --- | --- | --- | --- | --- | --- | --- | --- | --- | | |  |  |  |  |  | | --- | --- | --- | --- | --- | | |  | | --- | | 7-2.2\*  Will the subjects be exposed to any ionizing radiation during the course of this study? [Require Section 21] | |  | | Yes **No** | |  | | |  |  | | --- | --- | | |  | | --- | |  | | |  | | | |  |  |  |  |  | | --- | --- | --- | --- | --- | | |  | | --- | | 7-2.3\*  Will any organs, tissues, or cells from other humans (including fetal tissue) or animals be administered to the subjects for the purposes of this study? [Require Section 22] | |  | | Yes **No** | |  | | |  |  | | --- | --- | | |  | | --- | |  | | |  | | | |  |  |  |  |  | | --- | --- | --- | --- | --- | | |  | | --- | | 7-2.4\*  Does this study involve a gene transfer intervention or an intervention based on recombinant DNA technology? [Require Section 23] | |  | | Yes **No** | |  | | |  |  | | --- | --- | | |  | | --- | |  | | |  | | |


|  |  |  |
| --- | --- | --- |
| |  |  | | --- | --- | |  |  | |
| 08. Subject Participation     |  |  |  |  |  |  |  |  |  |  | | --- | --- | --- | --- | --- | --- | --- | --- | --- | --- | | |  |  |  |  |  | | --- | --- | --- | --- | --- | | |  | | --- | | 8.1\*  Please indicate the number of subjects required (from ALL study sources, sites, and locations combined) to achieve the goal of the study (the statistical "n" of the study): | |  | | 650 | |  | | |  |  | | --- | --- | | |  | | --- | |  | | |  | | | |  |  |  |  |  | | --- | --- | --- | --- | --- | | |  | | --- | | 8.1.1\*  Of the number provided in 8.1, indicate the number of subjects from performance sites where UM-related research activities are conducted (see list of sites in 8.2, below). | |  | | 650 | |  | | |  |  | | --- | --- | | |  | | --- | |  | | |  | | | |  |  |  |  |  |  |  |  |  |  |  |  |  |  |  |  |  |  |  |  |  |  |  |  |  |  |  |  |  |  |  |  |  |  |  |  |  |  |  |  |  |  |  |  |  |  |  |  |  |  |  |  |  |  |  |  |  |  |  |  |  |  |  |  |  |  |  |  |  |  |  |  |  |  |  |  |  |  |  |  |  |  |  |  |  |  |  |  |  |  |  |  |  |  |  |  |  | | --- | --- | --- | --- | --- | --- | --- | --- | --- | --- | --- | --- | --- | --- | --- | --- | --- | --- | --- | --- | --- | --- | --- | --- | --- | --- | --- | --- | --- | --- | --- | --- | --- | --- | --- | --- | --- | --- | --- | --- | --- | --- | --- | --- | --- | --- | --- | --- | --- | --- | --- | --- | --- | --- | --- | --- | --- | --- | --- | --- | --- | --- | --- | --- | --- | --- | --- | --- | --- | --- | --- | --- | --- | --- | --- | --- | --- | --- | --- | --- | --- | --- | --- | --- | --- | --- | --- | --- | --- | --- | --- | --- | --- | --- | --- | --- | --- | | |  | | --- | | 8.2\*  Enter the estimated number of subjects required from each site where UM-related activities are to be conducted over all of the years of the study. | |  | | | Location Or Institution | Total | | --- | --- | | **George C. Hawrot, MD** |  | | Adults | 15 | | Children | 0 | | **David Williams, MD PC** |  | | Adults | 40 | | Children | 0 | | **Lonnie Joe Jr., MD** |  | | Adults | 15 | | Children | 0 | | **Dua Family Practice** |  | | Adults | 35 | | Children | 0 | | **Mark W. Sawka, MD** |  | | Adults | 50 | | Children | 0 | | **Downriver Internists PC** |  | | Adults | 60 | | Children | 0 | | **Morang Chester Clinic PC** |  | | Adults | 60 | | Children | 0 | | **University of Michigan Family Medicine Clinics** |  | | Adults | 140 | | Children | 0 | | **up to 10 yet to be recruited practices in the Metro Detroit area** |  | | Adults | 20 | | Children | 0 | | **Michigan State university** |  | | Adults | 0 | | Children | 0 | | **Andrew Thomas, MD PC** |  | | Adults | 50 | | Children | 0 | | **IPC-Livonia Internal Medicine** |  | | Adults | 60 | | Children | 0 | | **Rice Lanzilote Dos and Egan MD** |  | | Adults | 50 | | Children | 0 | | **Children & Family Medical Clinic** |  | | Adults | 35 | | Children | 0 | | **University of Michigan** |  | | Adults | 20 | | Children | 0 | | |  | | |  |  | | --- | --- | | |  | | --- | |  | | | Additional Help | | |
| 08-1. Subject Recruitment     |  |  |  |  |  |  |  |  |  |  | | --- | --- | --- | --- | --- | --- | --- | --- | --- | --- | | |  |  |  |  |  | | --- | --- | --- | --- | --- | | |  | | --- | | 8-1.1\*  At what point in the study are you planning on beginning the recruitment of subjects? | |  | | 0-2 years after approval | |  | | |  |  | | --- | --- | | |  | | --- | |  | | |  | | | |  |  |  |  |  |  |  |  |  | | --- | --- | --- | --- | --- | --- | --- | --- | --- | | |  | | --- | | 8-1.2\*  Indicate which of the following established subject pools, if any, will be used for recruitment. | |  | | |  |  |  |  | | --- | --- | --- | --- | | | Select all that apply: | | --- | | UM Human Research Recruiting Registry (e.g., UMClinical Studies - http://www.UMClinicalStudies.org) | | Patients of non-UM physicians, dentists, or other health care providers or facilities | |   Provide Related UM IRB Project Number or Subject Pool Description: | |  | | |  |  | | --- | --- | | |  | | --- | |  | | |  | | | |  |  |  |  |  | | --- | --- | --- | --- | --- | | |  | | --- | | 8-1.3\*  Describe the manner in which potential study subjects will be recruited. List how, when, who will recruit and where they will be recruited. Include any provisions to protect or maintain subject privacy. | |  | | Using Umclinicalstudies.org and by staff invitation for Phase I   For Phase 2, practices will identify patients meeting eligibility criteria. The patient list will be electronically transferred to a secure server and will be password protected.  For supplement recruitment, participants meeting eligibility criteria will be identified prior to calling them for their 6-month follow-up. If they are interested in participating their names will be forwarded to Dr. Kelly-Blake at MSU. | |  | | |  |  | | --- | --- | | |  | | --- | |  | | | Additional Help | | | |  |  |  |  |  | | --- | --- | --- | --- | --- | | |  | | --- | | 8-1.3.1  If applicable, how will prospective subjects' healthcare providers (e.g., physician, dentist, etc.) be involved in the recruitment and/or be notified of their individual patients' participation in the study? | |  | | After a patient list with eligible patients is compiled by the practice, the practice physician will review and remove any patients they do not want to be included in the study | |  | | |  |  | | --- | --- | | |  | | --- | |  | | |  | | | |  |  |  |  |  | | --- | --- | --- | --- | --- | | |  | | --- | | 8-1.4\*  Explain how the recruitment strategy is equitable and represents the population required for the study.  If the information is covered in the attached protocol, please indicate section. | |  | | For Phase 1 of the study, Umclinicalstudies.org is open to the general public and the participants accrued for Usability Testing all have to meet the same eligibility requirements as the participants of the main study.   For Phase 2, any patients meeting study eligibility and not removed by their physician are eligible. Practices have been recruited, base on their racial make up, with the intent of meeting our recruitment goals.  As the objective of the supplement is to explore African American patients’ perceptions and concerns about the use and acceptability of IT within and outside the clinical encounters, only African Americans will be recruited | |  | | |  |  | | --- | --- | | |  | | --- | |  | | |  | | | |  |  |  |  |  | | --- | --- | --- | --- | --- | | |  | | --- | | 8-1.5\*  Does the recruitment strategy involve contacting individuals multiple times in an effort to secure their initial enrollment into the study? | |  | | **Yes** No | |  | | |  |  | | --- | --- | | |  | | --- | |  | | |  | |      |  |  |  |  |  |  |  |  |  |  | | --- | --- | --- | --- | --- | --- | --- | --- | --- | --- | | |  |  |  |  |  | | --- | --- | --- | --- | --- | | |  | | --- | | 8-1.5.1\*  Describe how frequently and in what manner individuals will be contacted.  If the information is covered in the attached protocol, please indicate section. | |  | | For Phase 1, interested participants from Umclinicalstudies.org will be attemped to be contacted three times via phone or email.  For Phase 2, eligible patients will be sent an invitation to participate letter from their physician by the study staff along with a study brochure. They will have the option to phone an 800 number or email to opt-out or out-in. If they do nothing they will be called 3-5 times to determine their interest.  For the Supplement, eligible participants will only be asked once during the 6-month follow-up phone call if they are interested in being contacted. | |  | | |  |  | | --- | --- | | |  | | --- | |  | | |  | |     |  |  |  |  |  |  |  |  |  |  |  |  |  |  |  |  |  | | --- | --- | --- | --- | --- | --- | --- | --- | --- | --- | --- | --- | --- | --- | --- | --- | --- | | |  |  |  |  |  |  |  |  |  |  |  |  | | --- | --- | --- | --- | --- | --- | --- | --- | --- | --- | --- | --- | | |  | | --- | | 8-1.6\*  Indicate which methods will be used for recruitment? | |  | | |  |  |  |  |  |  |  | | --- | --- | --- | --- | --- | --- | --- | | | Check all that apply: | | --- | | Email | | Postal mail | | Telephone | | Posting on UMClinicalStudies.org website (formerly ENGAGE) | | Other | |   If other please specify:  Staff invitation for Phase 1 | |  | | |  |  | | --- | --- | | |  | | --- | |  | | |  | | | |  |  |  |  |  | | --- | --- | --- | --- | --- | | |  | | --- | | 8-1.7  How will any email, address, and/or telephone lists be obtained? | |  | | For Phase 1, this information will be obtained from the volunteered information on Umclinicalstudies.org.  For Phase 2, address and telephone numbers will be obtained from the practices. Email addresses will be obtained from participants who chose to share them. Primary contact with participants will not be by email, but if they choose to contact the research team email is an option for them. | |  | | |  |  | | --- | --- | | |  | | --- | |  | | |  | | | |  |  |  |  |  |  |  |  |  |  |  |  |  |  |  |  |  |  |  |  |  |  |  |  |  |  |  |  |  |  |  |  |  |  |  |  |  |  |  |  |  |  |  |  |  |  |  |  |  |  |  |  |  |  |  |  |  |  |  |  |  |  |  |  |  |  |  |  |  |  |  |  |  |  |  |  |  |  |  |  |  |  |  |  |  |  | | --- | --- | --- | --- | --- | --- | --- | --- | --- | --- | --- | --- | --- | --- | --- | --- | --- | --- | --- | --- | --- | --- | --- | --- | --- | --- | --- | --- | --- | --- | --- | --- | --- | --- | --- | --- | --- | --- | --- | --- | --- | --- | --- | --- | --- | --- | --- | --- | --- | --- | --- | --- | --- | --- | --- | --- | --- | --- | --- | --- | --- | --- | --- | --- | --- | --- | --- | --- | --- | --- | --- | --- | --- | --- | --- | --- | --- | --- | --- | --- | --- | --- | --- | --- | --- | --- | | |  | | --- | | 8-1.8\*  What materials will be used for recruitment? The IRB must approve all recruitment materials. See Help for important information regarding the requirements for recruitment materials | |  | | |  |  |  |  |  |  | | --- | --- | --- | --- | --- | --- | | | Check all that apply: | | --- | | Pre-screening questions | | Flyers | | Oral scripts | | Letters/postal mail | |   If other please specify:   If Web pages will be used, provide the Web address (URL) for the location where the pages will be posted (also upload the content of the pages below):   Upload recruitment materials here:  See Help for more information about working with documents (e.g. uploading, downloading, and editing).    |  |  |  |  |  |  |  |  |  |  |  |  |  |  |  |  |  |  |  |  |  |  |  |  |  |  |  |  |  |  |  |  |  |  |  |  |  |  |  |  |  |  |  |  |  |  |  |  |  |  |  |  |  |  |  |  |  |  |  |  |  |  |  |  |  |  |  |  |  |  |  |  |  |  |  | | --- | --- | --- | --- | --- | --- | --- | --- | --- | --- | --- | --- | --- | --- | --- | --- | --- | --- | --- | --- | --- | --- | --- | --- | --- | --- | --- | --- | --- | --- | --- | --- | --- | --- | --- | --- | --- | --- | --- | --- | --- | --- | --- | --- | --- | --- | --- | --- | --- | --- | --- | --- | --- | --- | --- | --- | --- | --- | --- | --- | --- | --- | --- | --- | --- | --- | --- | --- | --- | --- | --- | --- | --- | --- | --- | | | Name | Version | | --- | --- | | 3X5 flyer | History | 0.01 | | Clean DATES flyer v.7.docx | History | 0.04 | | Clean Livonia Invite letter v-4.3.docx | History | 0.05 | | CLEAN revised invite letter v.4.3 | History | 0.05 | | Clean Screening script 2.21.12.docx | History | 0.03 | | clean waiting rm brochure | History | 0.01 | | CLEAN\_Screening survey\_ v.1.7\_6.4.13.edits.docx | History | 0.08 | | CLEAN\_UnknowApptInviteLetter\_v.4.2.2.docx | History | 0.02 | | CLEAN2\_FlyerBalckFemaleDr.docx | History | 0.02 | | CLEAN2\_FlyerHispanicFemaleDr.docx | History | 0.02 | | CLEAN2\_FlyerOlderWhiteMaleDr2.docx | History | 0.03 | | CLEAN2\_FlyerWhiteFemaleDr.docx | History | 0.02 | | CLEAN2\_FlyerWhiteFemaleDr2.docx | History | 0.02 | | CLEAN2\_FlyerYoungWhiteFemaleDr.docx | History | 0.02 | | CLEAN3\_FlyerOlderWhiteMaleDr.docx | History | 0.03 | | DATES flyer general info.docx | History | 0.01 | | FINAL\_Brochure\_BlackFemaleDr.docx | History | 0.01 | | FINAL\_Brochure\_HispanicFemaleDr.docx | History | 0.01 | | FINAL\_Brochure\_Nikki.docx | History | 0.01 | | FINAL\_Brochure\_OlderWhiteMaleDr.docx | History | 0.01 | | FINAL\_Brochure\_WhiteFemaleDr.docx | History | 0.01 | | FINAL\_Brochure-1.Blue.9.5.12.docx | History | 0.01 | | FINAL2\_Brochure\_OlderWhiteMaleDr2.docx | History | 0.02 | | hands flyer 4/page | History | 0.01 | | invite brochure v.5.docx | History | 0.03 | | RA Supplement Phone Script | History | 0.01 | | Track\_FlyerBalckFemaleDr.docx | History | 0.01 | | TRACK\_FlyerHispanicFemaleDr.docx | History | 0.01 | | TRACK\_Screening survey\_ v.1.7\_6.4.13.edits.docx | History | 0.06 | | TRACK\_UnknowApptInviteLetter\_v.4.2.2.docx | History | 0.02 | | Tracked DATES flyers v.7\_2.21.12.docx | History | 0.03 | | tracked invite brochure v.5 | History | 0.02 | | TRACKED Livonia Invite letter v-4.3.docx | History | 0.05 | | Tracked revised invite letter v.4.3. | History | 0.04 | | tracked waiting rm brochure | History | 0.01 | | UMClinicalStudiesAd\_v.2 | History | 0.02 | |    **Check here if any of the materials are not available electronically.**  **Note:** Study Teams are encouraged to scan and upload documents. See Help for a list of sites with scanning facilities | |  | | |  |  | | --- | --- | | |  | | --- | |  | | | Additional Help | | |

|  |  |  |
| --- | --- | --- |
| |  |  | | --- | --- | |  |  | |
| 09. Survey Populations     |  |  |  |  |  |  |  |  |  |  | | --- | --- | --- | --- | --- | --- | --- | --- | --- | --- | | |  |  |  |  |  | | --- | --- | --- | --- | --- | | |  | | --- | | 9.1\*  Is the study limited to a survey of either:  - The general adult population (aged 18 or older); or- A subgroup of the general population which does not specifically target:  - Pregnant women and/or fetuses- Lactating women- Women of child-bearing potential- Prisoners- Cognitively impaired adults- College students- Economically or educationally disadvantaged persons- Patients of the study team- Employees, students or trainees of the study team- Family members of the study team  where the survey is the sole interaction with the subject and does not pose more than minimal risk? | |  | | **Yes** No | |  | | |  |  | | --- | --- | | |  | | --- | |  | | |  | | |

|  |  |  |
| --- | --- | --- |
| |  |  | | --- | --- | |  |  | |
| 10. Informed Consent - Adults     |  |  |  |  |  |  |  |  |  |  |  |  |  |  |  | | --- | --- | --- | --- | --- | --- | --- | --- | --- | --- | --- | --- | --- | --- | --- | | |  |  |  |  |  |  |  |  |  |  | | --- | --- | --- | --- | --- | --- | --- | --- | --- | --- | | |  | | --- | | 10.1\*  What type of informed consent will be obtained from adults or minors legally able to consent to treatments or procedures involved in the research? | |  | | |  |  |  |  |  | | --- | --- | --- | --- | --- | | | Select all that apply: | | --- | | Comprehensive written | | Request for waiver of documentation of informed consent | | Request for waiver of informed consent/parental permission/legally authorized representative consent | | | |  | | |  |  | | --- | --- | | |  | | --- | |  | | | Additional Help | |      |  |  |  |  |  |  |  |  |  |  |  |  |  |  |  |  |  |  |  |  |  |  | | --- | --- | --- | --- | --- | --- | --- | --- | --- | --- | --- | --- | --- | --- | --- | --- | --- | --- | --- | --- | --- | --- | | |  |  |  |  |  |  |  |  |  |  |  |  |  |  |  |  |  | | --- | --- | --- | --- | --- | --- | --- | --- | --- | --- | --- | --- | --- | --- | --- | --- | --- | | |  | | --- | | 10.1.1\*  Waiver of assent is requested because: | |  | | |  |  |  |  |  |  |  |  |  |  |  |  | | --- | --- | --- | --- | --- | --- | --- | --- | --- | --- | --- | --- | | | Select all that apply: | | --- | |  |  |  |  |  |  |  |  |  |  | | --- | --- | --- | --- | --- | --- | --- | --- | --- | --- | | There are no items to display | | | | | | | | | | | | |  | | |  |  | | --- | --- | | |  | | --- | |  | | |  | |     |  |  |  |  |  |  |  |  |  |  | | --- | --- | --- | --- | --- | --- | --- | --- | --- | --- | | |  |  |  |  |  | | --- | --- | --- | --- | --- | | |  | | --- | | 10.1.2\*  Describe the process to seek and obtain informed consent and/or assent from adults. If requesting a waiver of documentation of assent, provide justification here. | |  | | Eigible participants will be consented at the beginning of the research visit. The research coordinator will address any questions or concerns raised by the Phase 1 or Phase 2 patients. In Phase 2, the patient will first be given the choice of consenting to be audio recorded or not. If they don't want to be recorded they will not be eligible to participate and are done. If they agree to be audio recorded, they must also chose to participate in the study. If they choose not to participate, they are done. Participant will be given a hard (paper) copy of the consent. | |  | | |  |  | | --- | --- | | |  | | --- | |  | | | Additional Help | | | |  |  |  |  |  | | --- | --- | --- | --- | --- | | |  | | --- | | 10.1.3\*  Is the cognitive capacity of the subjects expected to change significantly during the study? | |  | | Yes **No** | |  | | |  |  | | --- | --- | | |  | | --- | |  | | |  | |      |  |  |  |  |  |  |  |  |  |  | | --- | --- | --- | --- | --- | --- | --- | --- | --- | --- | | |  |  |  |  |  | | --- | --- | --- | --- | --- | | |  | | --- | | 10.1.3.1\*  Describe the plan to re-consent the subject or the subject’s legally authorized representative after the change in the cognitive capacity of the subject. | |  | |  | |  | | |  |  | | --- | --- | | |  | | --- | |  | | | Additional Help | | |
| 10-1. Informed Consent     |  |  |  |  |  |  |  |  |  |  |  |  |  |  |  |  |  |  |  |  |  |  |  |  |  | | --- | --- | --- | --- | --- | --- | --- | --- | --- | --- | --- | --- | --- | --- | --- | --- | --- | --- | --- | --- | --- | --- | --- | --- | --- | | |  |  |  |  |  |  |  |  |  |  |  |  |  |  |  |  |  |  |  |  | | --- | --- | --- | --- | --- | --- | --- | --- | --- | --- | --- | --- | --- | --- | --- | --- | --- | --- | --- | --- | | |  | | --- | | 10-1.1\*  All documents related to consent, assent, permission, and or debriefing documents, including oral scripts must be uploaded here. If you are requesting a waiver of documentation of informed consent, upload a copy of any written materials to be provided to participants, and provide a written description of any information to be provided orally. | |  | | |  |  |  |  |  |  |  |  |  |  |  |  |  |  |  | | --- | --- | --- | --- | --- | --- | --- | --- | --- | --- | --- | --- | --- | --- | --- | | | Name | Version | | --- | --- | | CLEAN\_PatientConsent\_v.10\_5.22.13.docx | History | 0.15 | | CLEAN\_PhysicianConsent\_v.9\_5.22.13.edit. 7.30.13docx.docx | History | 0.15 | | NO LONGER IN USE\_Usability IC v.3\_2.2.12.docx | History | 0.05 | | Supplement Verbal Consent | History | 0.01 | | TRACK\_PatientConsent\_v.10\_5.22.13.docx | History | 0.17 | | TRACK\_PhysicianConsent\_v.9\_5.22.13.edit. 7.30.13docx.docx | History | 0.16 | | | |  | | |  |  | | --- | --- | | |  | | --- | |  | | | Additional Help | | | |  |  |  |  |  | | --- | --- | --- | --- | --- | | |  | | --- | | 10-1.2\*  Will the subjects be audiotaped, videotaped, or photographed (identifiable images of subject) during the research? | |  | | **Yes** No | |  | | |  |  | | --- | --- | | |  | | --- | |  | | | Additional Help | | | |  |  |  |  |  | | --- | --- | --- | --- | --- | | |  | | --- | | 10-1.3\*  Is there a substantial likelihood that the research will be conducted among a non-English-speaking population? | |  | | Yes **No** | |  | | |  |  | | --- | --- | | |  | | --- | |  | | | Additional Help | |      |  |  |  |  |  |  |  |  |  |  |  |  |  |  |  |  |  |  |  |  |  |  |  | | --- | --- | --- | --- | --- | --- | --- | --- | --- | --- | --- | --- | --- | --- | --- | --- | --- | --- | --- | --- | --- | --- | --- | | |  |  |  |  |  |  |  |  |  |  |  |  |  |  |  |  |  |  | | --- | --- | --- | --- | --- | --- | --- | --- | --- | --- | --- | --- | --- | --- | --- | --- | --- | --- | | |  | | --- | | 10-1.3.1\*  Identify the language(s) expected to be encountered: | |  | | |  |  |  |  |  |  |  |  |  |  |  |  |  | | --- | --- | --- | --- | --- | --- | --- | --- | --- | --- | --- | --- | --- | | | Language | Translation Detail | | --- | --- | |  |  |  |  |  |  |  |  |  |  | | --- | --- | --- | --- | --- | --- | --- | --- | --- | --- | | There are no items to display | | | | | | | | | | | | |  | | |  |  | | --- | --- | | |  | | --- | |  | | |  | |     |  |  |  |  |  |  |  |  |  |  |  |  |  | | --- | --- | --- | --- | --- | --- | --- | --- | --- | --- | --- | --- | --- | | |  |  |  |  |  |  |  |  | | --- | --- | --- | --- | --- | --- | --- | --- | | |  | | --- | | 10-1.4\*  Indicate which anticipated costs could be the full or partial responsibility of the subject. | |  | | |  |  |  | | --- | --- | --- | | | Check all that apply: | | --- | | No anticipated costs | |  **If other, please specify:** | |  | | |  |  | | --- | --- | | |  | | --- | |  | | |  | | | |  |  |  |  |  | | --- | --- | --- | --- | --- | | |  | | --- | | 10-1.5\*  Is the study designed to collect identifiable information from primary research subjects about other individuals, including family members? | |  | | Yes **No** | |  | | |  |  | | --- | --- | | |  | | --- | |  | | | Additional Help | | |
| 10-3. Informed Consent Waiver     |  |  |  |  |  |  |  |  |  |  |  |  |  | | --- | --- | --- | --- | --- | --- | --- | --- | --- | --- | --- | --- | --- | | |  |  |  |  |  |  |  |  | | --- | --- | --- | --- | --- | --- | --- | --- | | |  | | --- | | 10-3.1\*  This request is for: | |  | | |  |  |  | | --- | --- | --- | | | Select all that apply: | | --- | | Waiver of informed consent for PART of the project (Note: Applicable only to the recruitment aspects of the study if the study is subject to FDA oversight) | | | |  | | |  |  | | --- | --- | | |  | | --- | |  | | |  | | | |  |  |  |  |  | | --- | --- | --- | --- | --- | | |  | | --- | | 10-3.1.1  If this request is for PART of the project, identify the specific research procedures (e.g., screening interview) and/or the specific subject populations (e.g., parents of child-subjects) involved. | |  | | Screening | |  | | |  |  | | --- | --- | | |  | | --- | |  | | |  | | | |  |  |  |  |  | | --- | --- | --- | --- | --- | | |  | | --- | | 10-3.1.2  Explain any requested alterations to the informed consent process. | |  | | determine eligibility before consenting | |  | | |  |  | | --- | --- | | |  | | --- | |  | | |  | | | |  |  |  |  |  | | --- | --- | --- | --- | --- | | |  | | --- | | 10-3.2\*  Check below to affirm that this study meets each of the following four criteria for waiver or alteration of informed consent and explain how: | |  | | The research involves no more than minimal risk to the subjects.  **Explain:** Participants will be reviewing a website to determine a method for colon cancer screening. No medical intervention is done. The screening allows the research team to determine eligibility without having the patient show up 90 minutes early for a scheduled appt with their own physician only to determine in 5 minutes that it was unecessary.   The waiver or alteration will not adversely affect the rights and welfare of the subjects.  **Explain:** Participants will simply be screened for eligibility criteria and interest in participating. They will not be denied services.   Research could not practicably (i.e., feasibly) be carried out without the waiver or alteration.  **Explain:** Patients are recruited to engage in the study on a date they are already scheduled with their physician for a HME or chronic care visit. The study intervention needs to occur before their appointment with their clinician. (Without contacting patients prior to their scheduled doctor's appointments to confirm possible eligibility,discuss study participation and the need for them to arrive 90 minutes prior to their appointment to participate in the study, we would not be able to follow the study protocol.) We cannot expect people to show up 90 min early for their doctor's appointments to participate in the DATES Study without knowledge of their eligibility.   Whenever appropriate, the subjects will be provided with additional pertinent information after participation.  **Explain:** Although the intervention deals with making a decision about a screening method which they will discuss with their physician and making a final decision before leaving their appointment, we will provide participants with any new and pertinent information pertaining to their participation. | |  | | |  |  | | --- | --- | | |  | | --- | |  | | | Additional Help | | |
| 10-4. Informed Consent Documentation Waiver     |  |  |  |  |  |  |  |  |  |  |  |  |  | | --- | --- | --- | --- | --- | --- | --- | --- | --- | --- | --- | --- | --- | | |  |  |  |  |  |  |  |  | | --- | --- | --- | --- | --- | --- | --- | --- | | |  | | --- | | 10-4.1\*  This is a request for a waiver of documentation of informed consent for the following reason: | |  | | |  |  |  | | --- | --- | --- | | | Select at least one: | | --- | | The research presents no more than minimal risk of harm to the subject and involves no procedures for which written consent is normally required outside of the research context. | | | |  | | |  |  | | --- | --- | | |  | | --- | |  | | |  | | | |  |  |  |  |  | | --- | --- | --- | --- | --- | | |  | | --- | | 10-4.2\*  Is this a request for a waiver of documentation of informed consent for all research procedures and all subject populations? | |  | | Yes **No** | |  | | |  |  | | --- | --- | | |  | | --- | |  | | |  | |      |  |  |  |  |  |  |  |  |  |  | | --- | --- | --- | --- | --- | --- | --- | --- | --- | --- | | |  |  |  |  |  | | --- | --- | --- | --- | --- | | |  | | --- | | 10-4.2.1\*  Identify the specific research procedures (e.g., screening interview) and/or the specific subject populations (e.g., parents of child subjects) for which a waiver of documentation is being requested. | |  | | screening interview and electronic consent for Usability and Phase 2  In addition, a waiver is being requested for the minority supplement activity, the 12 participants selected to participate will give verbal consent to participate when contacted by phone. | |  | | |  |  | | --- | --- | | |  | | --- | |  | | |  | | |

|  |  |  |
| --- | --- | --- |
| |  |  | | --- | --- | |  |  | |
| 11. Confidentiality/Security/Privacy     |  |  |  |  |  |  |  |  |  |  | | --- | --- | --- | --- | --- | --- | --- | --- | --- | --- | | |  |  |  |  |  | | --- | --- | --- | --- | --- | | |  | | --- | | 11.1\*  Will the study team access any data that is linked to a subject's identity by name or other identifier or code? [Require Section 11-1] | |  | | **Yes** No | |  | | |  |  | | --- | --- | | |  | | --- | |  | | | Additional Help | | | |  |  |  |  |  | | --- | --- | --- | --- | --- | | |  | | --- | | 11.2\*  Explain how the subjects' privacy will be protected. | |  | | Participant will be assigned a study number and their idenitfiable information will be link to the study number. Only research staff will have access to password-protected information. | |  | | |  |  | | --- | --- | | |  | | --- | |  | | | Additional Help | | | |  |  |  |  |  |  |  |  |  |  |  |  |  |  |  |  |  | | --- | --- | --- | --- | --- | --- | --- | --- | --- | --- | --- | --- | --- | --- | --- | --- | --- | | |  | | --- | | 11.3\*  How will the research records, data and/or specimens be protected against inappropriate use or disclosure, or malicious or accidental loss or destruction in order to protect the confidentiality of subject data? | |  | | |  |  |  |  |  |  |  |  |  |  |  |  | | --- | --- | --- | --- | --- | --- | --- | --- | --- | --- | --- | --- | | | Select all that apply: | | --- | | Locked office | | Restricted access | | Access rights terminated when authorized users leave the project or unit | | Secure laptop | | Individual ID plus password protection | | Routine electronic back up | | Encryption of digital data | | No non-UM devices are used to access project data, or any that are used to access project data use secure connections to communicate with U-M services (e.g. VPN – “virtual private network”) | | Security software (firewall, anti-virus, anti-intrusion) is installed and regularly updated on all servers, workstations, laptops, and other devices used in the project | | Safe disposition/destruction of data or devices, as appropriate (e.g., shredding paper documents, destroying disks or thumb drives, secure erasure of electronic media) | |    **If other please specify:** | |  | | |  |  | | --- | --- | | |  | | --- | |  | | |  | | | |  |  | | --- | --- | | |  | | --- | | 11.4\*  Will the research generate information that, if revealed, might place the subjects at risk of personal safety, criminal or civil liability, or damage to their financial standing, employability, or reputation [Require Section 11-2] | | | |
|  |
| Yes **No** |

  | |  |  | | --- | --- | | |  | | --- | |  | | |  | || |  |  |  |  |  | | --- | --- | --- | --- | --- | | |  | | --- | | 11.5\*  Will data be provided to a repository as part of a data sharing agreement? | |  | | Yes **No** | |  | | |  |  | | --- | --- | | |  | | --- | |  | | |  | |

|  |  |  |  |  |  |  |  |  |  |  |  |  |  |  |  |  |  |  |  |  |  |
| --- | --- | --- | --- | --- | --- | --- | --- | --- | --- | --- | --- | --- | --- | --- | --- | --- | --- | --- | --- | --- | --- |
| |  |  |  |  |  |  |  |  |  |  |  |  |  |  |  |  |  | | --- | --- | --- | --- | --- | --- | --- | --- | --- | --- | --- | --- | --- | --- | --- | --- | --- | | |  | | --- | | 11.5.1\*  Please indicate the repository: | |  | | |  |  |  |  |  |  |  |  |  |  |  |  | | --- | --- | --- | --- | --- | --- | --- | --- | --- | --- | --- | --- | | | Select all that apply: | | --- | |  |  |  |  |  |  |  |  |  |  | | --- | --- | --- | --- | --- | --- | --- | --- | --- | --- | | There are no items to display | | | | | | | | | | |    **If Other, please specify:** | |  | | |  |  | | --- | --- | | |  | | --- | |  | | |  | |

 

|  |  |  |  |  |  |  |  |  |  |  |  |  |
| --- | --- | --- | --- | --- | --- | --- | --- | --- | --- | --- | --- | --- |
| |  |  |  |  |  |  |  |  | | --- | --- | --- | --- | --- | --- | --- | --- | | |  | | --- | | 11.6\*  What will happen to the data and/or any specimens at the conclusion of this study? | |  | | |  |  |  | | --- | --- | --- | | | Select all that apply: | | --- | | Retain for study recordkeeping purposes | | | |  | | |  |  | | --- | --- | | |  | | --- | |  | | |  | |

|  |  |  |  |  |  |  |  |  |  |
| --- | --- | --- | --- | --- | --- | --- | --- | --- | --- |
| |  |  |  |  |  | | --- | --- | --- | --- | --- | | |  | | --- | | 11.6.1\*  If the data and/or specimens will be destroyed, describe the specific plan that will be employed following the required retention period. | |  | |  | |  | | |  |  | | --- | --- | | |  | | --- | |  | | |  | |

 

|  |  |  |  |  |  |  |  |  |  |
| --- | --- | --- | --- | --- | --- | --- | --- | --- | --- |
| |  |  |  |  |  | | --- | --- | --- | --- | --- | | |  | | --- | | 11.6.2\*  If the data and/or specimens will be retained for study recordkeeping purposes, provide the following information (if covered in the attached protocol, please indicate section):   - expected duration of the retention period,- any changes in the conditions or arrangements for storage of research data/specimens during the retention period, if different from those listed above in question 11.3. | |  | | Expected retention will be 7 years as required by Federal Regualtions | |  | | |  |  | | --- | --- | | |  | | --- | |  | | |  | |

      | 11-1. Identifiable Data  **Completion of this section is required based on the response provided to question 11.1.**   |  |  |  |  |  |  |  |  |  |  |  |  |  | | --- | --- | --- | --- | --- | --- | --- | --- | --- | --- | --- | --- | --- | | |  |  |  |  |  |  |  |  | | --- | --- | --- | --- | --- | --- | --- | --- | | |  | | --- | | 11-1.1\*  Indicate how subjects are identified in the research records. | |  | | |  |  |  | | --- | --- | --- | | | Select all that apply: | | --- | | Indirectly -- linked to data record but stored separately (e.g., name, initials, phone number, SSN, or medical record number linked to data record but stored separately) | | | |  | | |  |  | | --- | --- | | |  | | --- | |  | | |  | | | |  |  |  |  |  | | --- | --- | --- | --- | --- | | |  | | --- | | 11-1.2\*  Explain the necessity for collecting or maintaining data linked to subjects' identities. If the information is covered in the attached protocol, please indicate section. | |  | | The need to retain data linked to subjects' identities is described in the medical record audit section of the protocol. | |  | | |  |  | | --- | --- | | |  | | --- | |  | | |  | | | |  |  |  |  |  | | --- | --- | --- | --- | --- | | |  | | --- | | 11-1.3\*  How long will the identifiers be retained? | |  | | Until data set is cleaned. | |  | | |  |  | | --- | --- | | |  | | --- | |  | | |  | | | |  |  |  |  |  | | --- | --- | --- | --- | --- | | |  | | --- | | 11-1.4\*  Will individually identifiable sensitive data be accessed, collected, used, maintained, or disclosed in the study? | |  | | Yes **No** | |  | | |  |  | | --- | --- | | |  | | --- | |  | | |  | |      |  |  |  |  |  |  |  |  |  |  | | --- | --- | --- | --- | --- | --- | --- | --- | --- | --- | | |  |  |  |  |  | | --- | --- | --- | --- | --- | | |  | | --- | | 11-1.4.1\*  Will a continuous, periodic, or automatic feed of sensitive data be set up to provide data directly from any University information system (e.g., M-Pathways, U-M Data Warehouse, CareWeb)? | |  | | Yes No | |  | | |  |  | | --- | --- | | |  | | --- | |  | | |  | | | |  |  |  |  |  | | --- | --- | --- | --- | --- | | |  | | --- | | 11-1.4.2\*  Will sensitive data be accessed by individuals who are not University employees? | |  | | Yes No | |  | | |  |  | | --- | --- | | |  | | --- | |  | | |  | | | |  |  |  |  |  | | --- | --- | --- | --- | --- | | |  | | --- | | 11-1.4.3\*  Will sensitive data be stored on or accessed from computer equipment that is not maintained and supported by a University IT services provider (e.g., ITS, MCIT, MSIS) - such as home computers, grant-funded computers, etc.? | |  | | Yes No | |  | | |  |  | | --- | --- | | |  | | --- | |  | | |  | | | |  |  |  |  |  | | --- | --- | --- | --- | --- | | |  | | --- | | 11-1.4.4\*  Will sensitive data be stored on portable devices (e.g., laptops, PDAs, flash drives) in unencrypted form? | |  | | Yes No | |  | | |  |  | | --- | --- | | |  | | --- | |  | | |  | | |
| 11-3. End of Subject Participation     |  |  |  |  |  |  |  |  |  |  | | --- | --- | --- | --- | --- | --- | --- | --- | --- | --- | | |  |  |  |  |  | | --- | --- | --- | --- | --- | | |  | | --- | | 11-3.1\*  What specific criteria will be used to prematurely end a particular subject's participation in the study  (If covered in attached protocol or informed consent, indicate specific location). | |  | | Participant's refusal to complete surveys, view website, or give feedback in Phase 1.  In Phase 2: refusal to complete surveys, view website or be audio recorded. | |  | | |  |  | | --- | --- | | |  | | --- | |  | | |  | | | |  |  |  |  |  | | --- | --- | --- | --- | --- | | |  | | --- | | 11-3.2\*  If a participant withdraws from the research, what is the plan to use, disclose, store, or destroy the participant's data and/or specimen? | |  | | The data up to the point of withdrawal will be used. | |  | | |  |  | | --- | --- | | |  | | --- | |  | | |  | | |


|  |  |  |
| --- | --- | --- |
| |  |  | | --- | --- | |  |  | |
| 13. Subject Payments Or Other Incentives  **Completion of this section is required based on the response provided to question 7-1.1 or 7-3.3.**   |  |  |  |  |  |  |  |  |  |  |  |  |  |  | | --- | --- | --- | --- | --- | --- | --- | --- | --- | --- | --- | --- | --- | --- | | |  |  |  |  |  |  |  |  |  | | --- | --- | --- | --- | --- | --- | --- | --- | --- | | |  | | --- | | 13.1\*  Indicate all payments or other incentives provided to subjects for their participation in this study: | |  | | |  |  |  |  | | --- | --- | --- | --- | | | Select all that apply: | | --- | | Cash | | Other | |    **If other, please specify:**  Meijer gift card | |  | | |  |  | | --- | --- | | |  | | --- | |  | | |  | | | |  |  |  |  |  | | --- | --- | --- | --- | --- | | |  | | --- | | 13.2\*  If the subject is a child (under the age of 18 in Michigan), are any of the payments or incentives intended for the parent/guardian of the child? | |  | | N/A | |  | | |  |  | | --- | --- | | |  | | --- | |  | | |  | | | |  |  |  |  |  | | --- | --- | --- | --- | --- | | |  | | --- | | 13.3\*  Estimate the maximum total payment (including cash, checks, gift cards, and other cash-equivalent incentives) that an individual subject could receive for participating in this research in a single calendar year. | |  | | $26-$100 | |  | | |  |  | | --- | --- | | |  | | --- | |  | | |  | |      |  |  |  |  |  |  |  |  |  |  |  |  |  |  | | --- | --- | --- | --- | --- | --- | --- | --- | --- | --- | --- | --- | --- | --- | | |  |  |  |  |  |  |  |  |  | | --- | --- | --- | --- | --- | --- | --- | --- | --- | | |  | | --- | | 13.3.1\*  Please indicate what information you will be collecting from subjects that will be paid for their participation. | |  | | |  |  |  |  | | --- | --- | --- | --- | | | Select all that apply: | | --- | | Address | | Name | | | |  | | |  |  | | --- | --- | | |  | | --- | |  | | |  | |     |  |  |  |  |  |  |  |  |  |  | | --- | --- | --- | --- | --- | --- | --- | --- | --- | --- | | |  |  |  |  |  | | --- | --- | --- | --- | --- | | |  | | --- | | 13.4\*  Describe the frequency of the payments or incentives. If applicable, list any healthcare procedure(s) that will be provided to subjects at no charge. | |  | | The $25 cash payment will be presented to the participant on the day of their visit. No compensation will be paid at 6 month follow-up. One time payment of $20 gift card at end of supplement interview | |  | | |  |  | | --- | --- | | |  | | --- | |  | | |  | | | |  |  |  |  |  | | --- | --- | --- | --- | --- | | |  | | --- | | 13.5\*  What is the justification for offering these payments or incentives? | |  | | Compensation for time and effort on project | |  | | |  |  | | --- | --- | | |  | | --- | |  | | |  | | | |  |  |  |  |  | | --- | --- | --- | --- | --- | | |  | | --- | | 13.6\*  What is the plan to compensate subjects withdrawing from the research prior to completing the entire study. | |  | | Participants must complete all surveys and view website at their appointment (one day involvement) to receive compensation of $25 cash.  For supplement activity, if participants don't complete the interview, as indicated in the consent, they will not be paid. | |  | | |  |  | | --- | --- | | |  | | --- | |  | | |  | | |

|  |  |  |
| --- | --- | --- |
| |  |  | | --- | --- | |  |  | |
| 24. Secondary Data Analysis  **Completion of this section is required based on the response provided to either question 4-1.1 or 7.2.**   |  |  |  |  |  |  |  |  |  |  |  |  |  |  |  |  |  | | --- | --- | --- | --- | --- | --- | --- | --- | --- | --- | --- | --- | --- | --- | --- | --- | --- | | |  |  |  |  |  |  |  |  |  |  |  |  | | --- | --- | --- | --- | --- | --- | --- | --- | --- | --- | --- | --- | | |  | | --- | | 24.1\*  List each pre-existing data set that will be used in the study. | |  | | |  |  |  |  |  |  |  | | --- | --- | --- | --- | --- | --- | --- | | | Name | Identifying Info | Is Publicly Available | | --- | --- | --- | | medical record from their primary care physician | Colon cancer screening report for any screening done within the 6 months since the time of the study visit. The report will contain patient name, medical record number, contact information, age, date of birth-- anything that would be on a pathological report generated for the patient. This report will be scanned at the practice and uploaded to a secure server. The file name will contain only the research ID number. When the pathological results are entered into the data set all identifiers will be removed and will be linked by research ID number only. | no | | | |  | | |  |  | | --- | --- | | |  | | --- | |  | | | Additional Help | | |
| Secondary Data Set Detail     |  |  |  |  |  |  |  |  |  |  | | --- | --- | --- | --- | --- | --- | --- | --- | --- | --- | | |  |  |  |  |  | | --- | --- | --- | --- | --- | | |  | | --- | | 24.2\*  Name and source/location of data set: | |  | | medical record from their primary care physician | |  | | |  |  | | --- | --- | | |  | | --- | |  | | |  | | | |  |  |  |  |  | | --- | --- | --- | --- | --- | | |  | | --- | | 24.3\*  Describe the type of information contained in the data set, including any potential subject identifiers. | |  | | Colon cancer screening report for any screening done within the 6 months since the time of the study visit. The report will contain patient name, medical record number, contact information, age, date of birth-- anything that would be on a pathological report generated for the patient. This report will be scanned at the practice and uploaded to a secure server. The file name will contain only the research ID number. When the pathological results are entered into the data set all identifiers will be removed and will be linked by research ID number only. | |  | | |  |  | | --- | --- | | |  | | --- | |  | | |  | | | |  |  |  |  |  | | --- | --- | --- | --- | --- | | |  | | --- | | 24.4\*  Is the data set you are analyzing publicly available? | |  | | Yes **No** | |  | | |  |  | | --- | --- | | |  | | --- | |  | | |  | |      |  |  |  |  |  |  |  |  |  |  |  |  |  | | --- | --- | --- | --- | --- | --- | --- | --- | --- | --- | --- | --- | --- | | |  |  |  |  |  |  |  |  | | --- | --- | --- | --- | --- | --- | --- | --- | | |  | | --- | | 24.5\*  Does the data set contain: | |  | | |  |  |  | | --- | --- | --- | | | Identifier | | --- | | Coded Identifiers | | | |  | | |  |  | | --- | --- | | |  | | --- | |  | | |  | | | |  |  |  |  |  |  |  |  |  |  | | --- | --- | --- | --- | --- | --- | --- | --- | --- | --- | | |  | | --- | | 24.6  Upload any Data Use Agreement, letter of permission, or other access documentation for this data set | |  | | |  |  |  |  |  | | --- | --- | --- | --- | --- | | | Name | Version | | --- | --- | | SAWKADSA.pdf | History | 0.01 | | | |  | | |  |  | | --- | --- | | |  | | --- | |  | | |  | | | |  |  |  |  |  | | --- | --- | --- | --- | --- | | |  | | --- | | 24.7\*  Was prior consent from the subjects obtained to utilize the data set for this study (or for unspecified future research)? | |  | | Yes | |  | | |  |  | | --- | --- | | |  | | --- | |  | | |  | |      |  |  |  |  |  |  |  |  |  |  | | --- | --- | --- | --- | --- | --- | --- | --- | --- | --- | | |  |  |  |  |  | | --- | --- | --- | --- | --- | | |  | | --- | | 24.7.1\*  Indicate intention to obtain a waiver of informed consent (and, if applicable, HIPAA authorization) or describe the plan to re-establish the identifying links and re-consent individuals. | |  | |  | |  | | |  |  | | --- | --- | | |  | | --- | |  | | |  | |        |  |  |  |  |  |  |  |  |  |  | | --- | --- | --- | --- | --- | --- | --- | --- | --- | --- | | |  |  |  |  |  | | --- | --- | --- | --- | --- | | |  | | --- | | 24.8\*  Will you have access to a key that deciphers the code, thereby enabling linkage of identifying information to an individual subject’s private information or samples? | |  | | **Yes** No | |  | | |  |  | | --- | --- | | |  | | --- | |  | | | Additional Help | |      |  |  |  |  |  |  |  |  |  |  |  |  |  |  |  |  |  |  |  |  |  | | --- | --- | --- | --- | --- | --- | --- | --- | --- | --- | --- | --- | --- | --- | --- | --- | --- | --- | --- | --- | --- | | |  |  |  |  |  |  |  |  |  |  |  |  |  |  |  |  | | --- | --- | --- | --- | --- | --- | --- | --- | --- | --- | --- | --- | --- | --- | --- | --- | | |  | | --- | | 24.8.1\*  Identify the mechanism that precludes access to the codes, and upload (in Question 24.6) copies of any agreements or documents that explain these protections. | |  | | |  |  |  |  |  |  |  |  |  |  |  | | --- | --- | --- | --- | --- | --- | --- | --- | --- | --- | --- | | |  |  |  |  |  |  |  |  |  |  | | --- | --- | --- | --- | --- | --- | --- | --- | --- | --- | | There are no items to display | | | | | | | | | | |   If other, please specify: | |  | | |  |  | | --- | --- | | |  | | --- | |  | | |  | | |

|  |  |  |
| --- | --- | --- |
| |  |  | | --- | --- | |  |  | |
| 28. Internet/Email  **Completion of this section is required based on the response provided to question 7-1.4.**   |  |  |  |  |  |  |  |  |  |  | | --- | --- | --- | --- | --- | --- | --- | --- | --- | --- | | |  |  |  |  |  | | --- | --- | --- | --- | --- | | |  | | --- | | 28.1\*  Please explain the specific information technology resources that will be utilized. | |  | | Partcipants will be viewing either a non-interactive or interactive colorectal cancer website | |  | | |  |  | | --- | --- | | |  | | --- | |  | | |  | | | |  |  |  |  |  | | --- | --- | --- | --- | --- | | |  | | --- | | 28.2\*  Please explain the electronic security measures that will be employed to protect the privacy of the research subjects and the integrity of the information. | |  | | Website is housed on a secure server and data transfer is encrypted and participants will be given a login code. | |  | | |  |  | | --- | --- | | |  | | --- | |  | | |  | | | |  |  |  |  |  | | --- | --- | --- | --- | --- | | |  | | --- | | 28.3\*  Will representatives or advocates of the "community" under study be consulted in order to understand their expectations of privacy on the Internet or via email? | |  | | Yes **No** | |  | | |  |  | | --- | --- | | |  | | --- | |  | | |  | | | |  |  |  |  |  | | --- | --- | --- | --- | --- | | |  | | --- | | 28.3.1\*  Explain. | |  | | N/A | |  | | |  |  | | --- | --- | | |  | | --- | |  | | |  | | | |  |  |  |  |  | | --- | --- | --- | --- | --- | | |  | | --- | | 28.4\*  If the results will be published or presented, will the pseudonyms/screen names of individuals studied be disguised? | |  | | Yes | |  | | |  |  | | --- | --- | | |  | | --- | |  | | |  | | | |  |  |  |  |  | | --- | --- | --- | --- | --- | | |  | | --- | | 28.4.1\*  Explain. | |  | | Any identifiiers will be removed from the data before publication. | |  | | |  |  | | --- | --- | | |  | | --- | |  | | |  | | |

|  |  |  |
| --- | --- | --- |
| |  |  | | --- | --- | |  |  | |
| 29. Survey Research  **Completion of this section is required based on the response provided to question 7-1.5.**   |  |  |  |  |  |  |  |  |  |  |  |  |  |  |  |  |  |  |  |  |  |  |  |  |  |  |  |  |  |  |  |  |  |  |  |  |  |  |  |  |  |  |  |  |  |  |  |  |  |  |  |  |  |  |  |  |  |  |  |  |  |  |  |  |  |  | | --- | --- | --- | --- | --- | --- | --- | --- | --- | --- | --- | --- | --- | --- | --- | --- | --- | --- | --- | --- | --- | --- | --- | --- | --- | --- | --- | --- | --- | --- | --- | --- | --- | --- | --- | --- | --- | --- | --- | --- | --- | --- | --- | --- | --- | --- | --- | --- | --- | --- | --- | --- | --- | --- | --- | --- | --- | --- | --- | --- | --- | --- | --- | --- | --- | --- | | |  |  |  |  |  |  |  |  |  |  |  |  |  |  |  |  |  |  |  |  |  |  |  |  |  |  |  |  |  |  |  |  |  |  |  |  |  |  |  |  |  |  |  |  |  |  |  |  |  |  |  |  |  |  |  |  |  |  |  |  |  | | --- | --- | --- | --- | --- | --- | --- | --- | --- | --- | --- | --- | --- | --- | --- | --- | --- | --- | --- | --- | --- | --- | --- | --- | --- | --- | --- | --- | --- | --- | --- | --- | --- | --- | --- | --- | --- | --- | --- | --- | --- | --- | --- | --- | --- | --- | --- | --- | --- | --- | --- | --- | --- | --- | --- | --- | --- | --- | --- | --- | --- | | |  | | --- | | 29.1\*  Provide a list of all surveys and interviews used in the study: | |  | | |  |  |  |  |  |  |  |  |  |  |  |  |  |  |  |  |  |  |  |  |  |  |  |  |  |  |  |  |  |  |  |  |  |  |  |  |  |  |  |  |  |  |  |  |  |  |  |  |  |  |  |  |  |  |  |  | | --- | --- | --- | --- | --- | --- | --- | --- | --- | --- | --- | --- | --- | --- | --- | --- | --- | --- | --- | --- | --- | --- | --- | --- | --- | --- | --- | --- | --- | --- | --- | --- | --- | --- | --- | --- | --- | --- | --- | --- | --- | --- | --- | --- | --- | --- | --- | --- | --- | --- | --- | --- | --- | --- | --- | --- | | | Name | # of Questions | Duration | Sensitive? | Disturbing? | | --- | --- | --- | --- | --- | | 6-Month Chart Audit | 17 | 0 | no | no | | 6-month follow up | 8 | 5 minutes | no | no | | 6-month follow-up script | 0 | 5 minutes | no | no | | Baseline | 36 | 10 minutes | no | no | | Documenting Time | 0 | 0 | no | no | | Physician Survey | 19 | 5 minutes | no | no | | Post | 38 | 10 minutes | no | no | | post physician interaction | 30 | 5 minutes | no | no | | Risk Assessment Control Survey | 15 | 5 minutes | no | no | | Semi-structured phone survey | 4 | 30-60 minutes | no | no | | | |  | | |  |  | | --- | --- | | |  | | --- | |  | | | Additional Help | | | |  |  |  |  |  | | --- | --- | --- | --- | --- | | |  | | --- | | 29.13\*  Will the research involve the use of focus groups? | |  | | Yes **No** | |  | | |  |  | | --- | --- | | |  | | --- | |  | | |  | |      |  |  |  |  |  |  |  |  |  |  | | --- | --- | --- | --- | --- | --- | --- | --- | --- | --- | | |  |  |  |  |  | | --- | --- | --- | --- | --- | | |  | | --- | | 29.13.1\*  How will the identity of individuals participating in the groups be protected? | |  | |  | |  | | |  |  | | --- | --- | | |  | | --- | |  | | |  | |     |  |  |  |  |  |  |  |  |  |  | | --- | --- | --- | --- | --- | --- | --- | --- | --- | --- | | |  |  |  |  |  | | --- | --- | --- | --- | --- | | |  | | --- | | 29.14\*  Is any of the material disturbing? | |  | | Yes **No** | |  | | |  |  | | --- | --- | | |  | | --- | |  | | |  | |      |  |  |  |  |  |  |  |  |  |  | | --- | --- | --- | --- | --- | --- | --- | --- | --- | --- | | |  |  |  |  |  | | --- | --- | --- | --- | --- | | |  | | --- | | 29.15\*  Describe the arrangements made to provide professional counseling or support resources to any subjects desiring such assistance as a result of their participation in the study. | |  | |  | |  | | |  |  | | --- | --- | | |  | | --- | |  | | |  | | | |  |  |  |  |  | | --- | --- | --- | --- | --- | | |  | | --- | | 29.16\*  Indicate how the list of counseling or support resources will be provided: | |  | | If other, please specify: | |  | | |  |  | | --- | --- | | |  | | --- | |  | | |  | | |
| Survey Detail     |  |  |  |  |  |  |  |  |  |  | | --- | --- | --- | --- | --- | --- | --- | --- | --- | --- | | |  |  |  |  |  | | --- | --- | --- | --- | --- | | |  | | --- | | 29.2\*  Survey or interview name: | |  | | 6-Month Chart Audit | |  | | |  |  | | --- | --- | | |  | | --- | |  | | |  | | | |  |  |  |  |  | | --- | --- | --- | --- | --- | | |  | | --- | | 29.3\*  Is the design or development of this survey instrument dependent on receipt of funding or hiring of personnel? | |  | | Yes **No** | |  | | |  |  | | --- | --- | | |  | | --- | |  | | |  | |      |  |  |  |  |  |  |  |  |  |  | | --- | --- | --- | --- | --- | --- | --- | --- | --- | --- | | |  |  |  |  |  | | --- | --- | --- | --- | --- | | |  | | --- | | 29.4\*  In what manner will the survey or interview be conducted (e.g., in-person, Internet, mail, telephone, etc.)? *Special Note: For electronic surveys, the eResearch ID number must be included in the informed consent document (uploaded in section 10-1) or other material that serves as the informed consent.* | |  | | chart audit | |  | | |  |  | | --- | --- | | |  | | --- | |  | | |  | | | |  |  |  |  |  | | --- | --- | --- | --- | --- | | |  | | --- | | 29.5\*  What is the predicted response rate? | |  | | 100 % | |  | | |  |  | | --- | --- | | |  | | --- | |  | | |  | | | |  |  |  |  |  | | --- | --- | --- | --- | --- | | |  | | --- | | 29.6\*  What is the total number of questions? | |  | | 17 | |  | | |  |  | | --- | --- | | |  | | --- | |  | | |  | | | |  |  |  |  |  | | --- | --- | --- | --- | --- | | |  | | --- | | 29.7\*  What is the anticipated cumulative amount of time required for each subject? | |  | | 0 | |  | | |  |  | | --- | --- | | |  | | --- | |  | | |  | | | |  |  |  |  |  | | --- | --- | --- | --- | --- | | |  | | --- | | 29.8\*  What is the total number of interviews/data collection interactions with an individual subject? | |  | | 2 | |  | | |  |  | | --- | --- | | |  | | --- | |  | | |  | | | |  |  |  |  |  | | --- | --- | --- | --- | --- | | |  | | --- | | 29.9\*  Does the survey or interview contain questions of a sensitive nature (e.g., mental illness, sexual abuse, illicit drug use, etc.)? | |  | | Yes **No** | |  | | |  |  | | --- | --- | | |  | | --- | |  | | |  | | | |  |  |  |  |  | | --- | --- | --- | --- | --- | | |  | | --- | | 29.10\*  Is the survey or interview likely to produce psychological discomfort or negative feelings in the subjects? | |  | | Yes **No** | |  | | |  |  | | --- | --- | | |  | | --- | |  | | |  | | | |  |  |  |  |  | | --- | --- | --- | --- | --- | | |  | | --- | | 29.11\*  Has the survey instrument been validated or used in standard practice? | |  | | Yes **No** | |  | | |  |  | | --- | --- | | |  | | --- | |  | | |  | |      |  |  |  |  |  |  |  |  |  |  | | --- | --- | --- | --- | --- | --- | --- | --- | --- | --- | | |  |  |  |  |  | | --- | --- | --- | --- | --- | | |  | | --- | | 29.11.1\*  If yes, describe the origin of the instrument. | |  | |  | |  | | |  |  | | --- | --- | | |  | | --- | |  | | |  | |     |  |  |  |  |  |  |  |  |  |  |  |  |  |  |  |  |  | | --- | --- | --- | --- | --- | --- | --- | --- | --- | --- | --- | --- | --- | --- | --- | --- | --- | | |  |  |  |  |  |  |  |  |  |  |  |  | | --- | --- | --- | --- | --- | --- | --- | --- | --- | --- | --- | --- | | |  | | --- | | 29.12\*  Upload the survey instrument here. | |  | | |  |  |  |  |  |  |  | | --- | --- | --- | --- | --- | --- | --- | | | Name | Version | | --- | --- | | CLEAN\_DATESChartAudit\_6.4.13\_V.2.5.docx | History | 0.06 | | TRACK\_DATESChartAudit\_6.4.13\_V.2.5.docx | History | 0.05 | | | |  | | |  |  | | --- | --- | | |  | | --- | |  | | | Additional Help | | |
| Survey Detail     |  |  |  |  |  |  |  |  |  |  | | --- | --- | --- | --- | --- | --- | --- | --- | --- | --- | | |  |  |  |  |  | | --- | --- | --- | --- | --- | | |  | | --- | | 29.2\*  Survey or interview name: | |  | | 6-month follow up | |  | | |  |  | | --- | --- | | |  | | --- | |  | | |  | | | |  |  |  |  |  | | --- | --- | --- | --- | --- | | |  | | --- | | 29.3\*  Is the design or development of this survey instrument dependent on receipt of funding or hiring of personnel? | |  | | Yes **No** | |  | | |  |  | | --- | --- | | |  | | --- | |  | | |  | |      |  |  |  |  |  |  |  |  |  |  | | --- | --- | --- | --- | --- | --- | --- | --- | --- | --- | | |  |  |  |  |  | | --- | --- | --- | --- | --- | | |  | | --- | | 29.4\*  In what manner will the survey or interview be conducted (e.g., in-person, Internet, mail, telephone, etc.)? *Special Note: For electronic surveys, the eResearch ID number must be included in the informed consent document (uploaded in section 10-1) or other material that serves as the informed consent.* | |  | | telephone | |  | | |  |  | | --- | --- | | |  | | --- | |  | | |  | | | |  |  |  |  |  | | --- | --- | --- | --- | --- | | |  | | --- | | 29.5\*  What is the predicted response rate? | |  | | 100 % | |  | | |  |  | | --- | --- | | |  | | --- | |  | | |  | | | |  |  |  |  |  | | --- | --- | --- | --- | --- | | |  | | --- | | 29.6\*  What is the total number of questions? | |  | | 8 | |  | | |  |  | | --- | --- | | |  | | --- | |  | | |  | | | |  |  |  |  |  | | --- | --- | --- | --- | --- | | |  | | --- | | 29.7\*  What is the anticipated cumulative amount of time required for each subject? | |  | | 5 minutes | |  | | |  |  | | --- | --- | | |  | | --- | |  | | |  | | | |  |  |  |  |  | | --- | --- | --- | --- | --- | | |  | | --- | | 29.8\*  What is the total number of interviews/data collection interactions with an individual subject? | |  | | 3 | |  | | |  |  | | --- | --- | | |  | | --- | |  | | |  | | | |  |  |  |  |  | | --- | --- | --- | --- | --- | | |  | | --- | | 29.9\*  Does the survey or interview contain questions of a sensitive nature (e.g., mental illness, sexual abuse, illicit drug use, etc.)? | |  | | Yes **No** | |  | | |  |  | | --- | --- | | |  | | --- | |  | | |  | | | |  |  |  |  |  | | --- | --- | --- | --- | --- | | |  | | --- | | 29.10\*  Is the survey or interview likely to produce psychological discomfort or negative feelings in the subjects? | |  | | Yes **No** | |  | | |  |  | | --- | --- | | |  | | --- | |  | | |  | | | |  |  |  |  |  | | --- | --- | --- | --- | --- | | |  | | --- | | 29.11\*  Has the survey instrument been validated or used in standard practice? | |  | | Yes **No** | |  | | |  |  | | --- | --- | | |  | | --- | |  | | |  | |      |  |  |  |  |  |  |  |  |  |  | | --- | --- | --- | --- | --- | --- | --- | --- | --- | --- | | |  |  |  |  |  | | --- | --- | --- | --- | --- | | |  | | --- | | 29.11.1\*  If yes, describe the origin of the instrument. | |  | |  | |  | | |  |  | | --- | --- | | |  | | --- | |  | | |  | |     |  |  |  |  |  |  |  |  |  |  |  |  |  |  |  |  |  | | --- | --- | --- | --- | --- | --- | --- | --- | --- | --- | --- | --- | --- | --- | --- | --- | --- | | |  |  |  |  |  |  |  |  |  |  |  |  | | --- | --- | --- | --- | --- | --- | --- | --- | --- | --- | --- | --- | | |  | | --- | | 29.12\*  Upload the survey instrument here. | |  | | |  |  |  |  |  |  |  | | --- | --- | --- | --- | --- | --- | --- | | | Name | Version | | --- | --- | | DATES6 Month Follow Up Survey\_11.8.12.2.docx | History | 0.04 | | TRACK 6-month Follow-up Survey\_11.8.12.2 | History | 0.02 | | | |  | | |  |  | | --- | --- | | |  | | --- | |  | | | Additional Help | | |
| Survey Detail     |  |  |  |  |  |  |  |  |  |  | | --- | --- | --- | --- | --- | --- | --- | --- | --- | --- | | |  |  |  |  |  | | --- | --- | --- | --- | --- | | |  | | --- | | 29.2\*  Survey or interview name: | |  | | 6-month follow-up script | |  | | |  |  | | --- | --- | | |  | | --- | |  | | |  | | | |  |  |  |  |  | | --- | --- | --- | --- | --- | | |  | | --- | | 29.3\*  Is the design or development of this survey instrument dependent on receipt of funding or hiring of personnel? | |  | | Yes **No** | |  | | |  |  | | --- | --- | | |  | | --- | |  | | |  | |      |  |  |  |  |  |  |  |  |  |  | | --- | --- | --- | --- | --- | --- | --- | --- | --- | --- | | |  |  |  |  |  | | --- | --- | --- | --- | --- | | |  | | --- | | 29.4\*  In what manner will the survey or interview be conducted (e.g., in-person, Internet, mail, telephone, etc.)? *Special Note: For electronic surveys, the eResearch ID number must be included in the informed consent document (uploaded in section 10-1) or other material that serves as the informed consent.* | |  | | telephone | |  | | |  |  | | --- | --- | | |  | | --- | |  | | |  | | | |  |  |  |  |  | | --- | --- | --- | --- | --- | | |  | | --- | | 29.5\*  What is the predicted response rate? | |  | | 100 % | |  | | |  |  | | --- | --- | | |  | | --- | |  | | |  | | | |  |  |  |  |  | | --- | --- | --- | --- | --- | | |  | | --- | | 29.6\*  What is the total number of questions? | |  | | 0 | |  | | |  |  | | --- | --- | | |  | | --- | |  | | |  | | | |  |  |  |  |  | | --- | --- | --- | --- | --- | | |  | | --- | | 29.7\*  What is the anticipated cumulative amount of time required for each subject? | |  | | 5 minutes | |  | | |  |  | | --- | --- | | |  | | --- | |  | | |  | | | |  |  |  |  |  | | --- | --- | --- | --- | --- | | |  | | --- | | 29.8\*  What is the total number of interviews/data collection interactions with an individual subject? | |  | | 2 | |  | | |  |  | | --- | --- | | |  | | --- | |  | | |  | | | |  |  |  |  |  | | --- | --- | --- | --- | --- | | |  | | --- | | 29.9\*  Does the survey or interview contain questions of a sensitive nature (e.g., mental illness, sexual abuse, illicit drug use, etc.)? | |  | | Yes **No** | |  | | |  |  | | --- | --- | | |  | | --- | |  | | |  | | | |  |  |  |  |  | | --- | --- | --- | --- | --- | | |  | | --- | | 29.10\*  Is the survey or interview likely to produce psychological discomfort or negative feelings in the subjects? | |  | | Yes **No** | |  | | |  |  | | --- | --- | | |  | | --- | |  | | |  | | | |  |  |  |  |  | | --- | --- | --- | --- | --- | | |  | | --- | | 29.11\*  Has the survey instrument been validated or used in standard practice? | |  | | Yes **No** | |  | | |  |  | | --- | --- | | |  | | --- | |  | | |  | |      |  |  |  |  |  |  |  |  |  |  | | --- | --- | --- | --- | --- | --- | --- | --- | --- | --- | | |  |  |  |  |  | | --- | --- | --- | --- | --- | | |  | | --- | | 29.11.1\*  If yes, describe the origin of the instrument. | |  | |  | |  | | |  |  | | --- | --- | | |  | | --- | |  | | |  | |     |  |  |  |  |  |  |  |  |  |  |  |  |  |  |  | | --- | --- | --- | --- | --- | --- | --- | --- | --- | --- | --- | --- | --- | --- | --- | | |  |  |  |  |  |  |  |  |  |  | | --- | --- | --- | --- | --- | --- | --- | --- | --- | --- | | |  | | --- | | 29.12\*  Upload the survey instrument here. | |  | | |  |  |  |  |  | | --- | --- | --- | --- | --- | | | Name | Version | | --- | --- | | 6-month follow-up script | History | 0.02 | | | |  | | |  |  | | --- | --- | | |  | | --- | |  | | | Additional Help | | |
| Survey Detail     |  |  |  |  |  |  |  |  |  |  | | --- | --- | --- | --- | --- | --- | --- | --- | --- | --- | | |  |  |  |  |  | | --- | --- | --- | --- | --- | | |  | | --- | | 29.2\*  Survey or interview name: | |  | | Baseline | |  | | |  |  | | --- | --- | | |  | | --- | |  | | |  | | | |  |  |  |  |  | | --- | --- | --- | --- | --- | | |  | | --- | | 29.3\*  Is the design or development of this survey instrument dependent on receipt of funding or hiring of personnel? | |  | | Yes **No** | |  | | |  |  | | --- | --- | | |  | | --- | |  | | |  | |      |  |  |  |  |  |  |  |  |  |  | | --- | --- | --- | --- | --- | --- | --- | --- | --- | --- | | |  |  |  |  |  | | --- | --- | --- | --- | --- | | |  | | --- | | 29.4\*  In what manner will the survey or interview be conducted (e.g., in-person, Internet, mail, telephone, etc.)? *Special Note: For electronic surveys, the eResearch ID number must be included in the informed consent document (uploaded in section 10-1) or other material that serves as the informed consent.* | |  | | electronic | |  | | |  |  | | --- | --- | | |  | | --- | |  | | |  | | | |  |  |  |  |  | | --- | --- | --- | --- | --- | | |  | | --- | | 29.5\*  What is the predicted response rate? | |  | | 100 % | |  | | |  |  | | --- | --- | | |  | | --- | |  | | |  | | | |  |  |  |  |  | | --- | --- | --- | --- | --- | | |  | | --- | | 29.6\*  What is the total number of questions? | |  | | 36 | |  | | |  |  | | --- | --- | | |  | | --- | |  | | |  | | | |  |  |  |  |  | | --- | --- | --- | --- | --- | | |  | | --- | | 29.7\*  What is the anticipated cumulative amount of time required for each subject? | |  | | 10 minutes | |  | | |  |  | | --- | --- | | |  | | --- | |  | | |  | | | |  |  |  |  |  | | --- | --- | --- | --- | --- | | |  | | --- | | 29.8\*  What is the total number of interviews/data collection interactions with an individual subject? | |  | | 2 | |  | | |  |  | | --- | --- | | |  | | --- | |  | | |  | | | |  |  |  |  |  | | --- | --- | --- | --- | --- | | |  | | --- | | 29.9\*  Does the survey or interview contain questions of a sensitive nature (e.g., mental illness, sexual abuse, illicit drug use, etc.)? | |  | | Yes **No** | |  | | |  |  | | --- | --- | | |  | | --- | |  | | |  | | | |  |  |  |  |  | | --- | --- | --- | --- | --- | | |  | | --- | | 29.10\*  Is the survey or interview likely to produce psychological discomfort or negative feelings in the subjects? | |  | | Yes **No** | |  | | |  |  | | --- | --- | | |  | | --- | |  | | |  | | | |  |  |  |  |  | | --- | --- | --- | --- | --- | | |  | | --- | | 29.11\*  Has the survey instrument been validated or used in standard practice? | |  | | Yes **No** | |  | | |  |  | | --- | --- | | |  | | --- | |  | | |  | |      |  |  |  |  |  |  |  |  |  |  | | --- | --- | --- | --- | --- | --- | --- | --- | --- | --- | | |  |  |  |  |  | | --- | --- | --- | --- | --- | | |  | | --- | | 29.11.1\*  If yes, describe the origin of the instrument. | |  | |  | |  | | |  |  | | --- | --- | | |  | | --- | |  | | |  | |     |  |  |  |  |  |  |  |  |  |  |  |  |  |  |  | | --- | --- | --- | --- | --- | --- | --- | --- | --- | --- | --- | --- | --- | --- | --- | | |  |  |  |  |  |  |  |  |  |  | | --- | --- | --- | --- | --- | --- | --- | --- | --- | --- | | |  | | --- | | 29.12\*  Upload the survey instrument here. | |  | | |  |  |  |  |  | | --- | --- | --- | --- | --- | | | Name | Version | | --- | --- | | Baseline | History | 0.01 | | | |  | | |  |  | | --- | --- | | |  | | --- | |  | | | Additional Help | | |
| Survey Detail     |  |  |  |  |  |  |  |  |  |  | | --- | --- | --- | --- | --- | --- | --- | --- | --- | --- | | |  |  |  |  |  | | --- | --- | --- | --- | --- | | |  | | --- | | 29.2\*  Survey or interview name: | |  | | Documenting Time | |  | | |  |  | | --- | --- | | |  | | --- | |  | | |  | | | |  |  |  |  |  | | --- | --- | --- | --- | --- | | |  | | --- | | 29.3\*  Is the design or development of this survey instrument dependent on receipt of funding or hiring of personnel? | |  | | Yes **No** | |  | | |  |  | | --- | --- | | |  | | --- | |  | | |  | |      |  |  |  |  |  |  |  |  |  |  | | --- | --- | --- | --- | --- | --- | --- | --- | --- | --- | | |  |  |  |  |  | | --- | --- | --- | --- | --- | | |  | | --- | | 29.4\*  In what manner will the survey or interview be conducted (e.g., in-person, Internet, mail, telephone, etc.)? *Special Note: For electronic surveys, the eResearch ID number must be included in the informed consent document (uploaded in section 10-1) or other material that serves as the informed consent.* | |  | | RAs will document the amount of time involved during study visit | |  | | |  |  | | --- | --- | | |  | | --- | |  | | |  | | | |  |  |  |  |  | | --- | --- | --- | --- | --- | | |  | | --- | | 29.5\*  What is the predicted response rate? | |  | | 100 % | |  | | |  |  | | --- | --- | | |  | | --- | |  | | |  | | | |  |  |  |  |  | | --- | --- | --- | --- | --- | | |  | | --- | | 29.6\*  What is the total number of questions? | |  | | 0 | |  | | |  |  | | --- | --- | | |  | | --- | |  | | |  | | | |  |  |  |  |  | | --- | --- | --- | --- | --- | | |  | | --- | | 29.7\*  What is the anticipated cumulative amount of time required for each subject? | |  | | 0 | |  | | |  |  | | --- | --- | | |  | | --- | |  | | |  | | | |  |  |  |  |  | | --- | --- | --- | --- | --- | | |  | | --- | | 29.8\*  What is the total number of interviews/data collection interactions with an individual subject? | |  | | 2 | |  | | |  |  | | --- | --- | | |  | | --- | |  | | |  | | | |  |  |  |  |  | | --- | --- | --- | --- | --- | | |  | | --- | | 29.9\*  Does the survey or interview contain questions of a sensitive nature (e.g., mental illness, sexual abuse, illicit drug use, etc.)? | |  | | Yes **No** | |  | | |  |  | | --- | --- | | |  | | --- | |  | | |  | | | |  |  |  |  |  | | --- | --- | --- | --- | --- | | |  | | --- | | 29.10\*  Is the survey or interview likely to produce psychological discomfort or negative feelings in the subjects? | |  | | Yes **No** | |  | | |  |  | | --- | --- | | |  | | --- | |  | | |  | | | |  |  |  |  |  | | --- | --- | --- | --- | --- | | |  | | --- | | 29.11\*  Has the survey instrument been validated or used in standard practice? | |  | | Yes **No** | |  | | |  |  | | --- | --- | | |  | | --- | |  | | |  | |      |  |  |  |  |  |  |  |  |  |  | | --- | --- | --- | --- | --- | --- | --- | --- | --- | --- | | |  |  |  |  |  | | --- | --- | --- | --- | --- | | |  | | --- | | 29.11.1\*  If yes, describe the origin of the instrument. | |  | |  | |  | | |  |  | | --- | --- | | |  | | --- | |  | | |  | |     |  |  |  |  |  |  |  |  |  |  |  |  |  |  |  | | --- | --- | --- | --- | --- | --- | --- | --- | --- | --- | --- | --- | --- | --- | --- | | |  |  |  |  |  |  |  |  |  |  | | --- | --- | --- | --- | --- | --- | --- | --- | --- | --- | | |  | | --- | | 29.12\*  Upload the survey instrument here. | |  | | |  |  |  |  |  | | --- | --- | --- | --- | --- | | | Name | Version | | --- | --- | | Documenting Time | History | 0.01 | | | |  | | |  |  | | --- | --- | | |  | | --- | |  | | | Additional Help | | |
| Survey Detail     |  |  |  |  |  |  |  |  |  |  | | --- | --- | --- | --- | --- | --- | --- | --- | --- | --- | | |  |  |  |  |  | | --- | --- | --- | --- | --- | | |  | | --- | | 29.2\*  Survey or interview name: | |  | | Physician Survey | |  | | |  |  | | --- | --- | | |  | | --- | |  | | |  | | | |  |  |  |  |  | | --- | --- | --- | --- | --- | | |  | | --- | | 29.3\*  Is the design or development of this survey instrument dependent on receipt of funding or hiring of personnel? | |  | | Yes **No** | |  | | |  |  | | --- | --- | | |  | | --- | |  | | |  | |      |  |  |  |  |  |  |  |  |  |  | | --- | --- | --- | --- | --- | --- | --- | --- | --- | --- | | |  |  |  |  |  | | --- | --- | --- | --- | --- | | |  | | --- | | 29.4\*  In what manner will the survey or interview be conducted (e.g., in-person, Internet, mail, telephone, etc.)? *Special Note: For electronic surveys, the eResearch ID number must be included in the informed consent document (uploaded in section 10-1) or other material that serves as the informed consent.* | |  | | electronic | |  | | |  |  | | --- | --- | | |  | | --- | |  | | |  | | | |  |  |  |  |  | | --- | --- | --- | --- | --- | | |  | | --- | | 29.5\*  What is the predicted response rate? | |  | | 100 % | |  | | |  |  | | --- | --- | | |  | | --- | |  | | |  | | | |  |  |  |  |  | | --- | --- | --- | --- | --- | | |  | | --- | | 29.6\*  What is the total number of questions? | |  | | 19 | |  | | |  |  | | --- | --- | | |  | | --- | |  | | |  | | | |  |  |  |  |  | | --- | --- | --- | --- | --- | | |  | | --- | | 29.7\*  What is the anticipated cumulative amount of time required for each subject? | |  | | 5 minutes | |  | | |  |  | | --- | --- | | |  | | --- | |  | | |  | | | |  |  |  |  |  | | --- | --- | --- | --- | --- | | |  | | --- | | 29.8\*  What is the total number of interviews/data collection interactions with an individual subject? | |  | | 1 | |  | | |  |  | | --- | --- | | |  | | --- | |  | | |  | | | |  |  |  |  |  | | --- | --- | --- | --- | --- | | |  | | --- | | 29.9\*  Does the survey or interview contain questions of a sensitive nature (e.g., mental illness, sexual abuse, illicit drug use, etc.)? | |  | | Yes **No** | |  | | |  |  | | --- | --- | | |  | | --- | |  | | |  | | | |  |  |  |  |  | | --- | --- | --- | --- | --- | | |  | | --- | | 29.10\*  Is the survey or interview likely to produce psychological discomfort or negative feelings in the subjects? | |  | | Yes **No** | |  | | |  |  | | --- | --- | | |  | | --- | |  | | |  | | | |  |  |  |  |  | | --- | --- | --- | --- | --- | | |  | | --- | | 29.11\*  Has the survey instrument been validated or used in standard practice? | |  | | Yes **No** | |  | | |  |  | | --- | --- | | |  | | --- | |  | | |  | |      |  |  |  |  |  |  |  |  |  |  | | --- | --- | --- | --- | --- | --- | --- | --- | --- | --- | | |  |  |  |  |  | | --- | --- | --- | --- | --- | | |  | | --- | | 29.11.1\*  If yes, describe the origin of the instrument. | |  | |  | |  | | |  |  | | --- | --- | | |  | | --- | |  | | |  | |     |  |  |  |  |  |  |  |  |  |  |  |  |  |  |  | | --- | --- | --- | --- | --- | --- | --- | --- | --- | --- | --- | --- | --- | --- | --- | | |  |  |  |  |  |  |  |  |  |  | | --- | --- | --- | --- | --- | --- | --- | --- | --- | --- | | |  | | --- | | 29.12\*  Upload the survey instrument here. | |  | | |  |  |  |  |  | | --- | --- | --- | --- | --- | | | Name | Version | | --- | --- | | Physician Survey REV 2.21.12 | History | 0.02 | | | |  | | |  |  | | --- | --- | | |  | | --- | |  | | | Additional Help | | |
| Survey Detail     |  |  |  |  |  |  |  |  |  |  | | --- | --- | --- | --- | --- | --- | --- | --- | --- | --- | | |  |  |  |  |  | | --- | --- | --- | --- | --- | | |  | | --- | | 29.2\*  Survey or interview name: | |  | | Post | |  | | |  |  | | --- | --- | | |  | | --- | |  | | |  | | | |  |  |  |  |  | | --- | --- | --- | --- | --- | | |  | | --- | | 29.3\*  Is the design or development of this survey instrument dependent on receipt of funding or hiring of personnel? | |  | | Yes **No** | |  | | |  |  | | --- | --- | | |  | | --- | |  | | |  | |      |  |  |  |  |  |  |  |  |  |  | | --- | --- | --- | --- | --- | --- | --- | --- | --- | --- | | |  |  |  |  |  | | --- | --- | --- | --- | --- | | |  | | --- | | 29.4\*  In what manner will the survey or interview be conducted (e.g., in-person, Internet, mail, telephone, etc.)? *Special Note: For electronic surveys, the eResearch ID number must be included in the informed consent document (uploaded in section 10-1) or other material that serves as the informed consent.* | |  | | electronic | |  | | |  |  | | --- | --- | | |  | | --- | |  | | |  | | | |  |  |  |  |  | | --- | --- | --- | --- | --- | | |  | | --- | | 29.5\*  What is the predicted response rate? | |  | | 100 % | |  | | |  |  | | --- | --- | | |  | | --- | |  | | |  | | | |  |  |  |  |  | | --- | --- | --- | --- | --- | | |  | | --- | | 29.6\*  What is the total number of questions? | |  | | 38 | |  | | |  |  | | --- | --- | | |  | | --- | |  | | |  | | | |  |  |  |  |  | | --- | --- | --- | --- | --- | | |  | | --- | | 29.7\*  What is the anticipated cumulative amount of time required for each subject? | |  | | 10 minutes | |  | | |  |  | | --- | --- | | |  | | --- | |  | | |  | | | |  |  |  |  |  | | --- | --- | --- | --- | --- | | |  | | --- | | 29.8\*  What is the total number of interviews/data collection interactions with an individual subject? | |  | | 2 | |  | | |  |  | | --- | --- | | |  | | --- | |  | | |  | | | |  |  |  |  |  | | --- | --- | --- | --- | --- | | |  | | --- | | 29.9\*  Does the survey or interview contain questions of a sensitive nature (e.g., mental illness, sexual abuse, illicit drug use, etc.)? | |  | | Yes **No** | |  | | |  |  | | --- | --- | | |  | | --- | |  | | |  | | | |  |  |  |  |  | | --- | --- | --- | --- | --- | | |  | | --- | | 29.10\*  Is the survey or interview likely to produce psychological discomfort or negative feelings in the subjects? | |  | | Yes **No** | |  | | |  |  | | --- | --- | | |  | | --- | |  | | |  | | | |  |  |  |  |  | | --- | --- | --- | --- | --- | | |  | | --- | | 29.11\*  Has the survey instrument been validated or used in standard practice? | |  | | Yes **No** | |  | | |  |  | | --- | --- | | |  | | --- | |  | | |  | |      |  |  |  |  |  |  |  |  |  |  | | --- | --- | --- | --- | --- | --- | --- | --- | --- | --- | | |  |  |  |  |  | | --- | --- | --- | --- | --- | | |  | | --- | | 29.11.1\*  If yes, describe the origin of the instrument. | |  | |  | |  | | |  |  | | --- | --- | | |  | | --- | |  | | |  | |     |  |  |  |  |  |  |  |  |  |  |  |  |  |  |  | | --- | --- | --- | --- | --- | --- | --- | --- | --- | --- | --- | --- | --- | --- | --- | | |  |  |  |  |  |  |  |  |  |  | | --- | --- | --- | --- | --- | --- | --- | --- | --- | --- | | |  | | --- | | 29.12\*  Upload the survey instrument here. | |  | | |  |  |  |  |  | | --- | --- | --- | --- | --- | | | Name | Version | | --- | --- | | DATES Patient Post-test MarkUp 10-11-11.doc | History | 0.03 | | | |  | | |  |  | | --- | --- | | |  | | --- | |  | | | Additional Help | | |
| Survey Detail     |  |  |  |  |  |  |  |  |  |  | | --- | --- | --- | --- | --- | --- | --- | --- | --- | --- | | |  |  |  |  |  | | --- | --- | --- | --- | --- | | |  | | --- | | 29.2\*  Survey or interview name: | |  | | post physician interaction | |  | | |  |  | | --- | --- | | |  | | --- | |  | | |  | | | |  |  |  |  |  | | --- | --- | --- | --- | --- | | |  | | --- | | 29.3\*  Is the design or development of this survey instrument dependent on receipt of funding or hiring of personnel? | |  | | Yes **No** | |  | | |  |  | | --- | --- | | |  | | --- | |  | | |  | |      |  |  |  |  |  |  |  |  |  |  | | --- | --- | --- | --- | --- | --- | --- | --- | --- | --- | | |  |  |  |  |  | | --- | --- | --- | --- | --- | | |  | | --- | | 29.4\*  In what manner will the survey or interview be conducted (e.g., in-person, Internet, mail, telephone, etc.)? *Special Note: For electronic surveys, the eResearch ID number must be included in the informed consent document (uploaded in section 10-1) or other material that serves as the informed consent.* | |  | | in person | |  | | |  |  | | --- | --- | | |  | | --- | |  | | |  | | | |  |  |  |  |  | | --- | --- | --- | --- | --- | | |  | | --- | | 29.5\*  What is the predicted response rate? | |  | | 100 % | |  | | |  |  | | --- | --- | | |  | | --- | |  | | |  | | | |  |  |  |  |  | | --- | --- | --- | --- | --- | | |  | | --- | | 29.6\*  What is the total number of questions? | |  | | 30 | |  | | |  |  | | --- | --- | | |  | | --- | |  | | |  | | | |  |  |  |  |  | | --- | --- | --- | --- | --- | | |  | | --- | | 29.7\*  What is the anticipated cumulative amount of time required for each subject? | |  | | 5 minutes | |  | | |  |  | | --- | --- | | |  | | --- | |  | | |  | | | |  |  |  |  |  | | --- | --- | --- | --- | --- | | |  | | --- | | 29.8\*  What is the total number of interviews/data collection interactions with an individual subject? | |  | | 2 | |  | | |  |  | | --- | --- | | |  | | --- | |  | | |  | | | |  |  |  |  |  | | --- | --- | --- | --- | --- | | |  | | --- | | 29.9\*  Does the survey or interview contain questions of a sensitive nature (e.g., mental illness, sexual abuse, illicit drug use, etc.)? | |  | | Yes **No** | |  | | |  |  | | --- | --- | | |  | | --- | |  | | |  | | | |  |  |  |  |  | | --- | --- | --- | --- | --- | | |  | | --- | | 29.10\*  Is the survey or interview likely to produce psychological discomfort or negative feelings in the subjects? | |  | | Yes **No** | |  | | |  |  | | --- | --- | | |  | | --- | |  | | |  | | | |  |  |  |  |  | | --- | --- | --- | --- | --- | | |  | | --- | | 29.11\*  Has the survey instrument been validated or used in standard practice? | |  | | Yes **No** | |  | | |  |  | | --- | --- | | |  | | --- | |  | | |  | |      |  |  |  |  |  |  |  |  |  |  | | --- | --- | --- | --- | --- | --- | --- | --- | --- | --- | | |  |  |  |  |  | | --- | --- | --- | --- | --- | | |  | | --- | | 29.11.1\*  If yes, describe the origin of the instrument. | |  | |  | |  | | |  |  | | --- | --- | | |  | | --- | |  | | |  | |     |  |  |  |  |  |  |  |  |  |  |  |  |  |  |  |  |  | | --- | --- | --- | --- | --- | --- | --- | --- | --- | --- | --- | --- | --- | --- | --- | --- | --- | | |  |  |  |  |  |  |  |  |  |  |  |  | | --- | --- | --- | --- | --- | --- | --- | --- | --- | --- | --- | --- | | |  | | --- | | 29.12\*  Upload the survey instrument here. | |  | | |  |  |  |  |  |  |  | | --- | --- | --- | --- | --- | --- | --- | | | Name | Version | | --- | --- | | CLEAN\_Post Encounter Survey\_5.22.13\_v.1.8 | History | 0.08 | | TRACK\_Post Encounter Survey\_5.22.13\_v.1.8 | History | 0.03 | | | |  | | |  |  | | --- | --- | | |  | | --- | |  | | | Additional Help | | |
| Survey Detail     |  |  |  |  |  |  |  |  |  |  | | --- | --- | --- | --- | --- | --- | --- | --- | --- | --- | | |  |  |  |  |  | | --- | --- | --- | --- | --- | | |  | | --- | | 29.2\*  Survey or interview name: | |  | | Risk Assessment Control Survey | |  | | |  |  | | --- | --- | | |  | | --- | |  | | |  | | | |  |  |  |  |  | | --- | --- | --- | --- | --- | | |  | | --- | | 29.3\*  Is the design or development of this survey instrument dependent on receipt of funding or hiring of personnel? | |  | | Yes **No** | |  | | |  |  | | --- | --- | | |  | | --- | |  | | |  | |      |  |  |  |  |  |  |  |  |  |  | | --- | --- | --- | --- | --- | --- | --- | --- | --- | --- | | |  |  |  |  |  | | --- | --- | --- | --- | --- | | |  | | --- | | 29.4\*  In what manner will the survey or interview be conducted (e.g., in-person, Internet, mail, telephone, etc.)? *Special Note: For electronic surveys, the eResearch ID number must be included in the informed consent document (uploaded in section 10-1) or other material that serves as the informed consent.* | |  | | in person | |  | | |  |  | | --- | --- | | |  | | --- | |  | | |  | | | |  |  |  |  |  | | --- | --- | --- | --- | --- | | |  | | --- | | 29.5\*  What is the predicted response rate? | |  | | 100 % | |  | | |  |  | | --- | --- | | |  | | --- | |  | | |  | | | |  |  |  |  |  | | --- | --- | --- | --- | --- | | |  | | --- | | 29.6\*  What is the total number of questions? | |  | | 15 | |  | | |  |  | | --- | --- | | |  | | --- | |  | | |  | | | |  |  |  |  |  | | --- | --- | --- | --- | --- | | |  | | --- | | 29.7\*  What is the anticipated cumulative amount of time required for each subject? | |  | | 5 minutes | |  | | |  |  | | --- | --- | | |  | | --- | |  | | |  | | | |  |  |  |  |  | | --- | --- | --- | --- | --- | | |  | | --- | | 29.8\*  What is the total number of interviews/data collection interactions with an individual subject? | |  | | 2 | |  | | |  |  | | --- | --- | | |  | | --- | |  | | |  | | | |  |  |  |  |  | | --- | --- | --- | --- | --- | | |  | | --- | | 29.9\*  Does the survey or interview contain questions of a sensitive nature (e.g., mental illness, sexual abuse, illicit drug use, etc.)? | |  | | Yes **No** | |  | | |  |  | | --- | --- | | |  | | --- | |  | | |  | | | |  |  |  |  |  | | --- | --- | --- | --- | --- | | |  | | --- | | 29.10\*  Is the survey or interview likely to produce psychological discomfort or negative feelings in the subjects? | |  | | Yes **No** | |  | | |  |  | | --- | --- | | |  | | --- | |  | | |  | | | |  |  |  |  |  | | --- | --- | --- | --- | --- | | |  | | --- | | 29.11\*  Has the survey instrument been validated or used in standard practice? | |  | | Yes **No** | |  | | |  |  | | --- | --- | | |  | | --- | |  | | |  | |      |  |  |  |  |  |  |  |  |  |  | | --- | --- | --- | --- | --- | --- | --- | --- | --- | --- | | |  |  |  |  |  | | --- | --- | --- | --- | --- | | |  | | --- | | 29.11.1\*  If yes, describe the origin of the instrument. | |  | |  | |  | | |  |  | | --- | --- | | |  | | --- | |  | | |  | |     |  |  |  |  |  |  |  |  |  |  |  |  |  |  |  | | --- | --- | --- | --- | --- | --- | --- | --- | --- | --- | --- | --- | --- | --- | --- | | |  |  |  |  |  |  |  |  |  |  | | --- | --- | --- | --- | --- | --- | --- | --- | --- | --- | | |  | | --- | | 29.12\*  Upload the survey instrument here. | |  | | |  |  |  |  |  | | --- | --- | --- | --- | --- | | | Name | Version | | --- | --- | | Risk Assessment Control Survey | History | 0.01 | | | |  | | |  |  | | --- | --- | | |  | | --- | |  | | | Additional Help | | |
| Survey Detail     |  |  |  |  |  |  |  |  |  |  | | --- | --- | --- | --- | --- | --- | --- | --- | --- | --- | | |  |  |  |  |  | | --- | --- | --- | --- | --- | | |  | | --- | | 29.2\*  Survey or interview name: | |  | | Semi-structured phone survey | |  | | |  |  | | --- | --- | | |  | | --- | |  | | |  | | | |  |  |  |  |  | | --- | --- | --- | --- | --- | | |  | | --- | | 29.3\*  Is the design or development of this survey instrument dependent on receipt of funding or hiring of personnel? | |  | | Yes **No** | |  | | |  |  | | --- | --- | | |  | | --- | |  | | |  | |      |  |  |  |  |  |  |  |  |  |  | | --- | --- | --- | --- | --- | --- | --- | --- | --- | --- | | |  |  |  |  |  | | --- | --- | --- | --- | --- | | |  | | --- | | 29.4\*  In what manner will the survey or interview be conducted (e.g., in-person, Internet, mail, telephone, etc.)? *Special Note: For electronic surveys, the eResearch ID number must be included in the informed consent document (uploaded in section 10-1) or other material that serves as the informed consent.* | |  | | By phone | |  | | |  |  | | --- | --- | | |  | | --- | |  | | |  | | | |  |  |  |  |  | | --- | --- | --- | --- | --- | | |  | | --- | | 29.5\*  What is the predicted response rate? | |  | | 100 % | |  | | |  |  | | --- | --- | | |  | | --- | |  | | |  | | | |  |  |  |  |  | | --- | --- | --- | --- | --- | | |  | | --- | | 29.6\*  What is the total number of questions? | |  | | 4 | |  | | |  |  | | --- | --- | | |  | | --- | |  | | |  | | | |  |  |  |  |  | | --- | --- | --- | --- | --- | | |  | | --- | | 29.7\*  What is the anticipated cumulative amount of time required for each subject? | |  | | 30-60 minutes | |  | | |  |  | | --- | --- | | |  | | --- | |  | | |  | | | |  |  |  |  |  | | --- | --- | --- | --- | --- | | |  | | --- | | 29.8\*  What is the total number of interviews/data collection interactions with an individual subject? | |  | | 1 | |  | | |  |  | | --- | --- | | |  | | --- | |  | | |  | | | |  |  |  |  |  | | --- | --- | --- | --- | --- | | |  | | --- | | 29.9\*  Does the survey or interview contain questions of a sensitive nature (e.g., mental illness, sexual abuse, illicit drug use, etc.)? | |  | | Yes **No** | |  | | |  |  | | --- | --- | | |  | | --- | |  | | |  | | | |  |  |  |  |  | | --- | --- | --- | --- | --- | | |  | | --- | | 29.10\*  Is the survey or interview likely to produce psychological discomfort or negative feelings in the subjects? | |  | | Yes **No** | |  | | |  |  | | --- | --- | | |  | | --- | |  | | |  | | | |  |  |  |  |  | | --- | --- | --- | --- | --- | | |  | | --- | | 29.11\*  Has the survey instrument been validated or used in standard practice? | |  | | Yes **No** | |  | | |  |  | | --- | --- | | |  | | --- | |  | | |  | |      |  |  |  |  |  |  |  |  |  |  | | --- | --- | --- | --- | --- | --- | --- | --- | --- | --- | | |  |  |  |  |  | | --- | --- | --- | --- | --- | | |  | | --- | | 29.11.1\*  If yes, describe the origin of the instrument. | |  | |  | |  | | |  |  | | --- | --- | | |  | | --- | |  | | |  | |     |  |  |  |  |  |  |  |  |  |  |  |  |  |  |  | | --- | --- | --- | --- | --- | --- | --- | --- | --- | --- | --- | --- | --- | --- | --- | | |  |  |  |  |  |  |  |  |  |  | | --- | --- | --- | --- | --- | --- | --- | --- | --- | --- | | |  | | --- | | 29.12\*  Upload the survey instrument here. | |  | | |  |  |  |  |  | | --- | --- | --- | --- | --- | | | Name | Version | | --- | --- | | Semi-Structured Phone Interview | History | 0.01 | | | |  | | |  |  | | --- | --- | | |  | | --- | |  | | | Additional Help | | |

|  |  |  |
| --- | --- | --- |
| |  |  | | --- | --- | |  |  | |
| 32. Data Safety And Monitoring Plan   |  |  |  | | --- | --- | --- | | |  | | --- | | **Completion of this section is required based on the response provided to question 7-1.10.  The principal investigator (PI) has the ultimate responsibility for the conduct of this research study. The study-specific scientific protocol should include detailed information about tests and procedures employed to safeguard the health and safety of the subjects. Additionally, the PI must prepare a specific data and safety monitoring plan taking into account national guidelines and the study's complexity, risk, and size. The plan should include the administrative processes for recording and evaluating the data quality and integrity. The plan should also specify the responsibilities of research team members and the schedules for reviewing and reporting study progress and adverse events.  Components of this plan relating to the protection of subject privacy and data confidentiality should already have been included in the Confidentiality/Security section of this application.  Additionally, certain members of the research team must complete the PEERRS mandatory training on human subject protection. This includes personnel joining the study team after the initiation of the study.** | | | | |  |  |  |  |  |  |  |  |  |  |  |  | | --- | --- | --- | --- | --- | --- | --- | --- | --- | --- | --- | --- | | |  | | --- | | The Risk Level has been indicated as: | |  | | |  |  |  |  |  |  |  | | --- | --- | --- | --- | --- | --- | --- | | | Name | Risk Level | Direct Benefit | | --- | --- | --- | | HUM00053273 | No more than minimal risk | no | | | |  | | |  |  | | --- | --- | | |  | | --- | |  | | |  | | | |  |  |  |  |  |  |  |  | | --- | --- | --- | --- | --- | --- | --- | --- | | |  | | --- | | 32.1\*  Indicate who will provide study information and instructions to the subjects beyond what is included in the informed consent document. | |  | | |  |  |  | | --- | --- | --- | | | Select all that apply: | | --- | | Study Coordinator/Research Assistant | |   If other, please specify: | |  | | |  |  | | --- | --- | | |  | | --- | |  | | |  | | | |  |  |  |  |  |  |  |  | | --- | --- | --- | --- | --- | --- | --- | --- | | |  | | --- | | 32.2\*  Indicate who will obtain informed consent from the subjects. | |  | | |  |  |  | | --- | --- | --- | | | Select all that apply: | | --- | | Study Coordinator/Research Assistant | |   If other, please specify: | |  | | |  |  | | --- | --- | | |  | | --- | |  | | |  | | | |  |  |  |  |  |  |  |  | | --- | --- | --- | --- | --- | --- | --- | --- | | |  | | --- | | 32.3\*  Indicate who will collect and record study data. | |  | | |  |  |  | | --- | --- | --- | | | Select all that apply: | | --- | | Study Coordinator/Research Assistant | |   If other, please specify: | |  | | |  |  | | --- | --- | | |  | | --- | |  | | |  | | | |  |  |  |  |  |  |  |  |  |  |  |  |  |  |  |  |  |  |  |  |  |  |  |  |  |  |  |  |  |  |  |  |  |  |  |  |  |  |  |  |  |  |  |  |  |  |  |  |  |  |  |  |  |  |  |  |  |  |  |  | | --- | --- | --- | --- | --- | --- | --- | --- | --- | --- | --- | --- | --- | --- | --- | --- | --- | --- | --- | --- | --- | --- | --- | --- | --- | --- | --- | --- | --- | --- | --- | --- | --- | --- | --- | --- | --- | --- | --- | --- | --- | --- | --- | --- | --- | --- | --- | --- | --- | --- | --- | --- | --- | --- | --- | --- | --- | --- | --- | --- | | |  | | --- | | 32.4\*  Indicate what mechanism(s) will be used for monitoring subjects and identifying adverse events. | |  | | |  |  |  |  |  |  |  |  |  |  |  |  |  |  |  |  |  |  |  |  |  |  |  |  |  |  |  |  |  |  |  |  |  |  |  |  |  |  |  |  |  |  |  |  |  |  |  |  |  |  |  |  |  |  |  | | --- | --- | --- | --- | --- | --- | --- | --- | --- | --- | --- | --- | --- | --- | --- | --- | --- | --- | --- | --- | --- | --- | --- | --- | --- | --- | --- | --- | --- | --- | --- | --- | --- | --- | --- | --- | --- | --- | --- | --- | --- | --- | --- | --- | --- | --- | --- | --- | --- | --- | --- | --- | --- | --- | --- | | |  |  |  | | --- | --- | --- | | Mechanism (Select at least one:) | | Conducted by: | |  | Direct interviews/ physical exams conducted by: | |  |  |  |  |  |  |  |  |  |  |  |  | | --- | --- | --- | --- | --- | --- | --- | --- | --- | --- | --- | --- | | | Select all that apply: | | --- | |  |  |  |  |  |  |  |  |  |  | | --- | --- | --- | --- | --- | --- | --- | --- | --- | --- | | There are no items to display | | | | | | | | | | |   If other, please specify | |  | Review of lab work, tests, procedures, etc. by: | |  |  |  |  |  |  |  |  |  |  |  |  | | --- | --- | --- | --- | --- | --- | --- | --- | --- | --- | --- | --- | | | Select all that apply: | | --- | |  |  |  |  |  |  |  |  |  |  | | --- | --- | --- | --- | --- | --- | --- | --- | --- | --- | | There are no items to display | | | | | | | | | | |   If other, please specify | |  | Telephone follow-up conducted by: | |  |  |  |  |  |  |  |  |  |  |  |  | | --- | --- | --- | --- | --- | --- | --- | --- | --- | --- | --- | --- | | | Select all that apply: | | --- | |  |  |  |  |  |  |  |  |  |  | | --- | --- | --- | --- | --- | --- | --- | --- | --- | --- | | There are no items to display | | | | | | | | | | |   If other, please specify | |  | Self-reporting by subject | Instructions must be included in the Informed Consent Document. | |  | Other | If other, please specify Study coordinator will note any AEs | |   Reminder: Adverse Events that come to the attention of any member of the study team must be reported to the PI in a timely manner. | |  | | |  |  | | --- | --- | | |  | | --- | |  | | |  | | |
| 32-1. Data and Safety Monitoring Plan - AE Reporting  **Adverse Event (AE) Reporting**   |  |  |  |  |  |  |  |  |  |  |  |  |  |  |  | | --- | --- | --- | --- | --- | --- | --- | --- | --- | --- | --- | --- | --- | --- | --- | | |  |  |  |  |  |  |  |  |  |  | | --- | --- | --- | --- | --- | --- | --- | --- | --- | --- | | |  | | --- | | 32-1.1\*  Adverse events will be reported to: | |  | | |  |  |  |  |  | | --- | --- | --- | --- | --- | | | Organization | Reporting Mechanism | | --- | --- | | IRB | eResearch AE/ORIO submission | |   If other, please specify: | |  | | |  |  | | --- | --- | | |  | | --- | |  | | |  | | | |  |  |  |  |  | | --- | --- | --- | --- | --- | | |  | | --- | | 32-1.2\*  Indicate the AE reporting timetable that will be used to report adverse events to the IRB: | |  | | Standard IRBMED AE reporting timetable | |  | | |  |  | | --- | --- | | |  | | --- | |  | | |  | |      |  |  |  |  |  |  |  |  |  |  |  |  |  |  |  |  |  |  |  |  |  |  | | --- | --- | --- | --- | --- | --- | --- | --- | --- | --- | --- | --- | --- | --- | --- | --- | --- | --- | --- | --- | --- | --- | | |  |  |  |  |  |  |  |  |  |  |  |  |  |  |  |  |  | | --- | --- | --- | --- | --- | --- | --- | --- | --- | --- | --- | --- | --- | --- | --- | --- | --- | | |  | | --- | | 32-1.2.1\*  Indicate the reason for selecting the study-specific AE reporting rather than the Standard IRBMED AE Reporting Timetable. | |  | | |  |  |  |  |  |  |  |  |  |  |  |  | | --- | --- | --- | --- | --- | --- | --- | --- | --- | --- | --- | --- | | | Select all that apply: | | --- | |  |  |  |  |  |  |  |  |  |  | | --- | --- | --- | --- | --- | --- | --- | --- | --- | --- | | There are no items to display | | | | | | | | | | |   If other, please specify: | |  | | |  |  | | --- | --- | | |  | | --- | |  | | |  | | | |  |  |  |  |  |  |  |  |  |  |  |  |  |  |  |  |  |  | | --- | --- | --- | --- | --- | --- | --- | --- | --- | --- | --- | --- | --- | --- | --- | --- | --- | --- | | |  | | --- | | 32-1.2.2\*  Provide the study-specific AE reporting plan.   If the study-specific AE reporting plan is included in the previously uploaded scientific protocol, indicate section. | |  | | |  |  |  |  |  |  |  |  |  |  |  |  |  | | --- | --- | --- | --- | --- | --- | --- | --- | --- | --- | --- | --- | --- | | | Name | Version | | --- | --- | |  |  |  |  |  |  |  |  |  |  | | --- | --- | --- | --- | --- | --- | --- | --- | --- | --- | | There are no items to display | | | | | | | | | | |   Indicate specific protocol section here: | |  | | |  |  | | --- | --- | | |  | | --- | |  | | | Additional Help | |     |  |  |  |  |  |  |  |  |  |  | | --- | --- | --- | --- | --- | --- | --- | --- | --- | --- | | |  |  |  |  |  | | --- | --- | --- | --- | --- | | |  | | --- | | 32-1.3\*  Affirm that the adverse events will be reported to the IRB according to the following generalized AE GRADING SCALE: | |  | | - 0 - No adverse event- 1 - Mild AE – No treatment needed- 2 - Moderate AE – Resolved with treatment- 3 - Severe AE – Inability to carry on normal activities, required professional medical attention- 4 - Life-threatening or disabling AE- 5 - Fatal AE | |  | | |  |  | | --- | --- | | |  | | --- | |  | | |  | | | |  |  |  |  |  | | --- | --- | --- | --- | --- | | |  | | --- | | 32-1.4\*  Will Serious Adverse Events (SAEs) be categorized according to the following FDA definition? | |  | | Yes   - Death- A life-threatening adverse drug experience- Inpatient hospitalization or prolongation of existing hospitalization- A persistent or significant disability/incapacity- A congenital anomaly/birth defect- Important medical events that may not result in death, be life-threatening, or require hospitalization may be considered a serious adverse drug experience when, based upon appropriate medical judgment, they may jeopardize the patient or subject and may require medical or surgical intervention to prevent one of the outcomes listed in this definition. Examples of such medical events include allergic bronchospasm requiring intensive treatment in an emergency room or at home, blood dyscrasias or convulsions that do not result in inpatient hospitalization, or the development of drug dependency or drug abuse. | |  | | |  |  | | --- | --- | | |  | | --- | |  | | |  | | | |  |  |  |  |  | | --- | --- | --- | --- | --- | | |  | | --- | | 32-1.5\*  Affirm that either the principal investigator or a co-investigator will determine the ATTRIBUTION/RELATEDNESS for each adverse event. | |  | | - Definitely related- Probably related- Possibly related- Unlikely to be related- Definitely not related | |  | | |  |  | | --- | --- | | |  | | --- | |  | | |  | | | |  |  |  |  |  | | --- | --- | --- | --- | --- | | |  | | --- | | 32-1.6\*  Affirm that the EXPECTEDNESS will be assigned for each adverse event according to the following definitions: | |  | | - Unexpected adverse events (i.e., has NOT been addressed or described in one or more of the following: Informed consent document(s) for this study, IRB application for this study, grant application or study agreement, protocol or procedures for this study, investigators' brochure or equivalent (for FDA regulated drugs or devices), DSMB/DSC Reports, published literature, other documentation)- Expected adverse events (i.e., has been addressed or described in one or more of the following: Informed consent document(s) for this study, IRB application for this study, grant application or study agreement, protocol or procedures for this study, investigators' brochure or equivalent (for FDA regulated drugs or devices), DSMB/DSC Reports, published literature, other documentation, or characteristics of the study population) | |  | | |  |  | | --- | --- | | |  | | --- | |  | | |  | | |
| 32-2. Data Safety and Monitoring Plan - Monitoring the Study  **Monitoring the Study**   |  |  |  |  |  |  |  |  |  |  | | --- | --- | --- | --- | --- | --- | --- | --- | --- | --- | | |  |  |  |  |  | | --- | --- | --- | --- | --- | | |  | | --- | | 32-2.1\*  Indicate the frequency with which the study team will conduct scheduled assessments of study recruitment, data integrity and quality, adverse events, withdrawals, and compliance with protocol plan. | |  | | Quarterly  If other, please specify: | |  | | |  |  | | --- | --- | | |  | | --- | |  | | |  | |      |  |  |  |  |  |  |  |  |  |  | | --- | --- | --- | --- | --- | --- | --- | --- | --- | --- | | |  |  |  |  |  | | --- | --- | --- | --- | --- | | |  | | --- | | 32-2.1.1\*  If quarterly or other, provide a justification for the assessment schedule. | |  | | Very low risk study with no medical intervention | |  | | |  |  | | --- | --- | | |  | | --- | |  | | |  | |     |  |  |  |  |  |  |  |  |  |  |  |  |  | | --- | --- | --- | --- | --- | --- | --- | --- | --- | --- | --- | --- | --- | | |  |  |  |  |  |  |  |  | | --- | --- | --- | --- | --- | --- | --- | --- | | |  | | --- | | 32-2.2\*  Study oversight and safety monitoring may be required based on the nature, size, and complexity of the study.  Indicate the responsible entities. | |  | | |  |  |  | | --- | --- | --- | | | Select all that apply: | | --- | | No additional monitoring is required – the nature, size, and complexity of this study does not require additional safety monitoring to that provided by the IRB. | |   If other, please specify:  If no additional monitoring is required, jump to 32-2.3. | |  | | |  |  | | --- | --- | | |  | | --- | |  | | |  | | | |  |  |  |  |  | | --- | --- | --- | --- | --- | | |  | | --- | | 32-2.2.1  Provide the names and areas of expertise of those providing this additional monitoring | |  | |  | |  | | |  |  | | --- | --- | | |  | | --- | |  | | |  | | | |  |  |  |  |  | | --- | --- | --- | --- | --- | | |  | | --- | | 32-2.2.2  Indicate the frequency with which the additional monitoring activities will be conducted. | |  | | If other, please specify: | |  | | |  |  | | --- | --- | | |  | | --- | |  | | |  | | | |  |  |  |  |  |  |  |  |  |  |  |  |  |  |  |  |  | | --- | --- | --- | --- | --- | --- | --- | --- | --- | --- | --- | --- | --- | --- | --- | --- | --- | | |  | | --- | | 32-2.2.3  Indicate the data that will be reviewed. | |  | | |  |  |  |  |  |  |  |  |  |  |  |  | | --- | --- | --- | --- | --- | --- | --- | --- | --- | --- | --- | --- | | | Select all that apply: | | --- | |  |  |  |  |  |  |  |  |  |  | | --- | --- | --- | --- | --- | --- | --- | --- | --- | --- | | There are no items to display | | | | | | | | | | | | |  | | |  |  | | --- | --- | | |  | | --- | |  | | |  | | | |  |  |  |  |  |  |  |  |  |  |  |  |  |  |  |  |  |  | | --- | --- | --- | --- | --- | --- | --- | --- | --- | --- | --- | --- | --- | --- | --- | --- | --- | --- | | |  | | --- | | 32-2.2.4  If a DSMB or DSC charter exists, upload it here. | |  | | |  |  |  |  |  |  |  |  |  |  |  |  |  | | --- | --- | --- | --- | --- | --- | --- | --- | --- | --- | --- | --- | --- | | | Name | Version | | --- | --- | |  |  |  |  |  |  |  |  |  |  | | --- | --- | --- | --- | --- | --- | --- | --- | --- | --- | | There are no items to display | | | | | | | | | | | | |  | | |  |  | | --- | --- | | |  | | --- | |  | | | Additional Help | | | |  |  |  |  |  |  |  |  |  |  | | --- | --- | --- | --- | --- | --- | --- | --- | --- | --- | | |  | | --- | | 32-2.3\*  Monitoring reports will be provided to: | |  | | |  |  |  |  |  | | --- | --- | --- | --- | --- | | | Organization | Reporting Mechanism | | --- | --- | | IRB (required) | eResearch AE/ORIO submission | |   If other, please specify: | |  | | |  |  | | --- | --- | | |  | | --- | |  | | |  | | |

|  |  |  |
| --- | --- | --- |
| |  |  | | --- | --- | |  |  | |
| 37. Women of Child Bearing Potential  **Completion of this section is required based on the response provided to question 9-1.1.**   |  |  |  |  |  |  |  |  |  |  | | --- | --- | --- | --- | --- | --- | --- | --- | --- | --- | | |  |  |  |  |  | | --- | --- | --- | --- | --- | | |  | | --- | | 37.1\*  Is there a potential that any of the study procedures pose significant physical or psychological risks to women who are or may be pregnant, or to a fetus? | |  | | Yes **No** | |  | | |  |  | | --- | --- | | |  | | --- | |  | | |  | |      |  |  |  |  |  |  |  |  |  |  | | --- | --- | --- | --- | --- | --- | --- | --- | --- | --- | | |  |  |  |  |  | | --- | --- | --- | --- | --- | | |  | | --- | | 37.1.1\*  List the study procedures that may pose risks to pregnant women or fetuses. | |  | |  | |  | | |  |  | | --- | --- | | |  | | --- | |  | | |  | | | |  |  |  |  |  | | --- | --- | --- | --- | --- | | |  | | --- | | 37.1.2\*  Describe the steps that will be taken prior to the conduct of these procedures to confirm that subjects are not pregnant. | |  | |  | |  | | |  |  | | --- | --- | | |  | | --- | |  | | |  | | | |  |  |  |  |  | | --- | --- | --- | --- | --- | | |  | | --- | | 37.1.3\*  Describe the measures that will be required to prevent pregnancy during or, if applicable, following subjects' exposure to the study procedures. Specify the duration of the preventative measures. | |  | |  | |  | | |  |  | | --- | --- | | |  | | --- | |  | | |  | | |

|  |  |  |
| --- | --- | --- |
| |  |  | | --- | --- | |  |  | |
| 44. Additional Supporting Documents     |  |  |  |  |  |  |  |  |  |  |  |  |  |  |  |  |  |  |  |  |  | | --- | --- | --- | --- | --- | --- | --- | --- | --- | --- | --- | --- | --- | --- | --- | --- | --- | --- | --- | --- | --- | | |  |  |  |  |  |  |  |  |  |  |  |  |  |  |  |  | | --- | --- | --- | --- | --- | --- | --- | --- | --- | --- | --- | --- | --- | --- | --- | --- | | |  | | --- | | 44.1  Please upload any additional supporting documents related to your study that have not already been uploaded. Examples include, but are not limited to, data collection sheets, newsletters, subject brochures, and instructional brochures. | |  | | |  |  |  |  |  |  |  |  |  |  |  | | --- | --- | --- | --- | --- | --- | --- | --- | --- | --- | --- | | | Name | Version | | --- | --- | | Colorectal Website | History | 0.02 | | Documenting Study Visit Time CRF | History | 0.01 | | Kelly\_Blake\_SUPPLEMENT-1.pdf | History | 0.01 | | Lafata certification | History | 0.01 | | | |  | | |  |  | | --- | --- | | |  | | --- | |  | | |  | |      |  |  |  |  |  |  |  |  |  |  | | --- | --- | --- | --- | --- | --- | --- | --- | --- | --- | | |  |  |  |  |  | | --- | --- | --- | --- | --- | | |  | | --- | | 44.2  If the study sponsor requires that the IRBMED approval letter contain a list of supporting documents, list the names of the documents in the box below as they should appear on the IRBMED approval letter: | |  | |  | |  | | |  |  | | --- | --- | | |  | | --- | |  | | |  | | |

|  |  |  |
| --- | --- | --- |
| |  |  | | --- | --- | |  |  | |
| |  |  | | --- | --- | | |  | | --- | | 45. End of Application |             The form was successfully submitted. Click 'Exit' or 'Finish' to leave the form. | |
